# The Use of Aryl-Substituted Homophthalic Anhydrides in the Castagnoli–Cushman Reaction Provides Access to Novel Tetrahydroisoquinolone Carboxylic Acid Bearing an All-Carbon Quaternary Stereogenic Center

**DOI:** 10.3390/molecules27238462

**Published:** 2022-12-02

**Authors:** Nazar Moshnenko, Alexander Kazantsev, Olga Bakulina, Dmitry Dar’in, Mikhail Krasavin

**Affiliations:** 1 Institute of Chemistry, Saint Petersburg State University, 26 Universitetskii Prospect, 198504 Peterhof, Russia; 2 Institute of Living Systems, Immanuel Kant Baltic Federal University, 236041 Kaliningrad, Russia

**Keywords:** homophthalic anhydride, imine, Castagnoli–Cushman reaction, tetrahydroisoquinolone, lactam, all-carbon quaternary atom

## Abstract

Novel aryl-substituted homophthalic acids were cyclodehydrated to the respective homophthalic anhydrides for use in the Castagnoli–Cushman reaction. With a range of imines, this reaction proceeded smoothly and delivered hitherto undescribed 4-aryl-substituted tetrahydroisoquinolonic acids with remarkable diastereoselectivity, good yields and no need for chromatographic purification. These findings significantly extend the range of cyclic anhydrides employable in the Castagnoli–Cushman reaction and signify access to a novel substitution pattern around the medicinally relevant tetrahydroisoquinolonic acid scaffold.

## 1. Introduction

**Figure 1:**
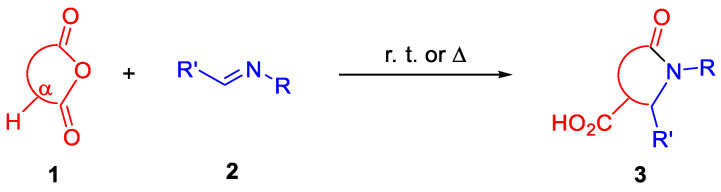
The Castagnoli–Cushman reaction.

The Castagnoli–Cushman reaction (CCR) [1] is a remarkably versatile [4 + 2]-type cyclocondensation of a-C-H-acidic cyclic anhydrides **1** with imines **2** leading, depending on the specific anhydride employed [2], to skeletally diverse [3] lactams **3** bearing multiple substituents, which in many cases proceeds in diastereoselective fashion. This reaction is multicomponent in nature because the requisite imine can be generated in situ from the respective amine and aldehyde [4], which makes this reaction particularly suitable for generating compound libraries in array format for drug discovery (Figure 1). 

Considering the fact that the cyclic anhydride (**1**) for the CCR input primarily controls the skeletal nature of the lactam product **3**, involvement of novel anhydrides in the reaction promises to deliver molecular frameworks which are either completely novel [5] or carry unprecedented substitution patterns around known cores. 

**Figure 2:**
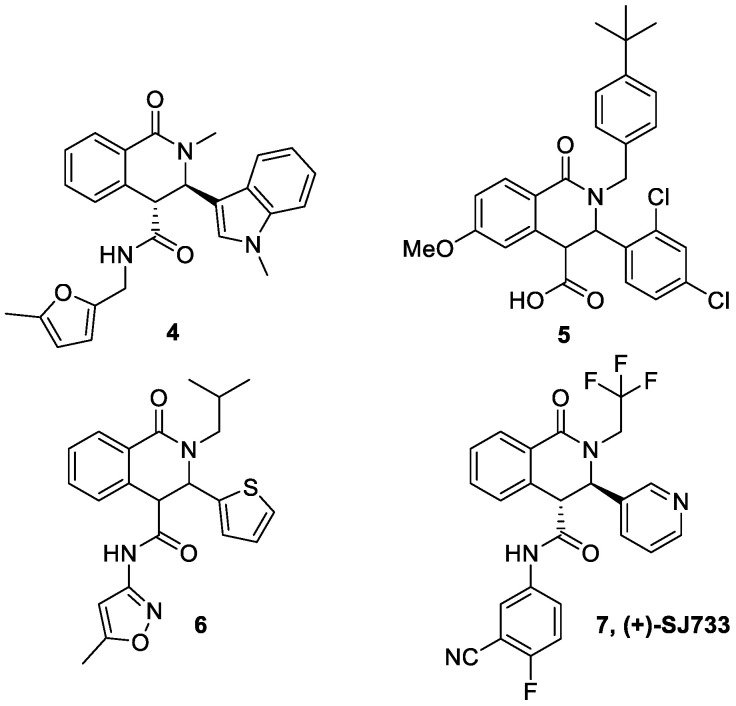
Examples of diversely biologically active tetrahydroisoquinolones.

Homophthalic anhydride (HPA) is one of the most popular and most reactive anhydrides used in the CCR. The reaction with HPA delivers tetrahydroisoquinolones (THIQs) with good control of diastereoselectivity [6,7,8]. The THIQ scaffold is of undisputable medicinal relevance, as evidenced by various molecular series possessing diverse biological activities reported in the literature. These can be exemplified by such compounds as adrenocorticotropic hormone receptor modulator **4** [9], apoptosis regulator **5** [10], trypanocidal cysteine protease inhibitor **6** [11], as well as antimalarial **7** [12] (Figure 2). 

**Figure 3:**
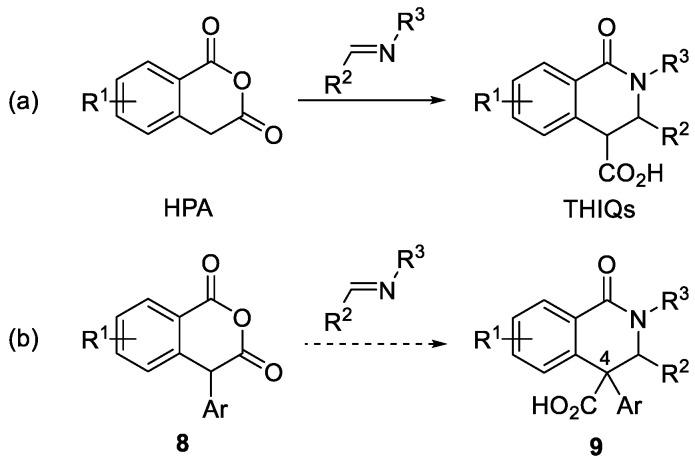
(**a**) Traditional CCR of HPA. (**b**) Synthetic goal pursued in this work.

The peripheral group diversity of HPA has been largely limited to the substitutions in the benzene ring [13], while substitutions at the methylene position remain almost completely unexplored except for methyl- [14,15] and benzyl- [15] substituted variants. We became interested in synthesizing novel HPA versions bearing an aryl group at the methylene linker (**8**) and exploring them as partners in the CCR. Our interest was fueled by the prospect of obtaining, possibly in diastereoselective manner, densely substituted THIQs **9** where the α-position (position 4 of the THIQ scaffold) of the hitherto undescribed carboxylic acid would be an all-carbon stereogenic center (Figure 3). Herein, we present the results obtained in the course of pursuing this goal.

## 2. Results

**Scheme 1:**
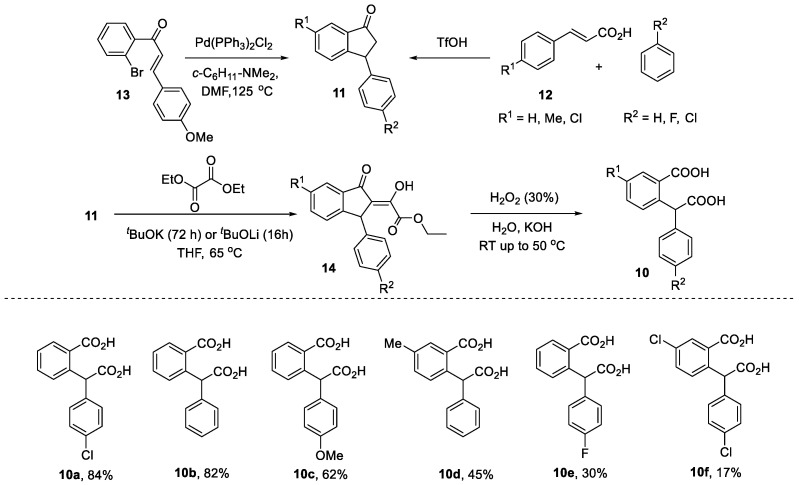
Synthesis of substituted homophthalic acids **10**.

4-Aryl-substituted homophthalic acids **10** required for the preparation of anhydrides **8** were synthesized from indanones **11**. These, in turn, were prepared either by triflic acid-promoted arylation of cinnamic acids **12** [16] or by intramolecular Heck reaction of bromochalcone **13** [17]. The Heck reaction approach was used for the methoxy-substituted substrate because the respective TfOH-promoted arylation, when attempted, led to extensive tar formation. Indanones **11** were condensed with diethyl oxalate using either potassium or lithium *tert*-butoxide as the base, and the resulting condensation products **14** were oxidized with hydrogen peroxide in basic medium (as described previously [18]) to furnish novel homophthalic acids **10a**–**f** in modest to excellent yields over two steps from indanones **11** (Scheme 1).

For the prospective employment of homophthalic acids in the CCR, anhydrides **8** were prepared immediately before the reaction using acetic anhydride as the cyclodehydrating agent and were used in the condensation with imines without further purification. For the preparation of anhydrides from homophthalic acids **10a**–**d,** the cyclodehydration was performed at room temperature in dichloromethane. For substrates **10e**–**f**, due to limited solubility in the latter conditions, the same reaction was performed in toluene at 80 ℃.

**Scheme 2:**
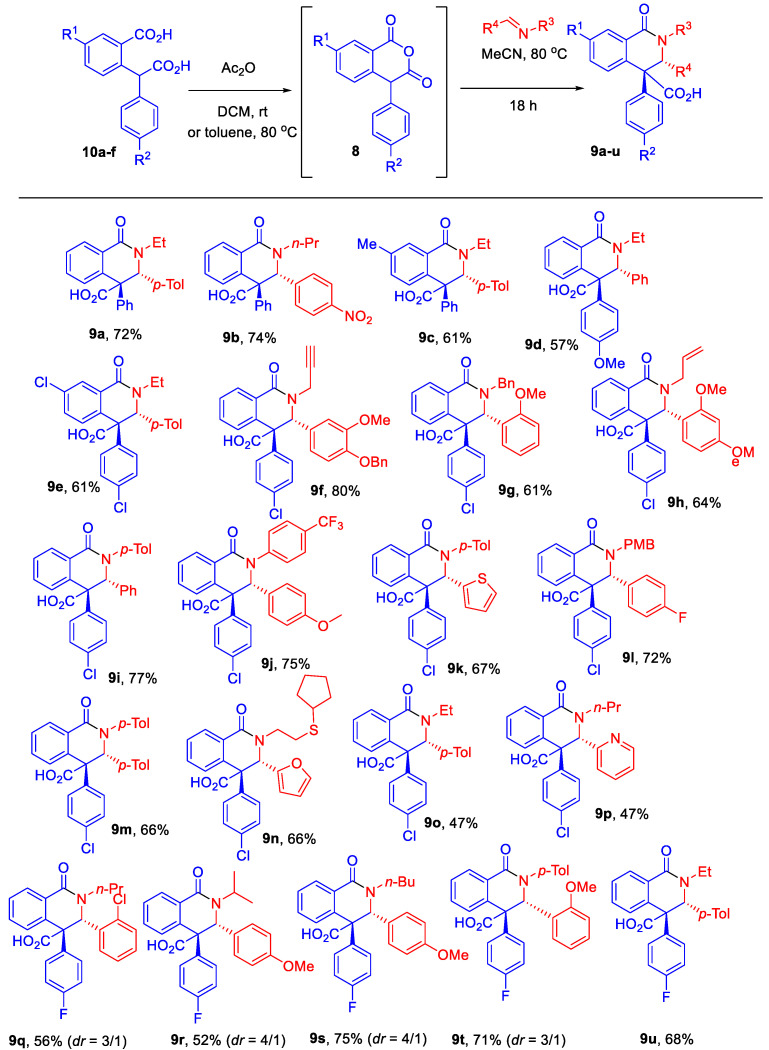
The CCR of cyclic anhydrides **8** with imines.

Although the CCR of HPA can be conducted in a range of different solvents [19], after brief optimization, we found the reaction of anhydride derived from unsubstituted diacid **10b** to furnish an optimum 72% yield of THIQ cycloadduct **9a** as a single diastereomer after refluxing the reaction partners in acetonitrile over 18 h. The same reaction conducted in refluxing toluene gave lower (66%) yield. Interestingly, the reaction in acetonitrile also proceeded to completion at room temperature but with lower yield (55%) and lower diastereoselectivity (*dr* 5:1, *trans*-/*cis*-). Thus, the conditions involving refluxing acetonitrile were extended to anhydrides **8** of this and other homophthalic acids **10** in combination with various imines prepared from aromatic aldehydes (Scheme 2).

**Figure 4:**
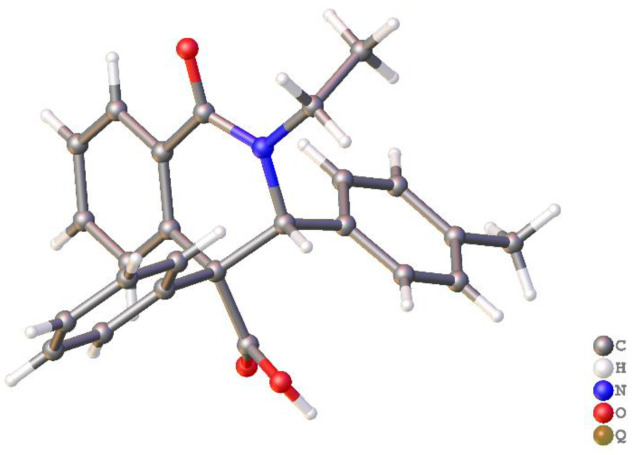
Crystallographic structure of compound **9a** (ORTEP plot, 50% probability level).

The yields of 4-aryl-substituted THIQ acids **9a**–**u** were generally good after simple evaporation of acetonitrile and trituration of the crude material with hexane and ether, with no need for chromatographic purification. The reactions were completely diastereoselective throughout except for those yielding products **9q**–**t**. The stereochemical identity of products **9a**–**u** was unequivocally confirmed as being *trans* with respect to the vicinal aryl groups by single-crystal *X*-ray analysis of compound **9a** (Figure 4, see ESI for details). The substituents in the homophthalic portion did not apparently influence the reaction outcome. The scope of the reaction was also quite broad with respect to the aromatic, aldehyde-derived group tolerating heterocyclic motifs as well as phenyl group with a nitro group. Likewise, the scope of amines, aromatic and aliphatic alike, was also fairly broad.

Despite our initial expectations of potentially lower reactivity of anhydrides **8** in the CCR due to increased steric bulk compared to HPA, the reactivity of these anhydrides was similar to that of HPA (considering the fact that the reaction also proceeded at room temperature, vide supra). This is in line with the observations by others for methyl- and benzyl-substituted versions of HPA [15].

**Scheme 3:**
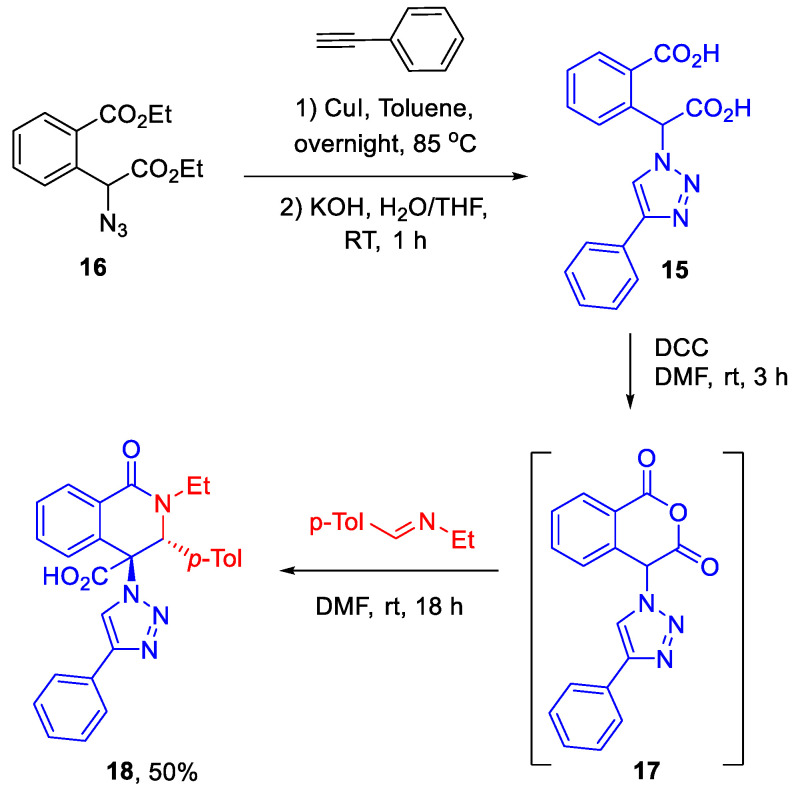
Preparation and use of 1,2,3-triazol-1-yl-substituted cyclic anhydride **17** in the CCR.

In addition to dicarboxylic acids **10a**–**f**, we prepared 1,2,3-triazol-1-yl-substituted dicarboxylic acid **15** by copper-catalyzed [3 + 2] azide-alkyne cycloaddition of the known [20] azido-substituted homophthalic diethyl ester **16** and phenylacetylene followed by hydrolysis. Due to solubility issues, the cyclodehydration procedure to anhydride **17** was modified, and the reaction was performed in DMF using dicyclohexylcarbodiimide (DCC) as the cyclodehydrating agent. Anhydride **17** proved to be a competent substrate for the CCR; however, due to low solubility of **17** in acetonitrile, the reaction was conducted in DMF at room temperature. *Trans*-configured cycloadduct **18** was obtained as a single diastereomer in 50% yield, also with no need for chromatographic purification (Scheme 3).

## 3. Conclusions

We have described the synthesis of novel aryl-substituted homophthalic acids. Their cyclodehydration to the respective homophthalic anhydrides and the Castagnoli–Cushman reaction of the latter with a range of imines resulted in good yields and delivered hitherto undescribed 4-aryl-substituted tetrahydroisoquinolonic acids with remarkable diastereoselectivity, good yields and no need for chromatographic purification. These products are distinct in that they contain an all-carbon quaternary stereogenic centers in the α-position to the carboxylic acid. The cyclodehydration–Castagnoli–Cushman reaction protocol was found to be also transferrable to a novel 1,2,3-triazol-1-yl-substituted homophthalic acid. These findings significantly extend the range of cyclic anhydrides employable in the Castagnoli–Cushman reaction and signify access to a novel substitution pattern around the medicinally relevant tetrahydroisoquinolonic acid scaffold.

## 4. Materials and Methods

### 4.1. General Information

All reagents were obtained from commercial sources and used without further purification. Acetonitrile, toluene and N,N-dimethylformamide were distilled from suitable drying agents (CaH_2_ or P_2_O_5_) and stored over MS 4Å. Mass spectra were recorded with a Bruker Maxis HRMS-ESI-qTOF spectrometer (Moscow, Russia) (electrospray ionization mode). NMR data were recorded with Bruker Avance 400/500 spectrometer (Moscow, Russia) (400.13 MHz for ^1^H, 100.61 MHz and 125.73 MHz for ^13^C and 376.50 MHz for ^19^F) in DMSO-d_6_ and were referenced to residual solvent proton peaks (δH = 2.51 ppm) and solvent carbon peaks (δC = 39.52 ppm). NMR and HRMS spectra are in the Supplementary Material.

### 4.2. Preparation of Arylhomophthalic Acids ***10a**–**10f***: General Procedure ***1***

Step 1. Condensation of arylindanones with diethyl oxalate

Compounds **10a**,**b**,**d**,**e**: Corresponding indanone (9.6 mmol, 1 equiv.) and diethyl oxalate (4.2 g, 3.9 mL, 28.8 mmol, 3 equiv.) were dissolved in THF (10 mL, dry) in a round-bottom flask, and to the resulting solution a suspension of *t*-BuOK (3.23 g, 28.8 mmol, 3 equiv.) in THF (15 mL, dry) at room temperature was added dropwise. Next, the flask was stoppered, and the mixture was heated in a metal heating block at 65 °C for 72 h (conversion was estimated by TLC, using DCM as an eluent). After cooling to room temperature, the solvent was evaporated and the mixture was dissolved in CHCl_3_ (30 mL), washed with 3% hydrochloric acid solution (1 × 15 mL), water (1 × 15 mL) and brine (1 × 15 mL), then organic layer was dried over anhydrous sodium sulfate. The solvent was evaporated, and the resulting mixture was used in the next step without purification. Compounds **10c**,**f** were obtained according to nearly the same procedure (but using *t*-BuOLi instead of *t*-BuOK), and the heating was performed for 16h.

Step 2. Oxidation

A solution of KOH (3.76 g, 67.2 mmol, 7 equiv.) in water (20 mL) was added to the product of the previous step in a round-bottom flask; the mixture was stirred for 20 min, then H_2_O_2_ (30%, 27.2 mL) was added dropwise. The solution was stirred overnight at room temperature, then heated in a metal heating block to 50 °C and stirred for two hours (until the mixture became transparent). Activated charcoal (12 g) (powder−100 particle size (mesh)) was added to the resulting chilled solution and intensively stirred for 15 min. The solution was filtered through zeolite, and a solution of concentrated hydrochloric acid was added to the filtrate at room temperature to reach pH 1. The precipitated acid was extracted into EtOAc (3 × 30 mL). The organic layer was combined, dried over anhydrous sodium sulfate and evaporated. The resulting acids **10a**–**e** did not require further purification. The acid **10f** was additionally crystallized from acetonitrile. Yields of compounds **10** were calculated for 2 steps.

#### 4.2.1. 2-[Carboxy(4-chlorophenyl)methyl]benzoic Acid (**10a**)

Prepared according to the general procedure GP1 from 3-(4-chlorophenyl)-2,3-dihydro-1*H*-inden-1-one[21]. Yield 2.344 g, 84%. Colorless amorphous solid. ^1^H NMR (400 MHz, DMSO-*d*_6_) *δ* 12.89 (s, 2H), 8.02–7.80 (m, 1H), 7.55–7.48 (m, 1H), 7.46–7.34 (m, 3H), 7.31–7.23 (m, 2H), 7.15–7.10 (m, 1H), 5.99 (s, 1H). ^13^C NMR (101 MHz, DMSO-*d*_6_) *δ* 173.6, 169.0, 140.1, 138.5, 132.3, 132.1, 131.4, 131.0, 130.9, 130.2, 128.9, 127.5, 52.9. HRMS (ESI/Q-TOF) *m/z*: [M + Na^+^]^+^ Calcd for C_15_H_11_ClO_4_Na^+^ 313.0238; Found 313.0234.

#### 4.2.2. 2-[Carboxy(phenyl)methyl]benzoic Acid (**10b**)

Prepared according to the general procedure GP1 from 3-phenyl-2,3-dihydro-1*H*-inden-1-one [16]. Yield 2.017 g, 82%. Colorless amorphous solid. ^1^H NMR (400 MHz, DMSO-*d*_6_) *δ* 12.81 (s, 2H), 7.95–7.82 (m, 1H), 7.53–7.44 (m, 1H), 7.41–7.32 (m, 3H), 7.32–7.22 (m, 3H), 7.13–7.04 (m, 1H), 5.97 (s, 1H). ^13^C NMR (101 MHz, DMSO-*d*_6_) *δ* 173.9, 169.1, 140.6, 139.5, 132.1, 131.1, 130.7, 130.3, 129.5, 129.0, 127.4, 127.3, 53.6. HRMS (ESI/Q-TOF) *m*/*z*: [M + Na^+^]^+^ Calcd for C_15_H_12_O_4_Na^+^ 279.0628; Found 279.0623.

#### 4.2.3. 2-[Carboxy(4-methoxyphenyl)methyl]benzoic Acid (**10c**)

Prepared according to the general procedure GP1 from 3-(4-methoxyphenyl)-2,3-dihydro-1H-inden-1-one [21]. Yield 703 mg, 62%. Colorless amorphous solid. ^1^H NMR (400 MHz, DMSO-d_6_) δ 12.75 (s, 2H), 7.86 (dd, J = 7.7, 1.6 Hz, 1H), 7.46 (td, J = 7.5, 1.6 Hz, 1H), 7.33 (t, J = 7.5 Hz, 1H), 7.15 (d, J = 8.7 Hz, 2H), 7.08 (d, J = 7.7 Hz, 1H), 6.92 (d, J = 8.7 Hz, 2H), 5.88 (s, 1H), 3.74 (s, 3H). ^13^C NMR (101 MHz, DMSO-d_6_) δ 173.7, 168.7, 158.2, 140.6, 131.5, 130.9, 130.6, 130.2, 130.1, 129.7, 126.7, 113.9, 55.1, 52.3. HRMS (ESI/Q-TOF) *m*/*z*: [M-H]^−^ Calcd for C_16_H_13_O_5_^−^ 285.0769; Found 285.0768.

#### 4.2.4. 2-[Carboxy(phenyl)methyl]-5-methylbenzoic Acid (**10d**)

Prepared according to the general procedure GP1 from 6-methyl-3-phenyl-2,3-dihydro-1*H*-inden-1-one [16]. Yield 1.167 g, 45%. Colorless amorphous solid. ^1^H NMR (400 MHz, DMSO-*d*_6_) *δ* 12.71 (s, 2H), 7.71–7.65 (m, 1H), 7.38–7.32 (m, 2H), 7.30–7.26 (m, 2H), 7.25–7.19 (m, 2H), 7.05–6.89 (m, 1H), 5.91 (s, 1H), 2.31 (s, 3H). ^13^C NMR (101 MHz, DMSO-*d*_6_) *δ* 174.00, 169.17, 161.41, 139.66, 137.68, 136.58, 132.61, 131.11, 130.88, 130.24, 129.44, 128.92, 127.30, 53.20, 20.78. ^13^C NMR (101 MHz, DMSO-*d*_6_) *δ* 174.0, 169.2, 161.4, 139.7, 137.7, 136.6, 132.6, 131.1, 130.9, 130.2, 129.4, 128.9, 127.3, 53.2, 20.8. HRMS (ESI/Q-TOF) *m*/*z*: [M + Na]^+^ Calcd for C_16_H_14_O_4_Na^+^ 293.0784; Found 293.0785.

#### 4.2.5. 2-[Carboxy(4-fluorophenyl)methyl]benzoic Acid (**10e**)

Prepared according to the general procedure GP1 from 3-(4-fluorophenyl)-2,3-dihydro-1*H*-inden-1-one [16]. Yield 0.789 g, 30%. Colorless amorphous solid. ^1^H NMR (400 MHz, DMSO-*d*_6_) *δ* 12.88 (s, 2H), 7.94–7.83 (m, 1H), 7.53–7.45 (m, 1H), 7.40–7.33 (m, 1H), 7.31–7.25 (m, 2H), 7.23–7.09 (m, 3H), 6.06–5.94 (m, 1H). ^13^C NMR (101 MHz, DMSO-*d*_6_) *δ* 173.8, 169.1, 161.6 (d, *J* = 243.3 Hz), 140.4, 135.7 (d, *J* = 3.1 Hz), 132.2, 131.4 (d, *J* = 8.1 Hz), 131.1, 130.8, 130.1, 127.4, 115.7 (d, *J* = 21.3 Hz), 52.8. ^19^F NMR (376 MHz, DMSO-*d*_6_) *δ* −115.9. HRMS (ESI/Q-TOF) *m*/*z*: [M + Na^+^]^+^ Calcd for C_15_H_11_FO_4_Na^+^ 297.0534; Found 297.0528.

#### 4.2.6. 2-[Carboxy(4-chlorophenyl)methyl]-5-chlorobenzoic Acid (**10f**)

Prepared according to the general procedure GP1 from 6-chloro-3-(4-chlorophenyl)-2,3-dihydro-1*H*-inden-1-one [22]. Yield 530 mg, 17%. Colorless amorphous solid. ^1^H NMR (400 MHz, DMSO-*d*_6_) *δ* 13.38 (s, 1H), 12.87 (s, 1H), 7.91–7.78 (m, 1H), 7.65–7.53 (m, 1H), 7.46–7.36 (m, 2H), 7.30–7.22 (m, 2H), 7.12–7.04 (m, 1H), 5.91 (s, 1H). ^13^C NMR (101 MHz, DMSO-*d*_6_) *δ* 173.3, 167.7, 139.1, 138.0, 133.0, 132.3, 132.3, 132.1, 132.0, 131.4, 130.3, 129.1, 52.6. HRMS (ESI/Q-TOF) *m*/*z*: [M + Na^+^]^+^ Calcd for C_15_H_10_Cl_2_O_4_Na^+^ 346.9848; Found 346.9841.

### 4.3. General Procedure for Preparation of Tetrahydroisoquinonolones ***9a**–**9u***

Step 1. Anhydride synthesis.

*Products***9a**–**c***and***f**–**u**:

Diacid **10a**–**c**,**f** (50 mg) was mixed with DCM (1 mL, dry.) in a screw-cap vial, after which acetic anhydride (6 equiv.) was added to the suspension and the reaction mixture was stirred overnight at room temperature. Then, the solvent was evaporated in vacuo. The resulting crude anhydride was used in the next step without purification or characterization.

*For products***9d**,**e***:*

Diacid 10c,f (50 mg) was dissolved in toluene (3 mL, dry) in screw-cap vial, after which acetic anhydride (6 equiv.) was added to the suspension and the reaction mixture was stirred overnight at 80 °C in a metal heating box. Then, the solvent was evaporated in vacuo. The resulting crude anhydride was used in the next step without further purification.

Step 2. The Castagnoli–Cushman reaction

*For products***9a**–**9u**:

The resulting crude anhydride from the previous step was dissolved in MeCN (0.3 mL, dry) in a screw-cap vial, then imine (1.05 equiv.) dissolved in MeCN (0.2 mL, dry) was added with stirring. The reaction mixture was kept at 80 °C overnight in a metal heating block. Then, the solvent was evaporated. Next, the crude product was treated with diethyl ether (1 mL), after which pentane (3 mL) was added and the solid was thoroughly ground. After cooling to −20 °C for 20 min, the liquid was decanted. The resulting solid was dried in vacuo to give pure title compound.

Dr values were calculated from integrals of methine protons (^1^H NMR spectra) from lactam ring.

#### 4.3.1. (±)-(3R,4R)-2-Ethyl-1-oxo-4-phenyl-3-(p-tolyl)-1,2,3,4-tetrahydroisoquinoline-4-carboxylic Acid (**9a**)

Prepared according to the general procedure GP2 from **10b** and *N*-(4-methylbenzylidene)ethanamine. Yield 52 mg, 72%. Colorless amorphous solid. ^1^H NMR (400 MHz, DMSO-*d*_6_) *δ* 13.14 (s, 1H), 8.19–8.10 (m, 1H), 8.05–7.98 (m, 1H), 7.64 (s, 1H), 7.59–7.47 (m, 1H), 7.36–7.26 (m, 4H), 7.26–7.21 (m, 1H), 6.98 (s, 4H), 5.59 (s, 1H), 3.58 (dq, *J* = 14.0, 7.1 Hz, 1H), 3.20 (dq, *J* = 14.0, 7.1 Hz, 1H), 2.21 (s, 3H), 0.76 (t, *J* = 7.0 Hz, 3H). ^13^C NMR (101 MHz, DMSO-*d*_6_) *δ* 171.7, 162.3, 142.6, 137.8, 137.7, 136.0, 131.9, 130.4, 130.1, 129.0, 129.0, 128.5, 128.2, 128.0, 128.0, 127.6, 66.4, 59.0, 42.1, 21.0, 13.1. HRMS (ESI/Q-TOF) *m*/*z*: [M + H^+^]^+^ Calcd for C_25_H_24_NO_3_^+^ 386.1751; Found 386.1744.

Crystal Data for C_28.571429_H_26.285714_N_1.142857_O_3.428571_ (*M* = 440.51 g/mol): orthorhombic, space group Pbca (no. 61), *a* = 15.8652(2) Å, *b* = 14.6469(2) Å, *c* = 16.7176(2) Å, *V* = 3884.77(9) Å^3^, *Z* = 7, *T* = 100.15 K, μ(CuKα) = 0.689 mm^−1^, *Dcalc* = 1.318 g/cm^3^, 41,022 reflections measured (9.774° ≤ 2Θ ≤ 152.44°), 4053 unique (*R*_int_ = 0.0439, R_sigma_ = 0.0168) which were used in all calculations. The final *R*_1_ was 0.0408 (I > 2σ(I)) and *wR*_2_ was 0.1105 (all data). Please see ESI (p.S2-5) for details.

#### 4.3.2. (±)-(3R,4R)-3-(4-Nitrophenyl)-1-oxo-4-phenyl-2-propyl-1,2,3,4-tetrahydroisoquinoline-4-carboxylic Acid (**9b**)

Prepared according to the general procedure GP2 from **10b** and *N*-(4-nitrobenzylidene)propan-1-amine. Yield 62 mg, 74%. Colorless amorphous solid. ^1^H NMR (400 MHz, DMSO-*d*_6_) *δ* 13.46 (s, 1H), 8.12–8.05 (m, 3H), 8.03–7.98 (m, 1H), 7.72–7.66 (m, 1H), 7.60 (t, *J* = 7.5 Hz, 1H), 7.39 (d, *J* = 8.5 Hz, 2H), 7.29 (p, *J* = 6.6 Hz, 5H), 5.80 (s, 1H), 3.51 (ddd, *J* = 13.2, 8.9, 6.7 Hz, 1H), 2.97 (ddd, *J* = 13.6, 9.0, 5.0 Hz, 1H), 1.23 (dt, *J* = 8.1, 4.9 Hz, 1H), 1.15–0.99 (m, 1H), 0.47 (t, *J* = 7.3 Hz, 3H). ^13^C NMR (101 MHz, DMSO-*d*_6_) *δ* 171.7, 162.7, 147.6, 147.2, 142.3, 137.0, 132.4, 130.5, 130.3, 130.0, 128.7, 128.5, 128.3, 128.1, 127.8, 123.5, 66.3, 59.5, 48.3, 20.7, 11.4. HRMS (ESI/Q-TOF) *m*/*z*: [M + H^+^]^+^ Calcd for C_25_H_23_N_2_O_5_^+^ 431.1601; Found 431.1606.

#### 4.3.3. (±)-(3R,4R)-2-Ethyl-7-methyl-1-oxo-4-phenyl-3-(p-tolyl)-1,2,3,4-tetrahydroisoquinoline-4-carboxylic Acid (**9c**)

Prepared according to the general procedure GP2 from **10d** and *N*-(4-methylbenzylidene)ethanamine. Yield 45 mg, 61%. Colorless amorphous solid. ^1^H NMR (400 MHz, DMSO-*d*_6_) *δ* 13.06 (s, 1H), 8.05–7.99 (m, 1H), 7.83–7.80 (m, 1H), 7.47–7.41 (m, 1H), 7.35–7.26 (m, 4H), 7.25–7.19 (m, 1H), 7.02–6.92 (m, 4H), 5.55 (s, 1H), 3.57 (dq, *J* = 14.0, 7.1 Hz, 1H), 3.18 (dq, *J* = 14.0, 7.1 Hz, 1H), 2.42 (s, 3H), 2.21 (s, 3H), 0.74 (t, *J* = 7.1 Hz, 3H). ^13^C NMR (126 MHz, DMSO-*d*_6_) *δ* 171.8, 162.4, 142.8, 137.6, 137.5, 136.1, 134.9, 132.6, 130.2, 130.1, 129.0, 129.0, 128.5, 128.4, 128.0, 127.5, 66.5, 58.8, 42.1, 21.2, 21.0, 13.1. HRMS (ESI/Q-TOF) *m*/*z*: [M + Na^+^]^+^ Calcd for C_26_H_25_NO_3_Na^+^ 422.1727; Found 422.1718.

#### 4.3.4. (±)-(3R,4R)-2-Ethyl-4-(4-methoxyphenyl)-1-oxo-3-(p-tolyl)-1,2,3,4-tetrahydroisoquinoline-4-carboxylic Acid (**9d**)

Prepared according to the general procedure GP2 from **10c** and *N*-(4-methylbenzylidene)ethanamine. Yield 41 mg, 57%. Colorless amorphous solid. ^1^H NMR (400 MHz, DMSO-*d*_6_) *δ* 13.04 (s, 1H), 8.17–8.09 (m, 1H), 8.05–7.94 (m, 1H), 7.64–7.59 (m, 1H), 7.54–7.46 (m, 1H), 7.25–7.19 (m, 2H), 7.02–6.95 (m, 4H), 6.88–6.82 (m, 2H), 5.54 (s, 1H), 3.70 (s, 3H), 3.57 (dq, *J* = 13.9, 7.2 Hz, 1H), 3.17 (dq, *J* = 13.9, 7.2 Hz, 1H), 2.20 (s, 3H), 0.77 (t, *J* = 7.1 Hz, 3H). ^13^C NMR (101 MHz, DMSO-*d*_6_) *δ* 172.0, 162.4, 158.6, 138.1, 137.6, 136.0, 134.4, 131.8, 130.4, 130.1, 129.3, 129.0, 128.9, 128.1, 128.0, 113.8, 66.5, 58.4, 55.5, 42.1, 21.0, 13.1. HRMS (ESI/Q-TOF) *m*/*z*: [M + H^+^]^+^ Calcd for C_26_H_26_NO_4_^+^ 416.1856; Found 416.1848.

#### 4.3.5. (±)-(3R,4R)-7-Chloro-4-(4-chlorophenyl)-2-ethyl-1-oxo-3-(p-tolyl)-1,2,3,4-tetrahydroisoquinoline-4-carboxylic acid (**9e**)

Prepared according to the general procedure GP2 from **10f** and *N*-(4-methylbenzylidene)ethanamine. Yield 43 mg, 61%. Colorless amorphous solid. ^1^H NMR (400 MHz, DMSO-*d*_6_) *δ* 13.48 (s, 1H), 8.26–8.13 (m, 1H), 8.01–7.93 (m, 1H), 7.79–7.55 (m, 1H), 7.52–7.27 (m, 4H), 7.10–6.85 (m, 4H), 5.65 (s, 1H), 3.58 (dq, *J* = 14.1, 7.1 Hz, 1H), 3.19 (dq, *J* = 14.1, 7.1 Hz, 1H), 2.21 (s, 3H), 0.78 (t, *J* = 7.1 Hz, 3H). ^13^C NMR (101 MHz, DMSO-*d*_6_) *δ* 171.0, 161.2, 141.0, 137.9, 136.4, 135.2, 133.4, 132.6, 132.6, 132.3, 131.9, 130.1, 129.2, 128.9, 128.5, 127.5, 66.3, 58.6, 42.3, 21.0, 13.0. HRMS (ESI/Q-TOF) *m*/*z*: [M + H^+^]^+^ Calcd for C_25_H_22_C_l2_NO_3_^+^ 454.0971; Found 454.0965.

#### 4.3.6. (±)-(3R,4R)-3-(4-(Benzyloxy)-3-methoxyphenyl)-4-(4-chlorophenyl)-1-oxo-2-(prop-2-yn-1-yl)-1,2,3,4-tetrahydroisoquinoline-4-carboxylic Acid (**9f**)

Prepared according to the general procedure GP2 from 10b and N-(4-(benzyloxy)-3-methoxybenzylidene)prop-2-yn-1-amine. Yield 76 mg, 80%. Colorless amorphous solid. ^1^H NMR (400 MHz, DMSO-d_6_) δ 13.37 (s, 1H), 8.29–8.18 (m, 1H), 8.08–7.96 (m, 1H), 7.74–7.65 (m, 1H), 7.58–7.26 (m, 10H), 6.92–6.82 (m, 1H), 6.67–6.61 (m, 1H), 6.59–6.50 (m, 1H), 5.75 (s, 1H), 4.98 (s, 2H), 4.65 (d, J = 17.4 Hz, 1H), 3.77 (d, J = 17.4 Hz, 1H), 3.51 (s, 3H). ^13^C NMR (126 MHz, DMSO-d_6_) δ 171.2, 162.3, 148.5, 148.3, 141.2, 137.9, 137.4, 136.7, 133.1, 132.7, 132.5, 130.2, 129.8, 129.3, 129.2, 128.9, 128.8, 128.6, 128.5, 128.4, 128.3, 128.3, 121.2, 113.1, 79.0, 76.1, 70.2, 58.4, 55.5. HRMS (ESI/Q-TOF) *m*/*z*: [M + H^+^]^+^ Calcd for C_33_H_27_ClNO_5_^+^ 552.1572; Found 552.1574.

#### 4.3.7. (±)-(3R,4R)-2-Benzyl-4-(4-chlorophenyl)-3-(2-methoxyphenyl)-1-oxo-1,2,3,4-tetrahydroisoquinoline-4-carboxylic Acid (**9g**)

Prepared according to the general procedure GP2 from **10a** and *N*-(2-methoxybenzylidene)-1-phenylmethanamine. Yield 62 mg, 61%. Colorless amorphous solid. ^1^H NMR (400 MHz, DMSO-*d*_6_) *δ* 12.95 (s, 1H), 8.20–8.11 (m, 1H), 7.71–7.65 (m, 1H), 7.64–7.58 (m, 1H), 7.55 (d, *J* = 7.7 Hz, 1H), 7.27–7.19 (m, 2H), 7.13–7.07 (m, 2H), 7.05–7.00 (m, 3H), 6.83–6.60 (m, 6H), 5.79 (s, 1H), 5.19 (d, *J* = 14.6 Hz, 1H), 3.87 (s, 3H), 3.40 (d, *J* = 14.6 Hz, 1H). ^13^C NMR (101 MHz, DMSO-*d*_6_) *δ* 171.5, 162.9, 158.3, 142.0, 137.4, 136.7, 132.8, 132.2, 129.9, 129.7, 128.8, 128.5, 128.5, 128.3, 127.9, 127.4, 127.3, 126.8, 120.8, 111.5, 59.5, 59.2, 56.2, 48.4. HRMS (ESI/Q-TOF) *m*/*z*: [M + H^+^]^+^ Calcd for C_30_H_25_ClNO_4_^+^ 498.1467; Found 498.1471.

#### 4.3.8. (±)-(3R,4R)-2-Allyl-4-(4-chlorophenyl)-3-(2,4-dimethoxyphenyl)-1-oxo-1,2,3,4-tetrahydroisoquinoline-4-carboxylic Acid (**9h**)

Prepared according to the general procedure GP2 from **10a** and *N*-(2,4-dimethoxybenzylidene)prop-2-en-1-amine. Yield 53 mg, 64%. Colorless amorphous solid. ^1^H NMR (400 MHz, DMSO-*d*_6_) *δ* 12.95 (s, 1H), 8.11–8.04 (m, 1H), 7.71–7.52 (m, 3H), 7.41–7.31 (m, 2H), 7.18–7.07 (m, 2H), 6.59–6.47 (m, 2H), 6.33–6.26 (m, 1H), 5.81 (s, 1H), 5.28–5.16 (m, 1H), 4.97 (d, *J* = 10.1 Hz, 1H), 4.88 (d, *J* = 17.1 Hz, 1H), 4.38 (dd, *J* = 15.8, 4.8 Hz, 1H), 3.82 (s, 3H), 3.69 (s, 3H), 3.21 (dd, *J* = 15.2, 7.6 Hz, 1H). ^13^C NMR (126 MHz, DMSO-*d*_6_) *δ* 171.6, 162.6, 160.7, 159.2, 142.1, 137.7, 133.0, 132.6, 132.3, 130.2, 130.0, 129.7, 128.6, 128.2, 128.1, 118.9, 118.7, 105.2, 98.7, 59.2, 59.0, 56.2, 55.6, 48.2. HRMS (ESI/Q-TOF) *m*/*z*: [M + H^+^]^+^ Calcd for C_27_H_25_ClNO_5_^+^ 478.1416; Found 478.1421.

#### 4.3.9. (±)-(3R,4R)-4-(4-Chlorophenyl)-1-oxo-3-phenyl-2-(p-tolyl)-1,2,3,4-tetrahydroisoquinoline-4-carboxylic Acid (**9i**)

Prepared according to the general procedure GP2 from **10a** and *N*-benzylidene-4-methylaniline. Yield 62 mg, 77%. Colorless amorphous solid. ^1^H NMR (400 MHz, DMSO-*d*_6_) *δ* 13.48 (s, 1H), 8.17–8.03 (m, 2H), 7.77–7.69 (m, 1H), 7.66–7.59 (m, 1H), 7.50–7.41 (m, 2H), 7.37–7.30 (m, 2H), 7.27–7.17 (m, 3H), 7.11–7.03 (m, 4H), 6.67–6.49 (m, 2H), 5.75 (s, 1H), 2.25 (s, 3H). ^13^C NMR (126 MHz, DMSO-*d*_6_) *δ* 171.1, 162.7, 141.3, 139.5, 138.2, 137.1, 136.8, 132.8, 132.8, 130.6, 130.3, 129.9, 129.1, 128.9, 128.7, 128.7, 126.5, 70.4, 59.7, 21.0. HRMS (ESI/Q-TOF) *m*/*z*: [M + H^+^]^+^ Calcd for C_29_H_23_ClNO_3_^+^ 468.1361; Found 468.1368.

#### 4.3.10. (±)-(3R,4R)-4-(4-Chlorophenyl)-3-(4-methoxyphenyl)-1-oxo-2-(4-(trifluoromethyl)phenyl)-1,2,3,4-tetrahydroisoquinoline-4-carboxylic Acid (**9j**)

Prepared according to the general procedure GP2 from **10a** and *N*-(4-methoxybenzylidene)-4-(trifluoromethyl)aniline. Yield 71 mg, 75%. Colorless amorphous solid. ^1^H NMR (400 MHz, DMSO-*d*_6_) *δ* 13.50 (s, 1H), 8.17–8.09 (m, 2H), 7.79–7.73 (m, 1H), 7.73–7.68 (m, 2H), 7.67–7.61 (m, 1H), 7.46–7.40 (m, 2H), 7.37–7.30 (m, 2H), 7.07–7.01 (m, 4H), 6.81–6.77 (m, 2H), 5.92 (s, 1H), 3.69 (s, 3H). ^13^C NMR (101 MHz, DMSO-*d*_6_) *δ* 170.9, 162.9, 159.5, 145.6, 141.1, 137.4, 133.1, 132.8, 130.4, 130.3, 130.1, 129.6, 128.9, 128.8, 128.7, 127.5 (q, *J* = 32.3 Hz), 127.2, 126.5 (q, *J* = 3.6 Hz), 124.4 (q, *J* = 271.9 Hz), 114.2, 69.0, 59.8, 55.5. ^19^F NMR (376 MHz, DMSO-*d*_6_) *δ* −60.9. HRMS (ESI/Q-TOF) *m*/*z*: [M + H^+^]^+^ Calcd for C_30_H_22_ClF_3_NO_4_^+^ 552.1184; Found 552.1184.

#### 4.3.11. (±)-(3S,4R)-4-(4-Chlorophenyl)-1-oxo-3-(thiophen-2-yl)-2-(p-tolyl)-1,2,3,4-tetrahydroisoquinoline-4-carboxylic Acid (**9k**)

Prepared according to the general procedure GP2 from **10a** and 4-methyl-*N*-(thiophen-2-ylmethylene)aniline. Yield 55 mg, 67%. Colorless amorphous solid. ^1^H NMR (400 MHz, DMSO-*d*_6_) *δ* 13.55 (s, 1H), 8.26–8.19 (m, 1H), 8.12–8.05 (m, 1H), 7.78–7.70 (m, 1H), 7.67–7.59 (m, 1H), 7.48–7.41 (m, 2H), 7.37–7.33 (m, 1H), 7.30–7.26 (m, 2H), 7.13–7.05 (m, 3H), 6.63–6.51 (m, 3H), 5.88 (s, 1H), 2.27 (s, 3H). ^13^C NMR (101 MHz, DMSO-*d*_6_) *δ* 171.2, 162.7, 140.8, 139.6, 139.4, 137.3, 136.7, 132.9, 132.8, 130.7, 130.1, 129.9, 129.8, 128.9, 128.8, 128.8, 128.0, 126.6, 126.3, 125.5, 66.2, 59.2, 21.0. HRMS (ESI/Q-TOF) *m*/*z*: [M + H^+^]^+^ Calcd for C_27_H_21_ClNO_3_S^+^ 474.0925; Found 474.0926.

#### 4.3.12. (±)-(3R,4R)-4-(4-Chlorophenyl)-3-(4-fluorophenyl)-2-(4-methoxybenzyl)-1-oxo-1,2,3,4-tetrahydroisoquinoline-4-carboxylic Acid (**9l**)

Prepared according to the general procedure GP2 from **10a** and *N*-(4-fluorobenzylidene)-1-(4-methoxyphenyl)methanamine. Yield 64 mg, 72%. Colorless amorphous solid. ^1^H NMR (400 MHz, DMSO-*d*_6_) *δ* 13.38 (s, 1H), 8.16–8.09 (m, 1H), 8.02–7.95 (m, 1H), 7.72–7.64 (m, 1H), 7.62–7.57 (m, 1H), 7.16–6.99 (m, 6H), 6.96–6.87 (m, 4H), 6.77–6.65 (m, 2H), 5.38 (s, 1H), 5.04 (d, *J* = 14.3 Hz, 1H), 3.76 (s, 3H), 3.69 (d, *J* = 14.3 Hz, 1H). ^13^C NMR (101 MHz, DMSO-*d*_6_) *δ* 171.3, 162.5, 162.3 (d, *J* = 244.5 Hz), 159.1, 140.9, 136.7, 134.1 (d, *J* = 3.1 Hz), 132.6, 132.3, 131.2 (d, *J* = 8.2 Hz), 130.6, 130.3, 130.0, 129.6, 128.8, 128.6, 128.5, 128.2, 115.4 (d, *J* = 21.3 Hz), 114.0, 65.5, 58.8, 55.5, 48.2. ^19^F NMR (376 MHz, DMSO-*d*_6_) *δ* −114.0. HRMS (ESI/Q-TOF) *m*/*z*: [M + H^+^]^+^ Calcd for C_30_H_24_ClFNO_4_^+^ 516.1372; Found 516.1373.

#### 4.3.13. (±)-(3R,4R)-4-(4-Chlorophenyl)-1-oxo-2,3-di-p-tolyl-1,2,3,4-tetrahydroisoquinoline-4-carboxylic Acid (**9m**)

Prepared according to the general procedure GP2 from **10a** and 4-methyl-*N*-(4-methylbenzylidene)aniline. Yield 55 mg, 66%. Colorless amorphous solid. ^1^H NMR (400 MHz, DMSO-*d*_6_) *δ* 13.44 (s, 1H), 8.12–8.06 (m, 2H), 7.76–7.68 (m, 1H), 7.66–7.57 (m, 1H), 7.49–7.38 (m, 2H), 7.35–7.29 (m, 2H), 7.12–7.07 (m, 2H), 7.04–6.89 (m, 2H), 6.65–6.58 (m, 2H), 5.72 (s, 1H), 2.25 (s, 3H), 2.22 (s, 3H). ^13^C NMR (101 MHz, DMSO-*d*_6_) *δ* 171.1, 162.7, 141.3, 139.6, 137.9, 137.2, 136.7, 135.2, 132.7, 132.7, 130.6, 130.3, 129.8, 129.3, 129.0, 128.8, 128.7, 126.4, 70.1, 59.7, 21.0, 21.0. HRMS (ESI/Q-TOF) *m*/*z*: [M + H^+^]^+^ Calcd for C_30_H_25_ClNO_3_^+^ 482.1517; Found 482.1518.

#### 4.3.14. (±)-(3S,4R)-4-(4-Chlorophenyl)-2-(2-(cyclopentylthio)ethyl)-3-(furan-2-yl)-1-oxo-1,2,3,4-tetrahydroisoquinoline-4-carboxylic Acid (**9n**)

Prepared according to the general procedure GP2 from **10a** and 2-(cyclopentylthio)-*N*-(furan-2-ylmethylene)ethanamine. Yield 56 mg, 66%. Colorless amorphous solid. ^1^H NMR (400 MHz, DMSO-*d*_6_) *δ* 13.52 (s, 1H), 8.20–8.14 (m, 1H), 8.00–7.94 (m, 1H), 7.73–7.64 (m, 1H), 7.59–7.50 (m, 1H), 7.48–7.43 (m, 1H), 7.42–7.35 (m, 2H), 7.32–7.23 (m, 2H), 6.27 (dd, *J* = 3.3, 1.9 Hz, 1H), 5.87 (d, *J* = 3.3 Hz, 1H), 5.84 (s, 1H), 3.68 (td, *J* = 13.2, 11.2, 5.0 Hz, 1H), 3.37 (td, 1H), 3.10 (p, *J* = 7.1 Hz, 1H), 2.33 (td, *J* = 12.8, 11.1, 5.0 Hz, 1H), 2.03 (ddd, *J* = 12.8, 11.1, 5.0 Hz, 1H), 1.92 (dt, *J* = 13.9, 6.9 Hz, 2H), 1.70–1.57 (m, 2H), 1.60–1.48 (m, 2H), 1.32 (dt, *J* = 13.9, 6.9 Hz, 2H). ^13^C NMR (101 MHz, DMSO-*d*_6_) *δ* 171.4, 163.1, 152.4, 143.0, 140.3, 132.8, 132.3, 130.0, 130.0, 129.1, 128.6, 128.5, 128.5, 110.8, 109.0, 61.2, 57.8, 47.7, 43.4, 33.9, 28.2, 24.8, 24.7. HRMS (ESI/Q-TOF) *m*/*z*: [M + H^+^]^+^ Calcd for C_27_H_27_ClNO_4_S^+^ 496.1344; Found 496.1344.

#### 4.3.15. (±)-(3R,4R)-4-(4-Chlorophenyl)-2-ethyl-1-oxo-3-(p-tolyl)-1,2,3,4-tetrahydroisoquinoline-4-carboxylic acid (**9o**)

Prepared according to the general procedure GP2 from **10a** and *N*-(4-methylbenzylidene)ethanamine. Yield 34 mg, 47%. Colorless amorphous solid. ^1^H NMR (400 MHz, DMSO-*d*_6_) *δ* 13.06 (s, 1H), 8.09–7.97 (m, 1H), 7.86–7.79 (m, 1H), 7.49–7.42 (m, 1H), 7.34–7.26 (m, 4H), 7.25–7.19 (m, 1H), 7.04–6.92 (m, 4H), 5.55 (s, 1H), 3.57 (dq, *J* = 14.0, 7.1 Hz, 1H), 3.18 (dq, *J* = 14.0, 7.1 Hz, 1H), 2.42 (s, 3H), 2.21 (s, 3H), 0.74 (t, *J* = 7.1 Hz, 3H). ^13^C NMR (101 MHz, DMSO-*d*_6_) *δ* 171.3, 162.3, 141.5, 137.8, 137.3, 135.7, 132.4, 132.1, 130.4, 130.2, 130.1, 129.1, 128.9, 128.5, 128.4, 128.1, 66.4, 58.9, 42.0, 21.0, 13.1. HRMS (ESI/Q-TOF) *m*/*z*: [M + Na^+^]^+^ Calcd for C_25_H_22_ClNO_3_Na^+^ 442.1180; Found 442.1175.

#### 4.3.16. (±)-(3S,4R)-4-(4-Chlorophenyl)-1-oxo-2-propyl-3-(pyridin-2-yl)-1,2,3,4-tetrahydroisoquinoline-4-carboxylic Acid (**9p**)

Prepared according to the general procedure GP2 from **10a** and *N*-(pyridin-3-ylmethylene)propan-1-amine. Yield 34 mg, 47%. Colorless amorphous solid. ^1^H NMR (400 MHz, DMSO-*d*_6_) *δ* 13.50 (s, 1H), 8.50–8.37 (m, 2H), 8.13–8.02 (m, 1H), 8.02–7.93 (m, 1H), 7.72–7.65 (m, 1H), 7.64–7.58 (m, 1H), 7.43–7.37 (m, 2H), 7.35–7.26 (m, 3H), 7.24–7.18 (m, 1H), 5.68 (s, 1H), 3.55–3.45 (m, 1H), 3.04–2.93 (m, 1H), 1.32–1.21 (m, 1H), 1.15–1.03 (m, 1H), 0.49 (t, *J* = 7.3 Hz, 3H). ^13^C NMR (101 MHz, DMSO-*d*_6_) *δ* 171.4, 162.6, 150.5, 149.6, 141.1, 136.5, 135.9, 134.7, 132.7, 132.5, 130.4, 130.1, 130.1, 128.9, 128.5, 128.4, 123.6, 64.7, 59.1, 48.2, 20.8, 11.3. HRMS (ESI/Q-TOF) *m*/*z*: [M + H^+^]^+^ Calcd for C_24_H_22_ClN_2_O_3_^+^ 421.1313; Found 421.1315.

#### 4.3.17. (±)-(3S,4R)-3-(2-Chlorophenyl)-4-(4-fluorophenyl)-1-oxo-2-propyl-1,2,3,4-tetrahydroisoquinoline-4-carboxylic Acid (**9q**)

Prepared according to the general procedure GP2 from **10e** and *N*-(2-chlorobenzylidene)propan-2-amine. Yield 45 mg, 56% (*dr* = 3/1). Colorless amorphous solid. Major isomer: ^1^H NMR (400 MHz, DMSO-*d*_6_) *δ* 13.34 (s, 1H), 8.17–8.10 (m, 1H), 7.75–7.69 (m, 1H), 7.68–7.60 (m, 2H), 7.51–7.45 (m, 1H), 7.30–7.24 (m, 1H), 7.22–7.07 (m, 5H), 6.80–6.73 (m, 1H), 5.90 (s, 1H), 3.71–3.58 (m, 1H), 2.62–2.54 (m, 1H), 1.28–1.13 (m, 1H), 1.09–0.96 (m, 1H), 0.38 (t, *J* = 7.5 Hz, 3H). ^13^C NMR (126 MHz, DMSO-*d*_6_) *δ* 171.3, 161.9, 161.2 (d, *J* = 244.8 Hz), 158.7, 138.0 (d, *J* = 2.9 Hz), 136.2, 131.6, 131.1, 131.1, 130.4 (d, *J* = 8.2 Hz), 130.1, 130.0, 128.1, 127.7, 114.6 (d, *J* = 21.5 Hz), 113.3, 64.1, 59.2, 55.0, 19.9, 19.6. ^19^F NMR (376 MHz, DMSO-*d*_6_) *δ* -115.5. HRMS (ESI/Q-TOF) *m*/*z*: [M + H^+^]^+^ Calcd for C_25_H_22_ClFNO_3_^+^ 438.1267; Found 438.1271. Minor isomer, partial data: ^1^H NMR (400 MHz, DMSO) δ 7.44 (d, *J* = 7.9 Hz, 1H), 7.40–7.33 (m, 2H), 7.04 (t, *J* = 7.3 Hz, 2H), 6.63 (dd, *J* = 7.9, 1.6 Hz, 1H), 6.32 (t, *J* = 7.8 Hz, 1H), 5.98 (d, *J* = 1.9 Hz, 1H). ^19^F NMR (376 MHz, DMSO) δ −108.77.

#### 4.3.18. (±)-(3R,4R)-4-(4-Fluorophenyl)-2-isopropyl-3-(4-methoxyphenyl)-1-oxo-1,2,3,4-tetrahydroisoquinoline-4-carboxylic Acid (**9r**)

Prepared according to the general procedure GP2 from **10e** and *N*-(4-methoxybenzylidene)propan-2-amine. Yield 41 mg, 52% (*dr* = 4/1). Colorless amorphous solid. Major isomer: ^1^H NMR (400 MHz, DMSO-*d*_6_) *δ* 13.21 (s, 1H), 8.12–8.04 (m, 1H), 7.98–7.88 (m, 1H), 7.66–7.59 (m, 1H), 7.61–7.51 (m, 1H), 7.38–7.30 (m, 2H), 7.18–7.07 (m, 2H), 7.06–7.00 (m, 2H), 6.77–6.66 (m, 2H), 5.51 (s, 1H), 4.27 (hept, *J* = 6.7 Hz, 1H), 3.67 (s, 3H), 0.83 (dd, *J* = 20.2, 6.7 Hz, 6H). ^13^C NMR (126 MHz, DMSO-*d*_6_) *δ* 171.7, 162.3, 161.6 (d, *J* = 244.8 Hz), 159.1, 138.4 (d, *J* = 2.9 Hz), 136.6, 132.0, 131.5, 131.5, 130.8 (d, *J* = 8.2 Hz), 130.5, 130.4, 128.5, 128.1, 115.0 (d, *J* = 21.5 Hz), 113.7, 64.5, 59.6, 55.4, 48.8, 20.3, 20.0. ^19^F NMR (376 MHz, DMSO-*d*_6_) *δ* −115.1. HRMS (ESI/Q-TOF) *m*/*z*: [M + H^+^]^+^ Calcd for C_26_H_25_FNO_4_^+^ 434.1762; Found 434.1768. Minor isomer, partial data: ^1^H NMR (400 MHz, DMSO-*d*_6_) δ 7.23 (dd, *J* = 12.4, 8.1 Hz, 1H), 6.95–6.81 (m, 2H), 5.35 (s, 1H). ^19^F NMR (376 MHz, DMSO-*d*_6_) δ −108.81.

#### 4.3.19. (±)-(3R,4R)-2-Butyl-4-(4-fluorophenyl)-3-(4-methoxyphenyl)-1-oxo-1,2,3,4-tetrahydroisoquinoline-4-carboxylic Acid (**9s**)

Prepared according to the general procedure GP2 from **10e** and *N*-(4-methoxybenzylidene)butan-1-amine. Yield 61 mg, 75% (*dr* = 4/1). Colorless amorphous solid. Major isomer: ^1^H NMR (400 MHz, DMSO-*d*_6_) *δ* 13.20 (s, 1H), 8.13–7.98 (m, 2H), 7.69–7.62 (m, 1H), 7.59–7.53 (m, 1H), 7.39–7.28 (m, 2H), 7.17–7.08 (m, 2H), 7.07–6.97 (m, 2H), 6.78–6.71 (m, 2H), 5.49 (s, 1H), 3.68 (s, 3H), 3.65–3.52 (m, 1H), 3.04–2.89 (m, 1H), 1.32–1.18 (m, 1H), 1.16–1.05 (m, 1H), 0.96–0.85 (m, 1H), 0.85–0.73 (m, 1H), 0.67 (t, *J* = 7.2 Hz, 3H). ^13^C NMR (126 MHz, DMSO-*d*_6_) *δ* 171.6, 162.6, 160.6, 159.3, 138.6 (d, *J* = 3.1 Hz), 137.3, 132.1, 130.4 (d, *J* = 5.9 Hz), 130.4, 130.3, 128.5, 128.1, 115.1 (d, *J* = 21.2 Hz), 113.8, 66.7, 58.9, 55.4, 46.0, 29.5, 19.8, 14.1. ^19^F NMR (376 MHz, DMSO-*d*_6_) *δ* −115.7. HRMS (ESI/Q-TOF) *m*/*z*: [M + H^+^]^+^ Calcd for C_27_H_27_FNO_4_^+^ 448.1919; Found 448.1924. Minor isomer, partial data: ^1^H NMR (400 MHz, DMSO-*d*_6_) δ 7.72 (dd, *J* = 8.1, 2.9 Hz, 2H), 7.25–7.18 (m, 1H), 7.06 (d, *J* = 8.2 Hz, 1H), 6.88 (d, *J* = 8.4 Hz, 2H), 5.31 (s, 1H), 3.24–3.12 (m, 1H). ^19^F NMR (376 MHz, DMSO-*d*_6_) δ −108.66.

#### 4.3.20. (±)-(3R,4R)-4-(4-Fluorophenyl)-3-(2-methoxyphenyl)-1-oxo-2-(p-tolyl)-1,2,3,4-tetrahydroisoquinoline-4-carboxylic Acid (**9t**)

Prepared according to the general procedure GP2 from **10e** and 2-methoxy-*N*-(4-methylbenzylidene)aniline. Yield 52 mg, 71% (dr = 3/1). Colorless amorphous solid. ^1^H NMR (400 MHz, DMSO-*d*_6_) *δ* 12.98 (s, 1H), 8.22–8.11 (m, 1H), 7.77–7.67 (m, 1H), 7.66–7.58 (m, 1H), 7.48–7.41 (m, 1H), 7.25–7.18 (m, 3H), 7.16–7.09 (m, 2H), 7.05–6.98 (m, 2H), 6.96–6.88 (m, 2H), 6.84–6.65 (m, 1H), 6.49–6.37 (m, 2H), 6.09 (s, 1H), 3.68 (s, 3H), 2.21 (s, 3H). ^13^C NMR (101 MHz, DMSO-*d*_6_) *δ* 171.6, 162.9, 161.6 (d, *J* = 244.7 Hz), 158.0, 140.0, 139.6 (d, *J* = 2.6 Hz), 137.8, 136.8, 133.0, 130.7 (d, *J* = 8.3 Hz), 130.3, 130.0, 129.8, 129.6, 128.8, 128.4, 127.7, 127.0, 126.3, 120.7, 115.1 (d, *J* = 21.2 Hz), 111.3, 65.2, 60.5, 55.9, 21.0. ^19^F NMR (376 MHz, DMSO-*d*_6_) *δ* −115.5. HRMS (ESI/Q-TOF) *m*/*z*: [M + H^+^]^+^ Calcd for C_30_H_25_FNO_4_^+^ 482.1762; Found 482.1763. Minor isomer, partial data: ^1^H NMR (400 MHz, DMSO-*d*_6_) δ 7.37–7.26 (m, 2H), 6.36 (d, *J* = 8.0 Hz, 2H), 6.27 (d, *J* = 2.8 Hz, 1H), 3.64 (s, 3H). ^19^F NMR (376 MHz, DMSO-*d*_6_) δ −108.30.

#### 4.3.21. (±)-(3R,4R)-2-Ethyl-4-(4-fluorophenyl)-1-oxo-3-(p-tolyl)-1,2,3,4-tetrahydroisoquinoline-4-carboxylic acid (**9u**)

Prepared according to the general procedure GP2 from **10e** and *N*-(4-methylbenzylidene)ethanamine. Yield 50 mg, 68%. Colorless amorphous solid. ^1^H NMR (400 MHz, DMSO-*d*_6_) *δ* 13.25 (s, 1H), 8.17–8.06 (m, 1H), 8.07–8.00 (m, 1H), 7.69–7.59 (m, 1H), 7.58–7.48 (m, 1H), 7.40–7.30 (m, 2H), 7.17–7.06 (m, 2H), 6.98 (s, 4H), 5.58 (s, 1H), 3.59 (dq, *J* = 13.9, 7.0 Hz, 1H), 3.15 (dq, *J* = 14.0, 6.8 Hz, 1H), 2.21 (s, 3H), 0.76 (t, *J* = 7.1 Hz, 3H). ^13^C NMR (101 MHz, DMSO-*d*_6_) *δ* 171.6, 162.3, 161.5 (d, *J* = 244.5 Hz), 138.8 (d, *J* = 3.4 Hz), 137.7, 135.8, 132.0, 130.4, 130.3, 130.2, 130.2, 129.0, 128.9, 128.3, 128.0, 115.2 (d, *J* = 21.2 Hz), 66.7, 58.7, 42.0, 21.0, 13.1. ^19^F NMR (376 MHz, DMSO-*d*_6_) *δ* −115.7. HRMS (ESI/Q-TOF) *m*/*z*: [M + Na^+^]^+^ Calcd for C_25_H_22_FNO_3_Na^+^ 426.1476; Found 426.1469.

### 4.4. 2-(Carboxy(4-phenyl-1H-1,2,3-triazol-1-yl)methyl)benzoic Acid (***15***)

Ethyl 2-(1-azido-2-methoxy-2-oxoethyl)benzoate[20] **16** (526 mg, 2 mmol, 1 equiv.) and phenylacetylene (206 mg, 1 equiv.) were added to a suspension of CuI (27 mg, 7 mol. %) in dry toluene. The reaction mixture was stirred at 85 °C overnight. The solvent was evaporated, and the title compound was extracted with ethyl acetate (30 mL). The organic layer was washed with water (20 mL × 2) and brine (20 mL × 1) and then dried over Na_2_SO_4_. The solvent was evaporated and the resulting compound (ethyl 2-(2-ethoxy-2-oxo-1-(4-phenyl-1H-1,2,3-triazol-1-yl)ethyl)benzoate) was used in the next step without further purification. The obtained ester and KOH (560 mg, 5 equiv.) were dissolved in 30 mL of 30% aq.THF and stirred for 1 h at room temperature. Activated charcoal (**12g**) (powder -100 particle size (mesh)) was added to the resulting mixture and intensively stirred at room temperature for 0.5 h. Next, the solution was filtered through a layer of zeolite, and 3 N HCl was added to it until pH = 1. The target compound (**15**) was extracted into diethyl ether, and the organic layer was combined and dried over Na_2_SO_4_ and was evaporated.

Yield 601 mg, 93% (2 steps). Colorless amorphous solid. ^1^H NMR (400 MHz, DMSO-*d*_6_) *δ* 13.55 (s, 2H), 8.72 (s, 1H), 8.07–8.00 (m, 1H), 7.94–7.82 (m, 2H), 7.71–7.61 (m, 1H), 7.60–7.51 (m, 2H), 7.50–7.42 (m, 2H), 7.39–7.30 (m, 1H), 7.29–7.20 (m, 1H). ^13^C NMR (101 MHz, DMSO-*d*_6_) *δ* 169.0, 168.5, 146.6, 135.7, 133.1, 131.4, 130.9, 130.6, 129.5, 129.4, 129.2, 128.5, 125.7, 122.9, 63.7. HRMS (ESI/Q-TOF) *m*/*z*: [M + H^+^]^+^ Calcd for C_17_H_14_N_3_O_4_^+^ 324.0979; Found 324.0974.

### 4.5. (±)-(3R,4S)-2-Ethyl-1-oxo-4-(4-phenyl-1H-1,2,3-triazol-1-yl)-3-(p-tolyl)-1,2,3,4-tetrahydroisoquinoline-4-carboxylic Acid (***18***)

The diacid **15** (50 mg, 0.15 mmol) was dissolved in DMF (0.5 mL, dry) in a screw-cap, and DCC (1.1 equiv.) was added with stirring. After 3 h, *N*-(4-methylbenzylidene)ethanamine (1.1 equiv.) was added, and the reaction mixture was kept for a day at room temperature. The solution was then filtered through celite, EtOAc (15 mL) and 10 mL of brine were added to the filtrate, the precipitate formed was filtered off, and then organic layer of the filtrate was washed with brine (10 mL × 3), dried over sodium sulfate and evaporated. The residue was treated with diethyl ether (1 mL), after which pentane (3 mL) was added and the solid was thoroughly ground. After cooling to −20 °C for 20 min, the liquid was decanted. The resulting solid was dried in vacuo to give pure title compound. The substance undergoes decarboxylation easily and is therefore unstable in solutions even at room temperature.

Yield 35 mg, 50%. Colorless amorphous solid. ^1^H NMR (400 MHz, DMSO-*d*_6_) *δ* 14.25 (s, 1H), 8.83–8.72 (m, 1H), 8.17–8.07 (m, 1H), 7.88–7.79 (m, 3H), 7.75–7.62 (m, 2H), 7.50–7.40 (m, 2H), 7.39–7.31 (m, 1H), 7.16–6.90 (m, 4H), 6.05 (s, 1H), 3.69 (dq, *J* = 14.0, 7.0 Hz, 1H), 3.18 (dq, *J* = 14.0, 7.0 Hz, 1H), 2.24 (s, 3H), 0.90 (t, *J* = 7.0 Hz, 3H). ^13^C NMR (101 MHz, DMSO-*d*_6_) *δ* 167.7, 161.6, 146.2, 138.4, 133.3, 132.4, 131.5, 130.9, 130.4, 130.0, 129.4, 129.4, 128.8, 128.5, 127.9, 125.6, 122.1, 72.2, 65.6, 42.1, 21.1, 13.3. HRMS (ESI/Q-TOF) *m*/*z*: [M + Na^+^]^+^ Calcd for C_27_H_24_N_4_O_3_Na^+^ 475.1741; Found 475.1730.

## Data Availability

Data available from the corresponding authors upon reasonable request.

## Supplementary Materials

The following supporting information can be downloaded at: https://www.mdpi.com/article/10.3390/molecules27238462/s1. Copies of NMR and HRMS spectra. *X*-ray data [23,24,25].

## Sample Availability

Samples of the compounds **9** are available from the authors upon reasonable request.
