# Supplementary material for: The Use of Aryl-Substituted Homophthalic Anhydrides in the Castagnoli–Cushman Reaction Provides Access to Novel Tetrahydroisoquinolone Carboxylic Acid Bearing an All-Carbon Quaternary Stereogenic Center
Source: Molecules. 2022 Dec 2;27(23):8462. doi: 10.3390/molecules27238462 (PMC9738897; doi:10.3390/molecules27238462)
Supplement: Supplementary file 1 [file molecules-27-08462-s001.zip › molecules-2016613-supplementary.pdf]

Supporting Information (SI)

The use of aryl-substituted homophthalic anhydrides in the Castagnoli-Cushman reaction provides access to novel tetrahydroisoquinolone carboxylic acid bearing an all-carbon quaternary stereogenic center

Nazar Moshnenko[a], Alexander Kazantsev[a], Olga Bakulina[a], Dmitry Dar'in[a] and Mikhail Krasavin*[,a,b]

* Corresponding author.

[a]Saint Petersburg State University, 26 Universitetskii prospect, Peterhof 198504 Russian Federation

[b]Immanuel Kant Baltic Federal University, Kaliningrad 236041 Russian Federation

E-mail: m.krasavin@spbu.ru, URL: http://krasavin-group.org

Contents

1. X-Ray crystallographic data S2
2. References S3
3. Copies of ^{1}H, ^{13}C and ^{19}F NMR and HRMS spectra S6

1. X-ray crystallographic data

X-ray Single Crystal analysis was performed on Rigaku XtaLAB Synergy-S diffractometer with monochromated CuKα radiation. Crystal growth was performed by slow evaporation of solution in methanol/acetone mixture (1:1) at 5 °C. The crystal was kept at 100 K during data collection. Using Olex2[1], the structures were solved with the SHELXT[2] structure solution program using Intrinsic Phasing and refined with the SHELXL[3] refinement package using Least Squares minimization. CCDC 2211662 **(9a)** contain the supplementary crystallographic data for this paper. These data can be obtained free of charge from The Cambridge Crystallographic Data Centre via https://www.ccdc.cam.ac.uk/.

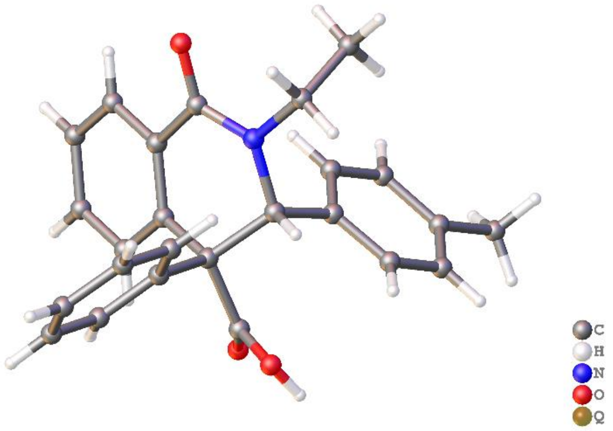

Figure S1. Crystal structure of compound 9a (ORTEP plot at 50% probability level)

Table S1 Crystal data and structure refinement for compound 9a.	
Identification code	9a
Empirical formula	$C_{28.57}H_{26.29}N_{1.14}O_{3.43}$
Formula weight	440.51
Temperature/K	100.15
Crystal system	orthorhombic
Space group	Pbca
a/Å	15.8652(2)
b/Å	14.6469(2)
c/Å	16.7176(2)
α/°	90
β/°	90
γ/°	90
Volume/Å^3	3884.77(9)
Z	7
ρ_{calc}g/cm^3	1.318
μ/mm^{-1}	0.689
F(000)	1632.0
Crystal size/mm^3	0.28 × 0.24 × 0.16
Radiation	CuKα (λ = 1.54184)
2Θ range for data collection/°	9.774 to 152.44
Index ranges	$-19 \le h \le 19, -18 \le k \le 18, -21 \le l \le 20$

Reflections collected	41022
Independent reflections	4053 [R_{int} = 0.0439, R_{sigma} = 0.0168]
Data/restraints/parameters	4053/0/265
Goodness-of-fit on F^2	1.029
Final R indexes [I>=2σ (I)]	R_1 = 0.0408, wR_2 = 0.1069
Final R indexes [all data]	R_1 = 0.0437, wR_2 = 0.1105
Largest diff. peak/hole / e $Å^{-3}$	0.39/-0.22

2. References

1. Dolomanov, O. V.; Bourhis, L. J.; Gildea, R. J.; Howard, J. A. K.; Puschmann, H., OLEX2: a complete structure solution, refinement and analysis program. *Journal of Applied Crystallography* **2009,** 42, (2), 339-341, 10.1107/s0021889808042726.

2. Sheldrick, G. M., SHELXT - integrated space-group and crystal-structure determination. *Acta Crystallogr A Found Adv* **2015,** 71, (Pt 1), 3-8, 10.1107/S2053273314026370.

3. Sheldrick, G. M., Crystal structure refinement with SHELXL. *Acta Crystallogr C Struct Chem* **2015,** 71, (Pt 1), 3-8, 10.1107/S2053229614024218.

No syntax errors found.
Please wait while processing ....

CIF dictionary
Interpreting this report

Datablock: 1ver0-20683_usa-041

Bond precision:	C-C = 0.0017 A		Wavelength=1.54184
Cell:	a=15.8652(2)	b=14.6469(2)	c=16.7176(2)
	alpha=90	beta=90	gamma=90
Temperature:	100 K		

	Calculated	Reported
Volume	3884.77(9)	3884.77(9)
Space group	P b c a	P b c a
Hall group	-P 2ac 2ab	-P 2ac 2ab
Moiety formula	C25 H23 N O3	1.143(C25 H23 N O3)
Sum formula	C25 H23 N O3	C28.57 H26.29 N1.14 O3.43
Mr	385.44	440.51
Dx,g cm-3	1.318	1.318
Z	8	7
Mu (mm-1)	0.689	0.689
F000	1632.0	1632.0
F000'	1636.83	
h,k,lmax	19,18,21	19,18,21
Nref	4062	4053
Tmin,Tmax	0.825,0.896	
Tmin'	0.825	

Correction method= Not given

Data completeness= 0.998 Theta(max)= 76.220

R(reflections)= 0.0408(3728) wR2(reflections)= 0.1105(4053)

S = 1.029 Npar= 265

The following ALERTS were generated. Each ALERT has the format
test-name_ALERT_alert-type_alert-level.
Click on the hyperlinks for more details of the test.

Alert level C

PLAT052_ALERT_1_C Info on Absorption Correction Method Not Given Please Do !

Alert level G

CELLZ01_ALERT_1_G Difference between formula and atom_site contents detected.
CELLZ01_ALERT_1_G ALERT: check formula stoichiometry or atom site occupancies.
From the CIF: _cell_formula_units_Z 7
From the CIF: _chemical_formula_sum C28.57 H26.29 N1.14 O3.43
TEST: Compare cell contents of formula and atom_site data

atom	Z*formula	cif sites	diff
C	199.99	200.00	-0.01
H	184.03	184.00	0.03
N	7.98	8.00	-0.02
O	24.01	24.00	0.01

PLAT007_ALERT_5_G Number of Unrefined Donor-H Atoms .... 1 Report
PLAT045_ALERT_1_G Calculated and Reported Z Differ by a Factor ... 1.143 Check
PLAT793_ALERT_4_G Model has Chirality at C4 (Centro SPGR) S Verify
PLAT793_ALERT_4_G Model has Chirality at C5 (Centro SPGR) S Verify
PLAT912_ALERT_4_G Missing # of FCF Reflections Above STh/L= 0.600 9 Note
PLAT978_ALERT_2_G Number C-C Bonds with Positive Residual Density. 22 Info

0 ALERT level A = Most likely a serious problem - resolve or explain
0 ALERT level B = A potentially serious problem, consider carefully
1 ALERT level C = Check. Ensure it is not caused by an omission or oversight
8 ALERT level G = General information/check it is not something unexpected

4 ALERT type 1 CIF construction/syntax error, inconsistent or missing data
1 ALERT type 2 Indicator that the structure model may be wrong or deficient
0 ALERT type 3 Indicator that the structure quality may be low
3 ALERT type 4 Improvement, methodology, query or suggestion
1 ALERT type 5 Informative message, check

It is advisable to attempt to resolve as many as possible of the alerts in all categories. Often the minor

alerts point to easily fixed oversights, errors and omissions in your CIF or refinement strategy, so attention to these fine details can be worthwhile. In order to resolve some of the more serious problems it may be necessary to carry out additional measurements or structure refinements. However, the purpose of your study may justify the reported deviations and the more serious of these should normally be commented upon in the discussion or experimental section of a paper or in the "special_details" fields of the CIF. checkCIF was carefully designed to identify outliers and unusual parameters, but every test has its limitations and alerts that are not important in a particular case may appear. Conversely, the absence of alerts does not guarantee there are no aspects of the results needing attention. It is up to the individual to critically assess their own results and, if necessary, seek expert advice.

Publication of your CIF in IUCr journals

A basic structural check has been run on your CIF. These basic checks will be run on all CIFs submitted for publication in IUCr journals (*Acta Crystallographica*, *Journal of Applied Crystallography*, *Journal of Synchrotron Radiation*); however, if you intend to submit to *Acta Crystallographica Section C* or *E* or *IUCrData*, you should make sure that full publication checks are run on the final version of your CIF prior to submission.

Publication of your CIF in other journals

Please refer to the *Notes for Authors* of the relevant journal for any special instructions relating to CIF submission.

PLATON version of 12/09/2022; check.def file version of 09/08/2022

Datablock 1ver0-20683_usa-041 - ellipsoid plot

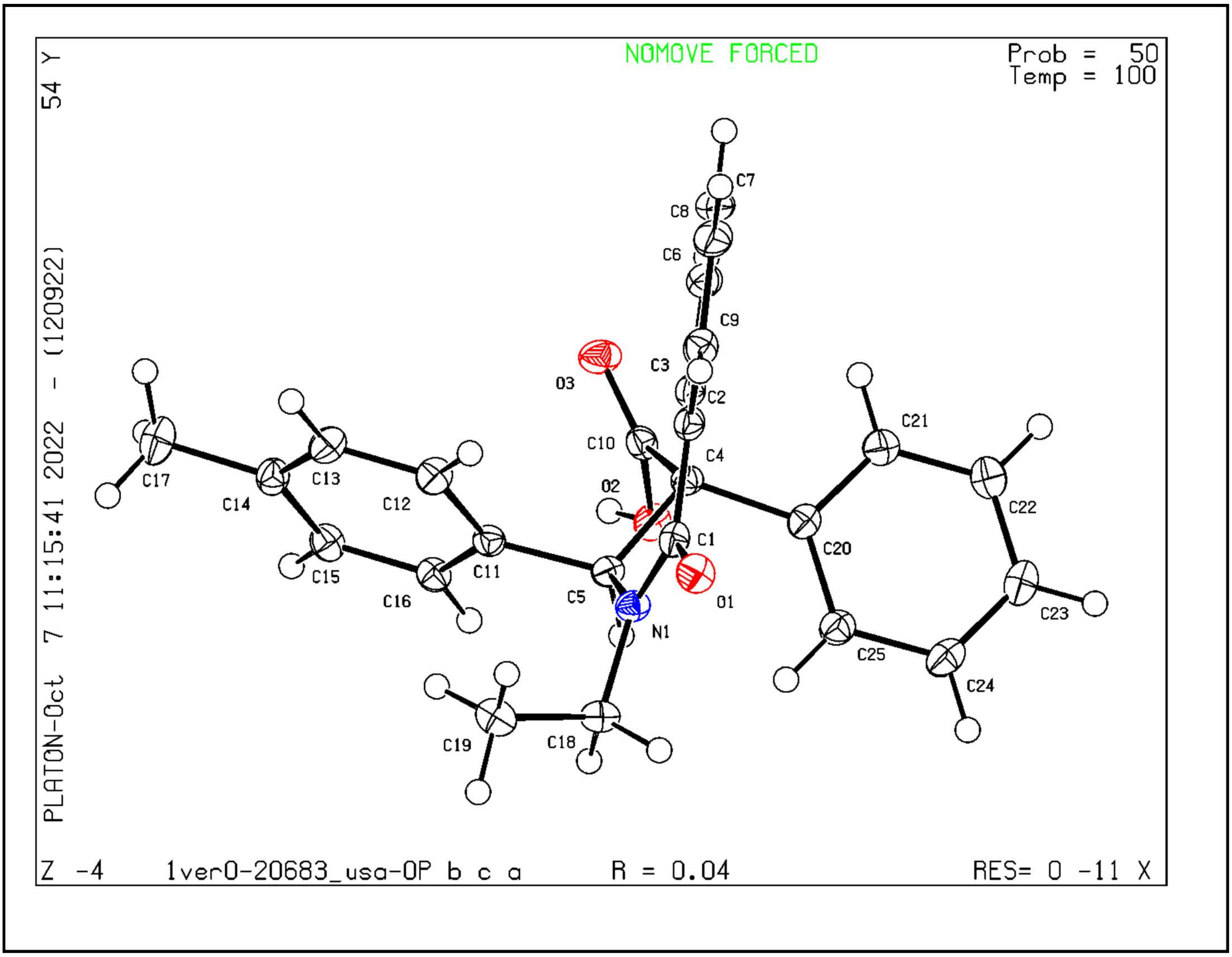

3. Copies of ^{1}H, ^{13}C and ^{19}F NMR spectra

^{1}H and ^{13}C NMR spectra of compound **10a**

1H NMR, 400.13 MHz, DMSO-d6

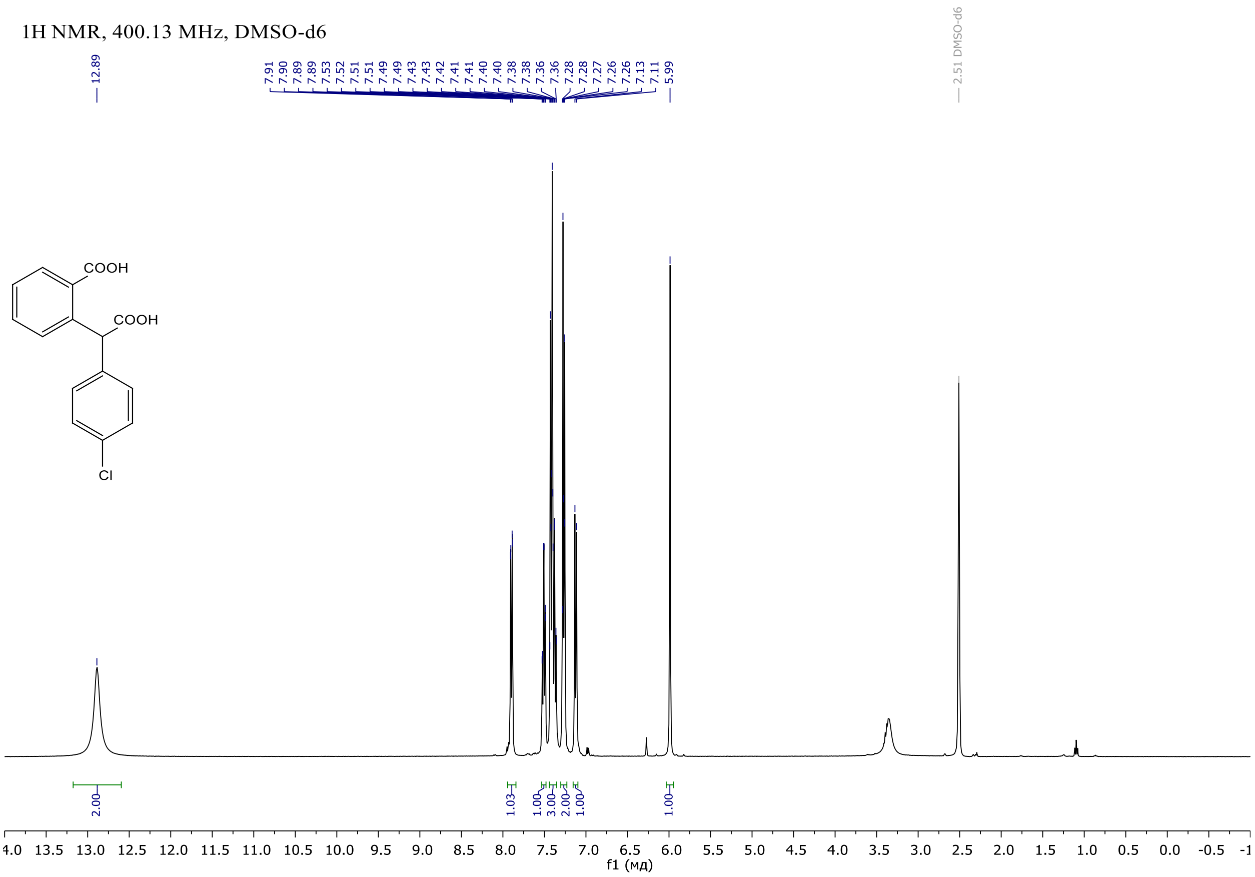

13C NMR, 100.61 MHz, DMSO-d6

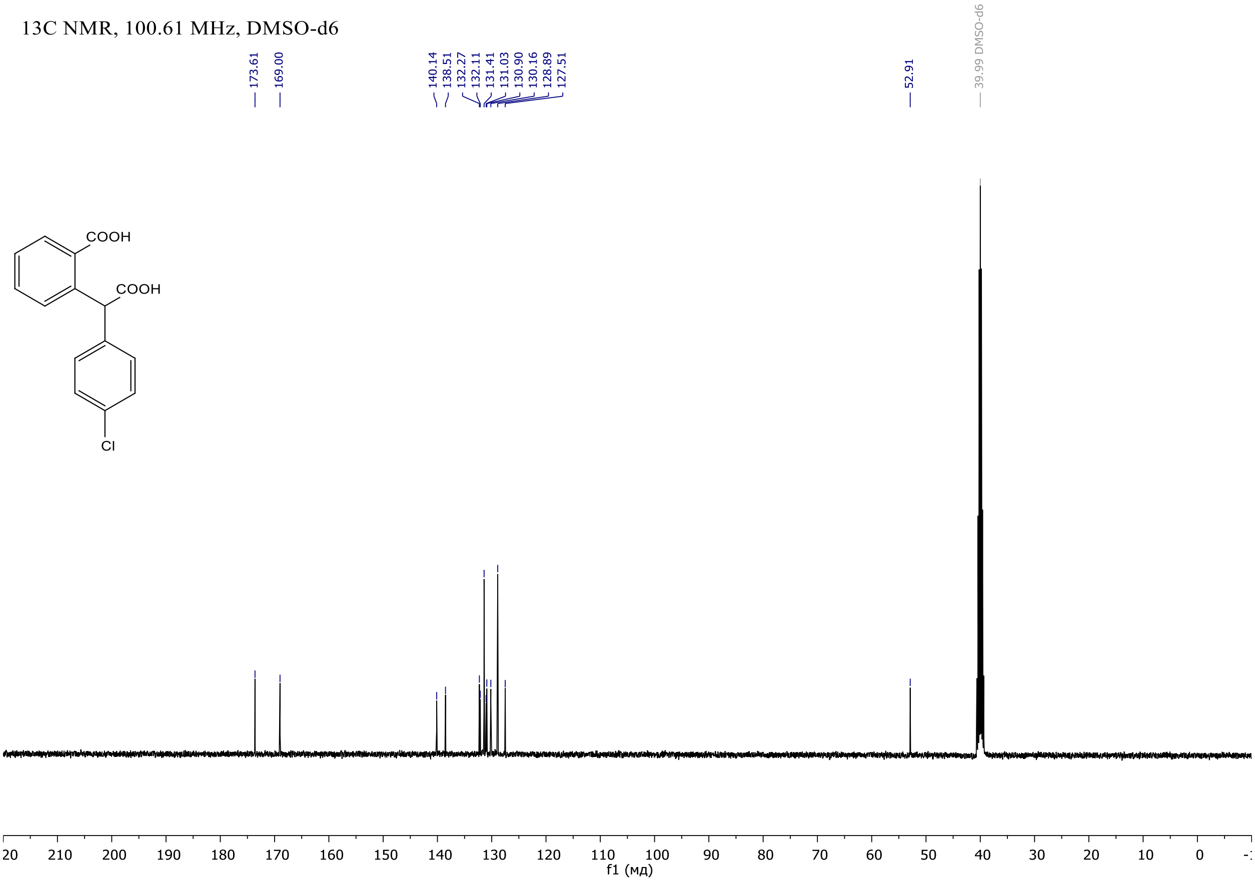

^{1}H and ^{13}C NMR spectra of compound **10b**

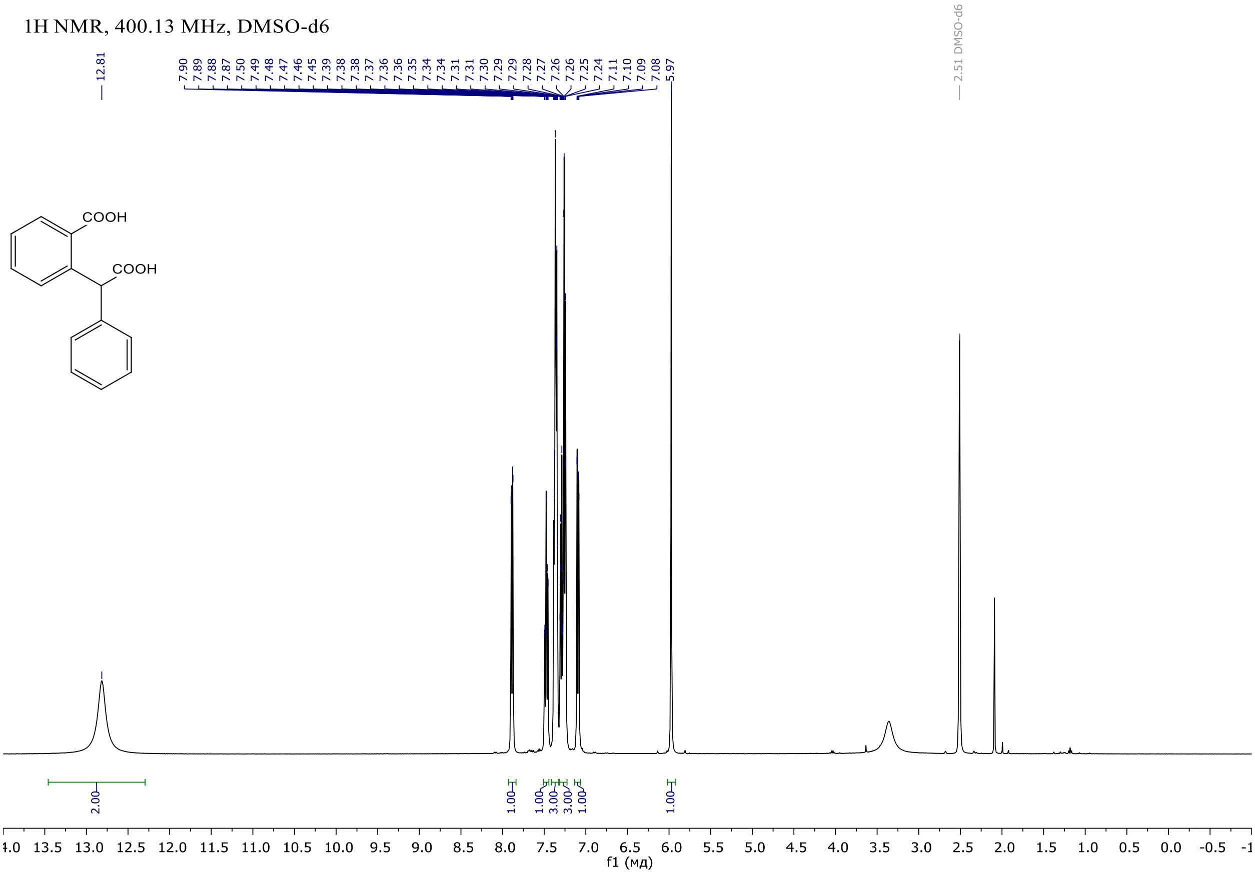

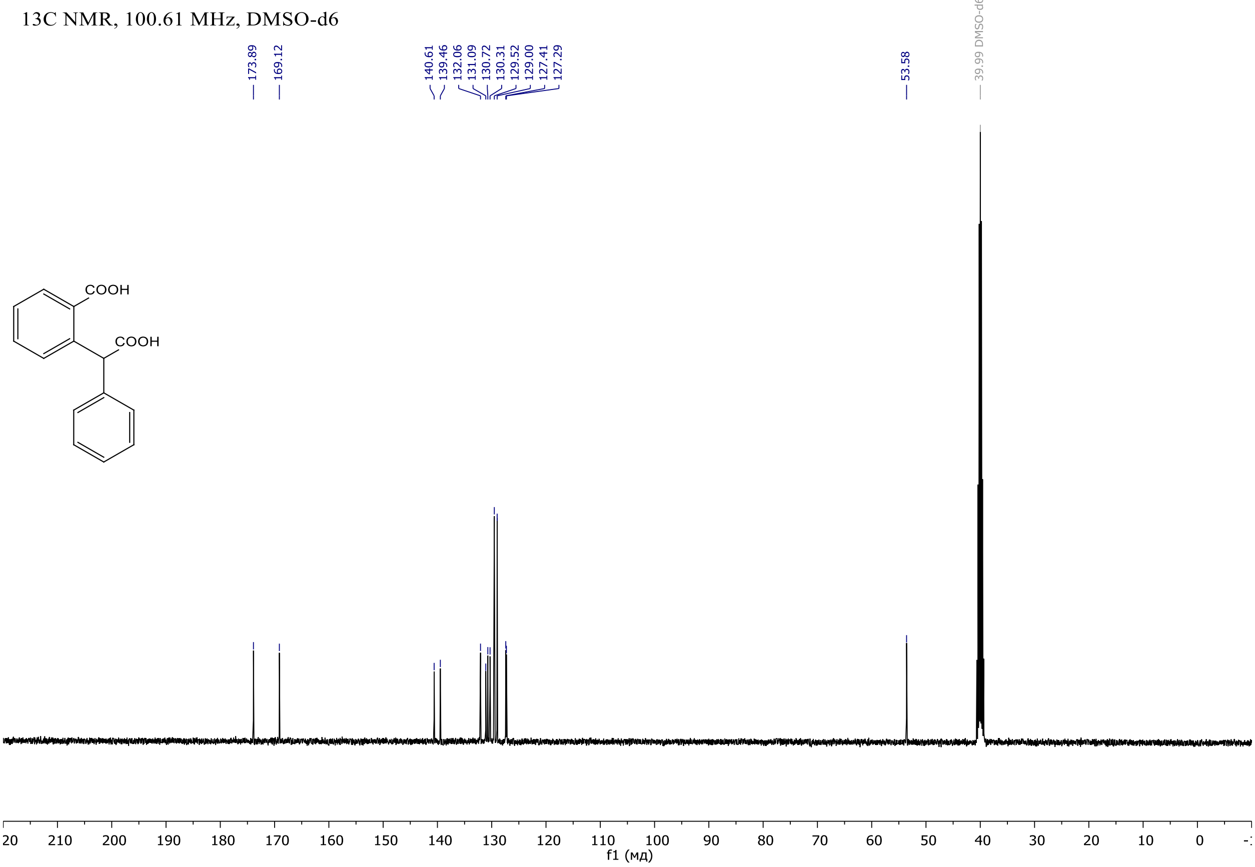

^{1}H and ^{13}C NMR spectra of compound **10c**

1H NMR, 400.13 MHz, DMSO-d6

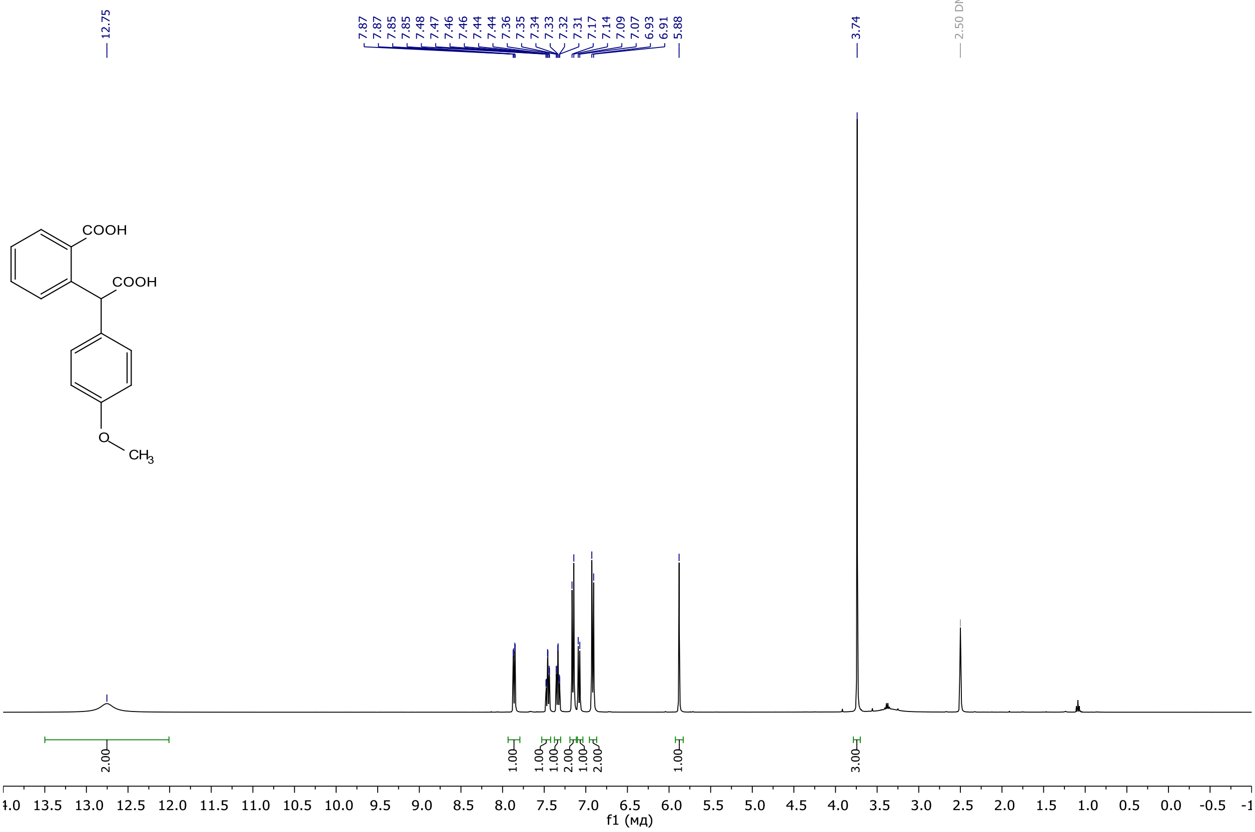

13C NMR, 100.61 MHz, DMSO-d6

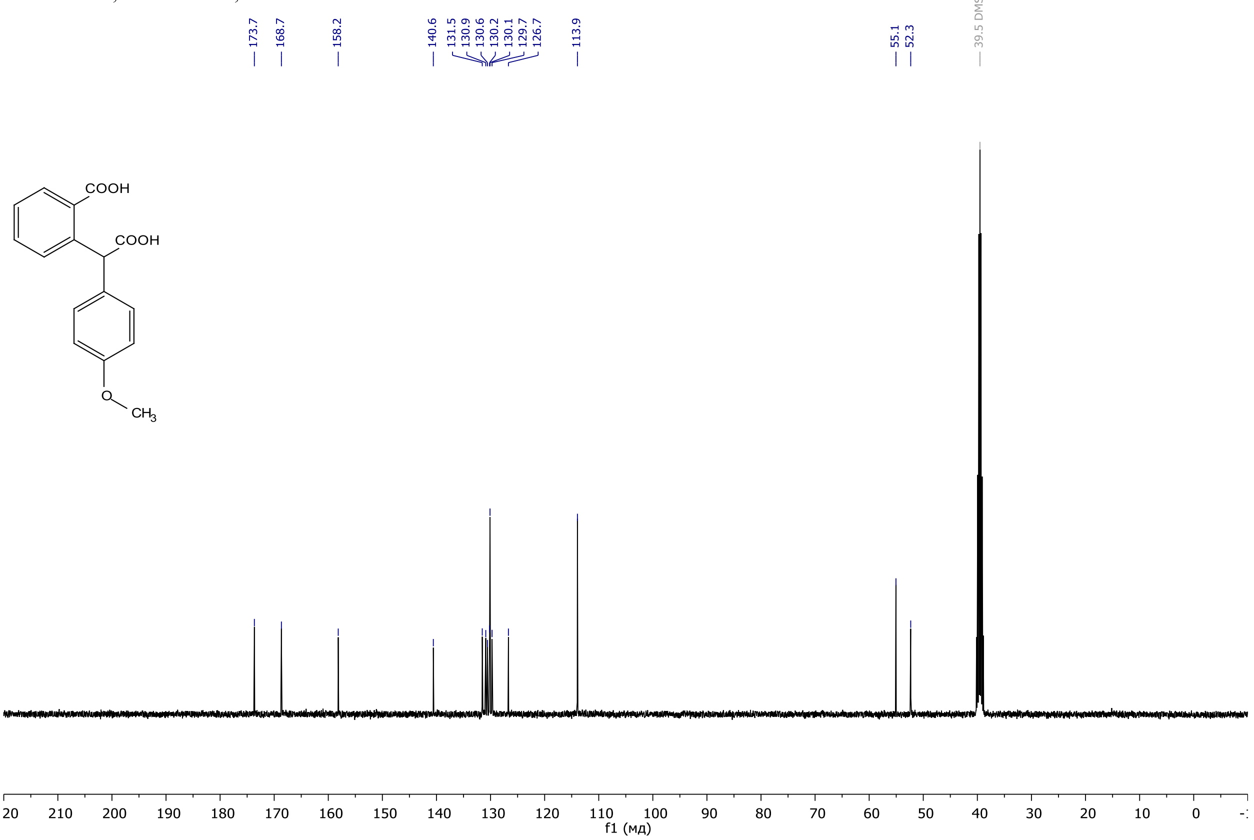

^{1}H and ^{13}C NMR spectra of compound **10d**

1H NMR, 400.13 MHz, DMSO-d6

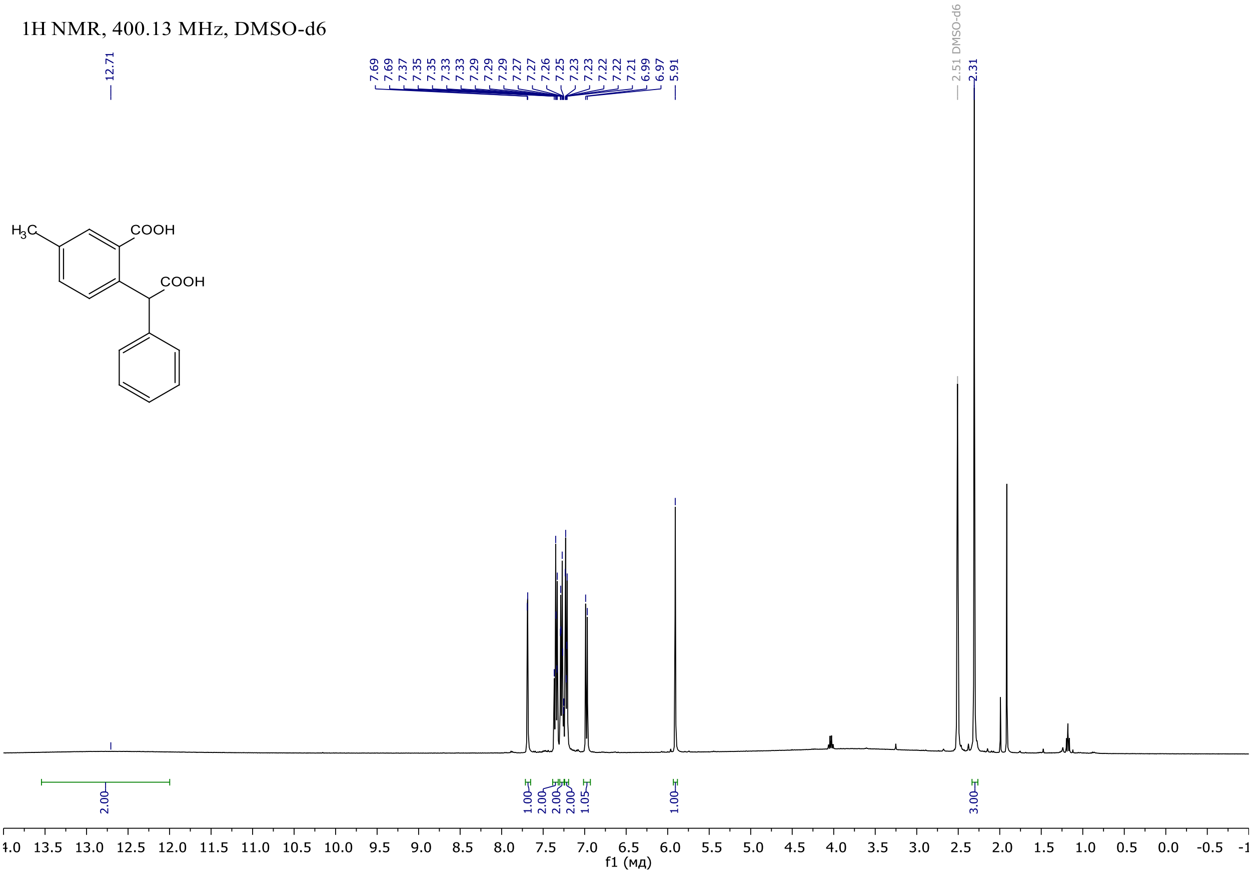

13C NMR. 100.61 MHz, DMSO-d6

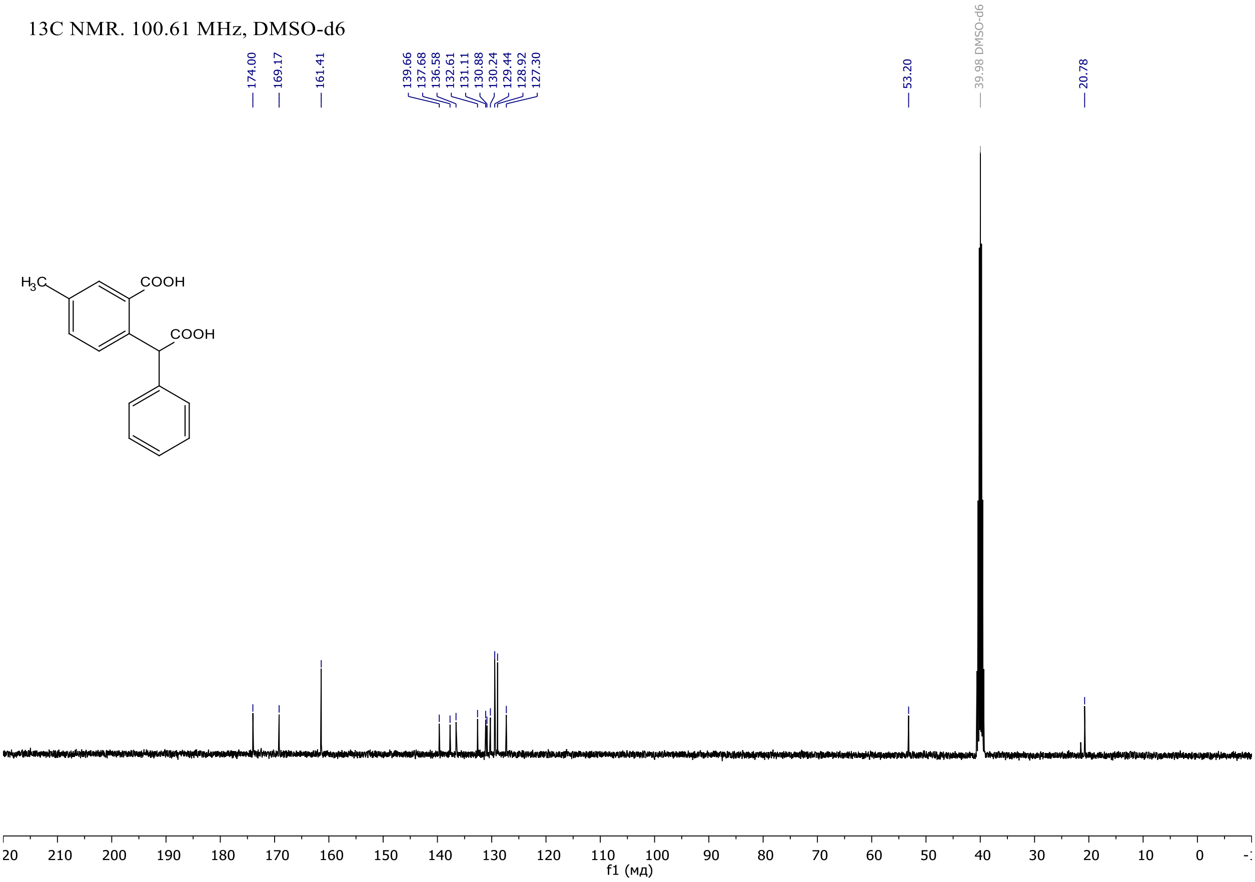

^{1}H, ^{13}C and ^{19}F NMR spectra of compound **10e**

1H NMR, 400.13 MHz, DMSO-d6

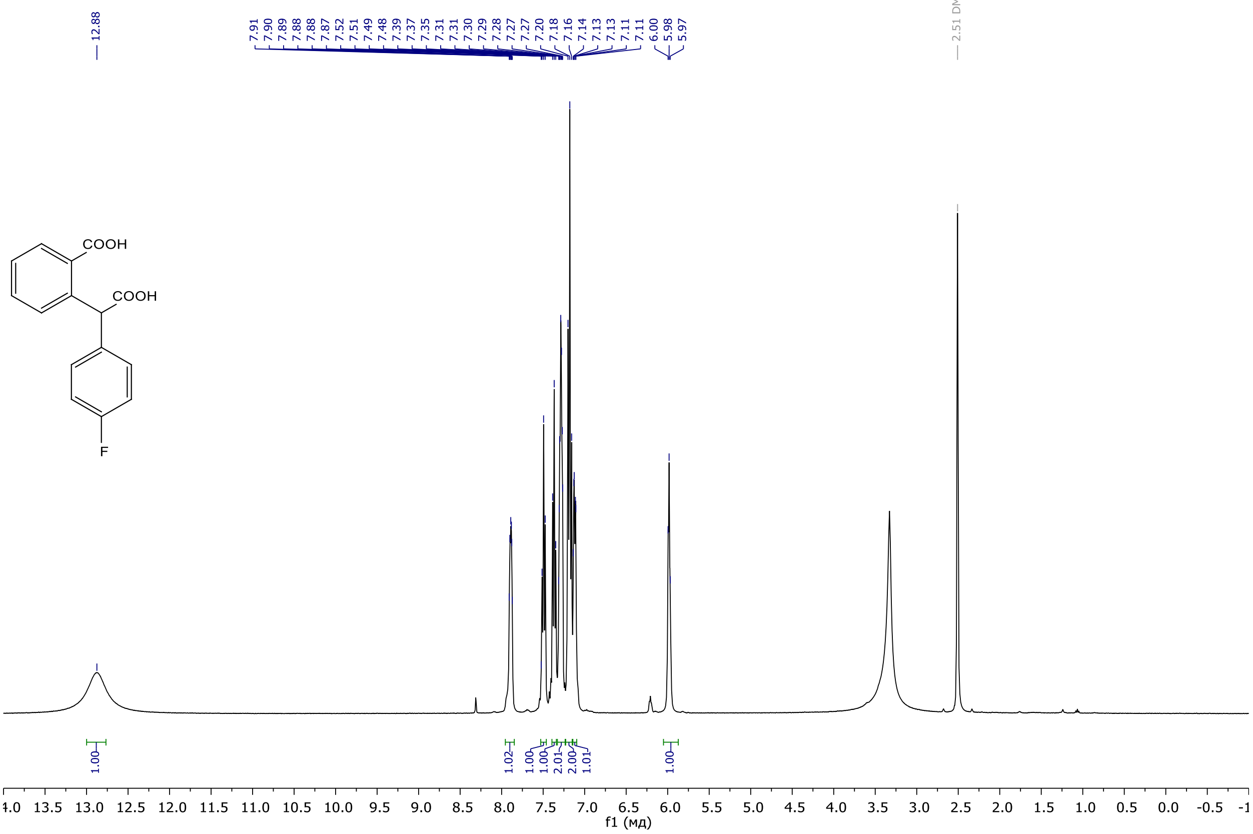

13C NMR, 100.61 MHz, DMSO-d6

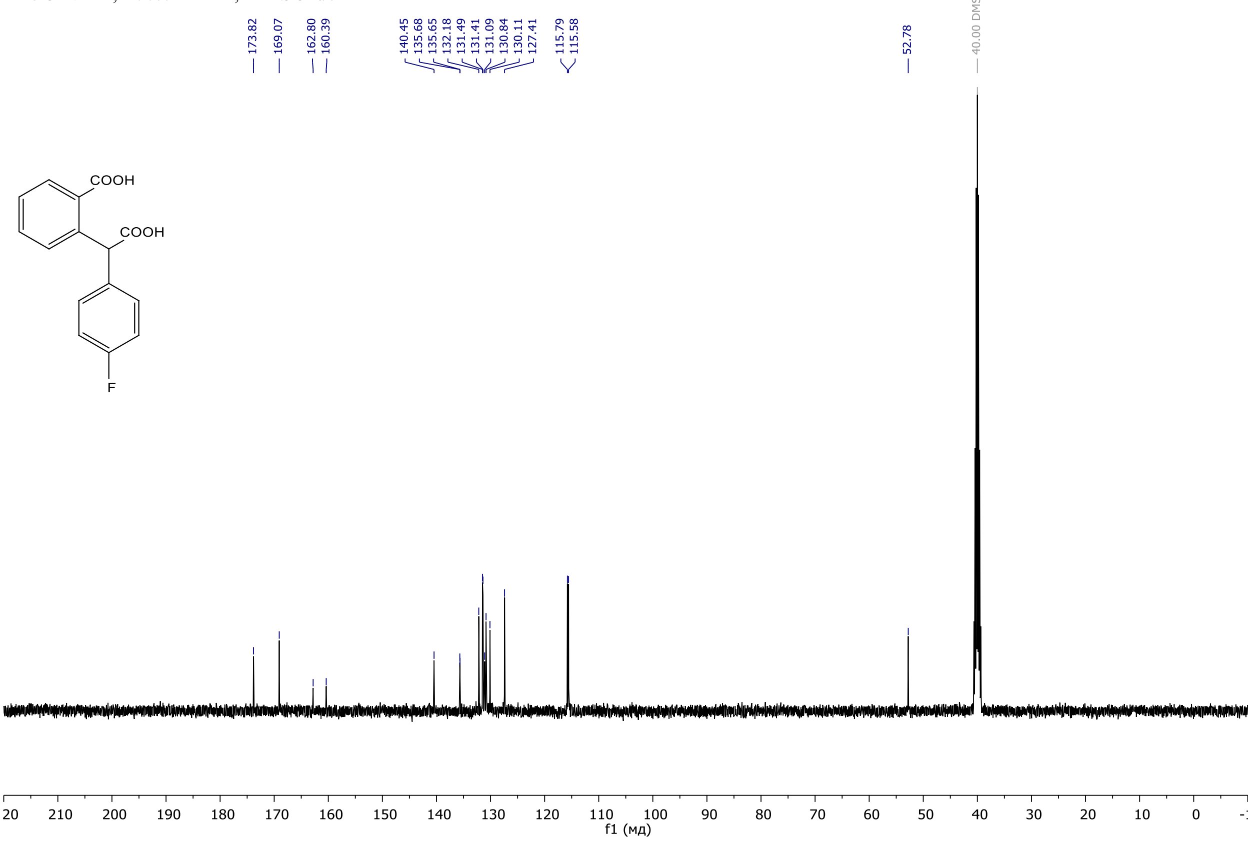

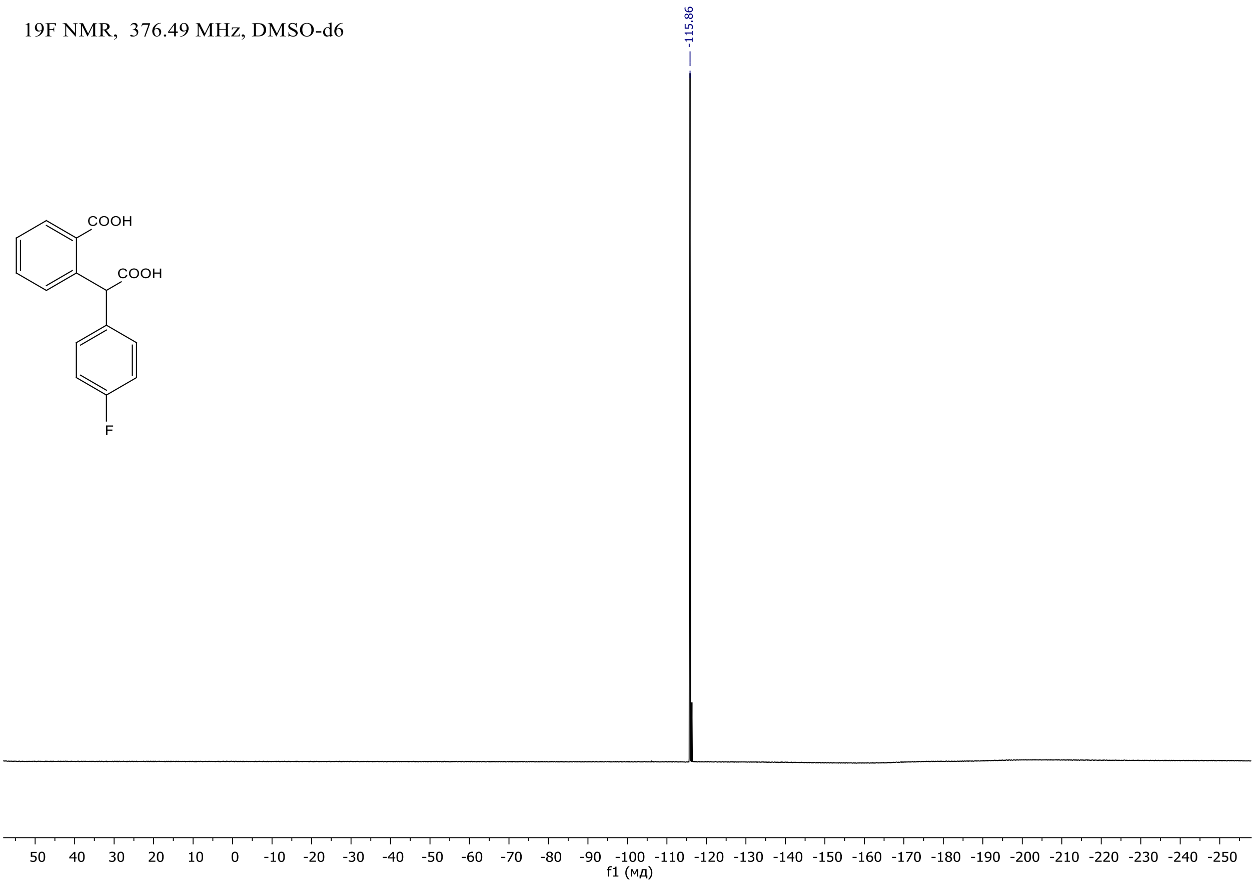
19F NMR, 376.49 MHz, DMSO-d6
-115.86
COOH
COOH
F
50 40 30 20 10 0 -10 -20 -30 -40 -50 -60 -70 -80 -90 -100 -110 -120 -130 -140 -150 -160 -170 -180 -190 -200 -210 -220 -230 -240 -250
f1 (мд)

^1H and ^{13}C NMR spectra of compound **10f**

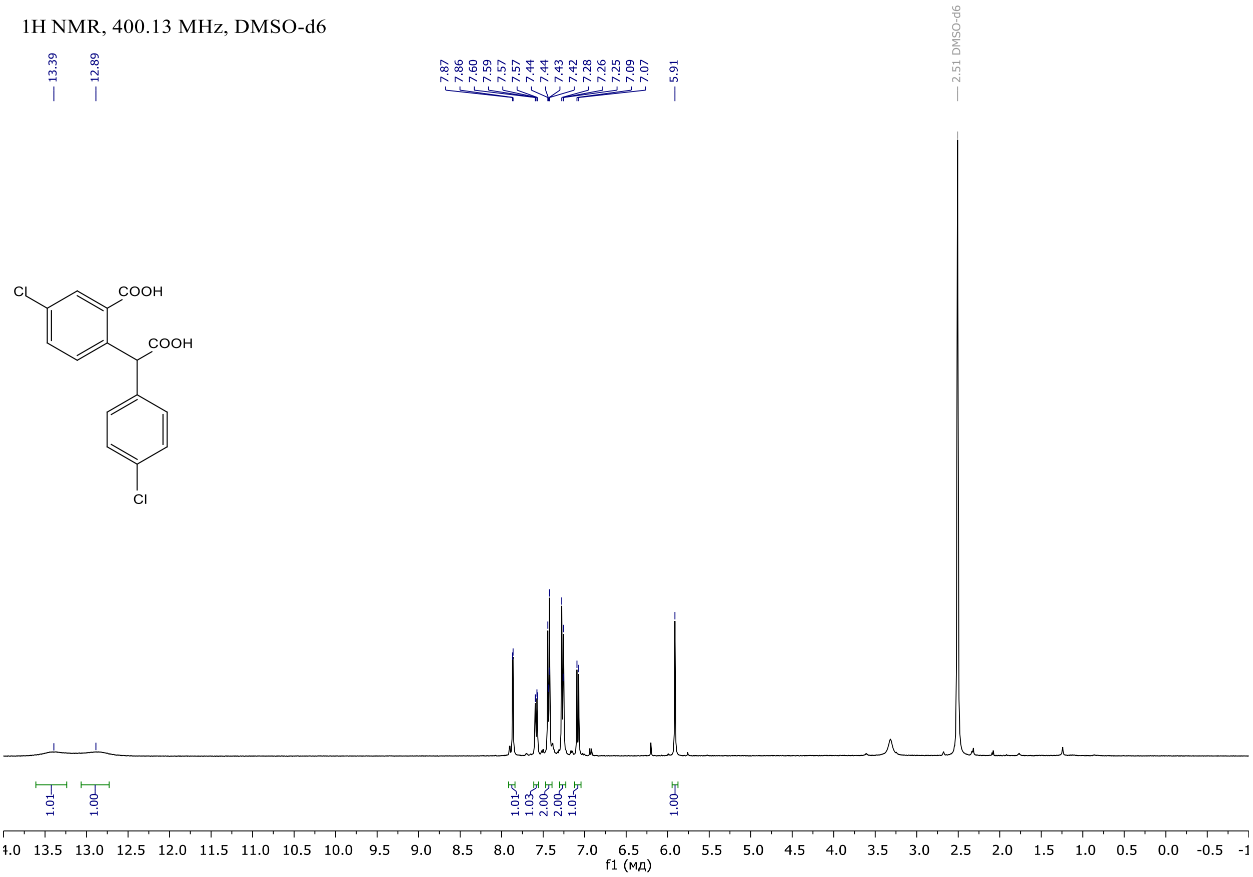

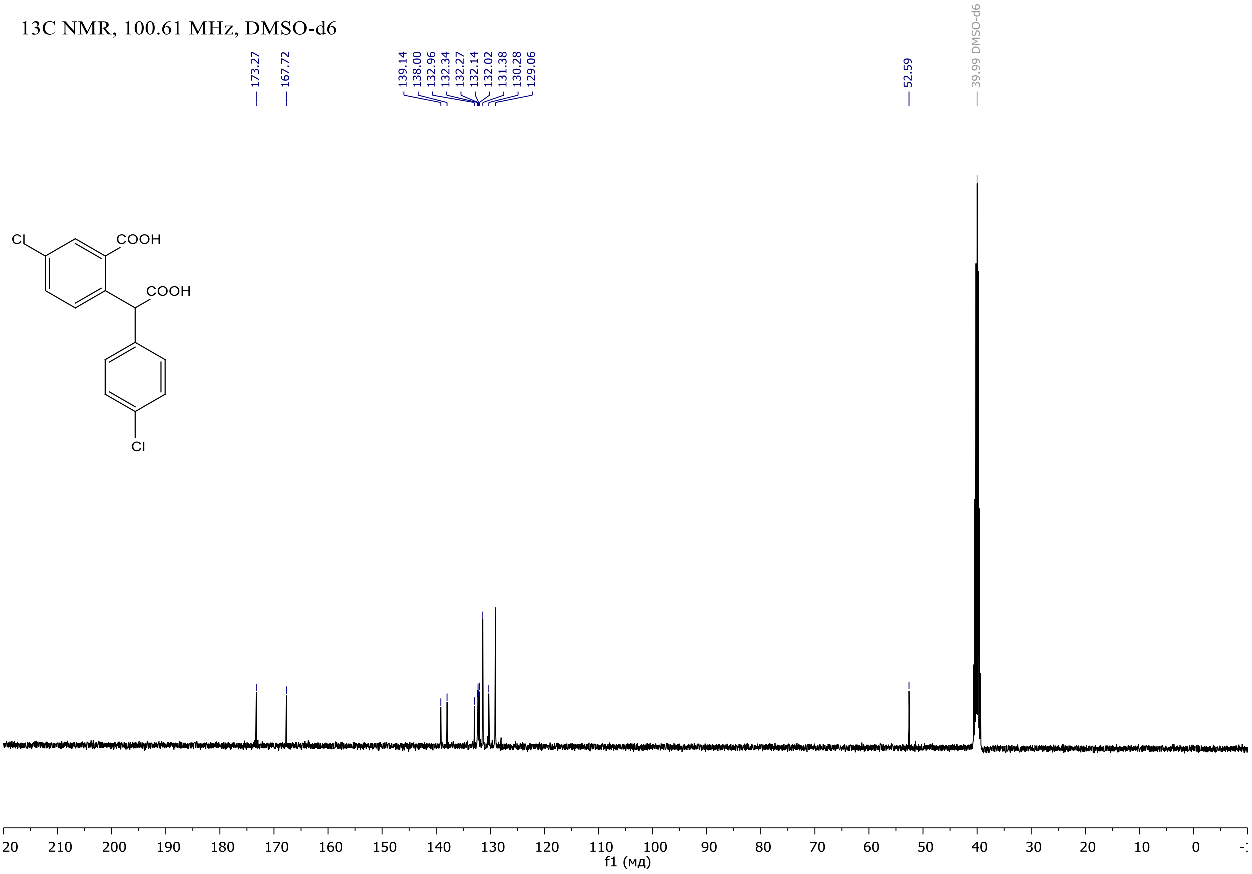

^{1}H and ^{13}C NMR spectra of compound **9a**

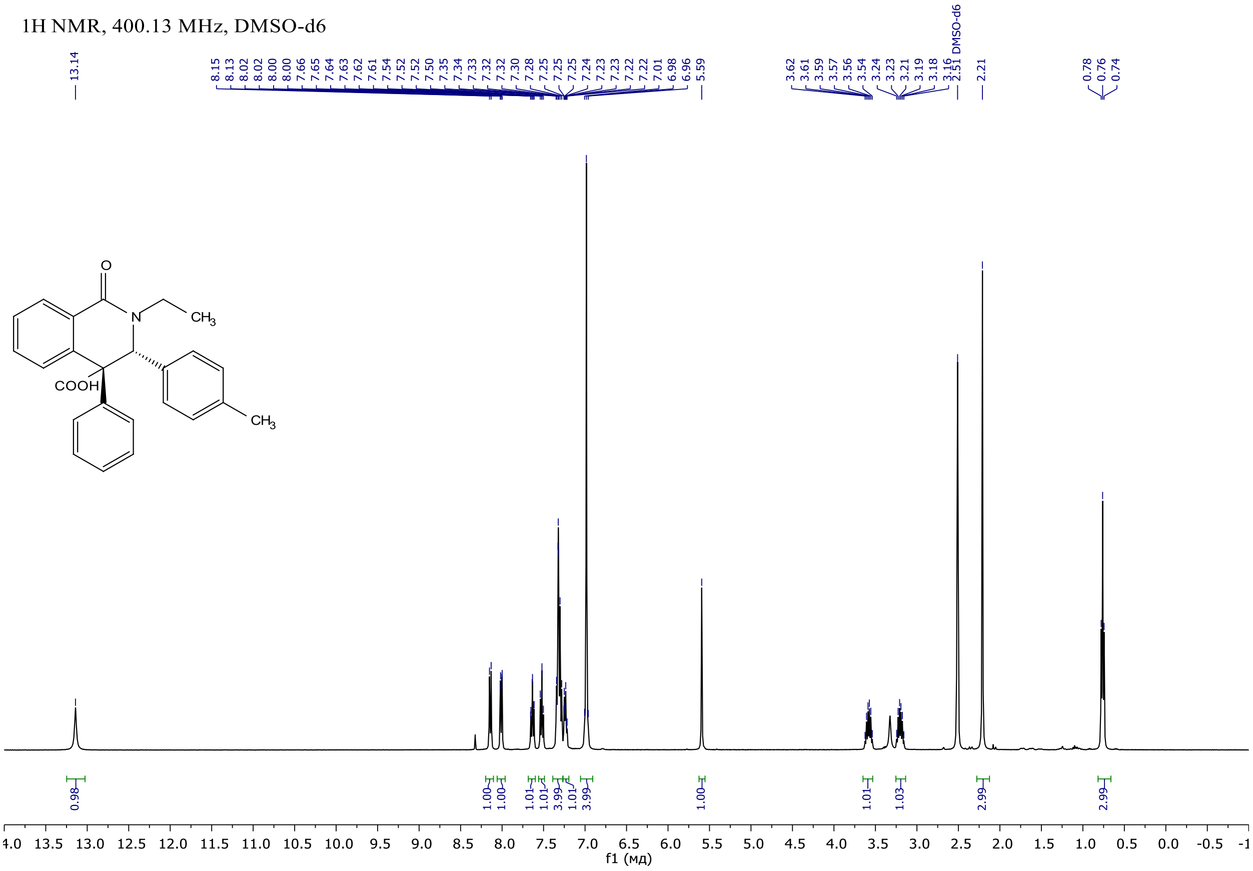

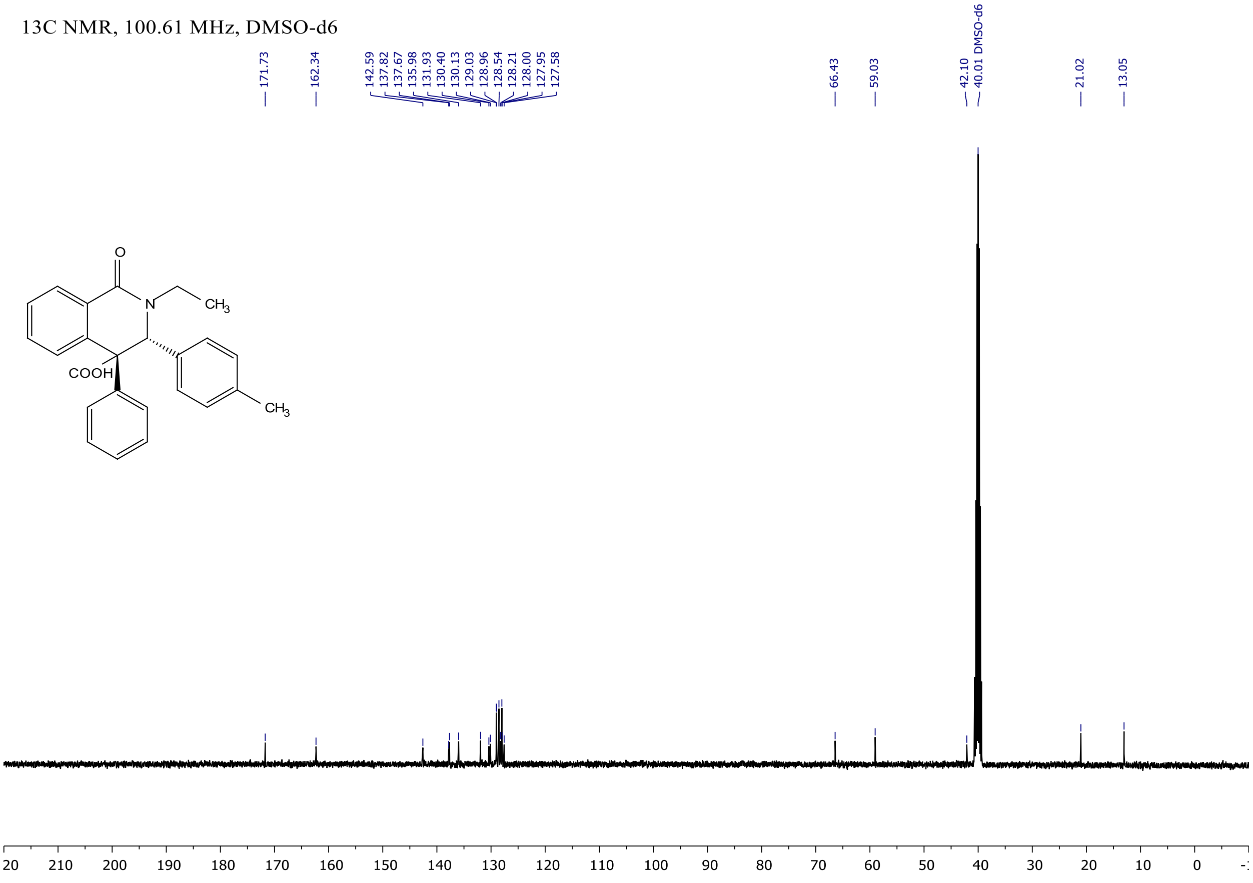

^{1}H and ^{13}C NMR spectra of compound **9b**

1H NMR, 400.13 MHz, DMSO-d6

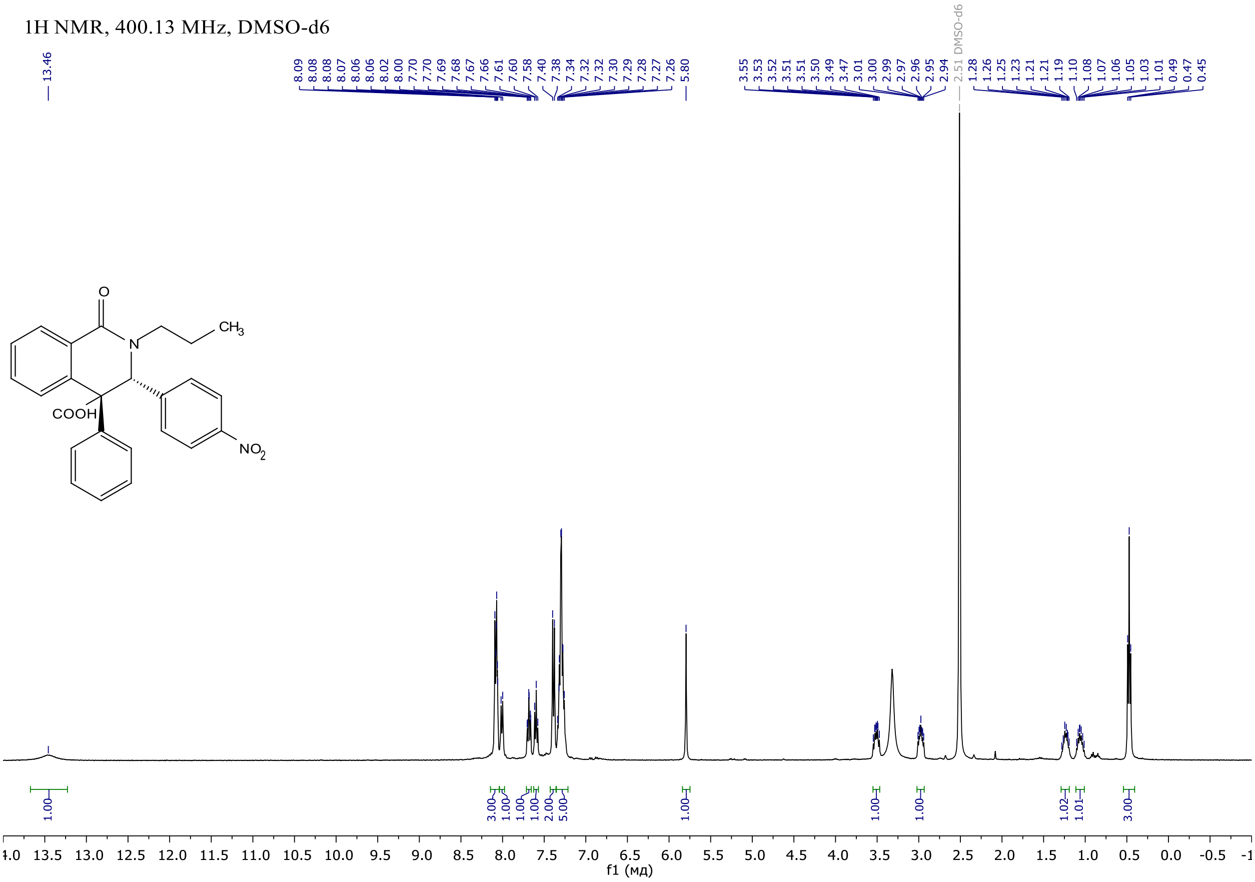

13C NMR, 100.61 MHz, DMSO-d6

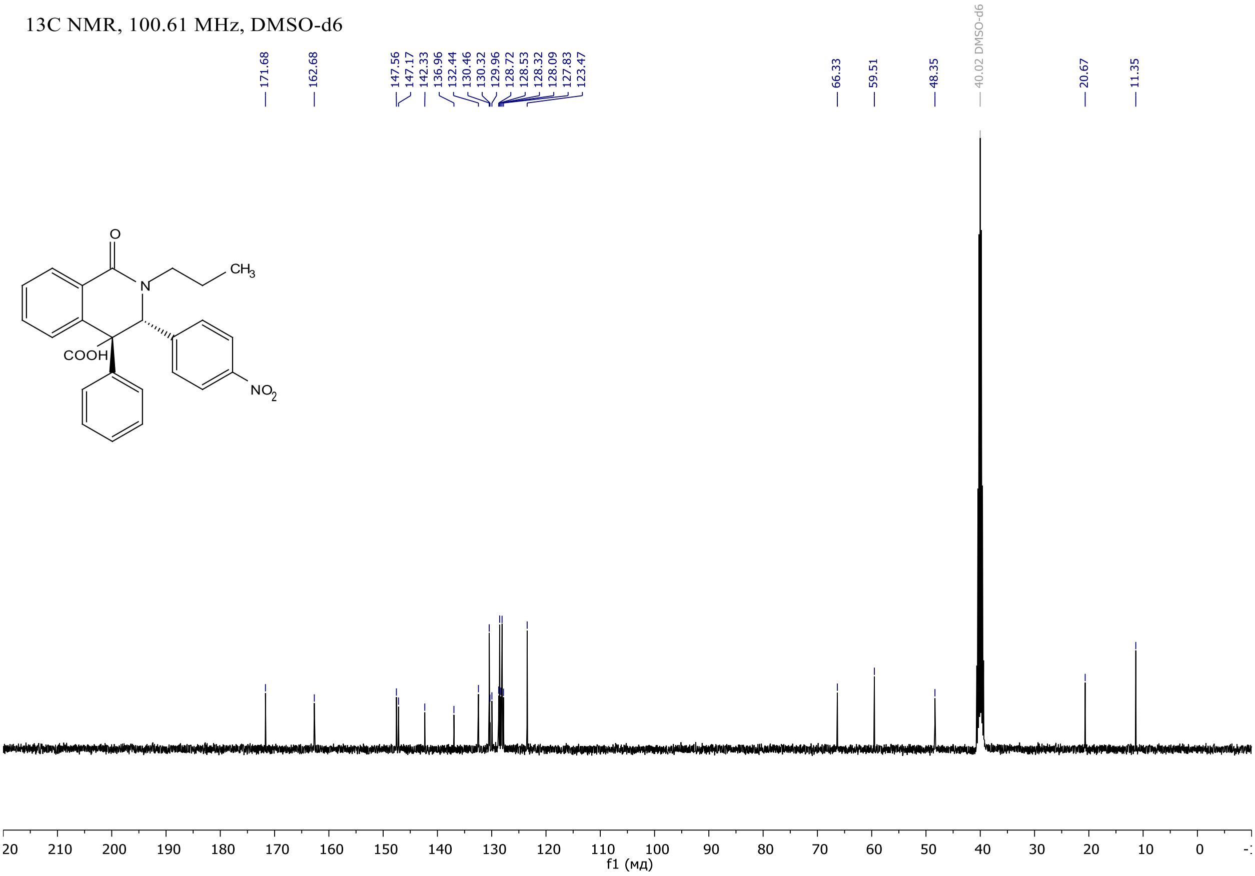

^1H and ^{13}C NMR spectra of compound **9c**

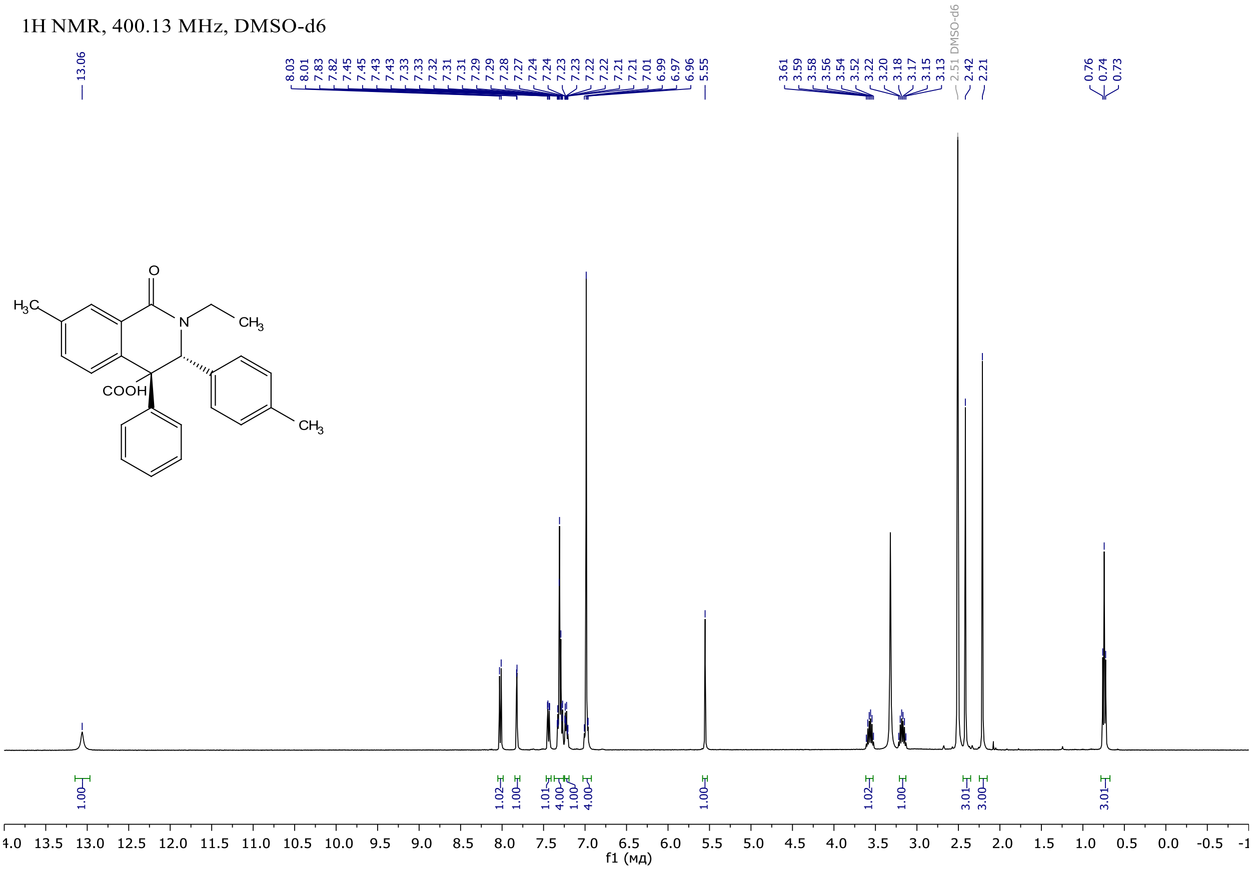

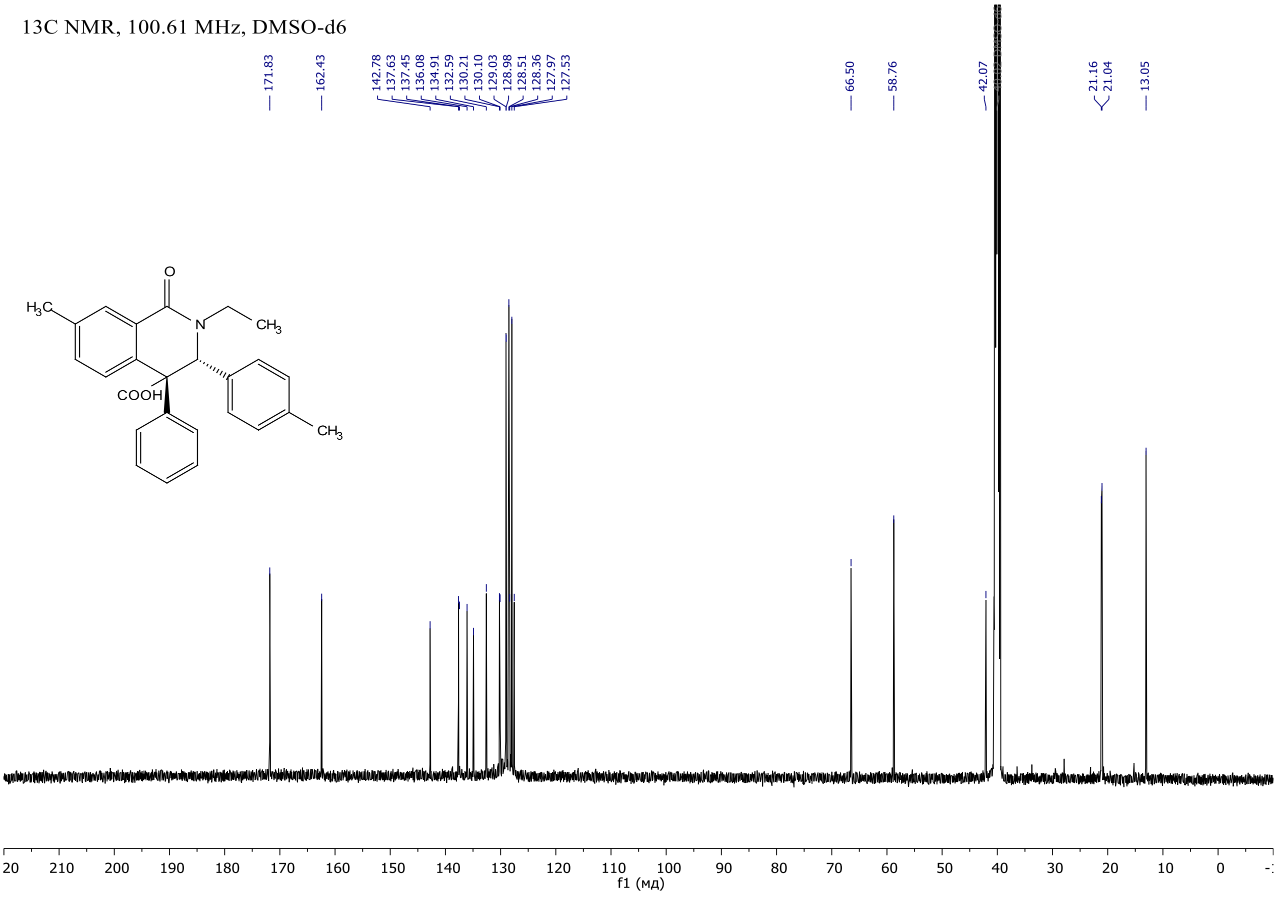

^{1}H and ^{13}C NMR spectra of compound **9d**

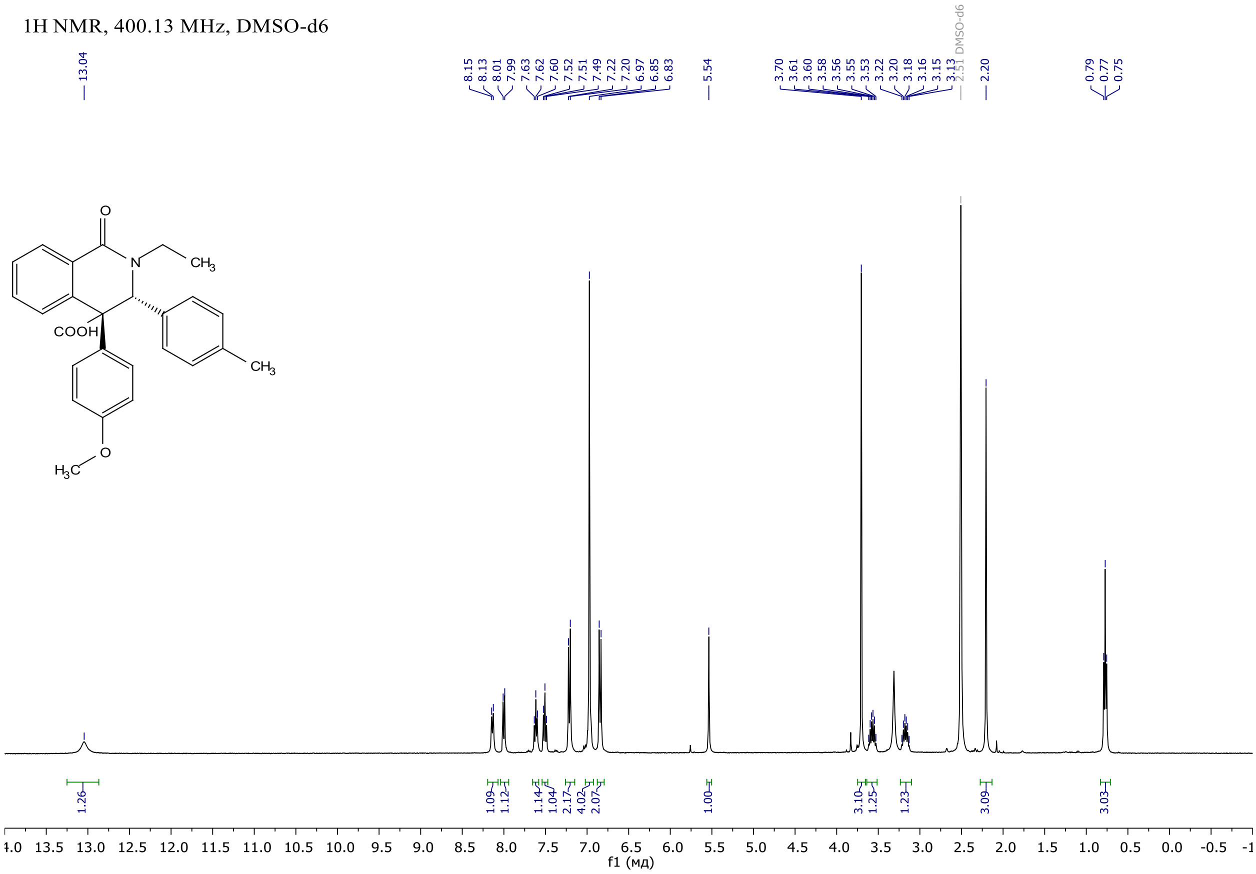

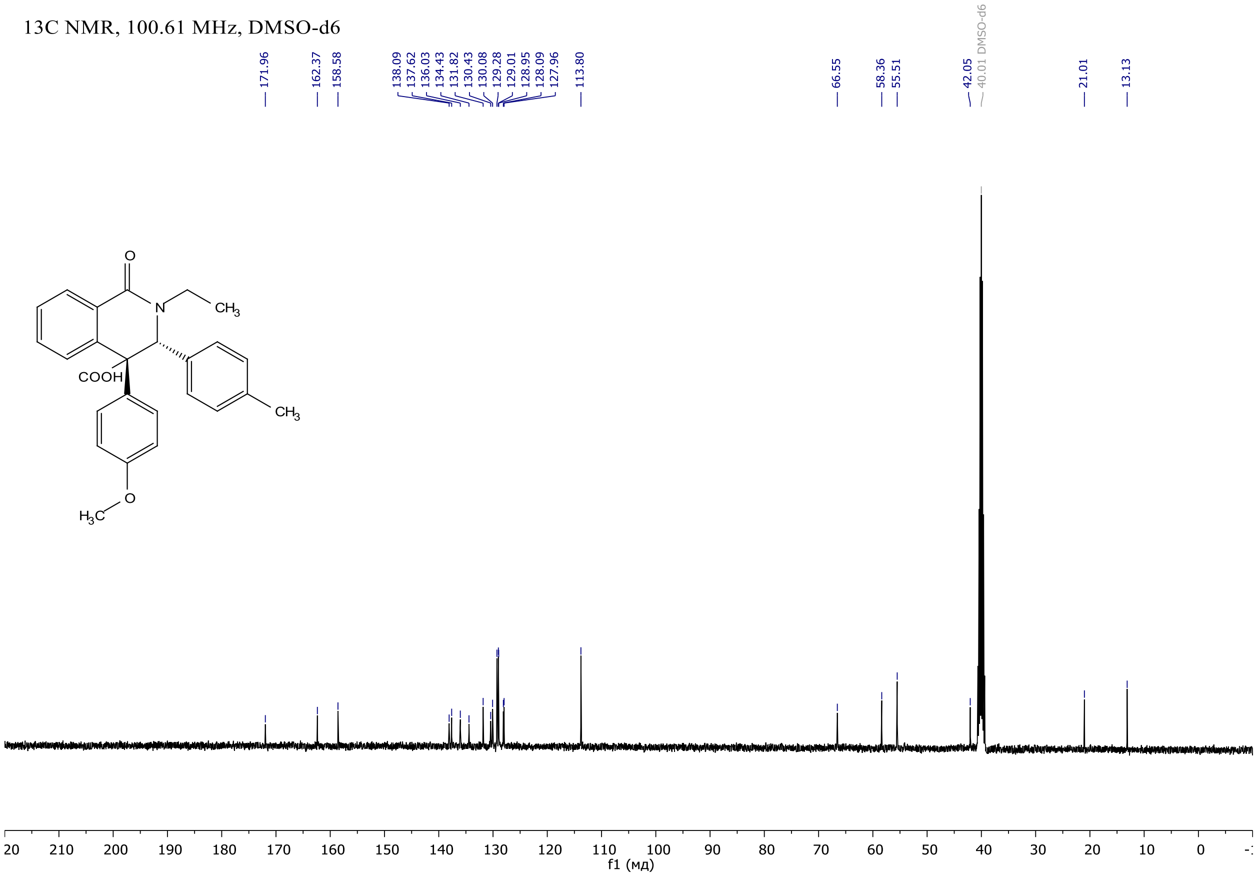

^{1}H and ^{13}C NMR spectra of compound **9e**

1H NMR, 400.13 MHz, DMSO-d6

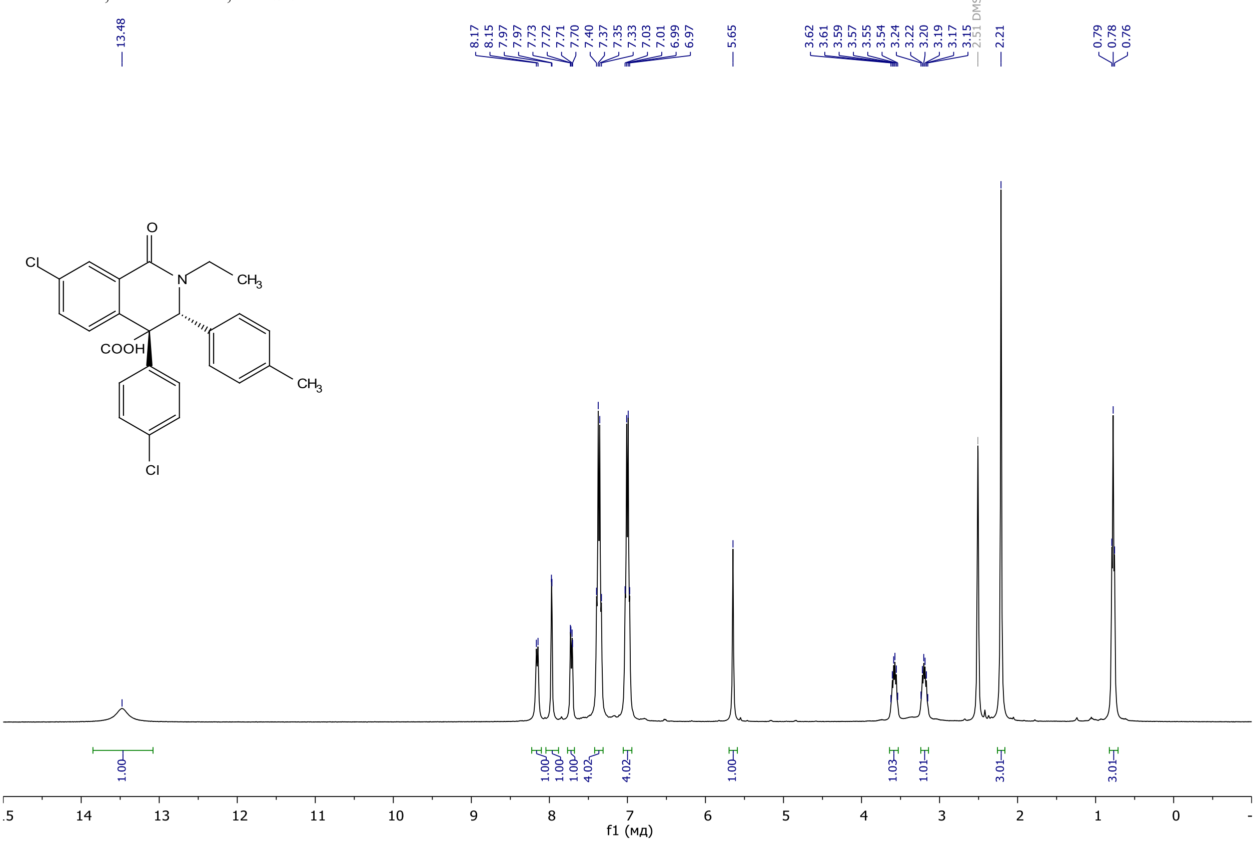

13C NMR, 100.61 MHz, DMSO-d6

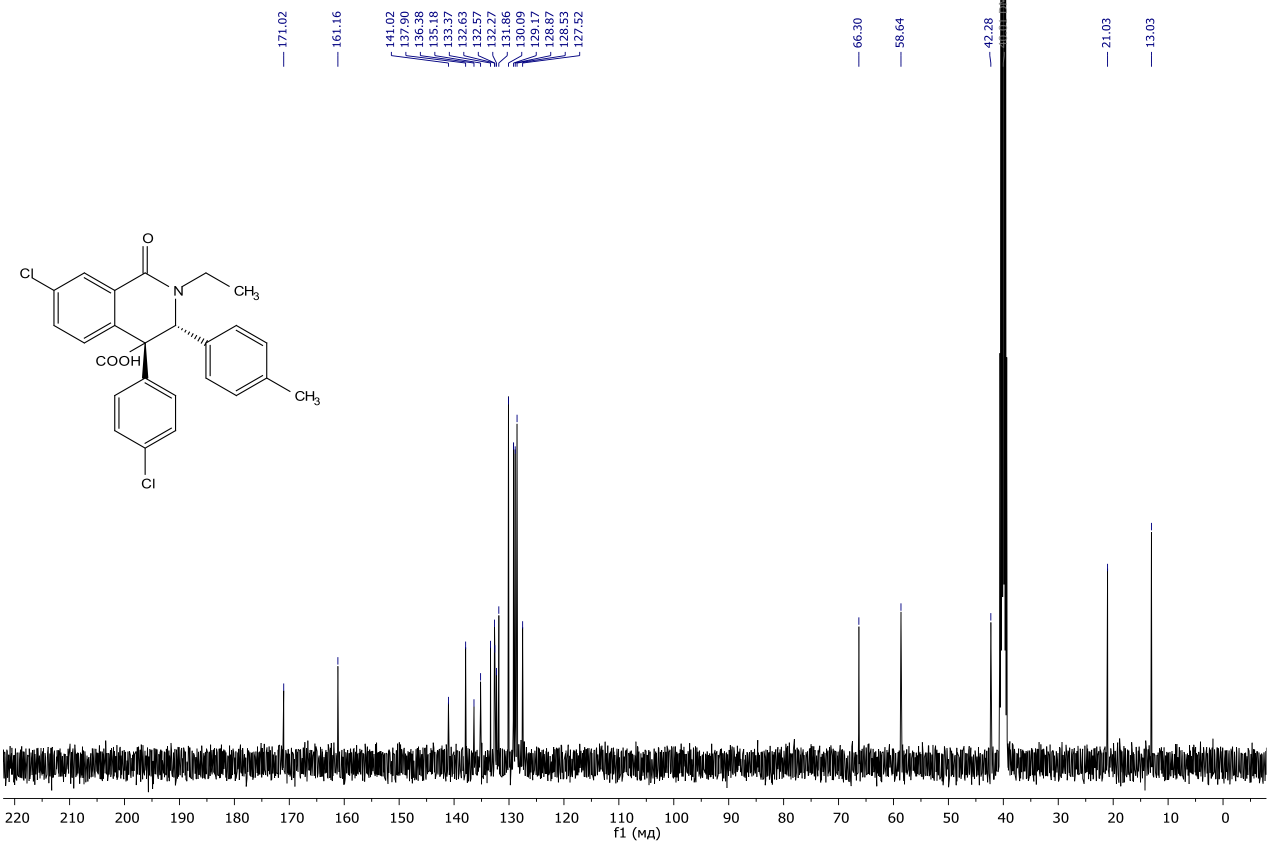

^{1}H and ^{13}C NMR spectra of compound **9f**

1H NMR, 400.13 MHz, DMSO-d6

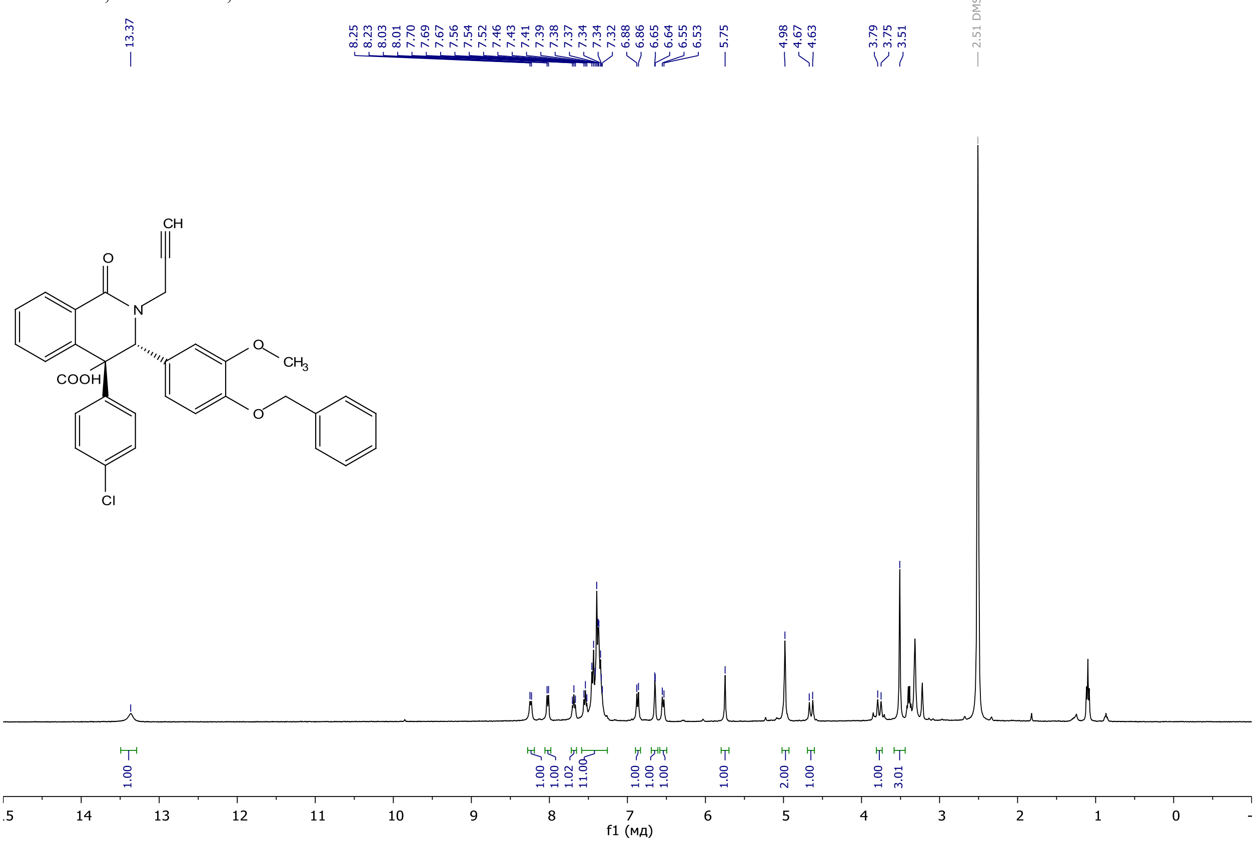

13C NMR, 125.73 MHz, DMSO-d6

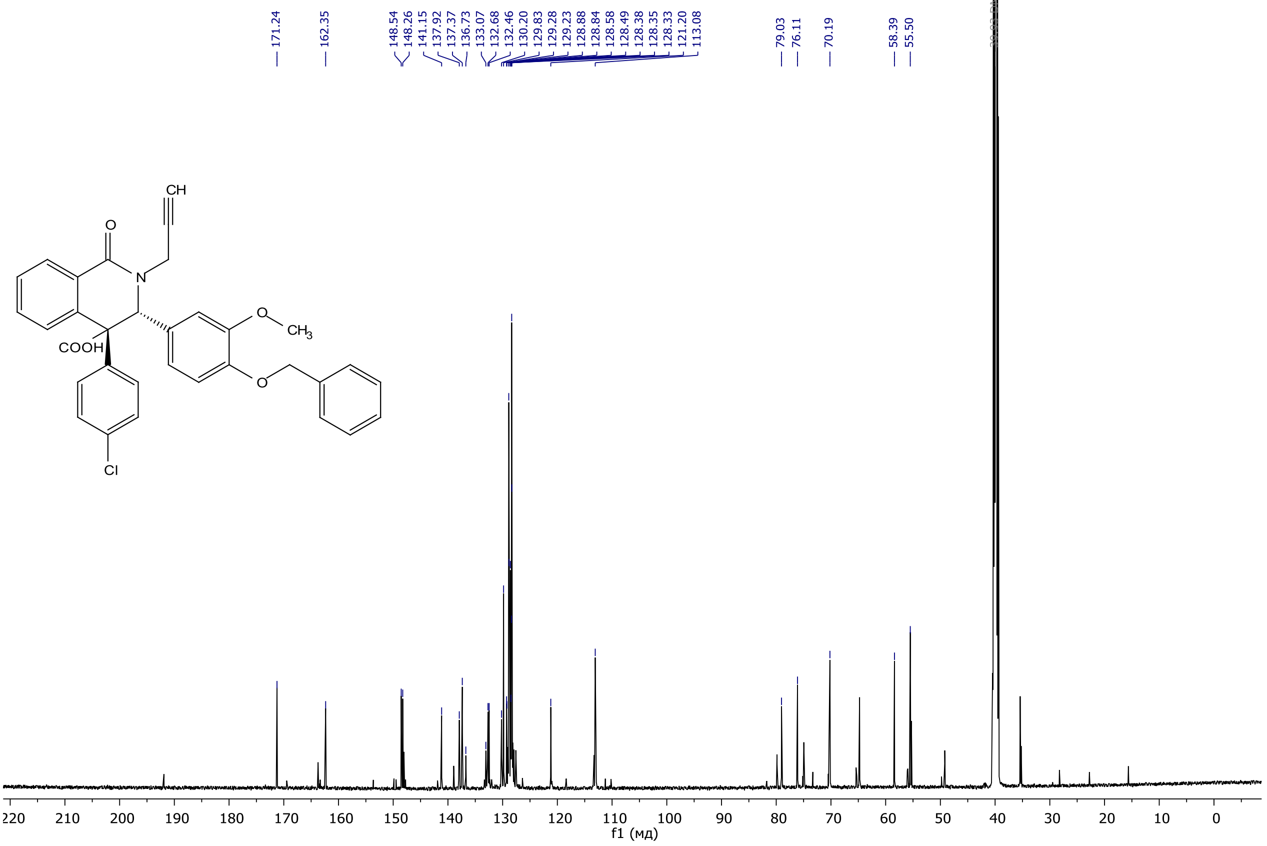

^{1}H and ^{13}C NMR spectra of compound **9g**

1H NMR, 400.13 MHz, DMSO-d6

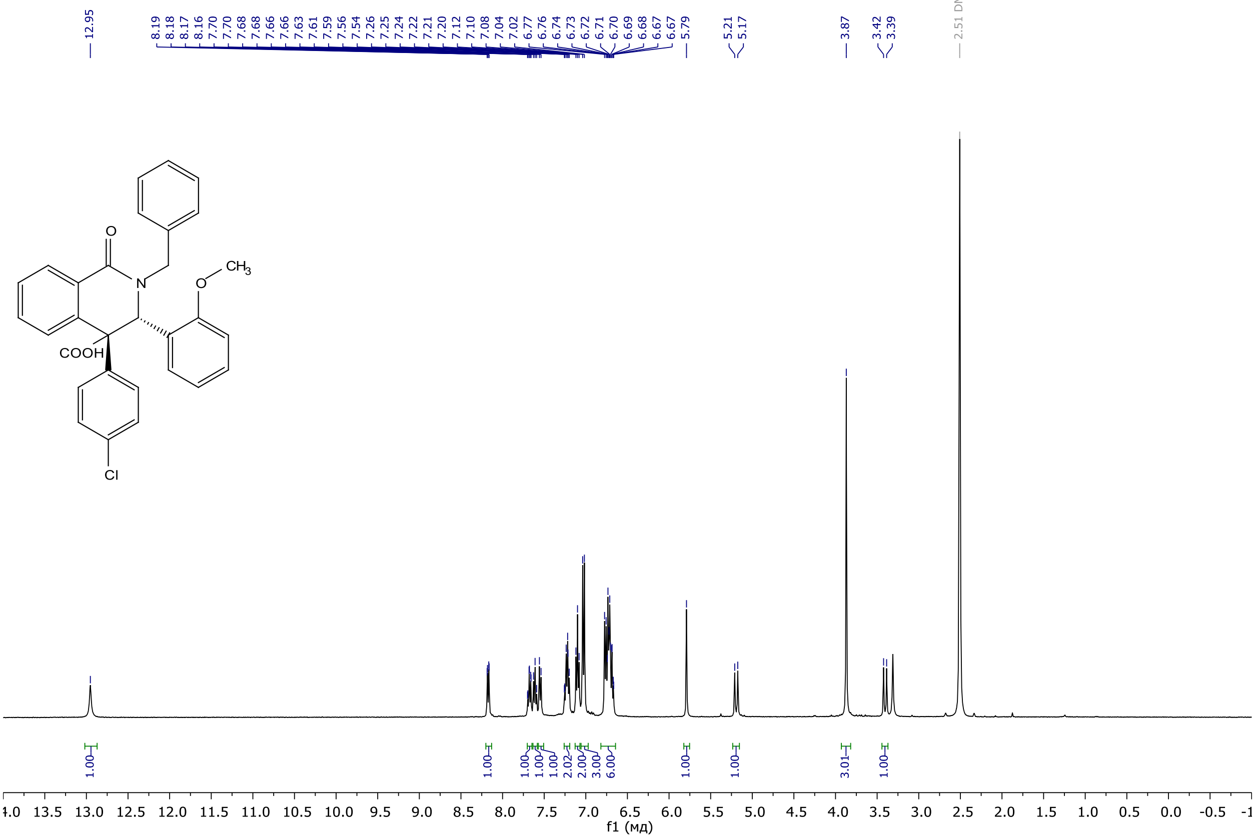

13C NMR, 100.61 MHz, DMSO-d6

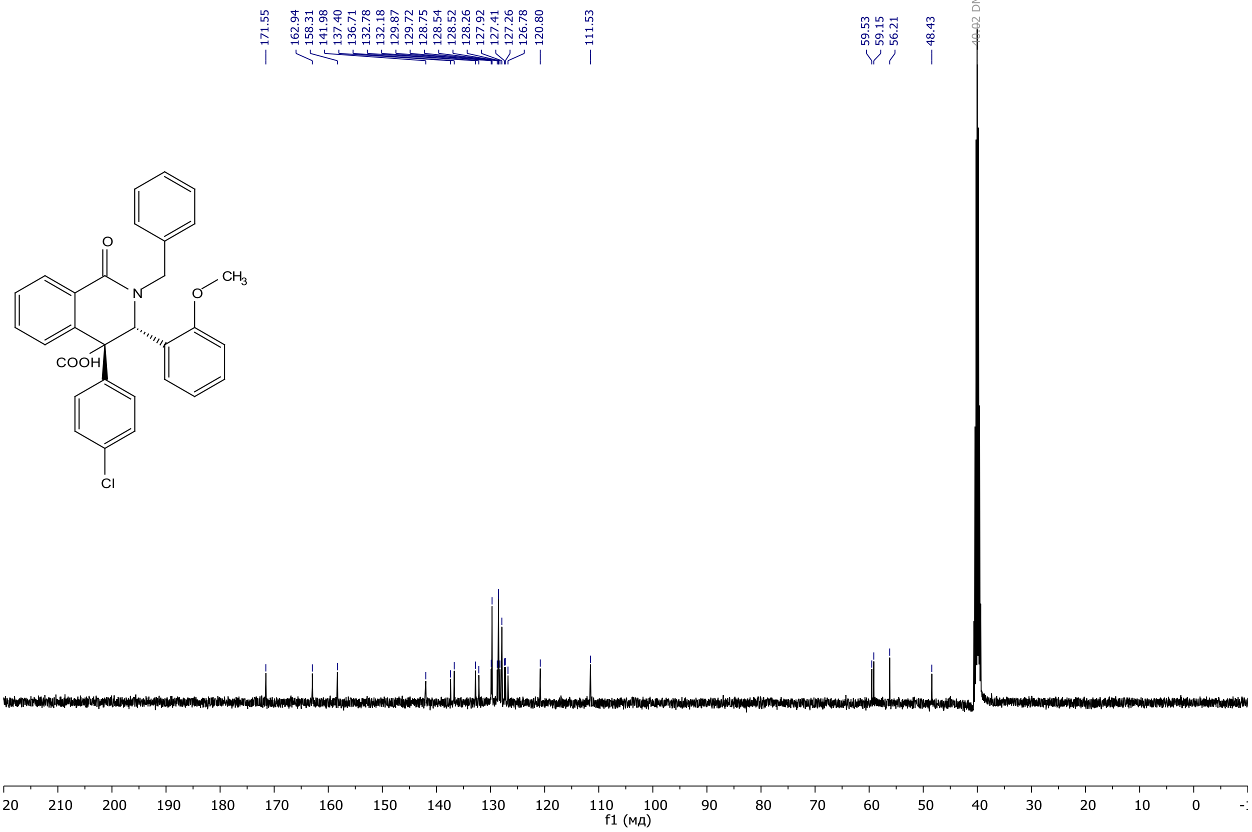

^{1}H and ^{13}C NMR spectra of compound **9h**

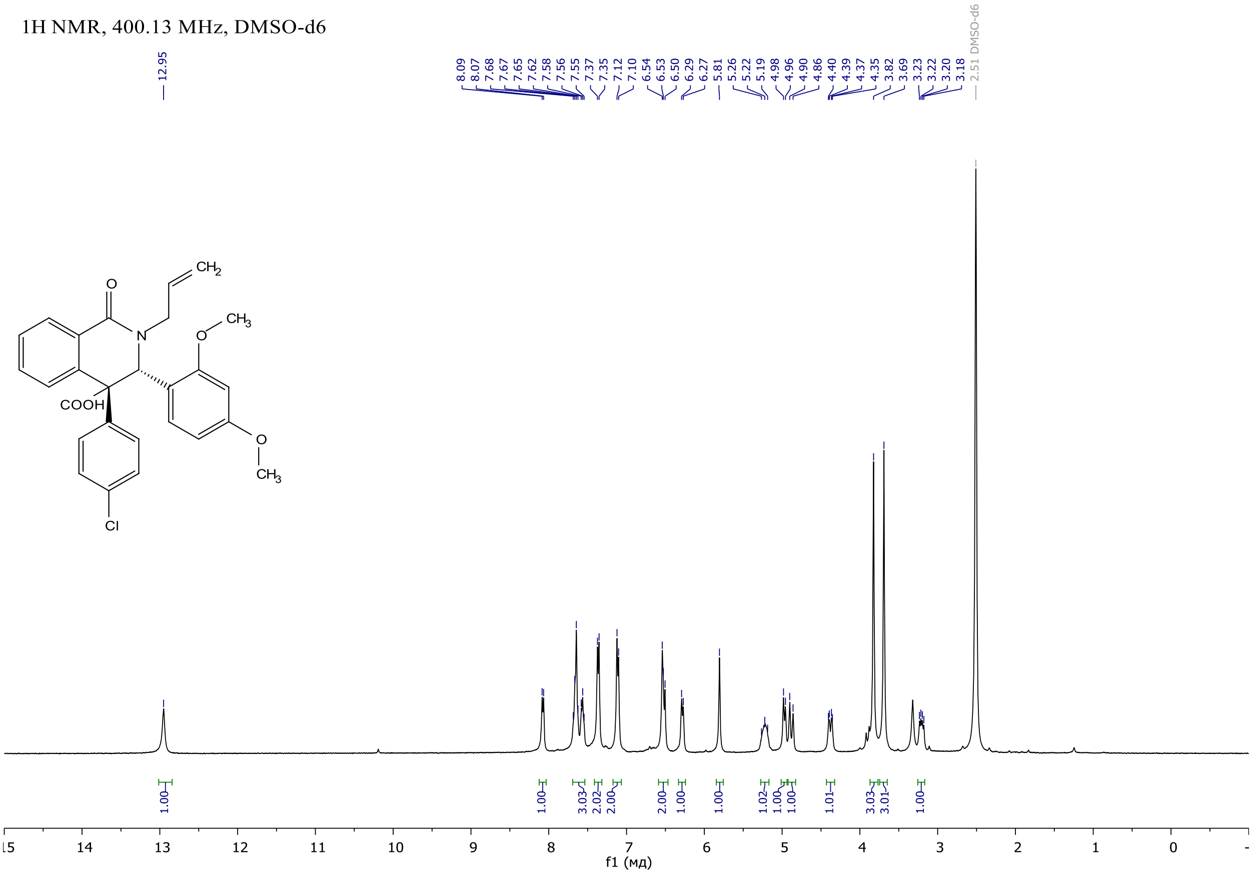

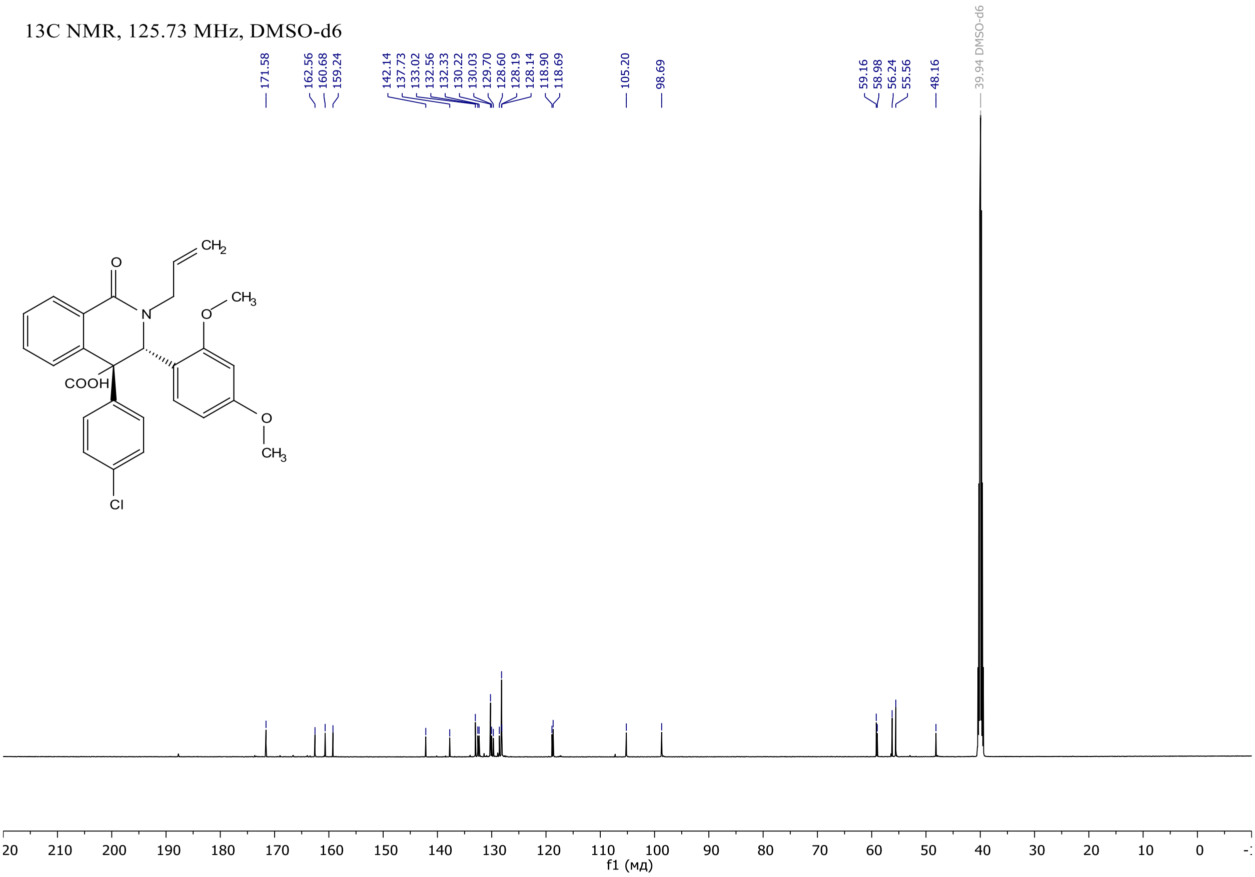

^{1}H and ^{13}C NMR spectra of compound **9i**

1H NMR, 400.13 MHz, DMSO-d6

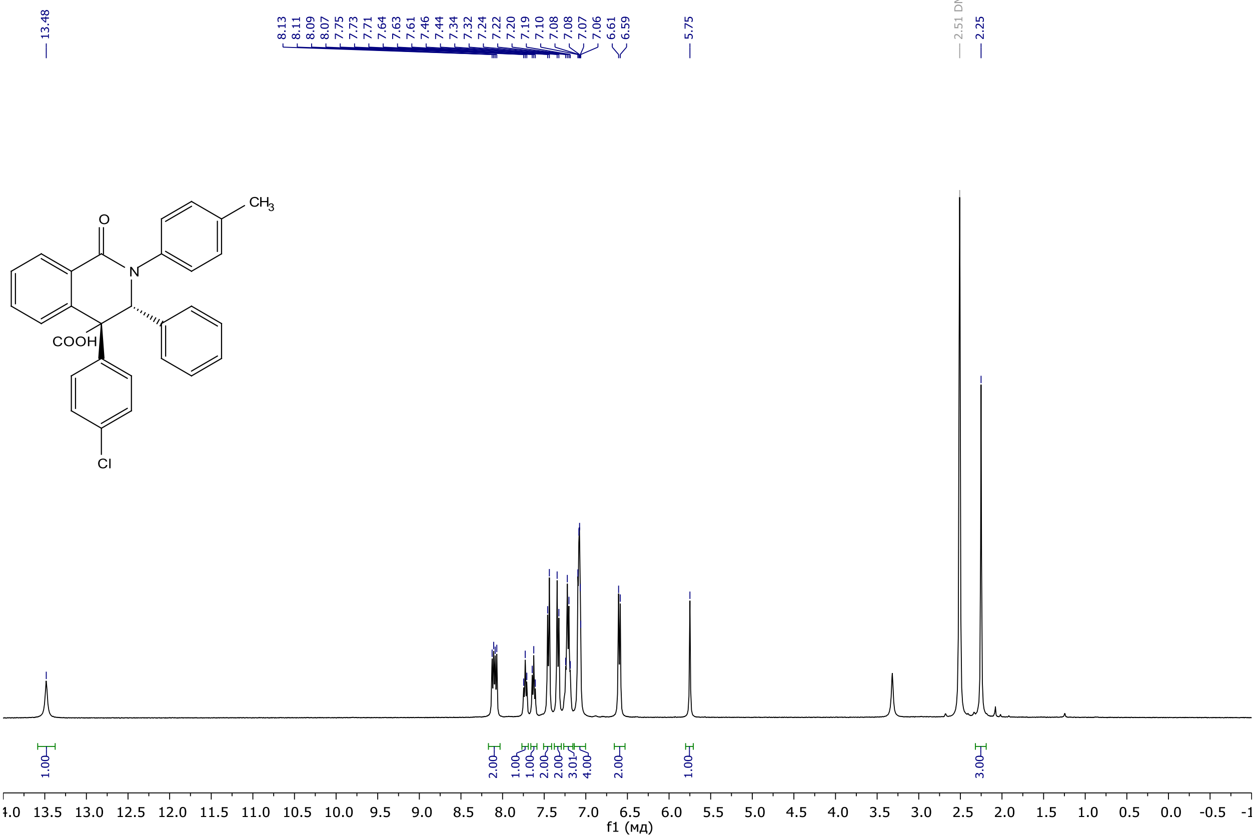

13C NMR, 125.73 MHz, DMSO-d6

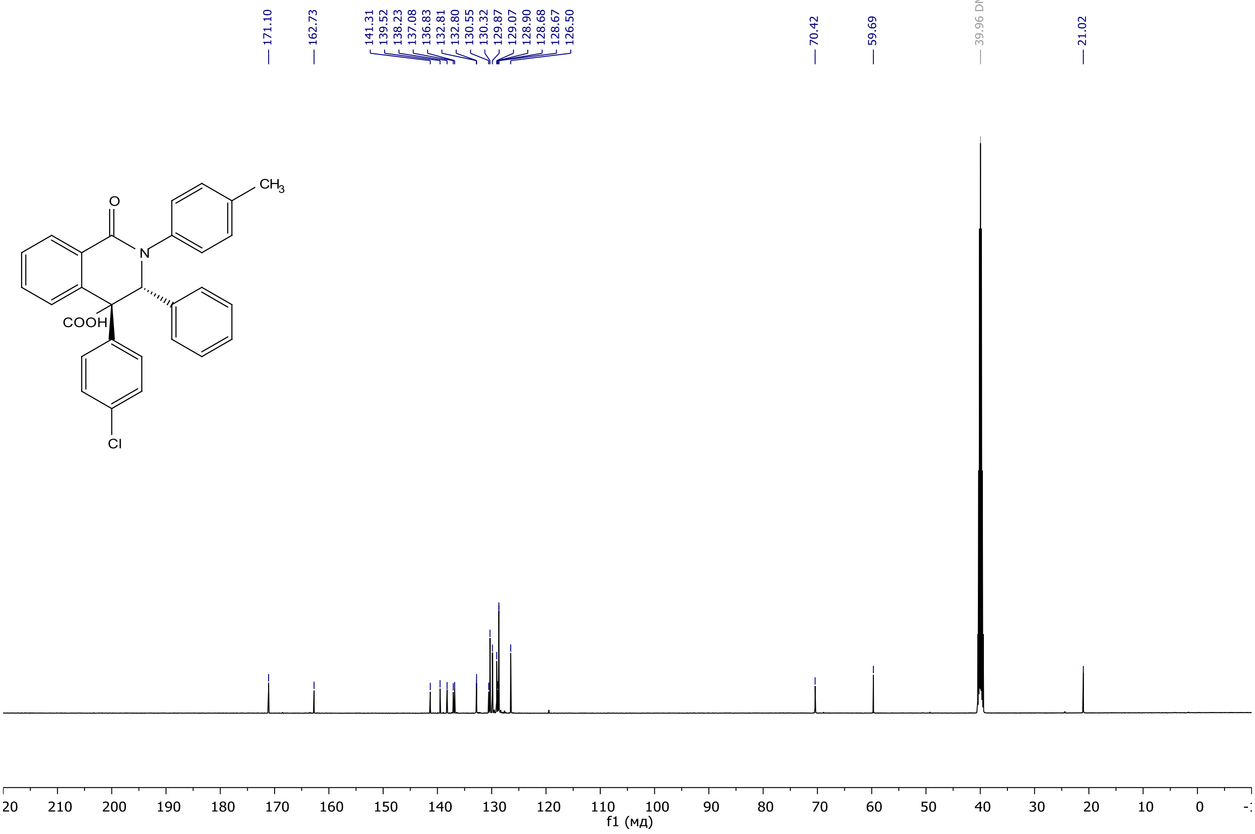

^{1}H, ^{13}C and ^{19}F NMR spectra of compound **9j**

1H NMR, 400.13 MHz, DMSO-d6

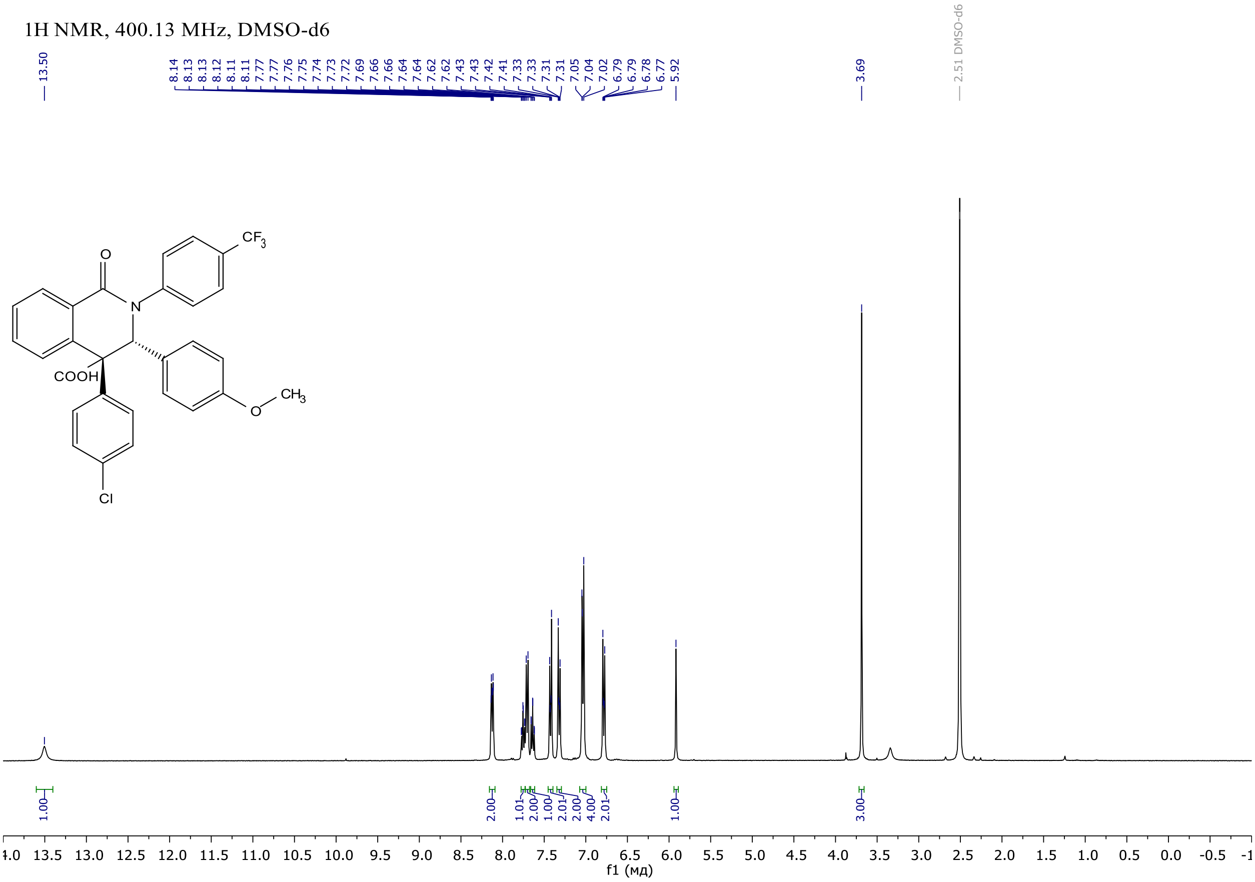

13C NMR, 100.61 MHz, DMSO-d6

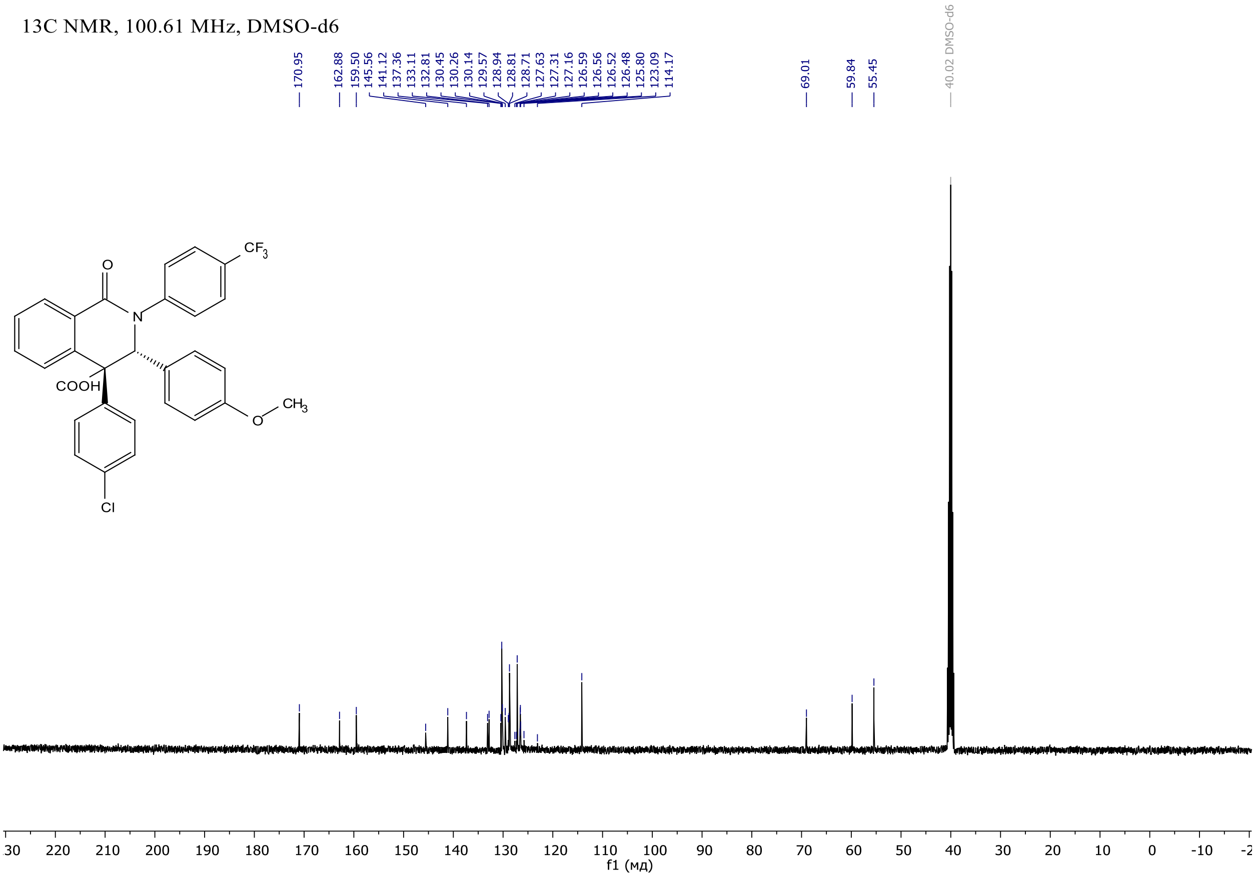

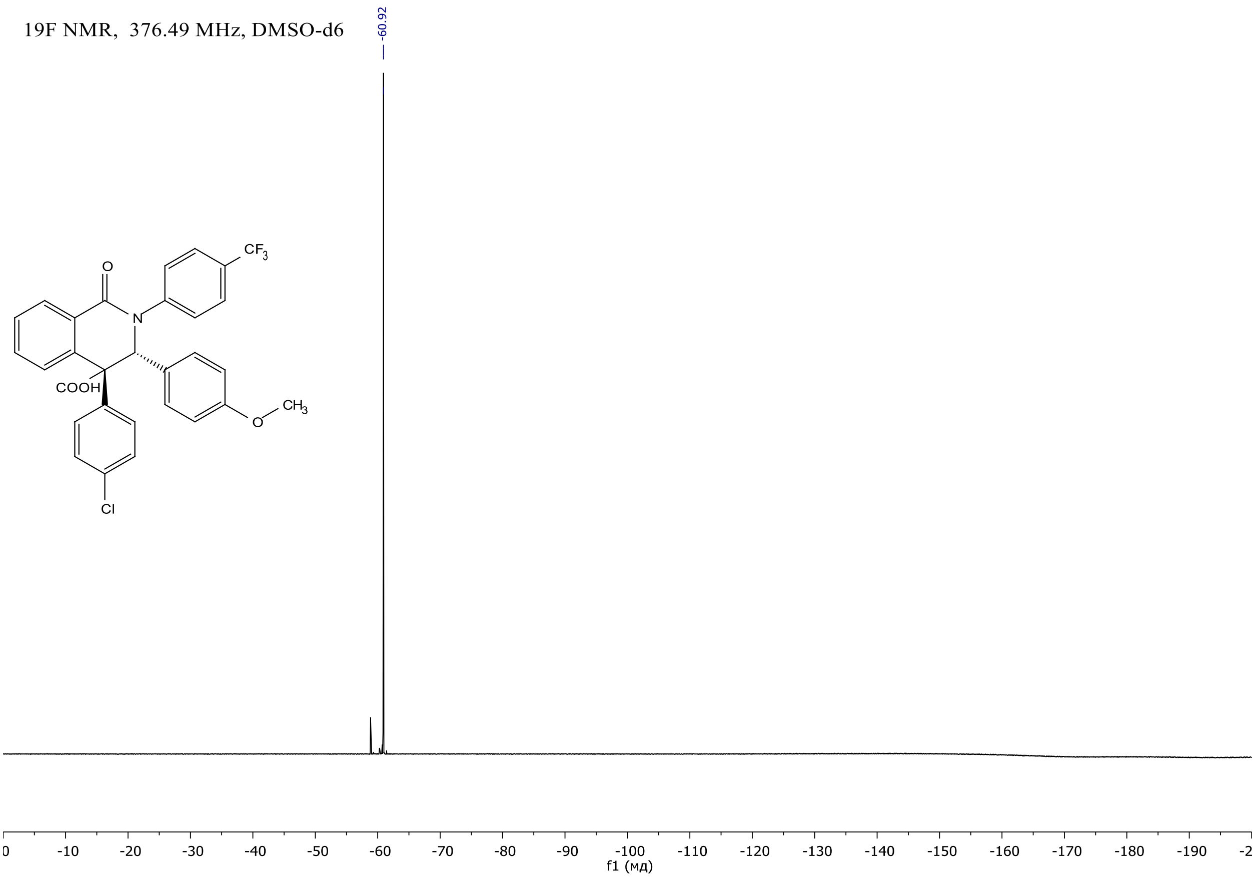
19F NMR, 376.49 MHz, DMSO-d6
-60.92
CF3
O
N
COOH
CH3
O
Cl
0 -10 -20 -30 -40 -50 -60 -70 -80 -90 -100 -110 -120 -130 -140 -150 -160 -170 -180 -190 -2
f1 (мд)

^{1}H and ^{13}C NMR spectra of compound **9k**

1H NMR, 400.13 MHz, DMSO-d6

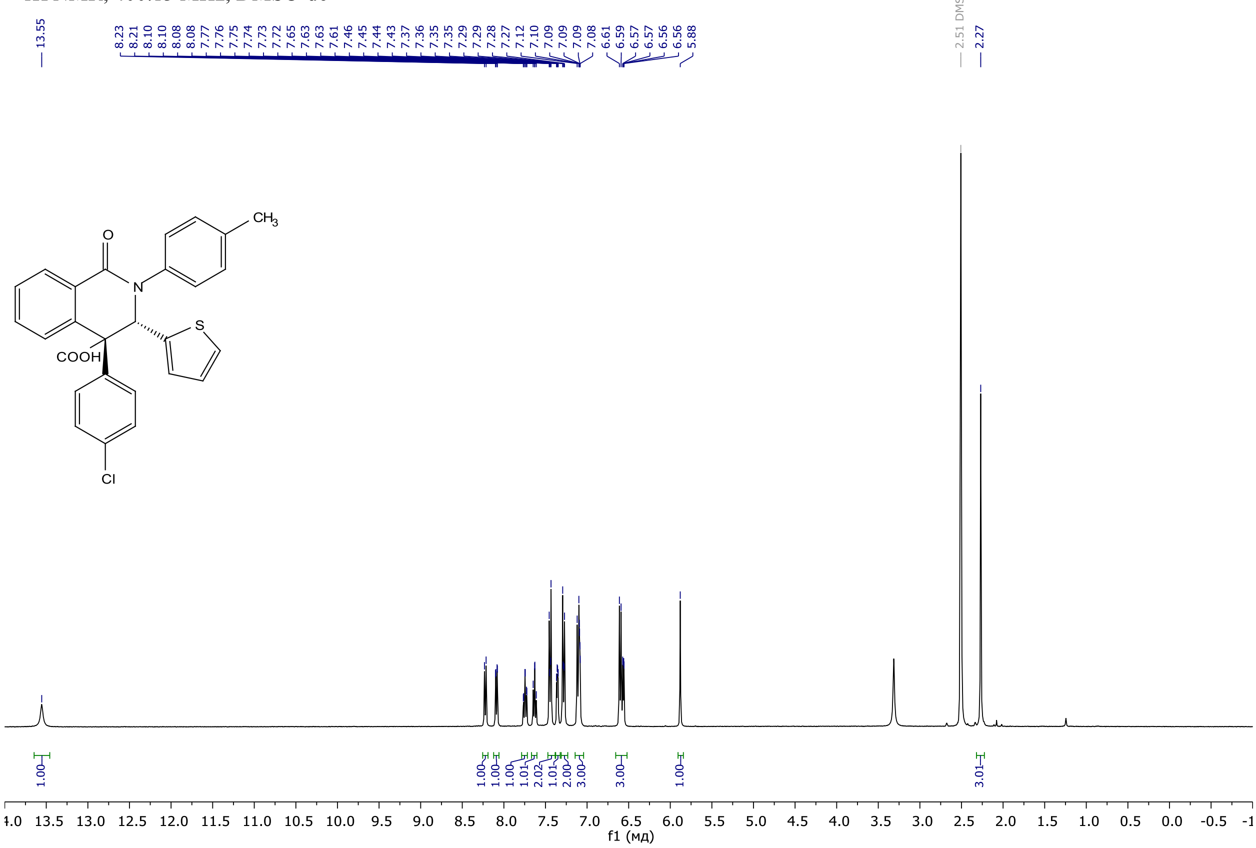

13C NMR, 100.61 MHz, DMSO-d6

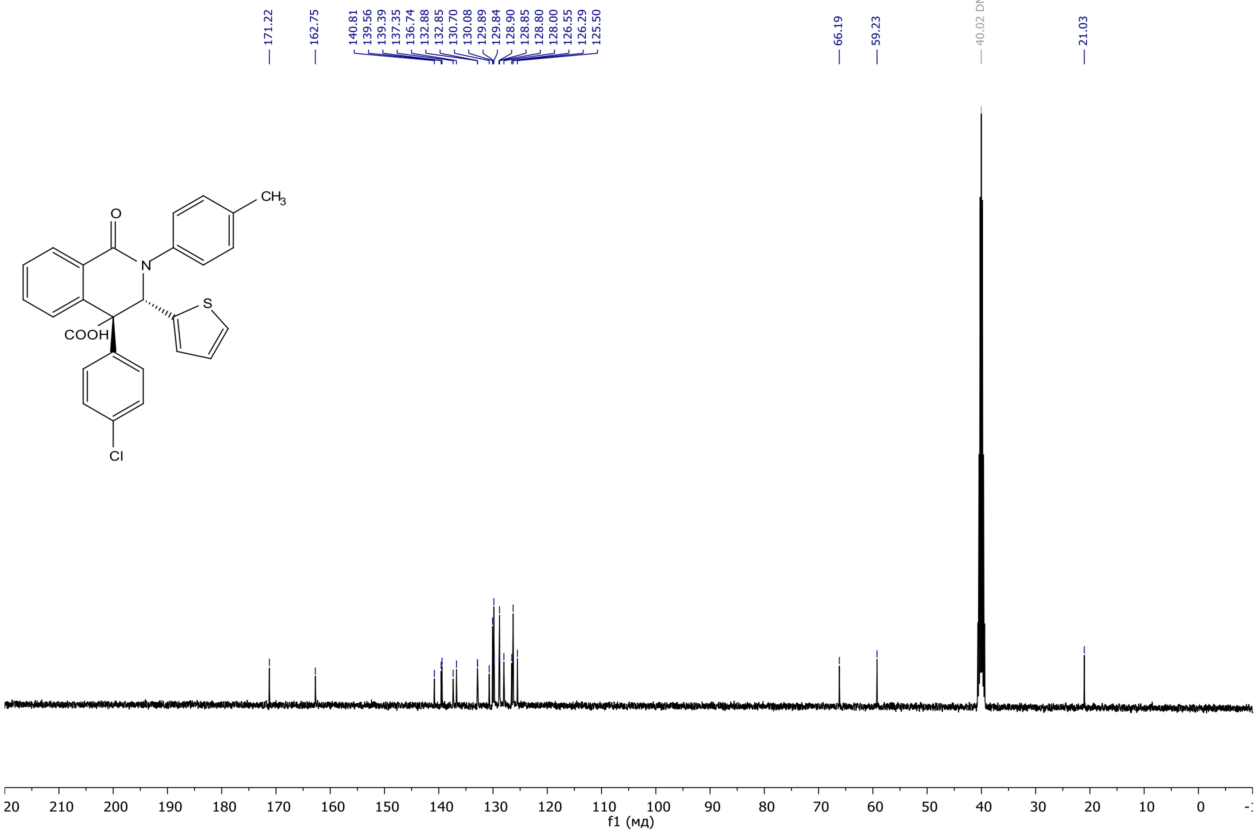

^{1}H, ^{13}C and ^{19}F NMR spectra of compound **9l**

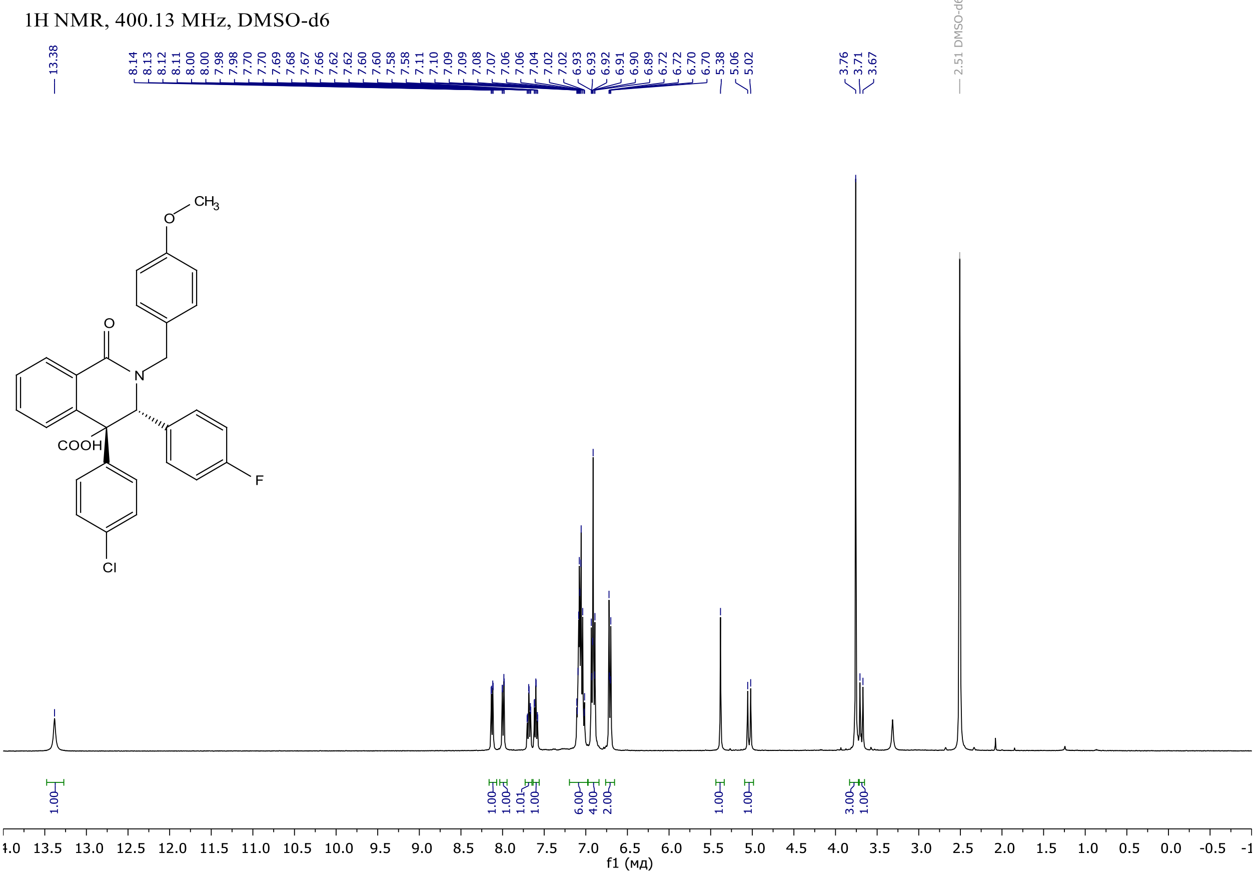

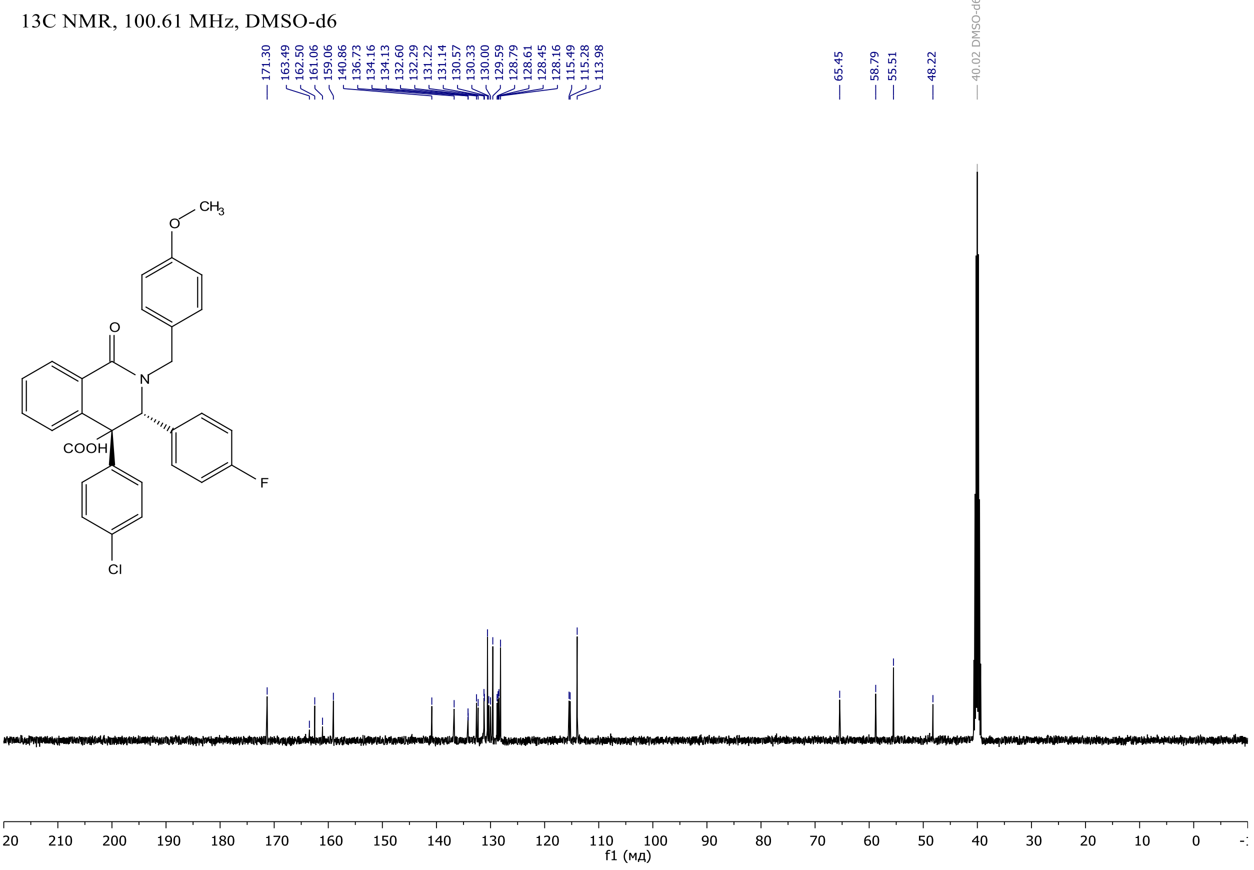

19F NMR, 376.49 MHz, DMSO-d6

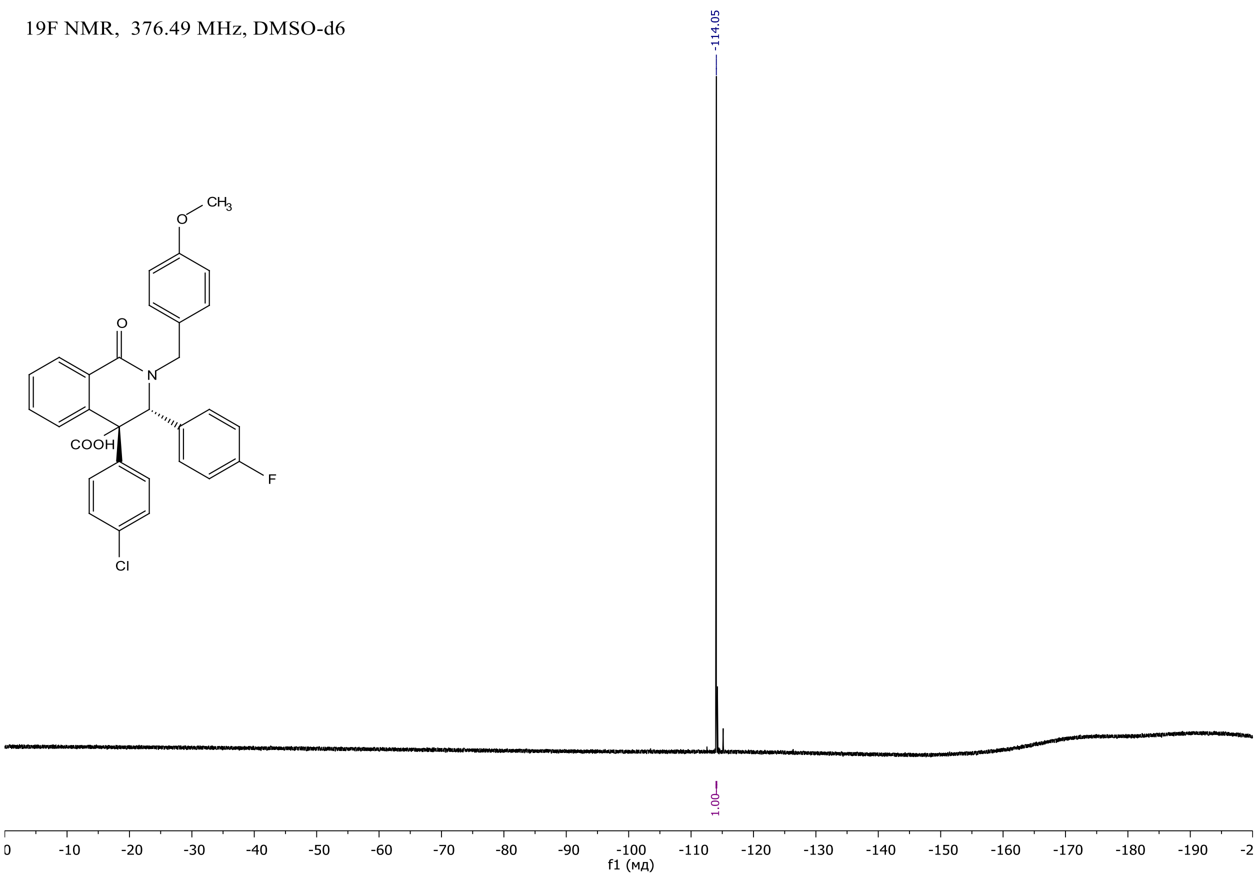

^{1}H and ^{13}C NMR spectra of compound **9m**

1H NMR, 400.13 MHz, DMSO-d6

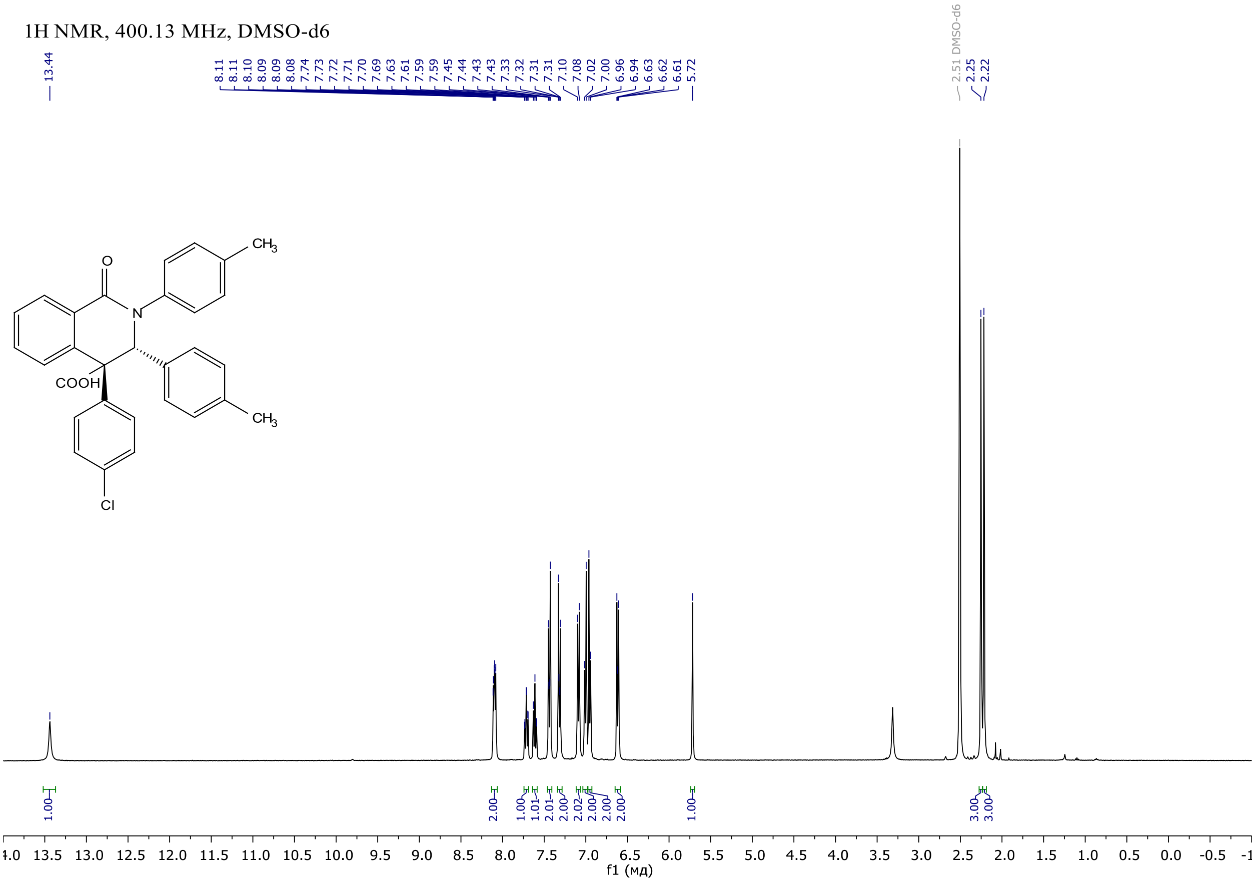

13C NMR, 100.61 MHz, DMSO-d6

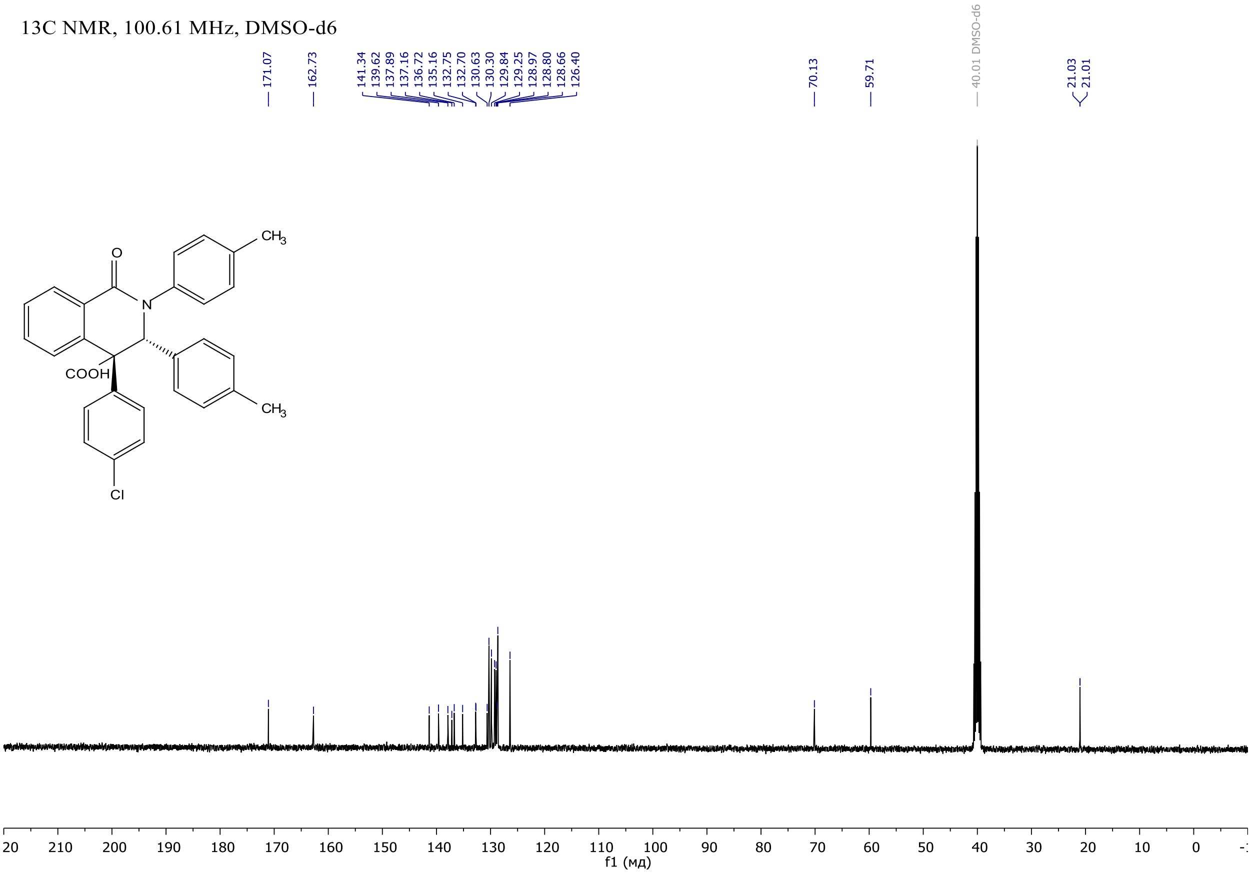

^{1}H and ^{13}C NMR spectra of compound **9n**

1H NMR, 400.13 MHz, DMSO-d6

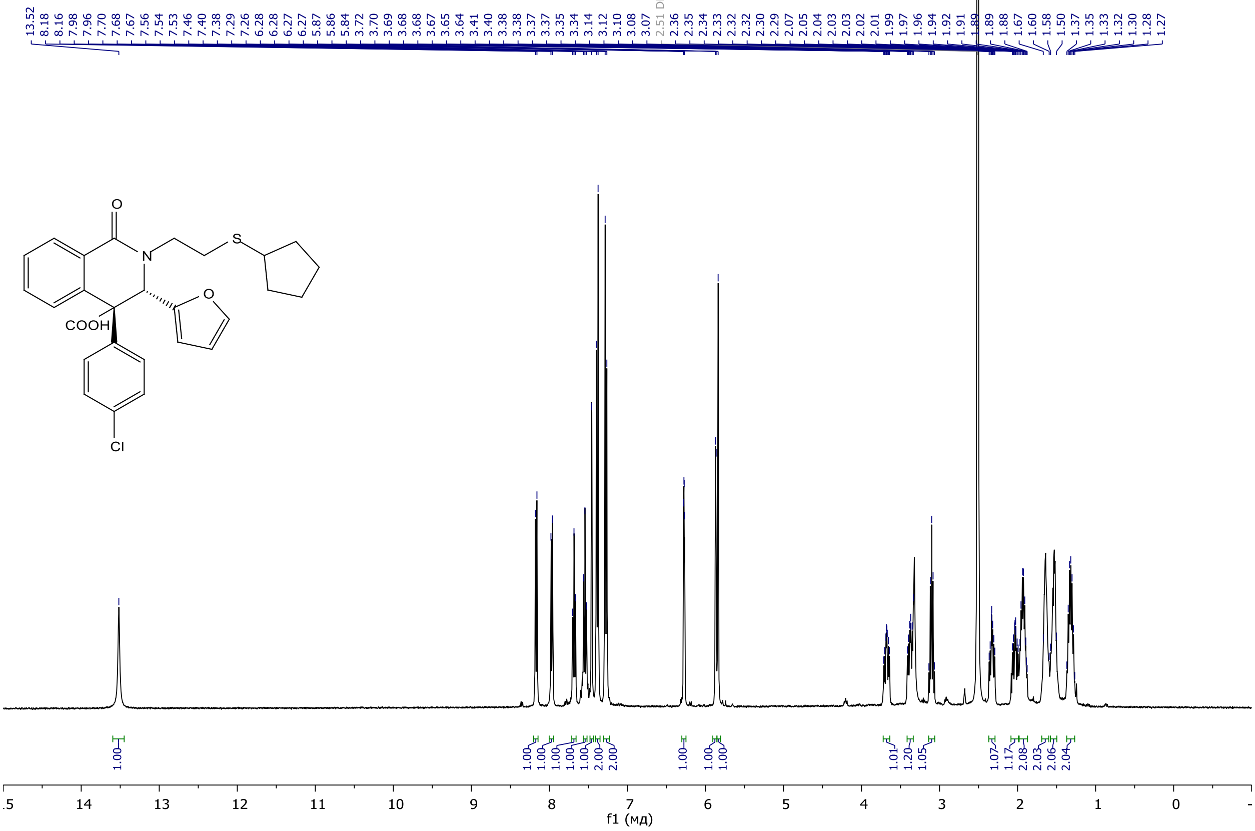

13C NMR, 100.61 MHz, DMSO-d6

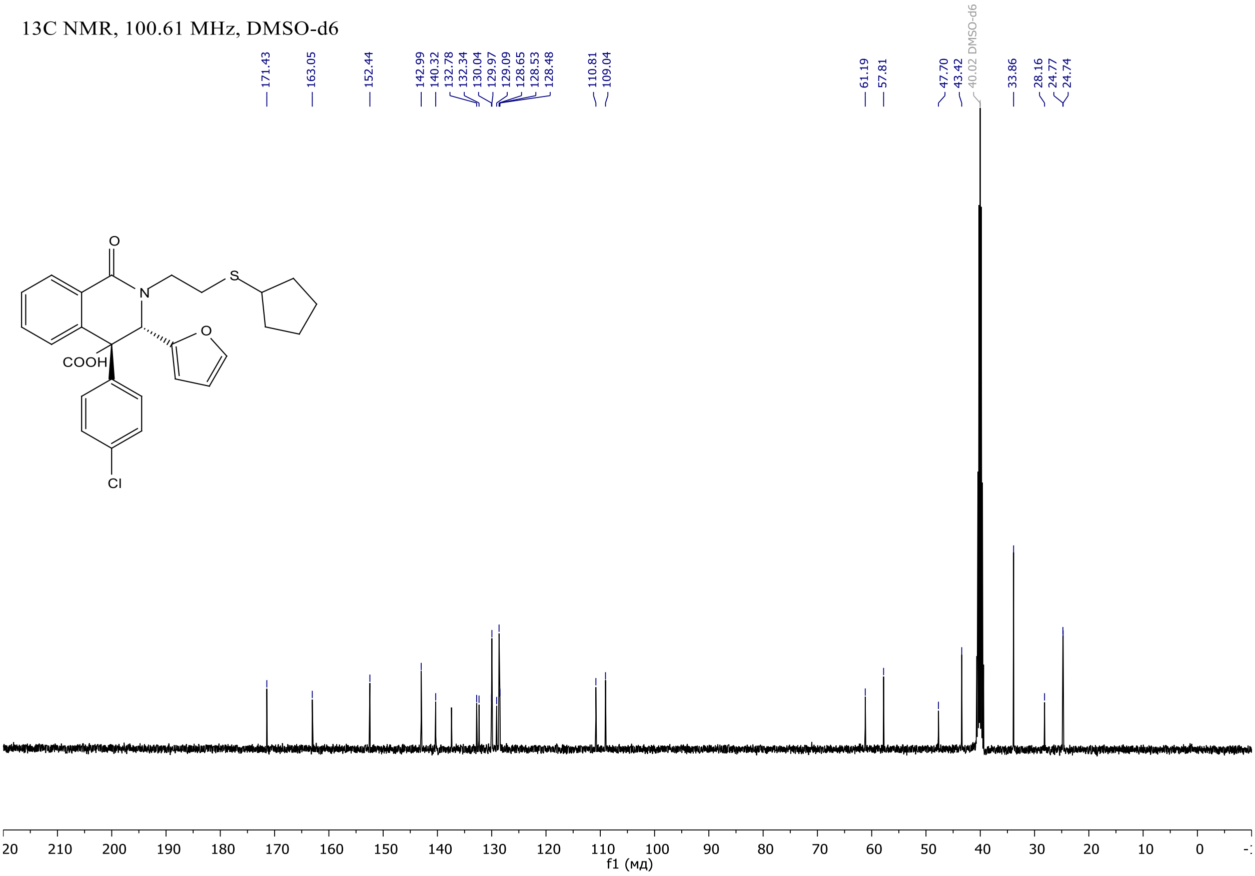

^{1}H and ^{13}C NMR spectra of compound **9o**

1H NMR, 400.13 MHz, DMSO-d6

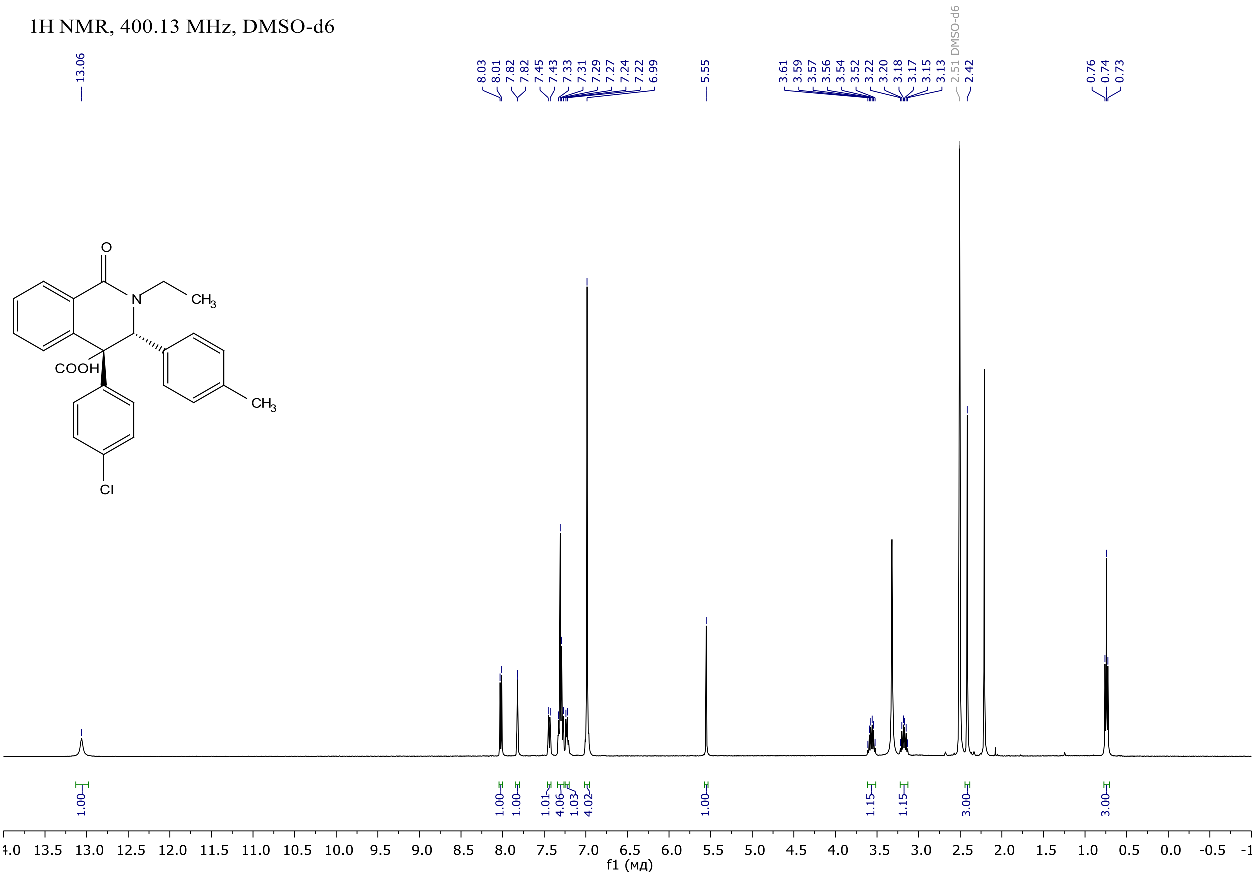

13C NMR, 100.61 MHz, DMSO-d6

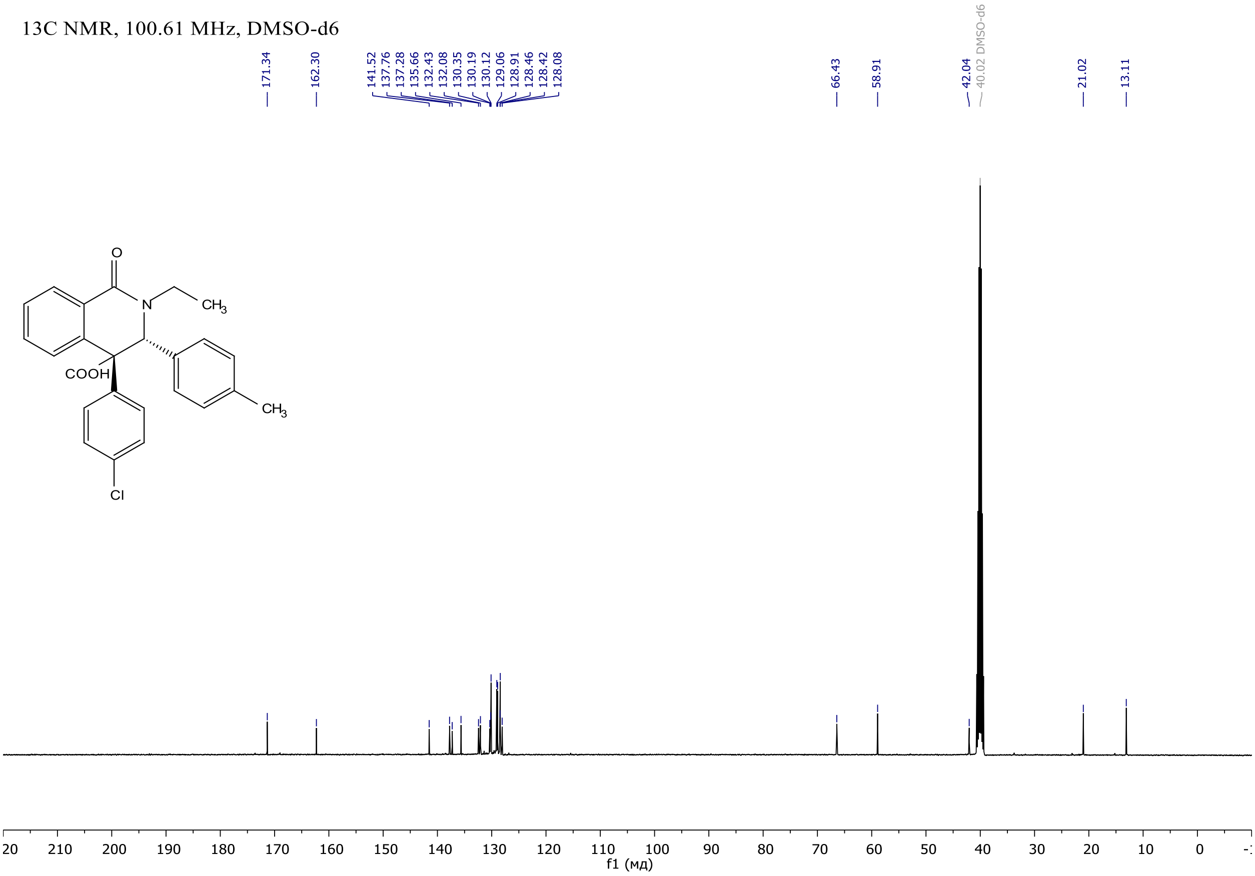

^{1}H and ^{13}C NMR spectra of compound **9p**

1H NMR, 400.13 MHz, DMSO-d6

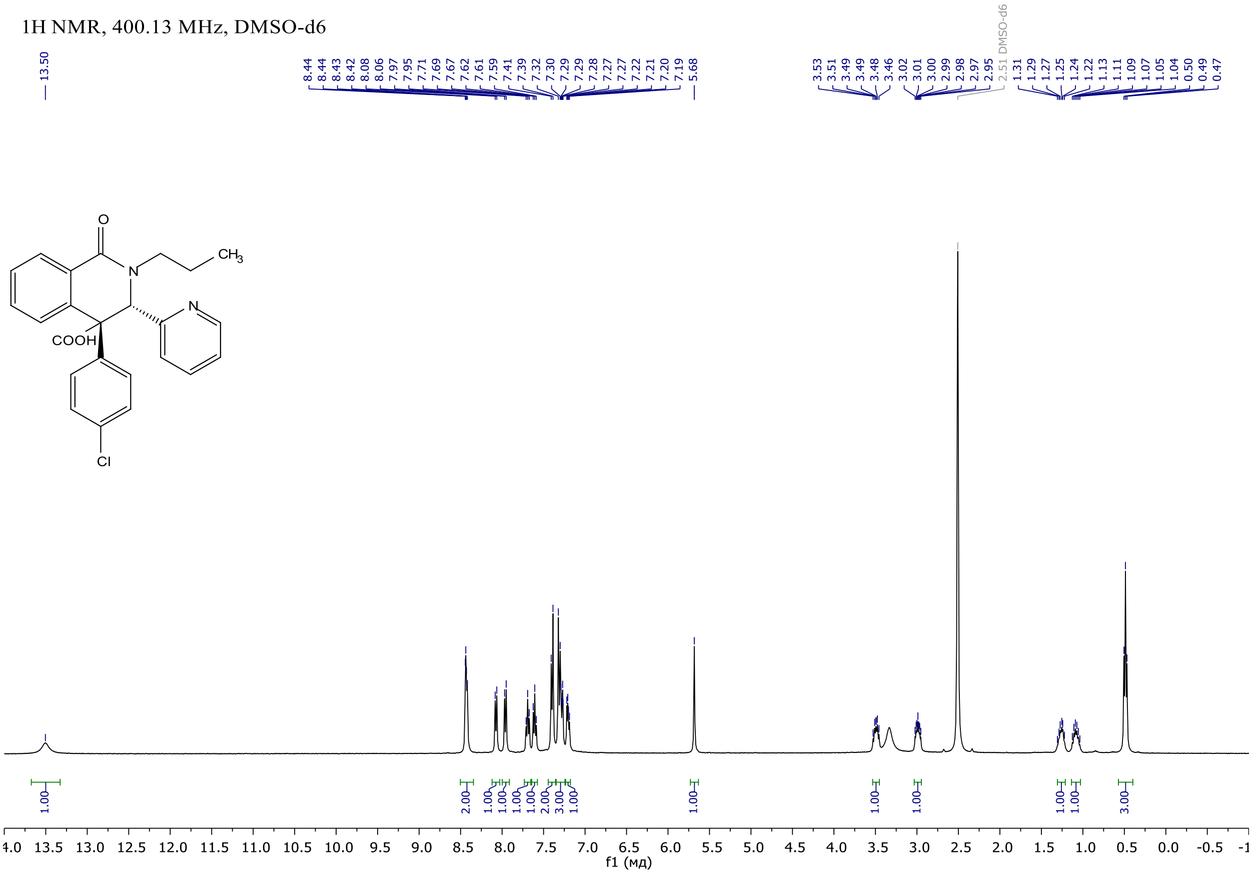

13C NMR, 100.61 MHz, DMSO-d6

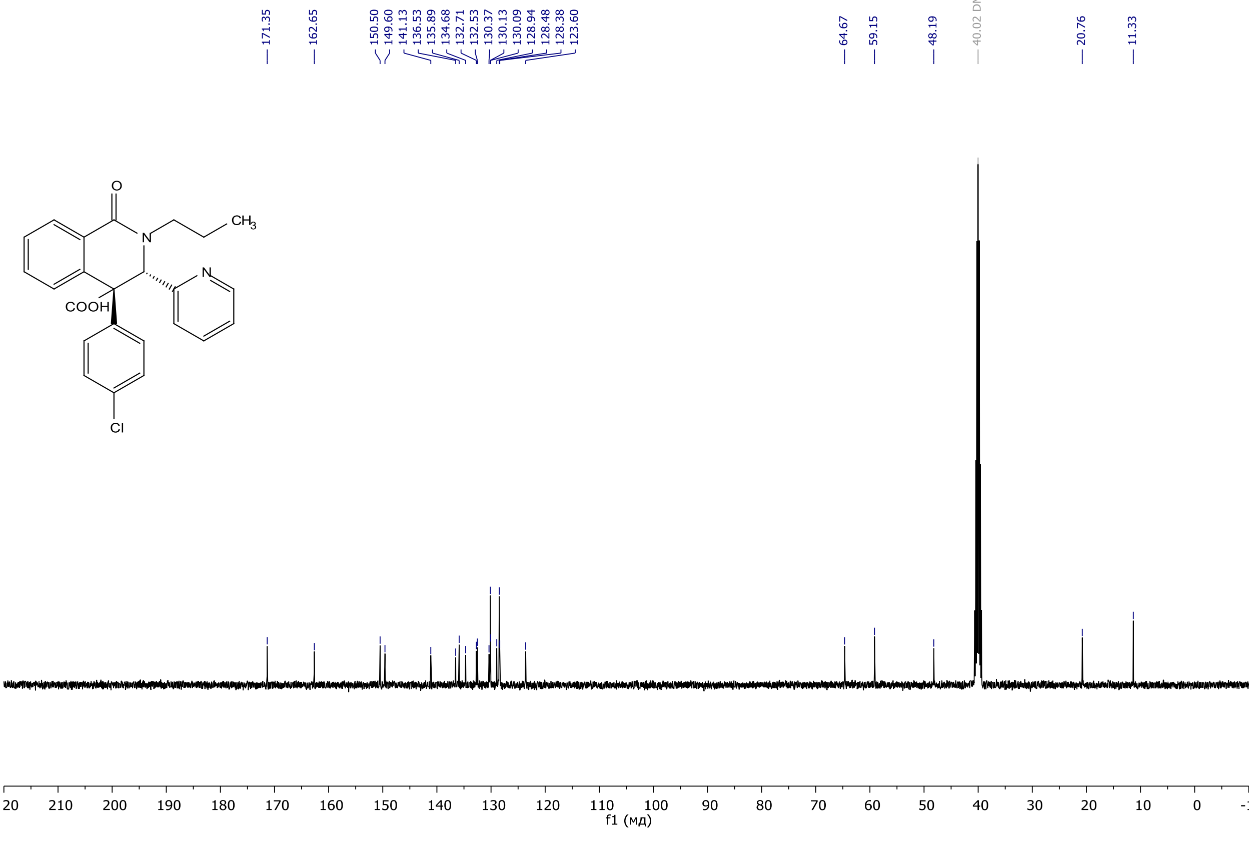

^{1}H, ^{13}C and ^{19}F NMR spectra of compound **9q**

1H NMR, 400.13 MHz, DMSO-d6

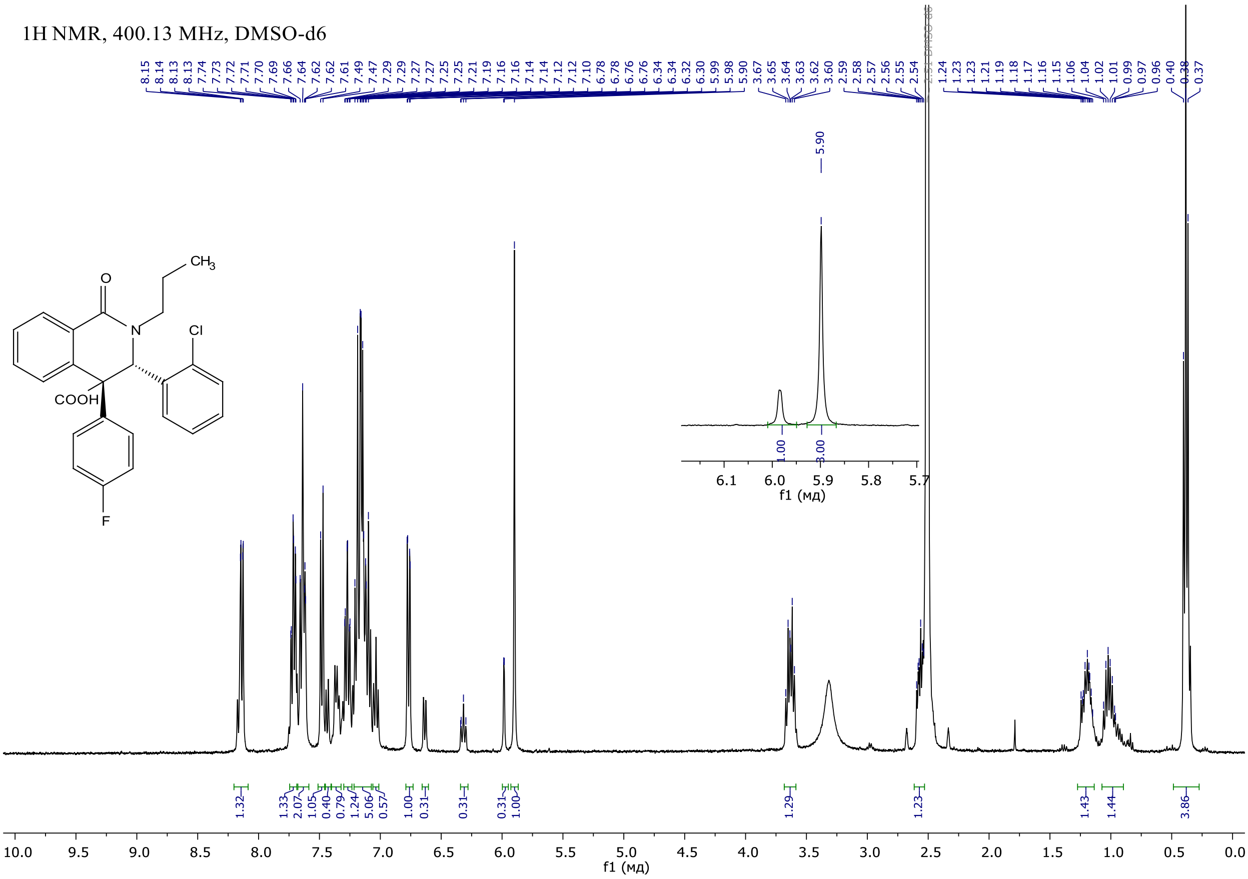

13C NMR, 100.61 MHz, DMSO-d6

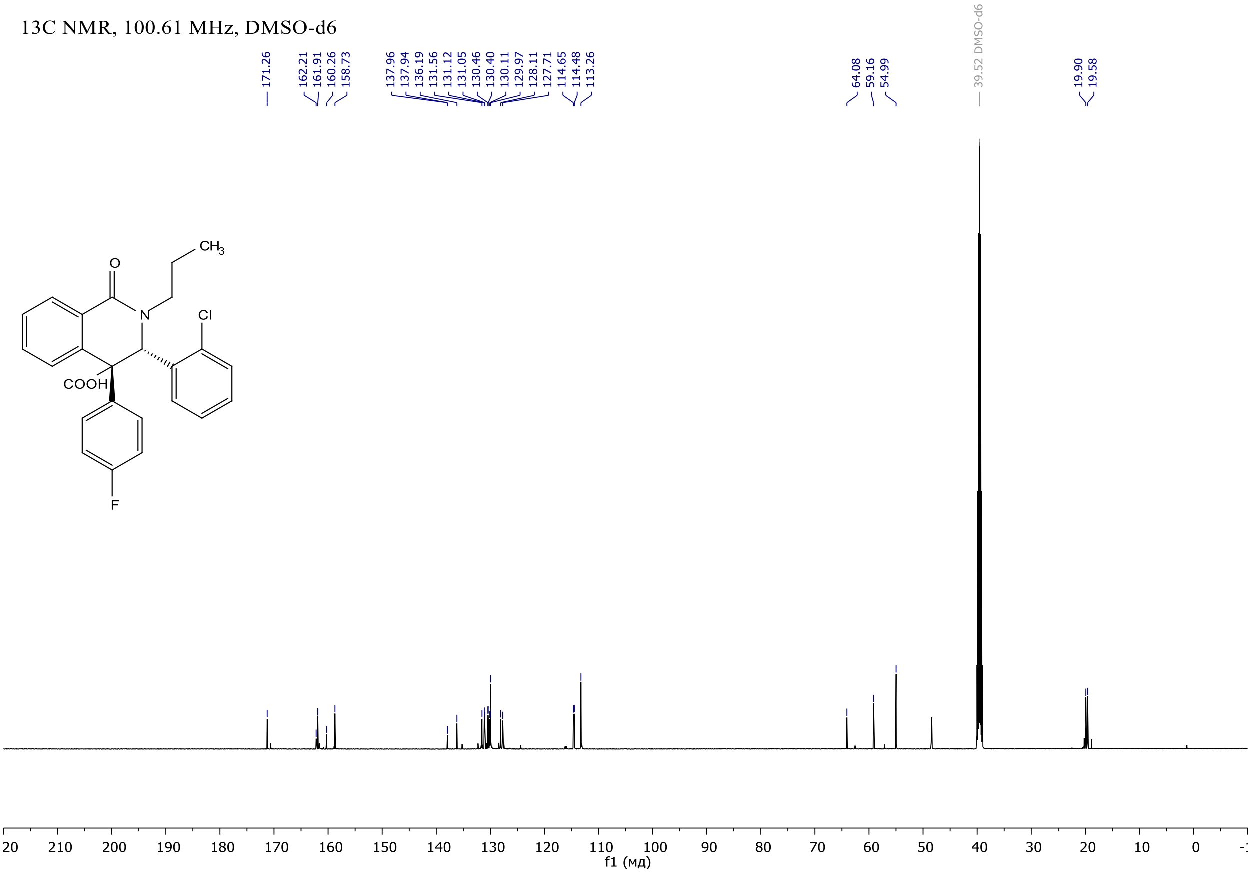

19F NMR, 376.49 MHz, DMSO-d6

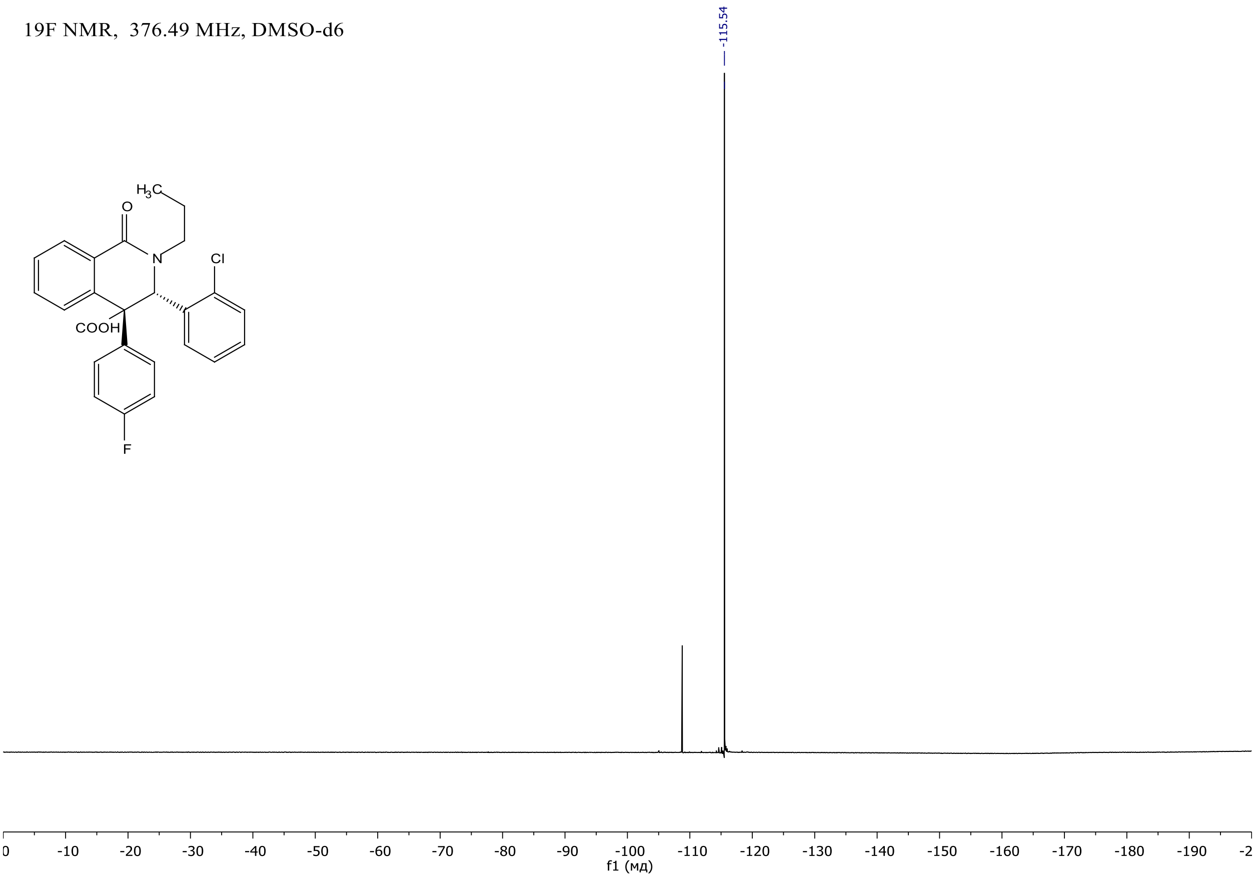

^{1}H, ^{13}C and ^{19}F NMR spectra of compound **9r**

1H NMR, 400.13 MHz, DMSO-d6

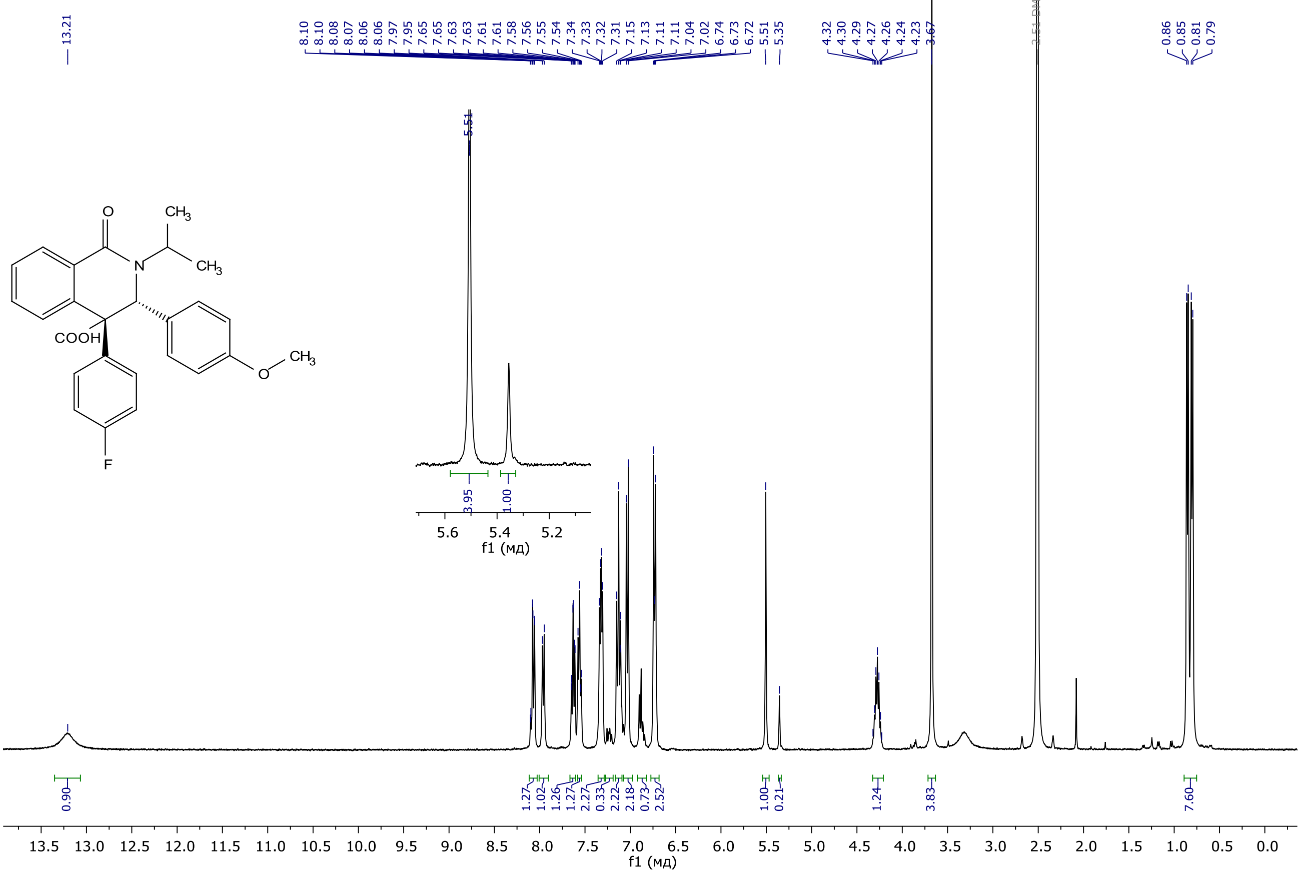

13C NMR, 125.73 MHz, DMSO-d6

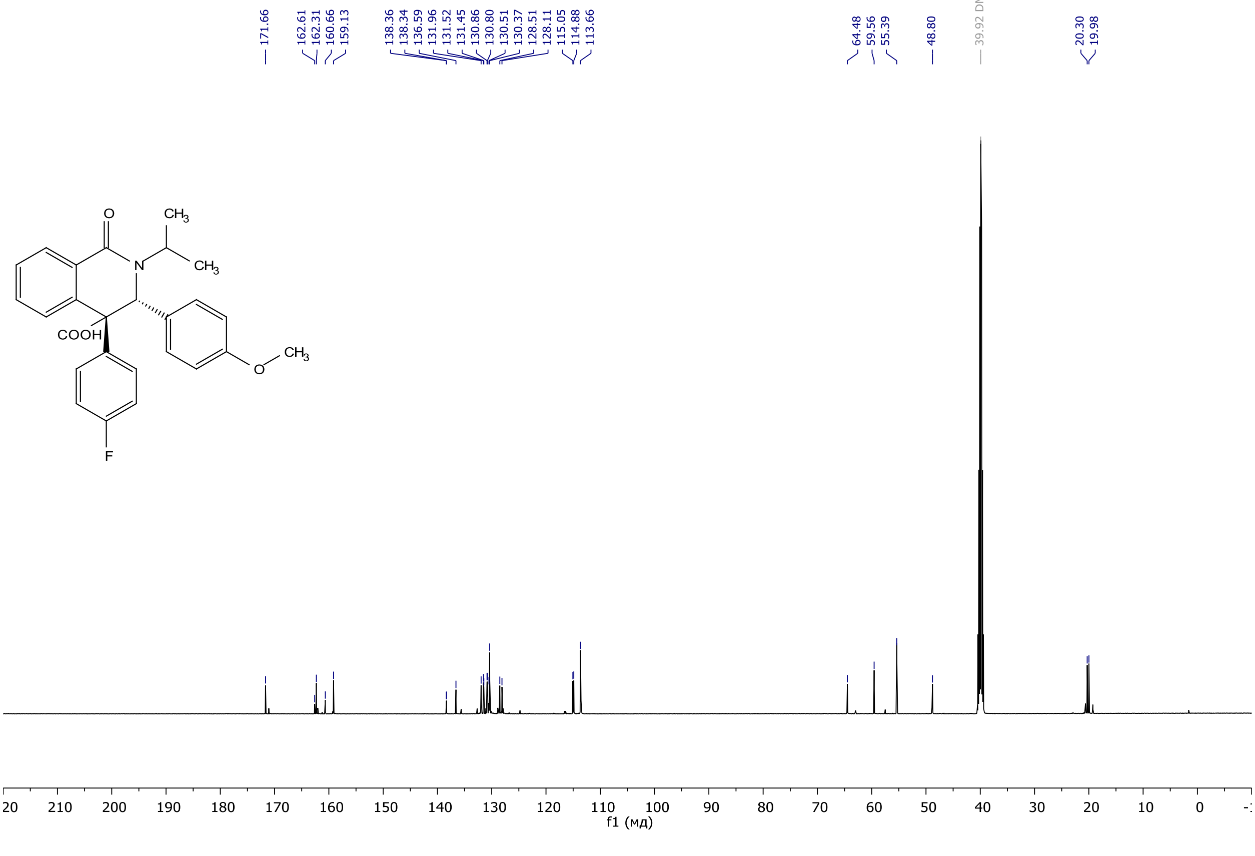

19F NMR, 376.49 MHz, DMSO-d6

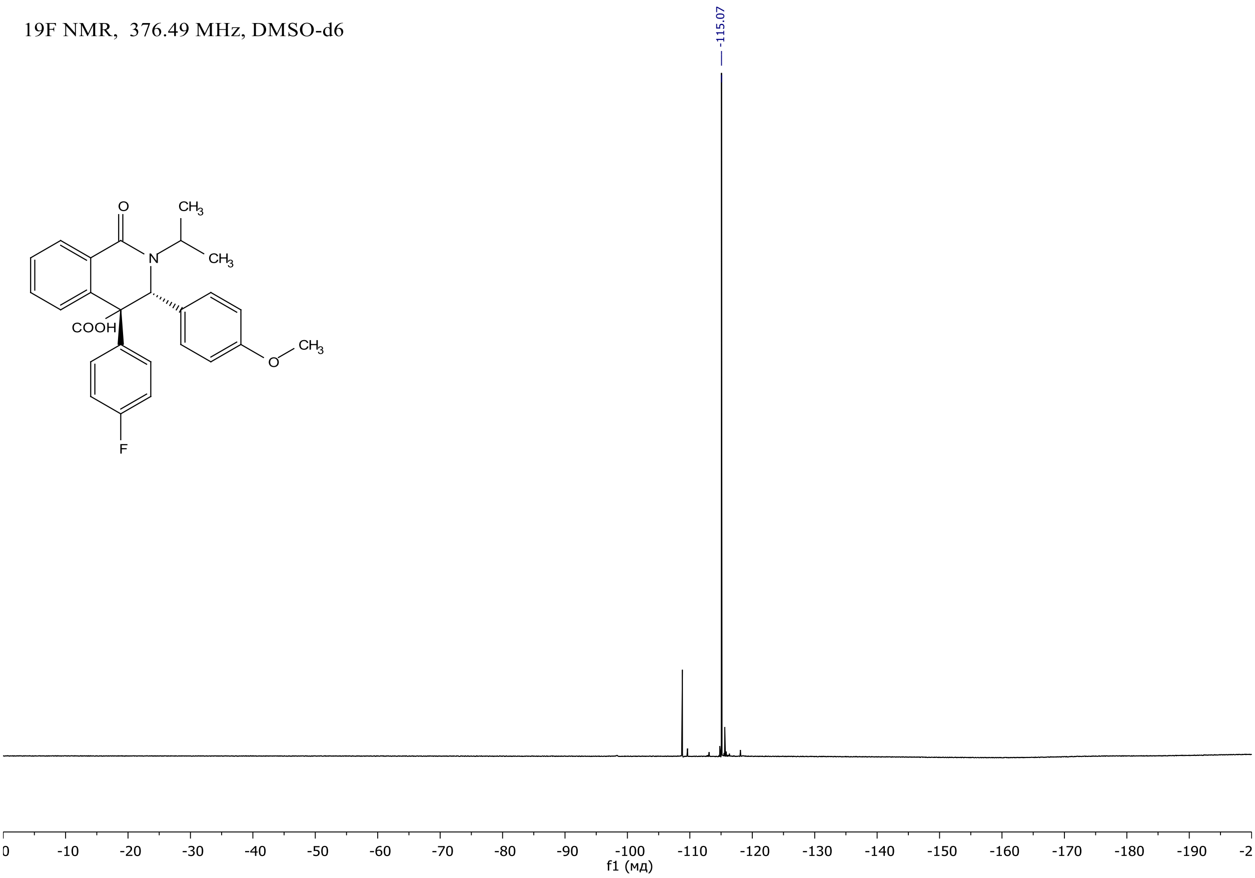

^{1}H, ^{13}C and ^{19}F NMR spectra of compound **9s**

1H NMR, 400.13 MHz, DMSO-d6

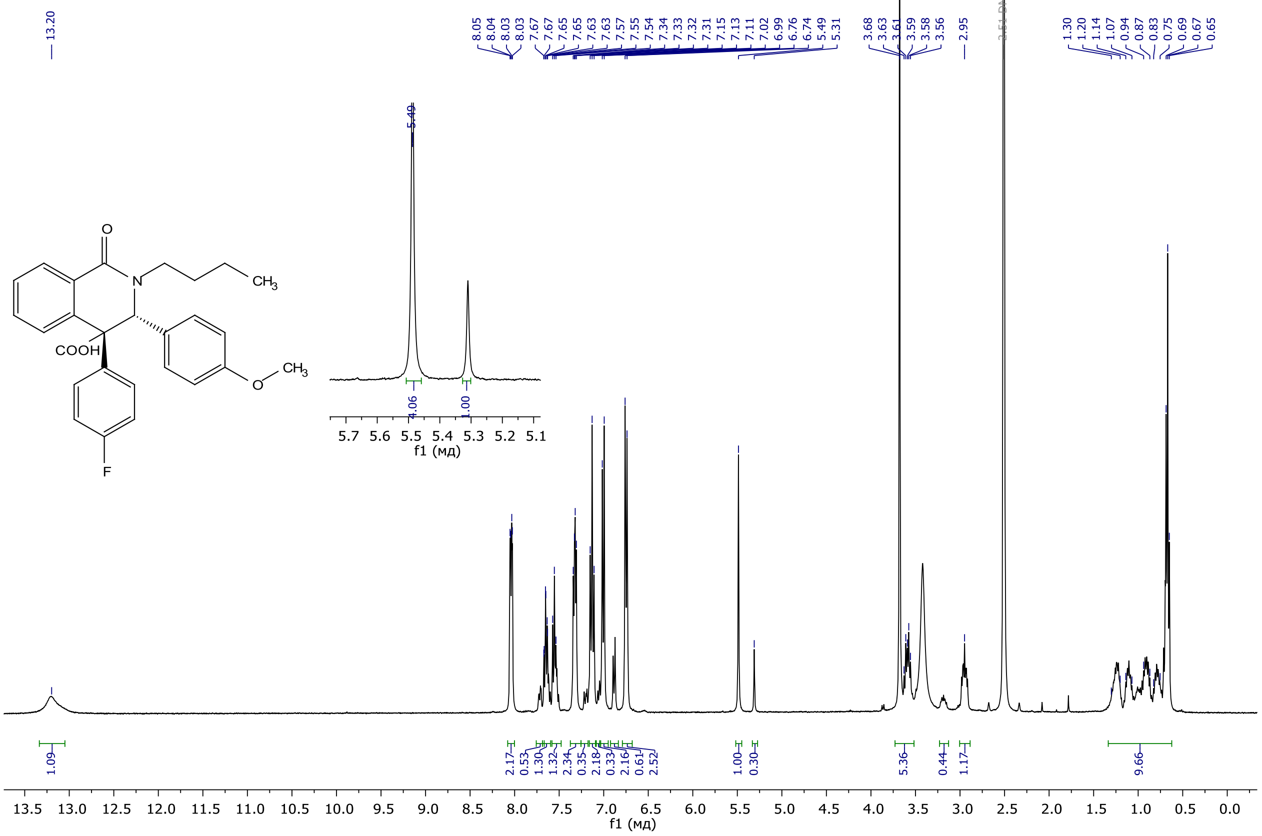

13C NMR, 125.73 MHz, DMSO-d6

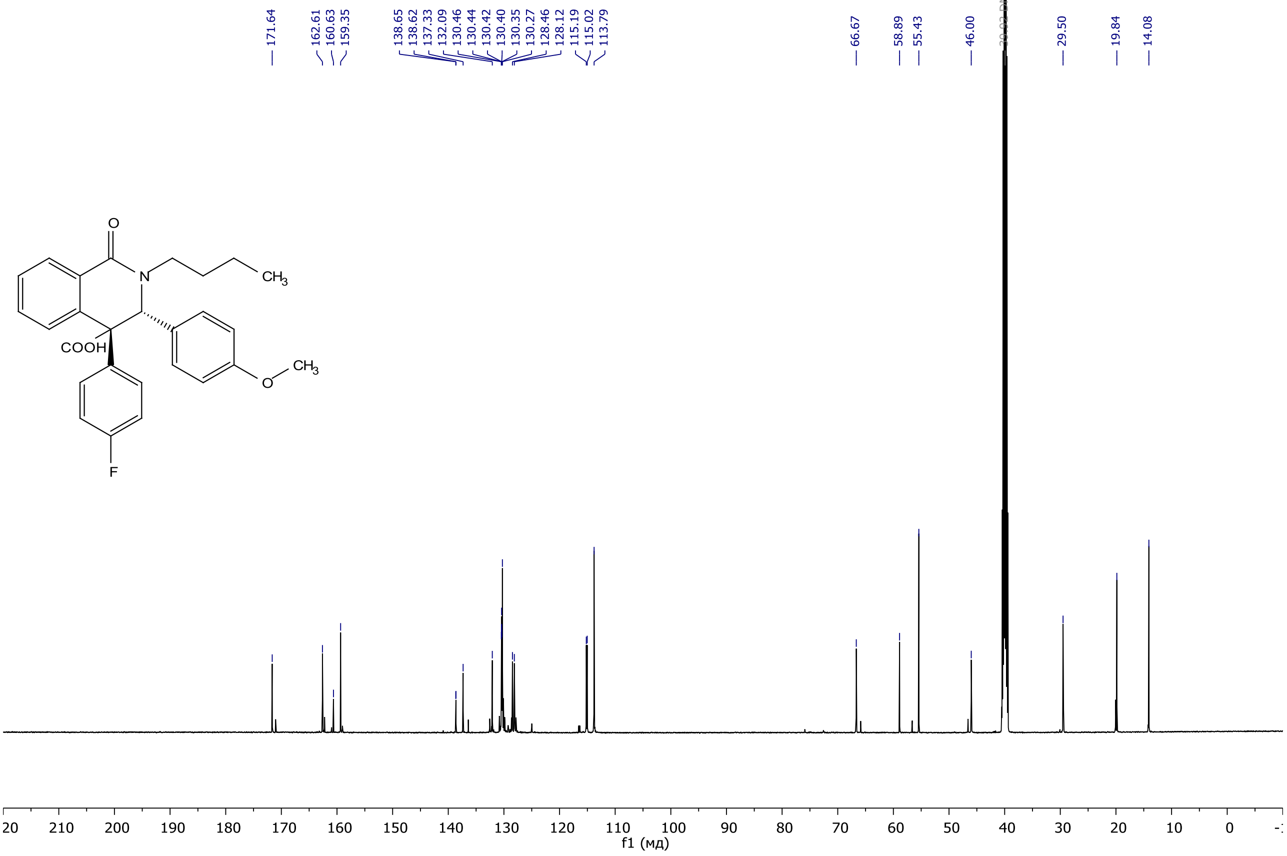

19F NMR, 376.49 MHz, DMSO-d6

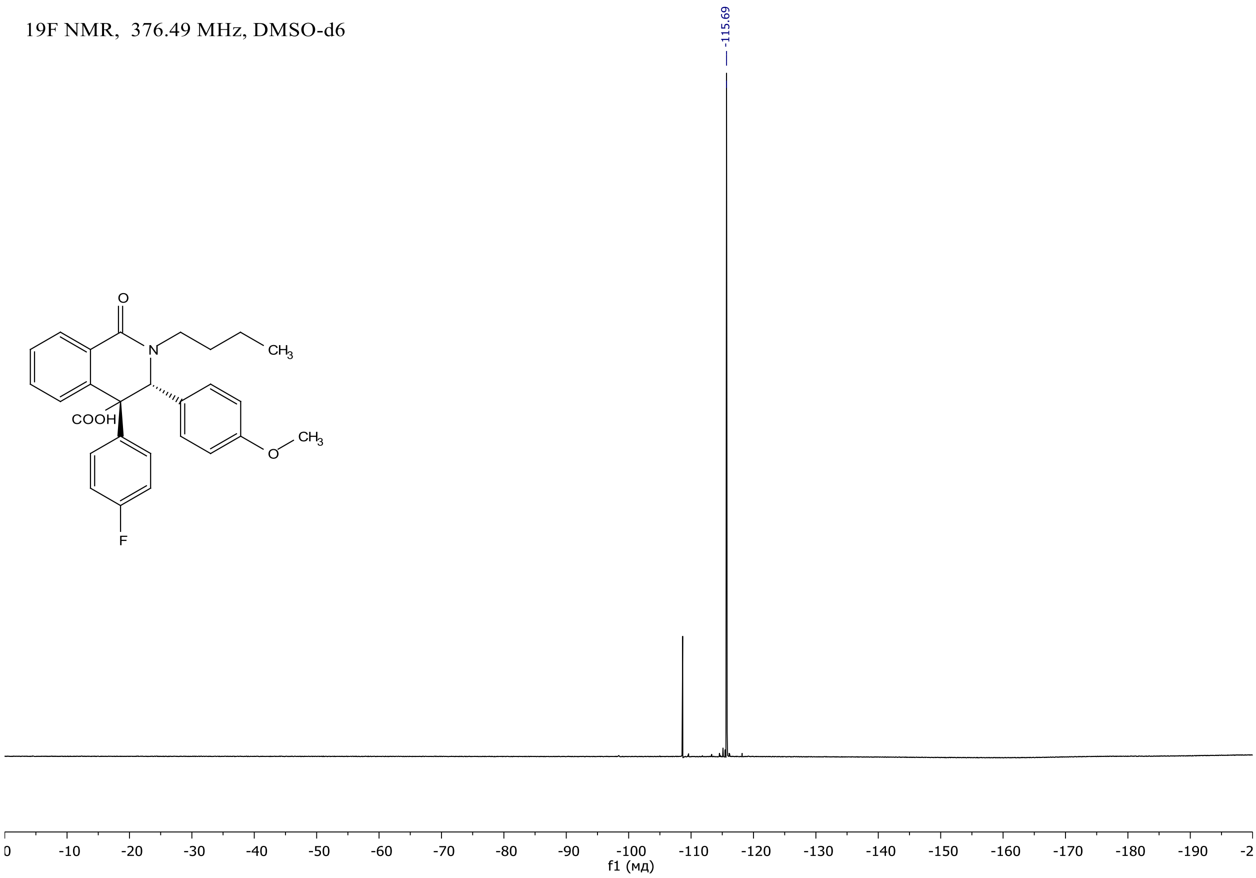

^{1}H, ^{13}C and ^{19}F NMR spectra of compound **9t**

1H NMR, 400.13 MHz, DMSO-d6

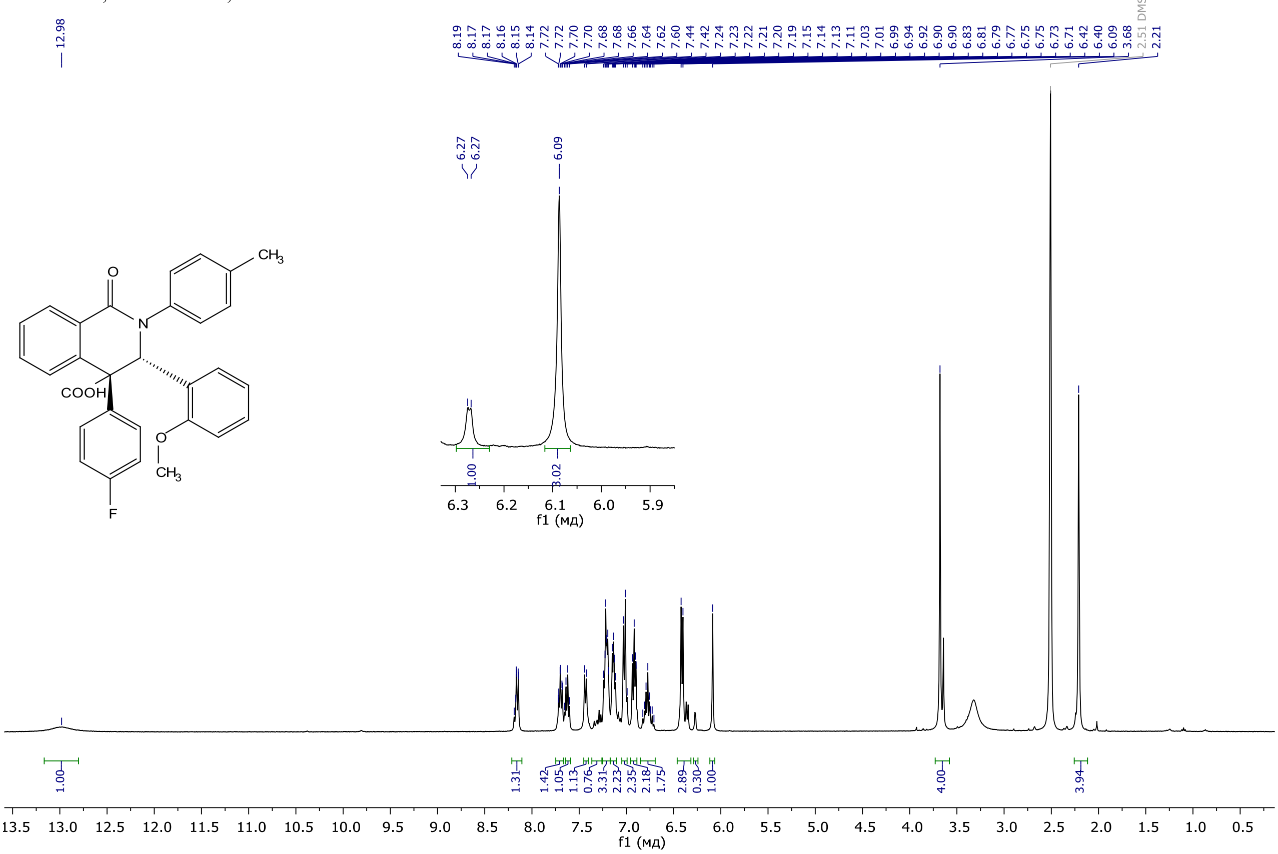

13C NMR, 100.61 MHz, DMSO-d6

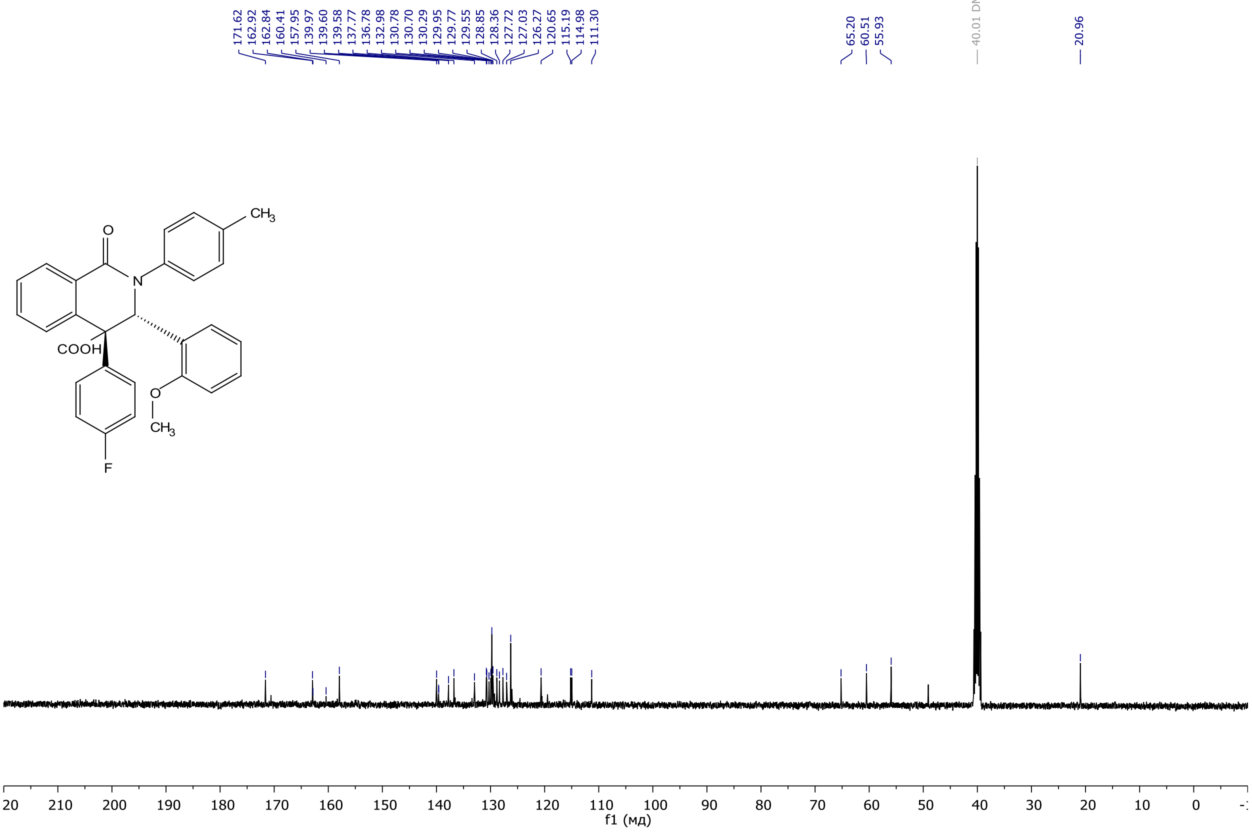

19F NMR, 376.49 MHz, DMSO-d6

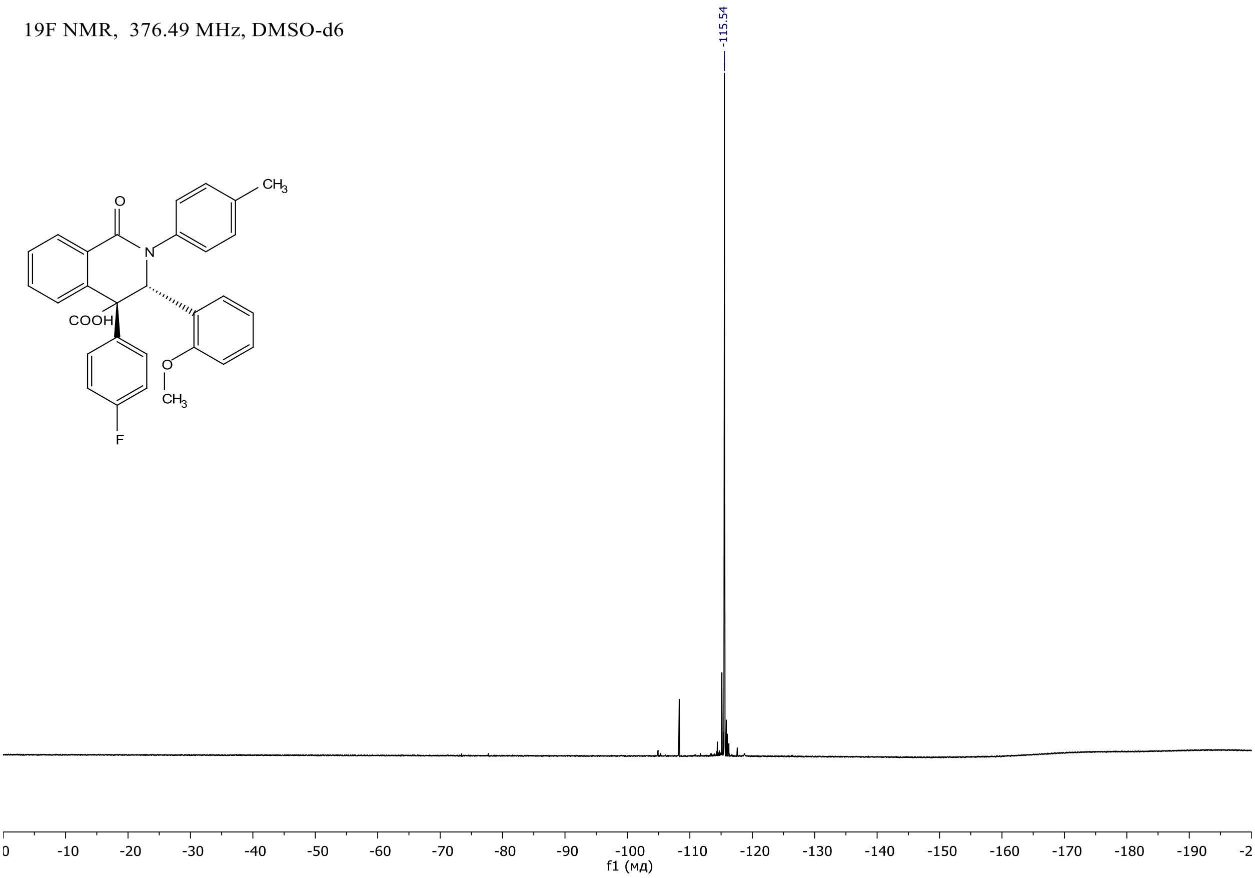

^{1}H, ^{13}C and ^{19}F NMR spectra of compound **9u**

1H NMR, 400.13 MHz, DMSO-d6

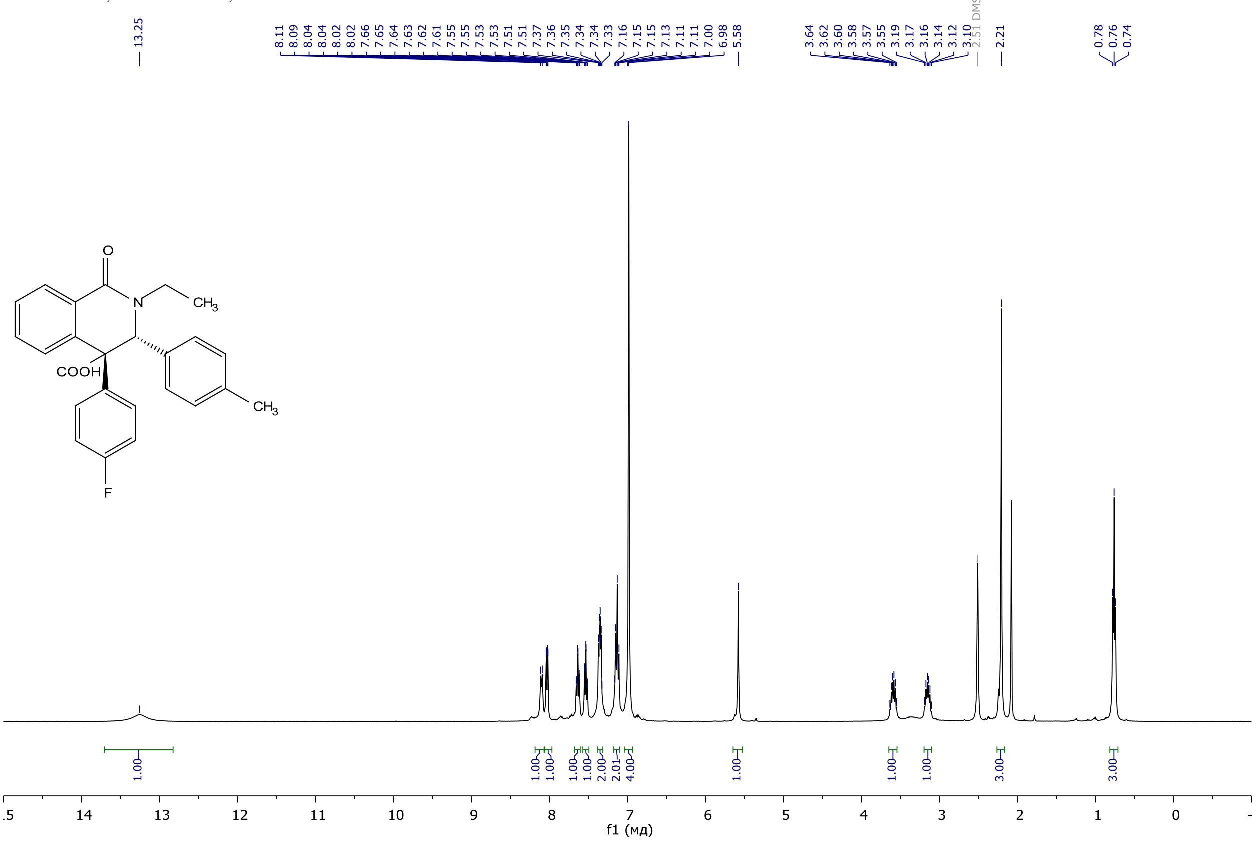

13C NMR, 100.61 MHz, DMSO-d6

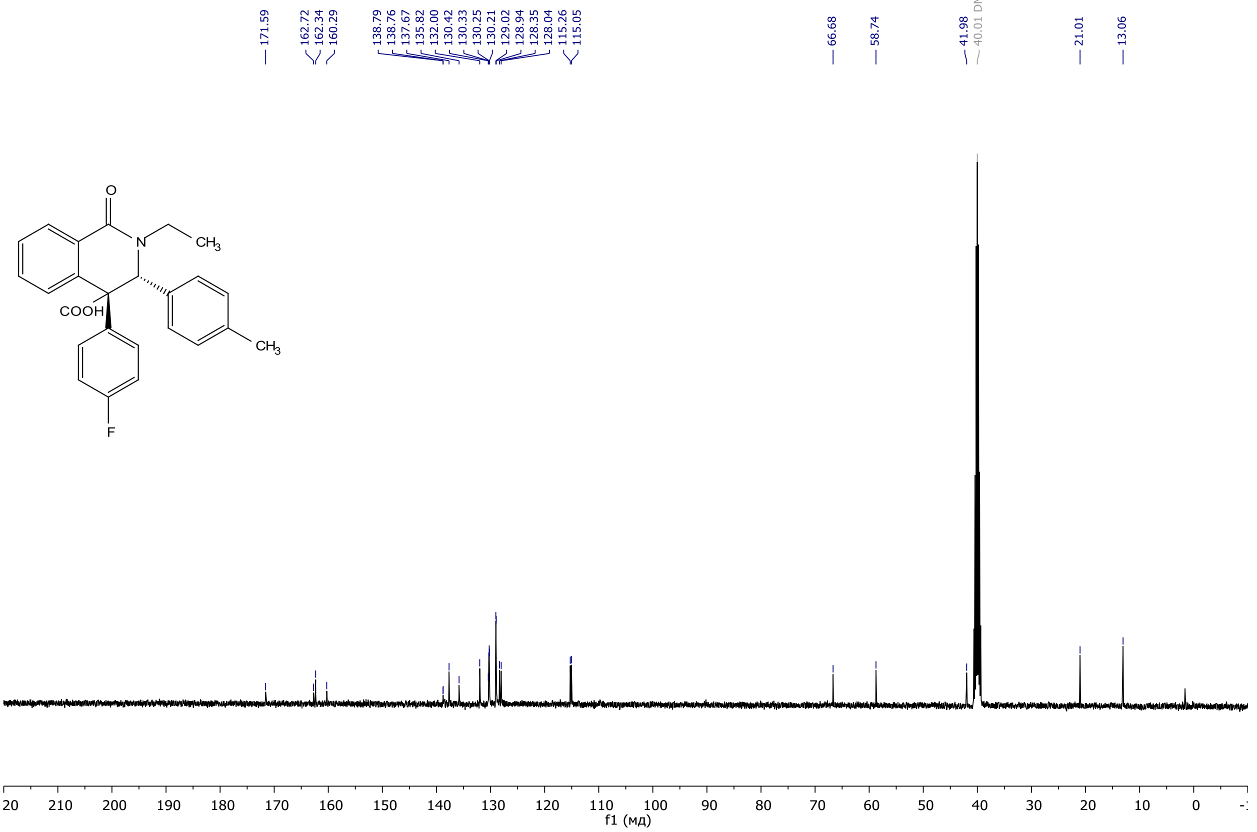

19F NMR, 376.49 MHz, DMSO-d6

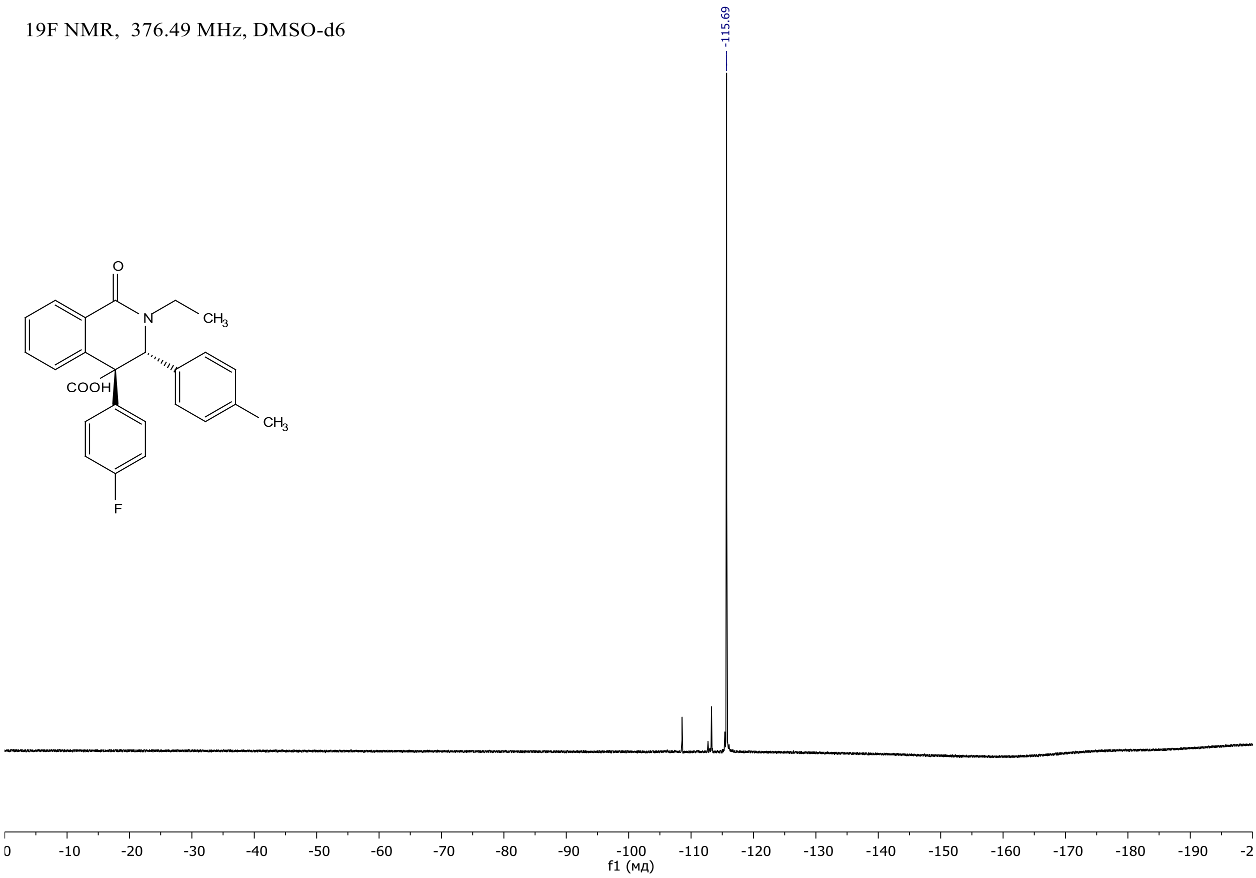

^{1}H and ^{13}C NMR spectra of compound **15**

1H NMR, 400.13 MHz, DMSO-d6

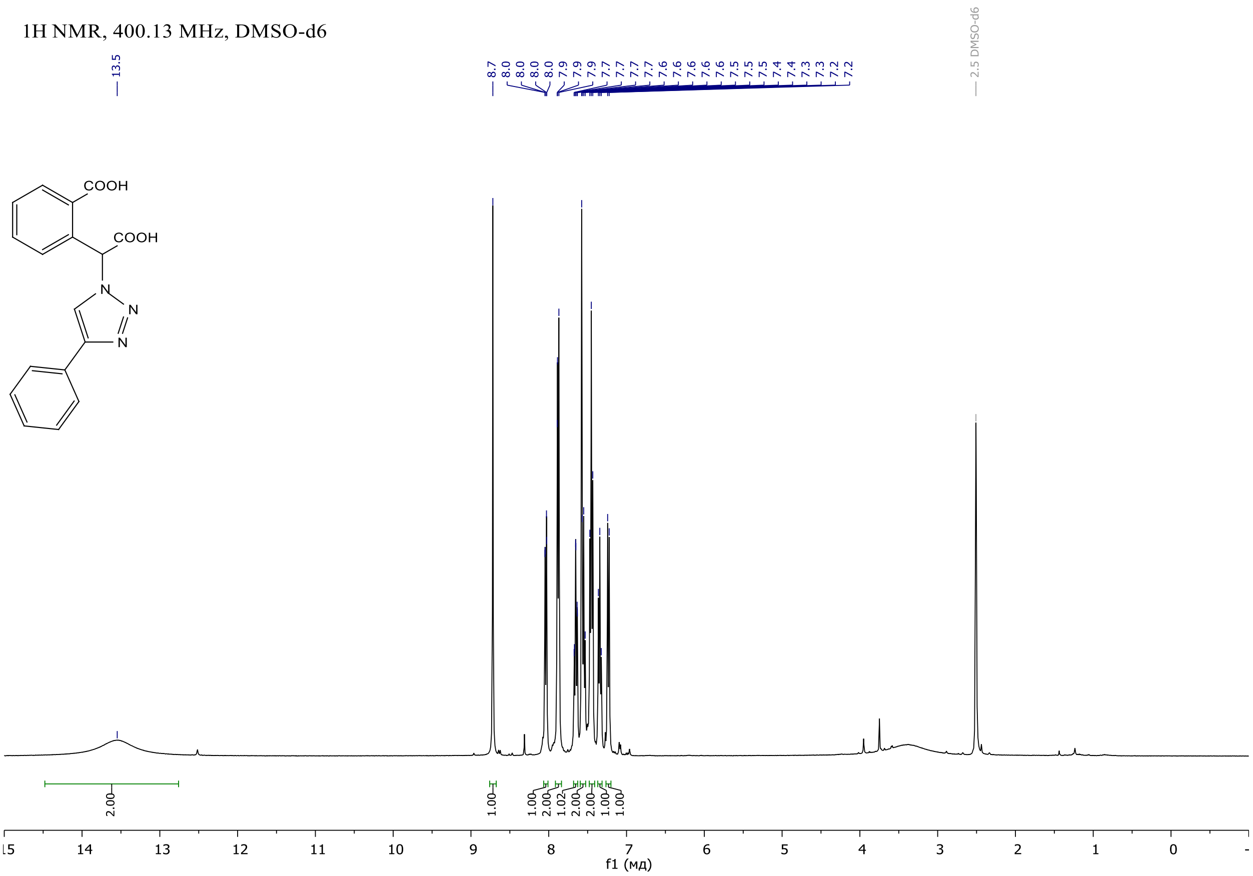

13C NMR, 100.61 MHz, DMSO-d6

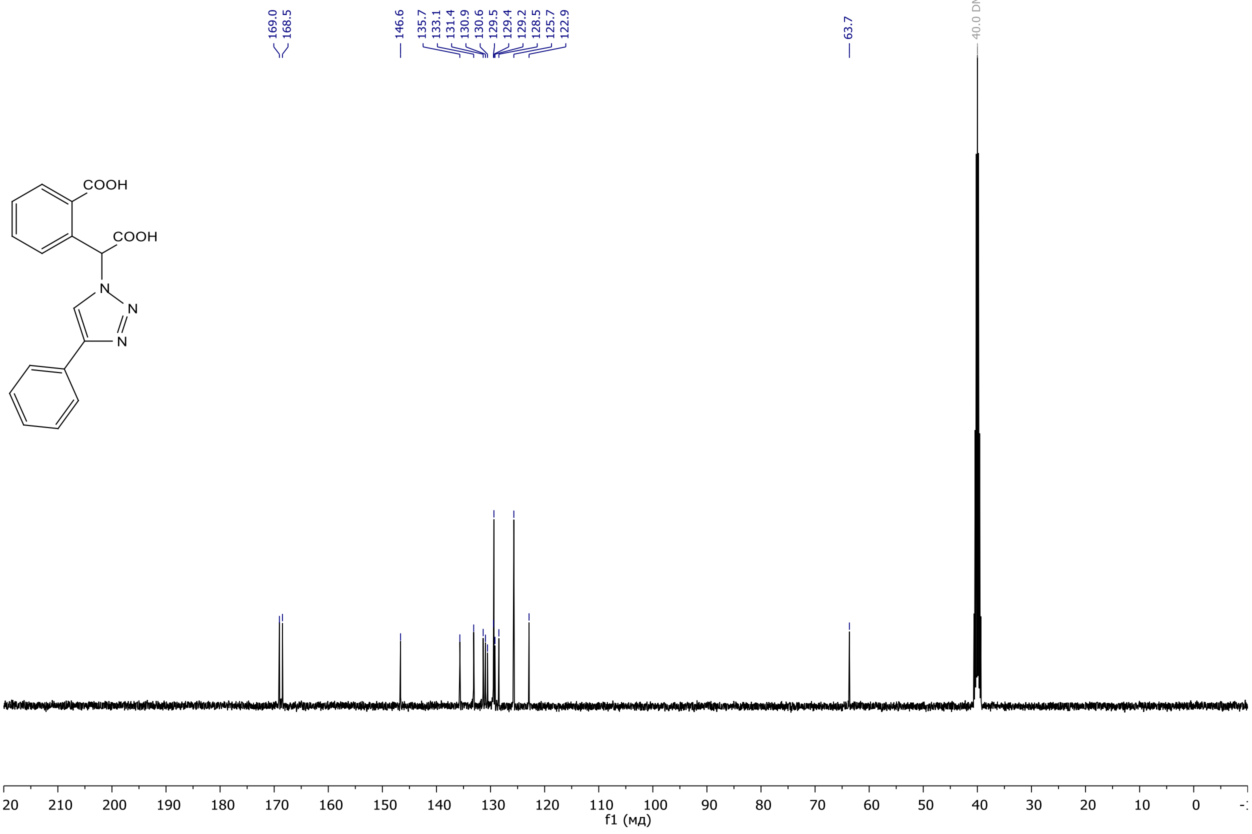

^{1}H and ^{13}C NMR spectra of compound **18**

1H NMR, 400.13 MHz, DMSO-d6

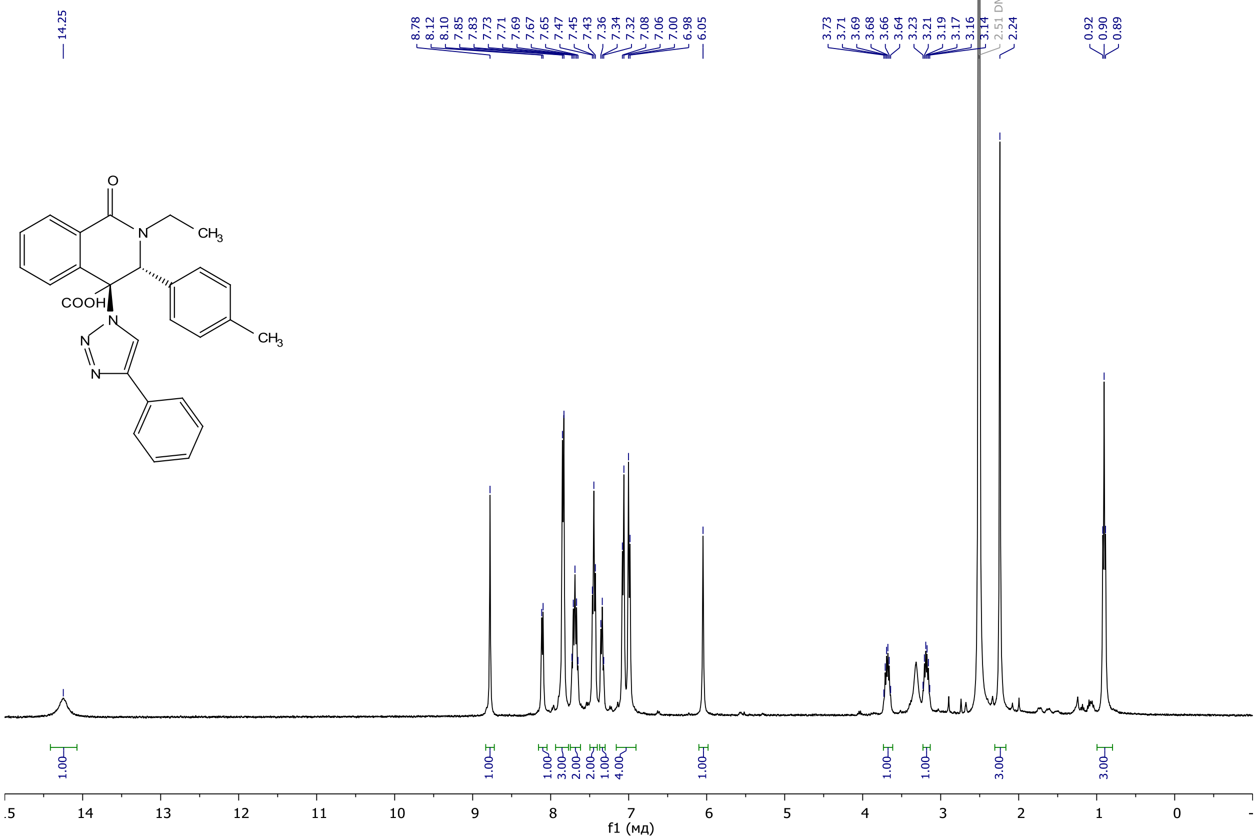

13C NMR, 100.61 MHz, DMSO-d6

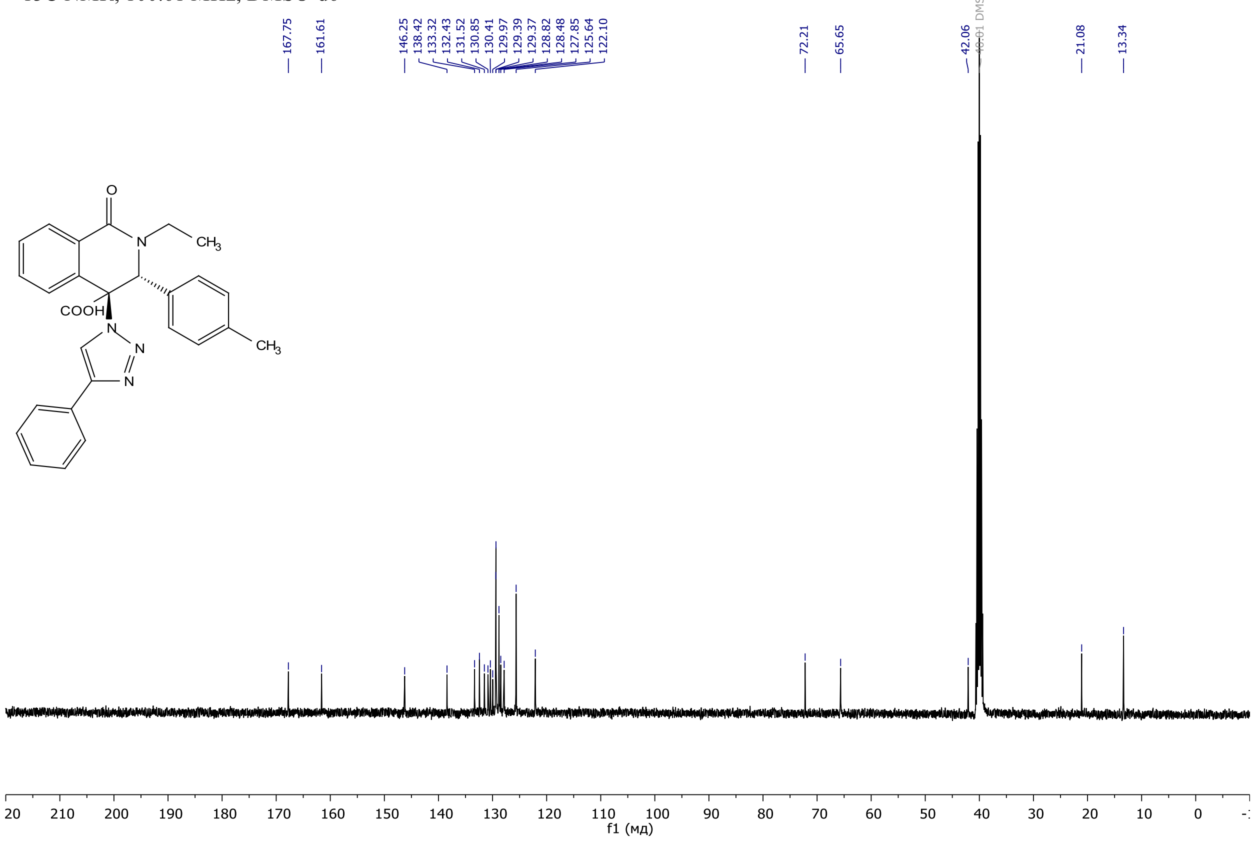

C:\Users\Brr\D...zxc\USA_41.mzML Injection 1 MS1 (+) profile MS + spectrum 0.00

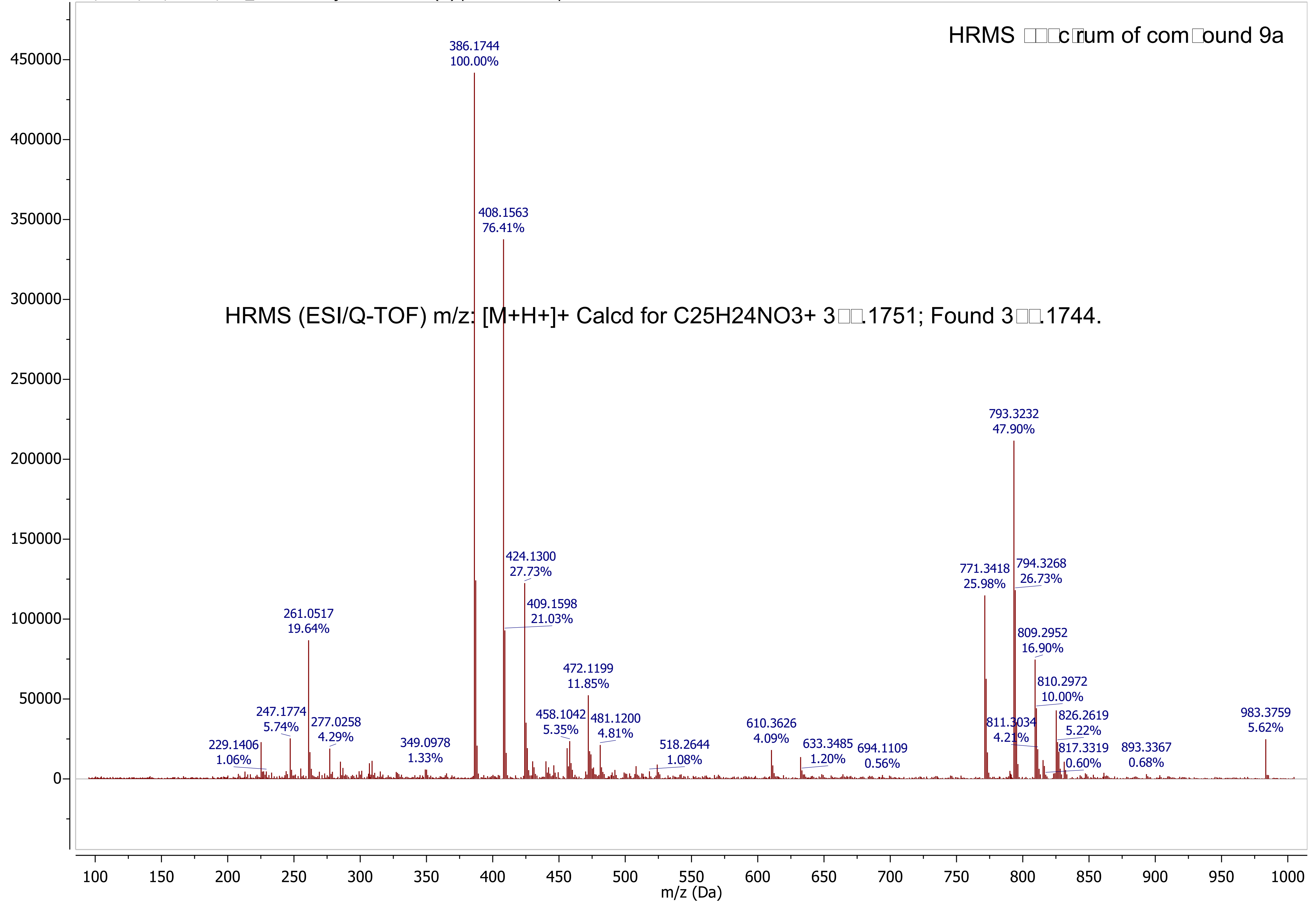

HRMS [illegible]c[illegible]rum of com[illegible]ound 9a

HRMS (ESI/Q-TOF) m/z: [M+H+]+ Calcd for C25H24NO3+ 3[illegible].1751; Found 3[illegible].1744.

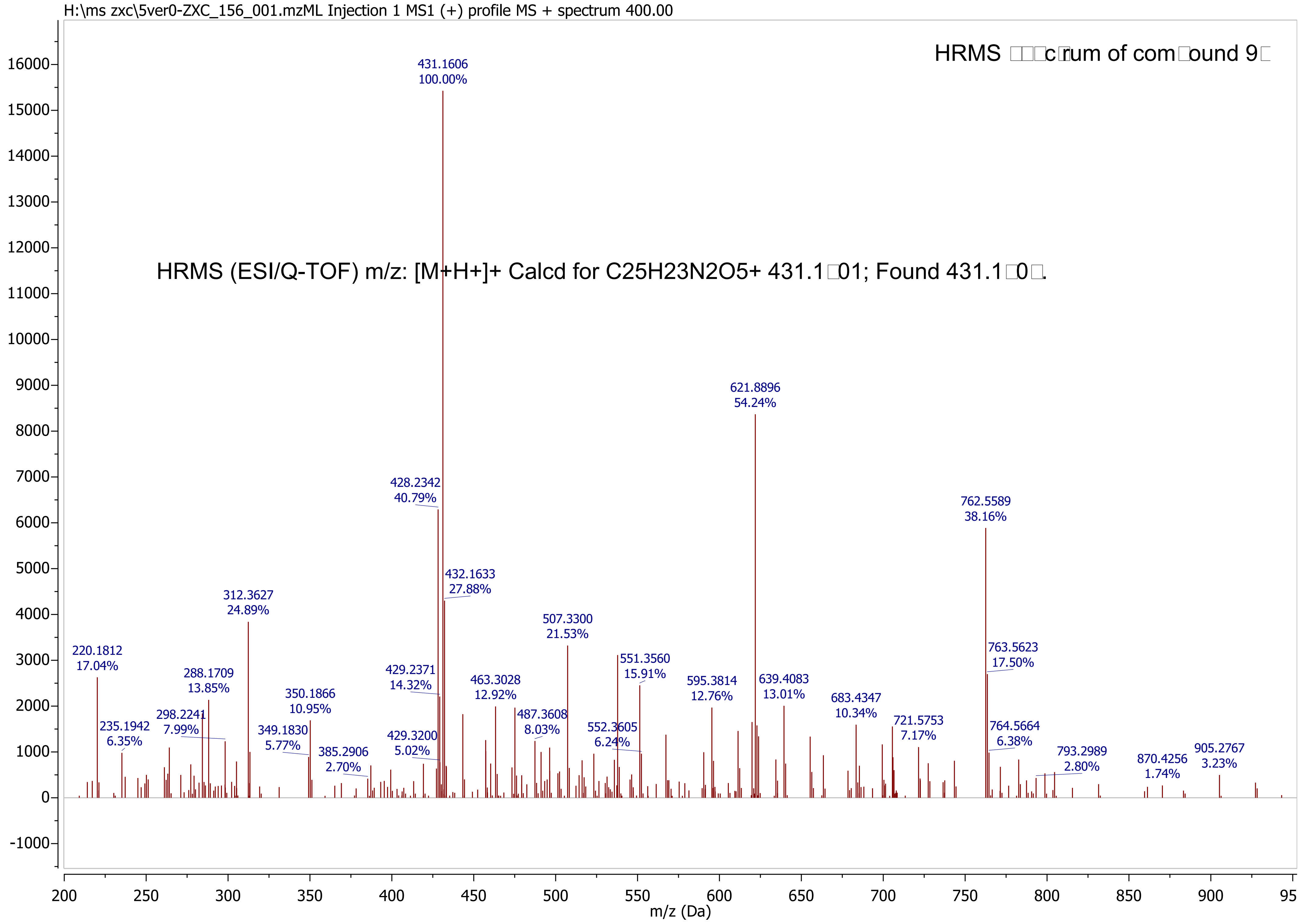
H:\ms zxc\5ver0-ZXC_156_001.mzML Injection 1 MS1 (+) profile MS + spectrum 400.00
HRMS [illegible]c[illegible]rum of com[illegible]ound 9[illegible]
HRMS (ESI/Q-TOF) m/z: [M+H+]+ Calcd for C25H23N2O5+ 431.1[illegible]01; Found 431.1[illegible]0[illegible].
431.1606 100.00%
621.8896 54.24%
428.2342 40.79%
762.5589 38.16%
432.1633 27.88%
312.3627 24.89%
507.3300 21.53%
763.5623 17.50%
220.1812 17.04%
551.3560 15.91%
288.1709 13.85%
429.2371 14.32%
463.3028 12.92%
595.3814 12.76%
639.4083 13.01%
350.1866 10.95%
683.4347 10.34%
487.3608 8.03%
298.2241 7.99%
721.5753 7.17%
235.1942 6.35%
552.3605 6.24%
764.5664 6.38%
349.1830 5.77%
429.3200 5.02%
385.2906 2.70%
793.2989 2.80%
870.4256 1.74%
905.2767 3.23%
m/z (Da)

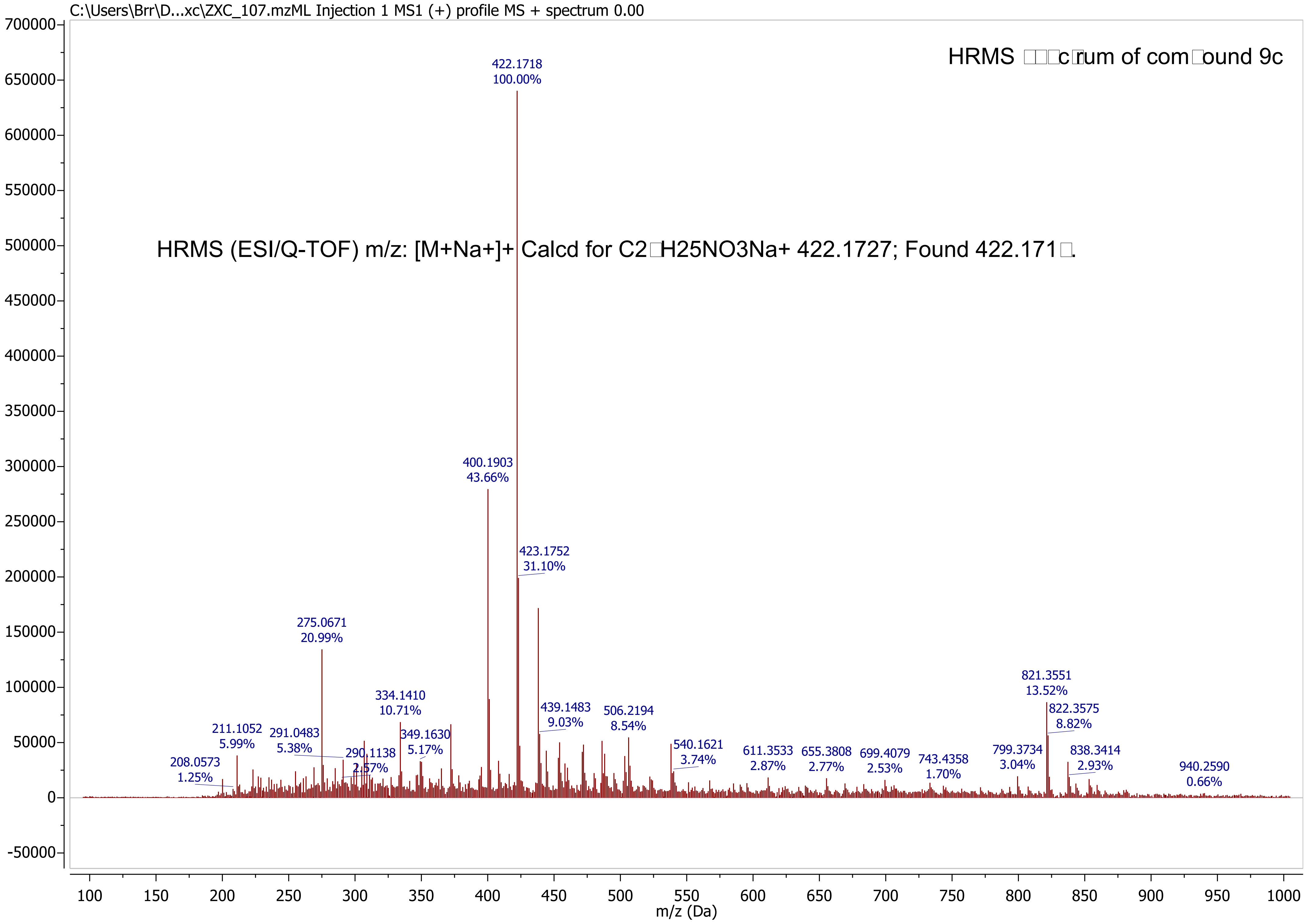
C:\Users\Brr\D...xc\ZXC_107.mzML Injection 1 MS1 (+) profile MS + spectrum 0.00
HRMS □□□c□rum of com□ound 9c
HRMS (ESI/Q-TOF) m/z: [M+Na+]+ Calcd for C2□H25NO3Na+ 422.1727; Found 422.171□.
422.1718 100.00%
400.1903 43.66%
423.1752 31.10%
275.0671 20.99%
821.3551 13.52%
334.1410 10.71%
439.1483 9.03%
822.3575 8.82%
506.2194 8.54%
211.1052 5.99%
291.0483 5.38%
349.1630 5.17%
290.1138 2.57%
208.0573 1.25%
540.1621 3.74%
611.3533 2.87%
655.3808 2.77%
699.4079 2.53%
743.4358 1.70%
799.3734 3.04%
838.3414 2.93%
940.2590 0.66%
m/z (Da)

C:\Users\Brr\D...xc\ZXC_215.mzML Injection 1 MS1 (+) profile MS + spectrum 0.00

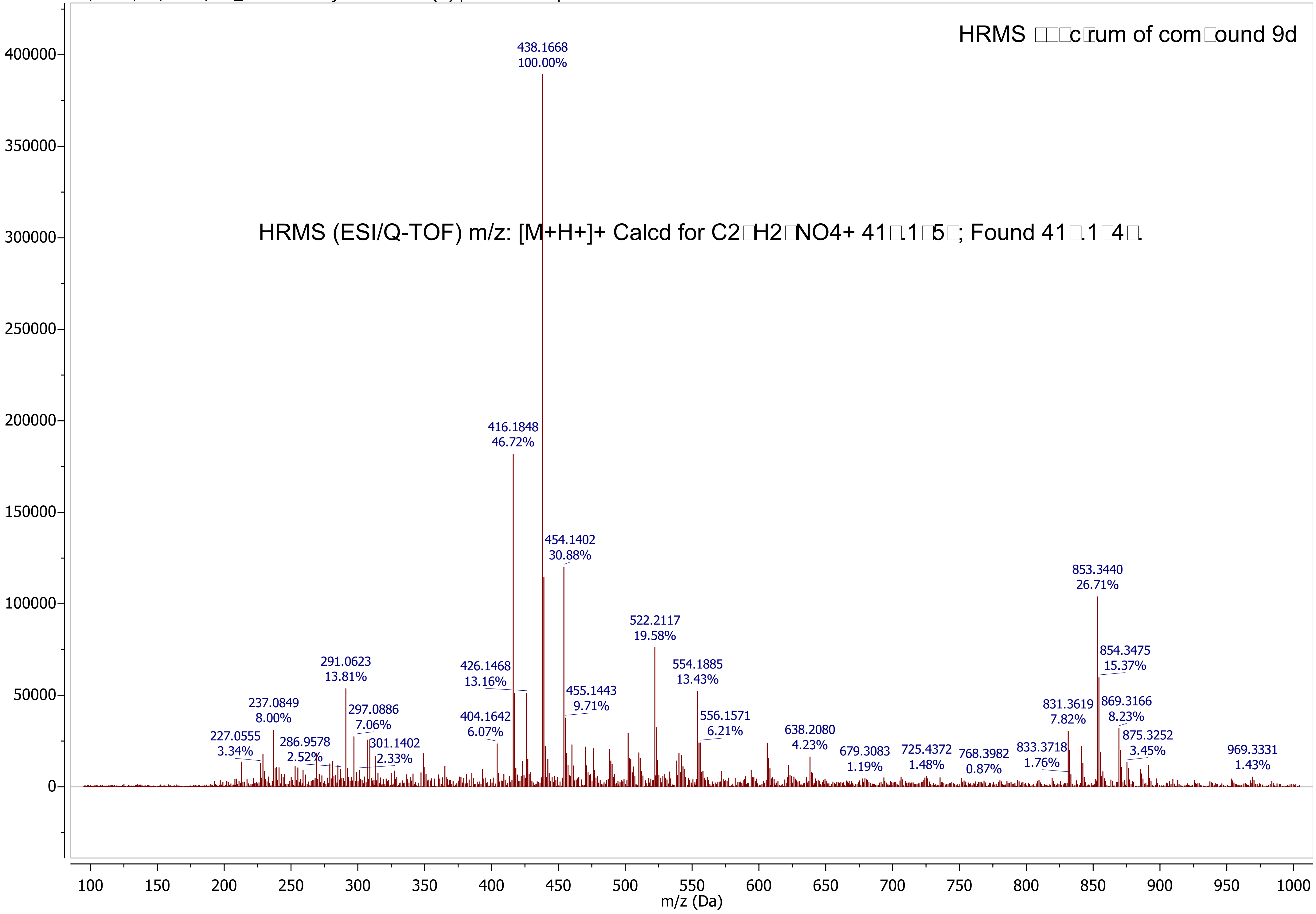

C:\Users\Brr\D...xc\ZXC_277.mzML Injection 1 MS1 (+) profile MS + spectrum 0.00

HRMS [illegible]c[illegible]rum of com[illegible]ound 9[illegible]

HRMS (ESI/Q-TOF) m/z: [M+H+]+ Calcd for C25H22Cl2NO3+ 454.0971; Found 454.09[illegible]5.

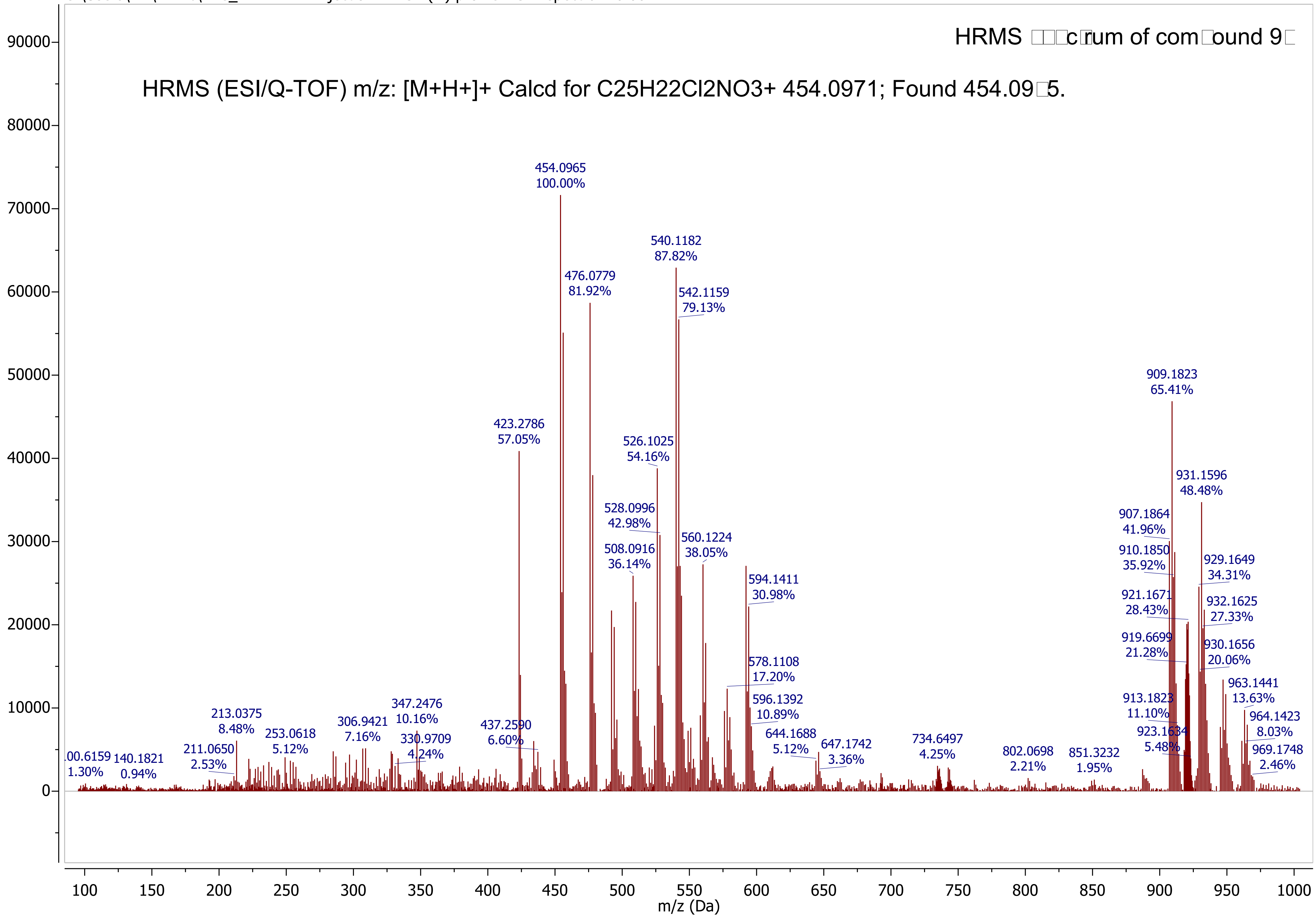

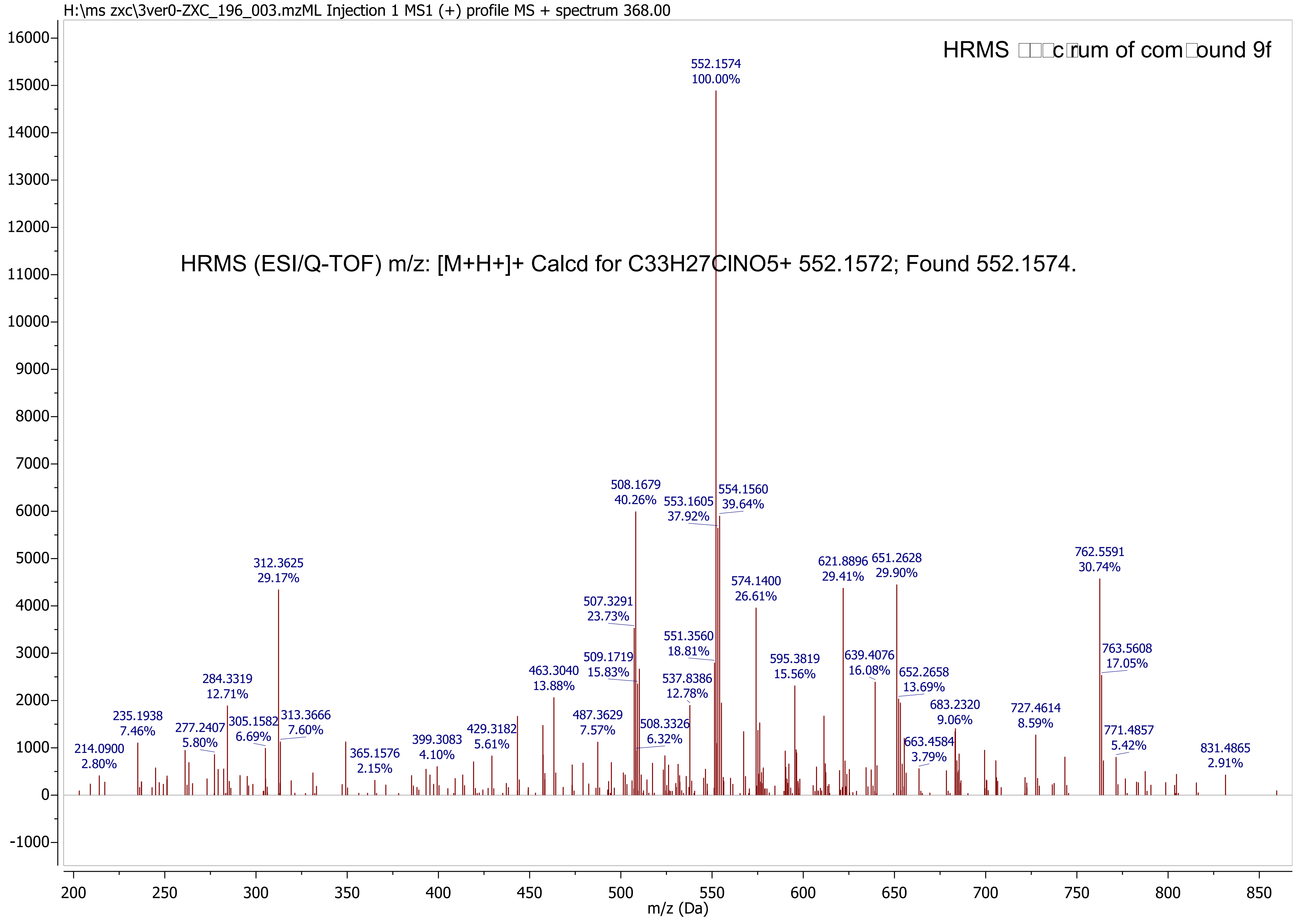

H:\ms zxc\3ver0-ZXC_196_003.mzML Injection 1 MS1 (+) profile MS + spectrum 368.00
HRMS c rum of com ound 9f
HRMS (ESI/Q-TOF) m/z: [M+H+]+ Calcd for C33H27ClNO5+ 552.1572; Found 552.1574.
552.1574 100.00%
508.1679 40.26%
554.1560 39.64%
553.1605 37.92%
762.5591 30.74%
651.2628 29.90%
621.8896 29.41%
312.3625 29.17%
574.1400 26.61%
507.3291 23.73%
551.3560 18.81%
763.5608 17.05%
639.4076 16.08%
509.1719 15.83%
595.3819 15.56%
463.3040 13.88%
652.2658 13.69%
537.8386 12.78%
284.3319 12.71%
683.2320 9.06%
727.4614 8.59%
313.3666 7.60%
487.3629 7.57%
235.1938 7.46%
305.1582 6.69%
508.3326 6.32%
277.2407 5.80%
429.3182 5.61%
771.4857 5.42%
399.3083 4.10%
663.4584 3.79%
831.4865 2.91%
214.0900 2.80%
365.1576 2.15%
m/z (Da)

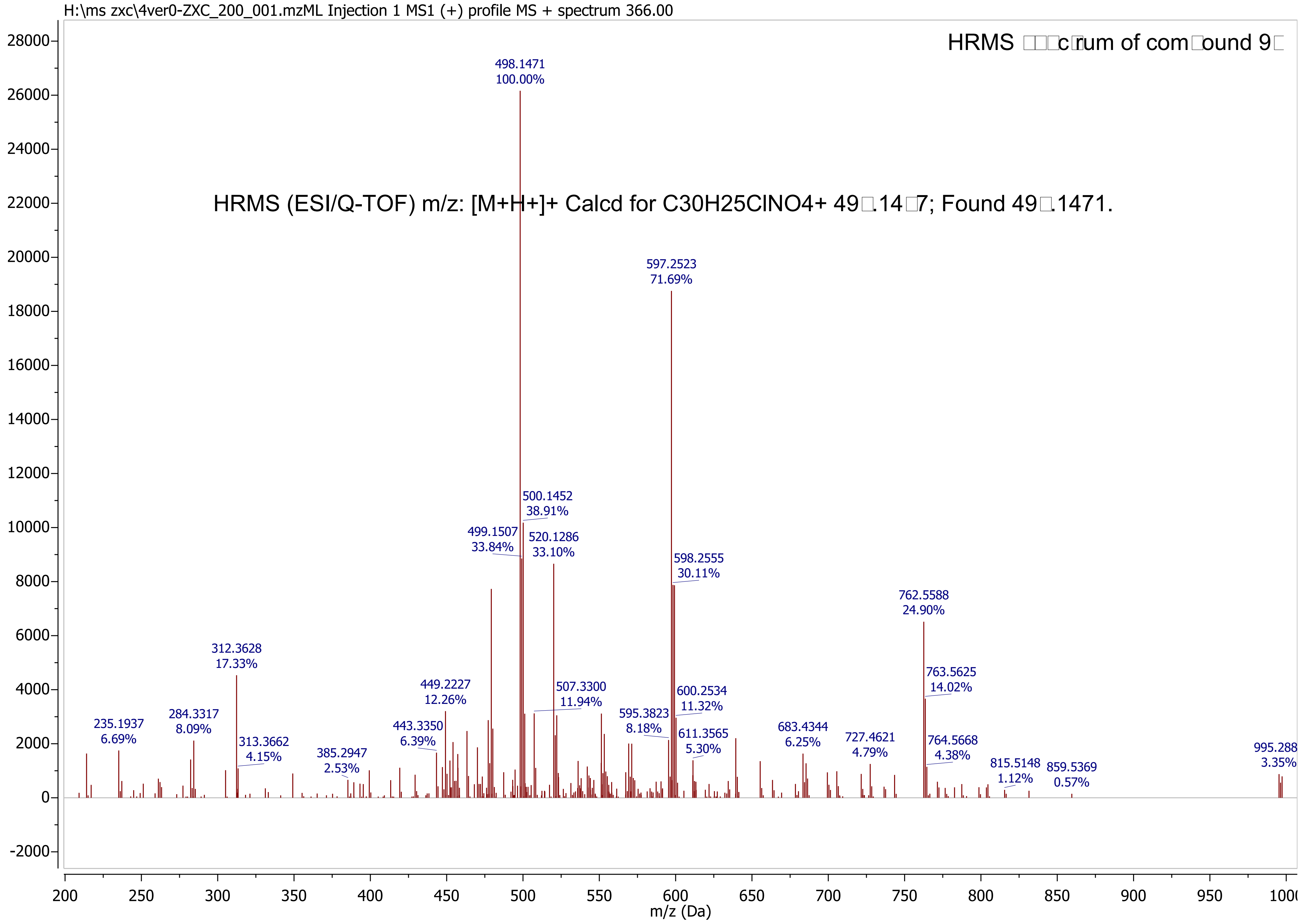
H:\ms zxc\4ver0-ZXC_200_001.mzML Injection 1 MS1 (+) profile MS + spectrum 366.00
HRMS [illegible]c[illegible]rum of com[illegible]ound 9[illegible]
HRMS (ESI/Q-TOF) m/z: [M+H+]+ Calcd for C30H25ClNO4+ 49[illegible].14[illegible]7; Found 49[illegible].1471.
498.1471 100.00%
597.2523 71.69%
500.1452 38.91%
499.1507 33.84%
520.1286 33.10%
598.2555 30.11%
762.5588 24.90%
312.3628 17.33%
763.5625 14.02%
449.2227 12.26%
507.3300 11.94%
600.2534 11.32%
235.1937 6.69%
284.3317 8.09%
313.3662 4.15%
385.2947 2.53%
443.3350 6.39%
595.3823 8.18%
611.3565 5.30%
683.4344 6.25%
727.4621 4.79%
764.5668 4.38%
815.5148 1.12%
859.5369 0.57%
995.288 3.35%
m/z (Da)

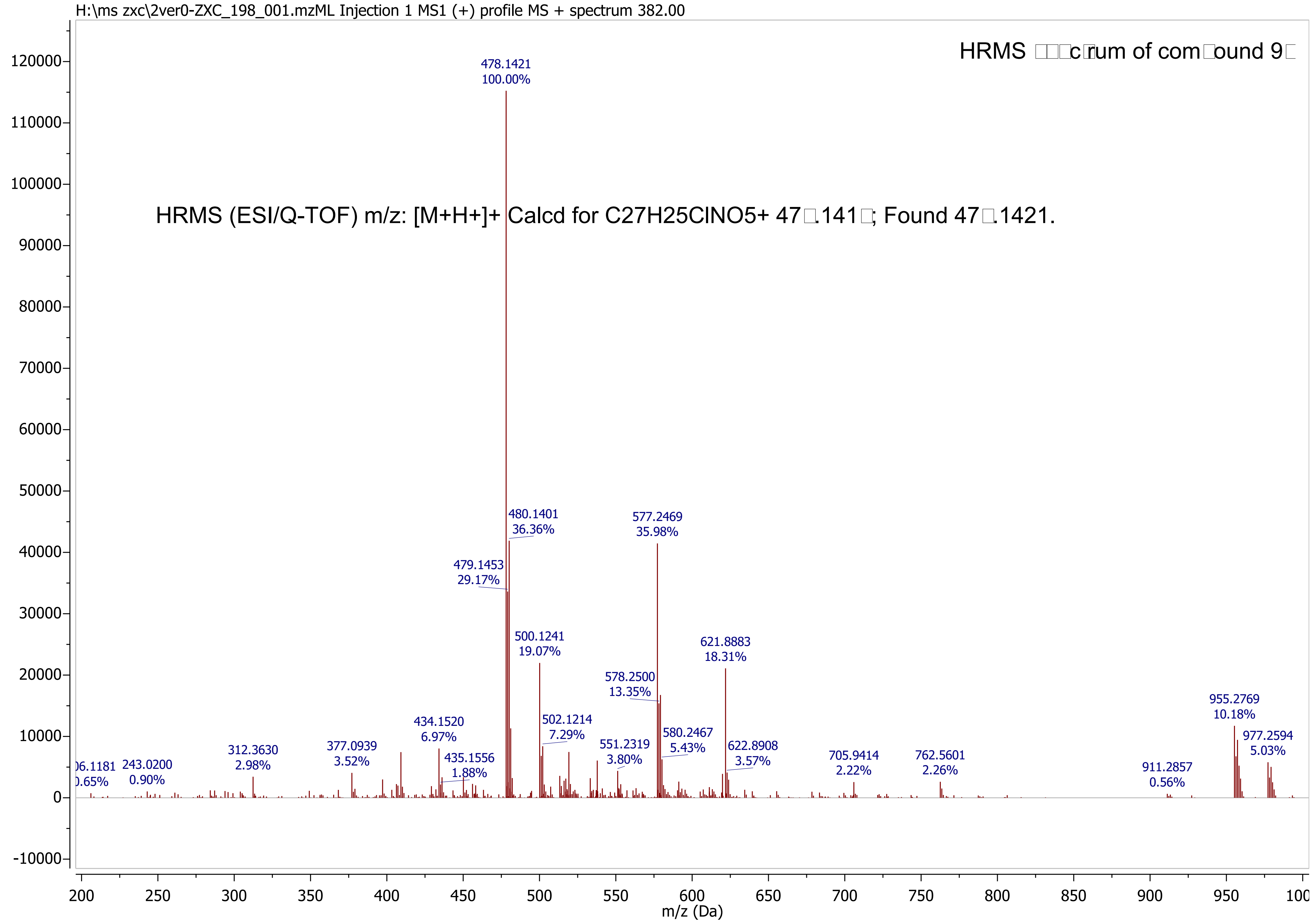
H:\ms zxc\2ver0-ZXC_198_001.mzML Injection 1 MS1 (+) profile MS + spectrum 382.00
HRMS □□□c□rum of com□ound 9□
HRMS (ESI/Q-TOF) m/z: [M+H+]+ Calcd for C27H25ClNO5+ 47□.141□; Found 47□.1421.
478.1421
100.00%
480.1401
36.36%
479.1453
29.17%
577.2469
35.98%
500.1241
19.07%
621.8883
18.31%
578.2500
13.35%
955.2769
10.18%
434.1520
6.97%
502.1214
7.29%
580.2467
5.43%
977.2594
5.03%
312.3630
2.98%
377.0939
3.52%
435.1556
1.88%
551.2319
3.80%
622.8908
3.57%
705.9414
2.22%
762.5601
2.26%
911.2857
0.56%
243.0200
0.90%
)6.1181
).65%
m/z (Da)

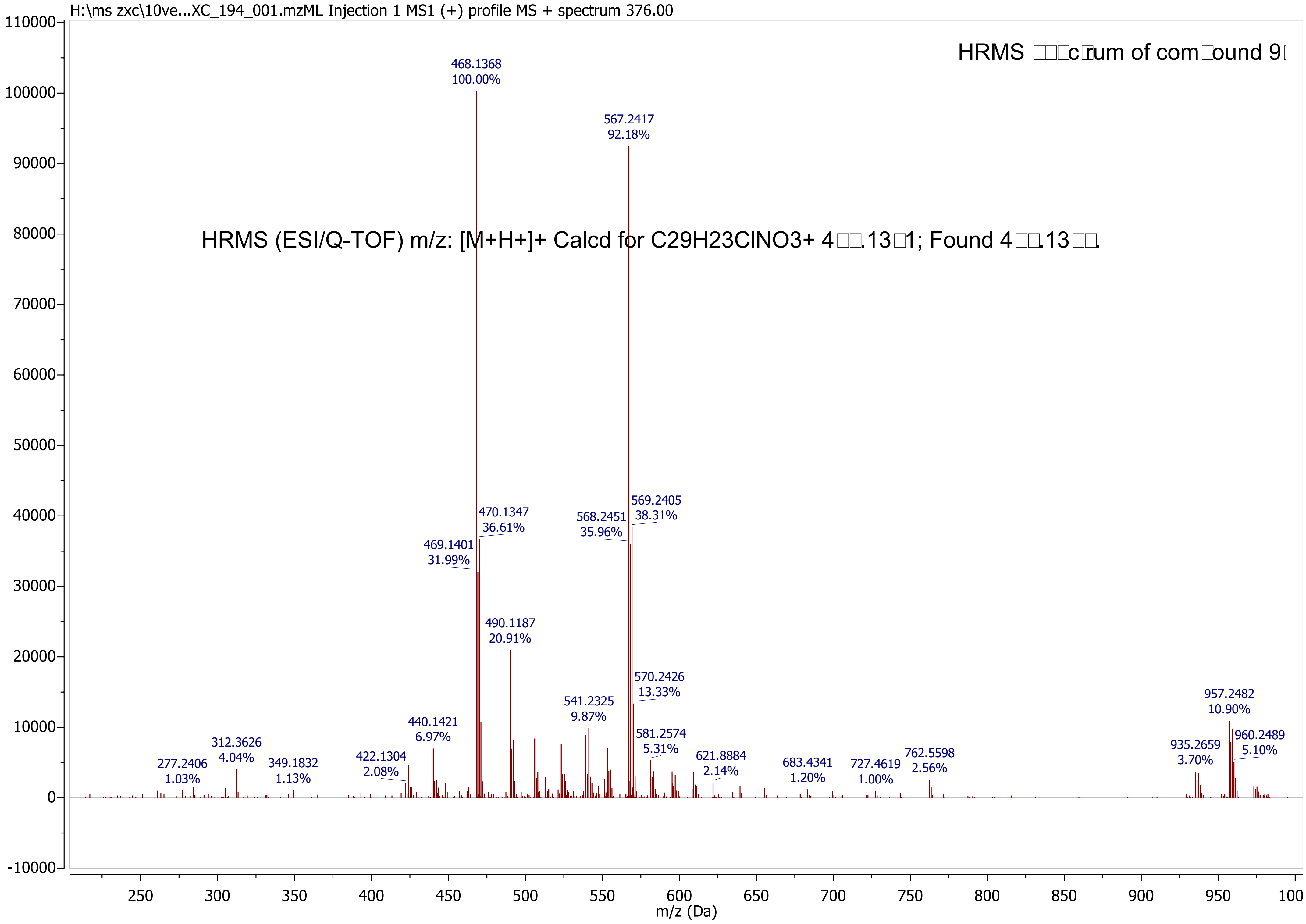
H:\ms zxc\10ve...XC_194_001.mzML Injection 1 MS1 (+) profile MS + spectrum 376.00
HRMS (ESI/Q-TOF) m/z: [M+H+]+ Calcd for C29H23ClNO3+
468.1368
100.00%
567.2417
92.18%
470.1347
36.61%
469.1401
31.99%
568.2451
35.96%
569.2405
38.31%
490.1187
20.91%
570.2426
13.33%
541.2325
9.87%
440.1421
6.97%
581.2574
5.31%
312.3626
4.04%
277.2406
1.03%
349.1832
1.13%
422.1304
2.08%
621.8884
2.14%
683.4341
1.20%
727.4619
1.00%
762.5598
2.56%
935.2659
3.70%
957.2482
10.90%
960.2489
5.10%
m/z (Da)

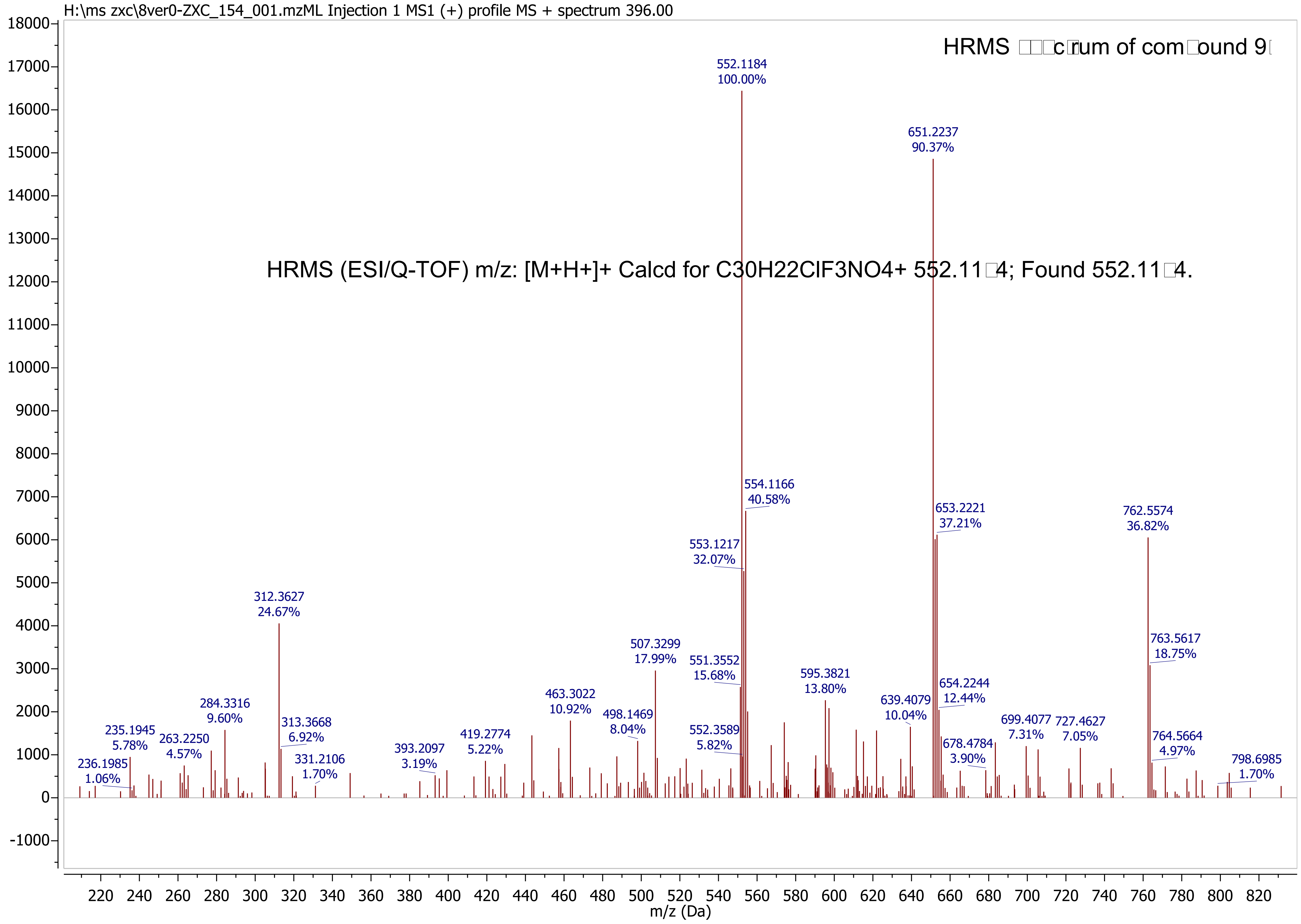
H:\ms zxc\8ver0-ZXC_154_001.mzML Injection 1 MS1 (+) profile MS + spectrum 396.00
HRMS □□□c□rum of com□ound 9□
HRMS (ESI/Q-TOF) m/z: [M+H+]+ Calcd for C30H22ClF3NO4+ 552.11□4; Found 552.11□4.
552.1184 100.00%
651.2237 90.37%
554.1166 40.58%
653.2221 37.21%
762.5574 36.82%
553.1217 32.07%
312.3627 24.67%
763.5617 18.75%
507.3299 17.99%
551.3552 15.68%
595.3821 13.80%
654.2244 12.44%
463.3022 10.92%
639.4079 10.04%
284.3316 9.60%
498.1469 8.04%
699.4077 7.31%
727.4627 7.05%
313.3668 6.92%
552.3589 5.82%
235.1945 5.78%
419.2774 5.22%
764.5664 4.97%
263.2250 4.57%
678.4784 3.90%
393.2097 3.19%
331.2106 1.70%
798.6985 1.70%
236.1985 1.06%
m/z (Da)

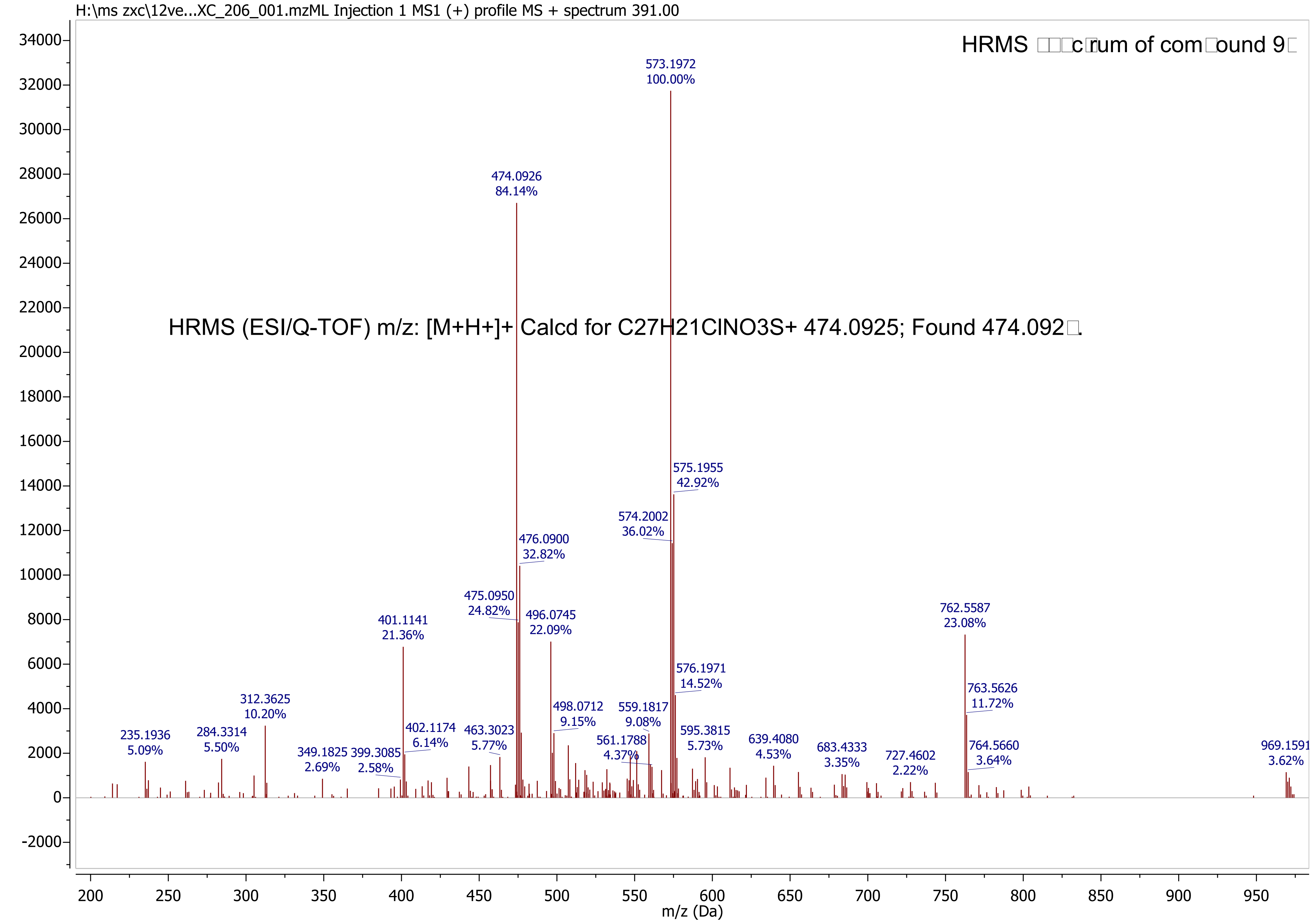

HRMS [illegible]c[illegible]rum of com[illegible]ound 9[illegible]

HRMS (ESI/Q-TOF) m/z: [M+H+]+ Calcd for C27H21ClNO3S+ 474.0925; Found 474.092[illegible].

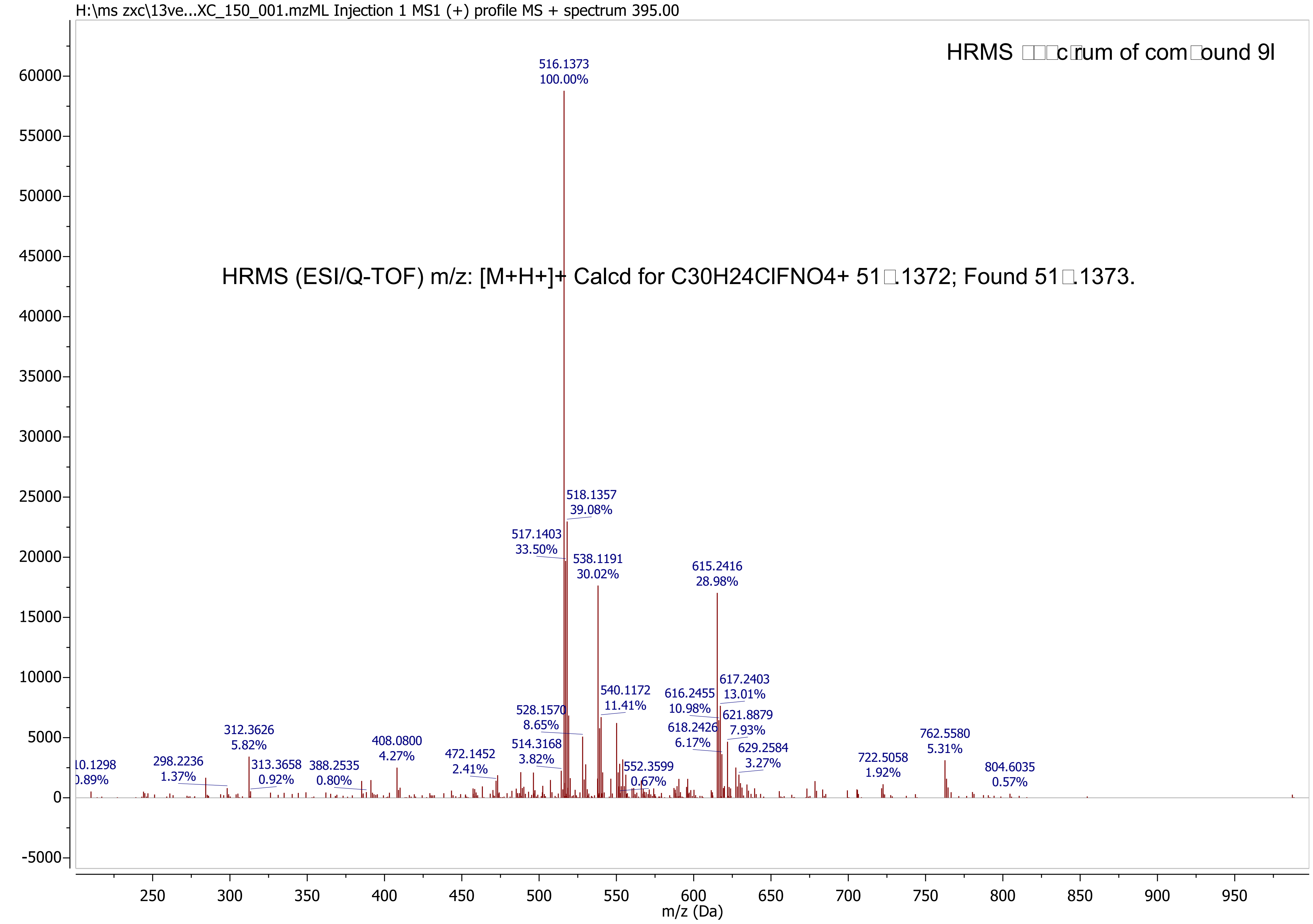
H:\ms zxc\13ve...XC_150_001.mzML Injection 1 MS1 (+) profile MS + spectrum 395.00
HRMS □□□c□rum of com□ound 9l
HRMS (ESI/Q-TOF) m/z: [M+H+]+ Calcd for C30H24ClFNO4+ 51□.1372; Found 51□.1373.
516.1373 100.00%
518.1357 39.08%
517.1403 33.50%
538.1191 30.02%
615.2416 28.98%
617.2403 13.01%
540.1172 11.41%
616.2455 10.98%
528.1570 8.65%
621.8879 7.93%
618.2426 6.17%
312.3626 5.82%
762.5580 5.31%
408.0800 4.27%
514.3168 3.82%
629.2584 3.27%
472.1452 2.41%
722.5058 1.92%
298.2236 1.37%
313.3658 0.92%
10.1298 0.89%
388.2535 0.80%
552.3599 0.67%
804.6035 0.57%
m/z (Da)

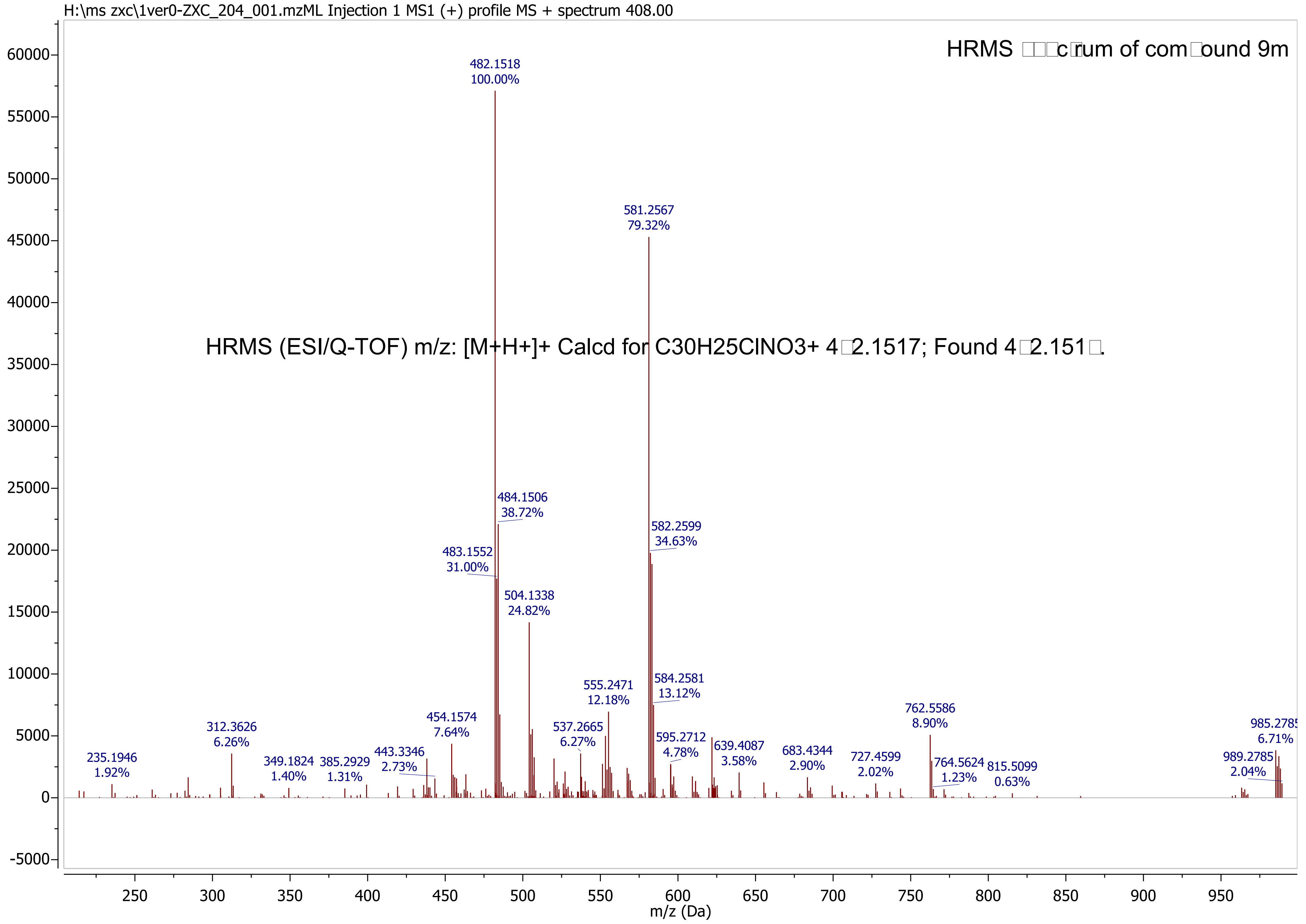

H:\ms zxc\1ver0-ZXC_204_001.mzML Injection 1 MS1 (+) profile MS + spectrum 408.00
HRMS
9m
HRMS (ESI/Q-TOF) m/z: [M+H+]+ Calcd for C30H25ClNO3+
482.1518 100.00%
581.2567 79.32%
484.1506 38.72%
582.2599 34.63%
483.1552 31.00%
504.1338 24.82%
555.2471 12.18%
584.2581 13.12%
454.1574 7.64%
537.2665 6.27%
312.3626 6.26%
762.5586 8.90%
985.2785 6.71%
595.2712 4.78%
639.4087 3.58%
683.4344 2.90%
443.3346 2.73%
727.4599 2.02%
235.1946 1.92%
349.1824 1.40%
385.2929 1.31%
764.5624 1.23%
815.5099 0.63%
989.2785 2.04%
m/z (Da)

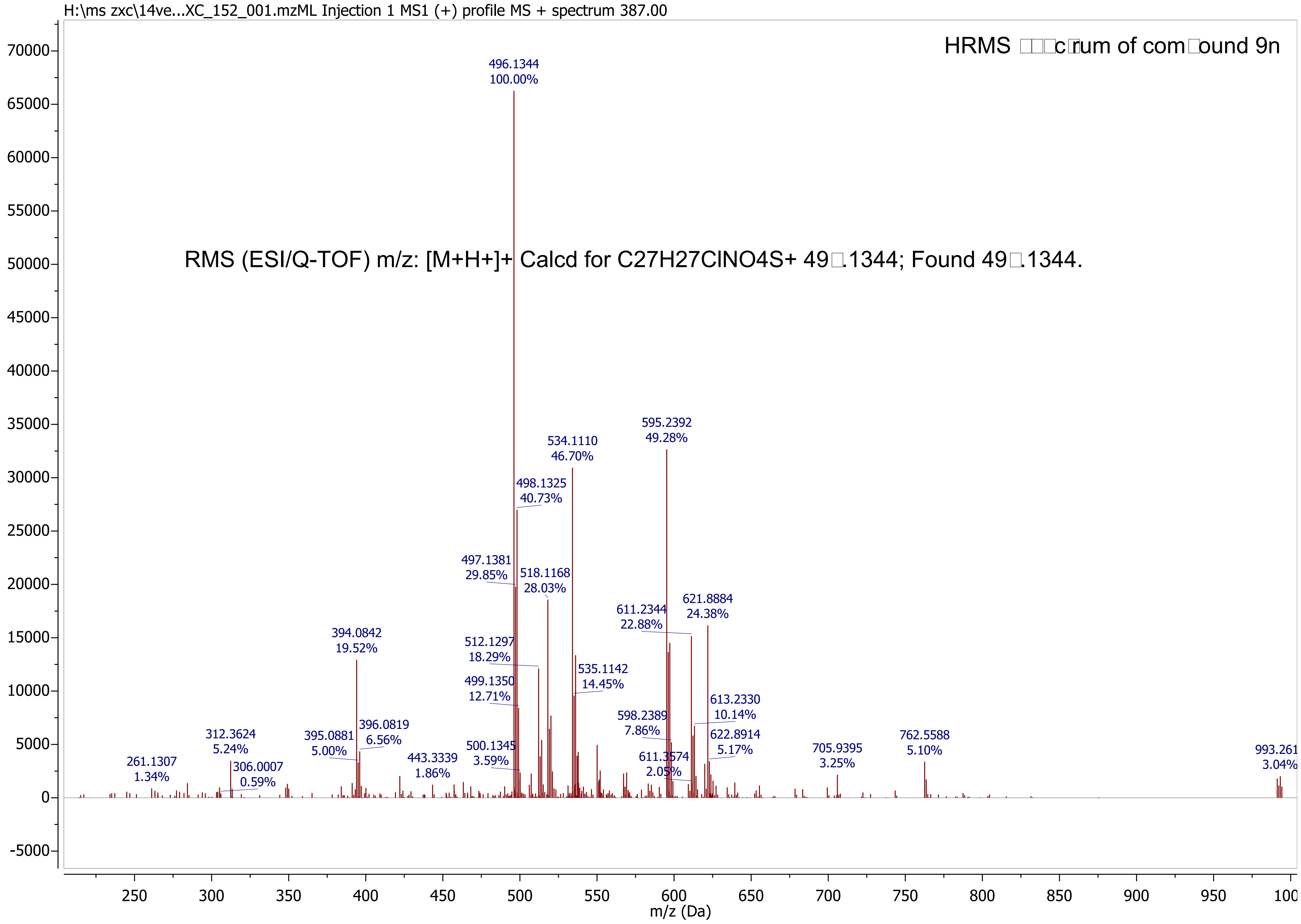
H:\ms zxc\14ve...XC_152_001.mzML Injection 1 MS1 (+) profile MS + spectrum 387.00
HRMS □□□c□rum of com□ound 9n
RMS (ESI/Q-TOF) m/z: [M+H+]+ Calcd for C27H27ClNO4S+ 49□.1344; Found 49□.1344.
496.1344 100.00%
595.2392 49.28%
534.1110 46.70%
498.1325 40.73%
497.1381 29.85%
518.1168 28.03%
621.8884 24.38%
611.2344 22.88%
394.0842 19.52%
512.1297 18.29%
535.1142 14.45%
499.1350 12.71%
613.2330 10.14%
598.2389 7.86%
396.0819 6.56%
312.3624 5.24%
622.8914 5.17%
762.5588 5.10%
395.0881 5.00%
500.1345 3.59%
705.9395 3.25%
993.261 3.04%
611.3574 2.05%
443.3339 1.86%
261.1307 1.34%
306.0007 0.59%
70000
65000
60000
55000
50000
45000
40000
35000
30000
25000
20000
15000
10000
5000
0
-5000
250 300 350 400 450 500 550 600 650 700 750 800 850 900 950 100
m/z (Da)

C:\Users\Brr\D...xc\ZXC_106.mzML Injection 1 MS1 (+) profile MS + spectrum 0.00

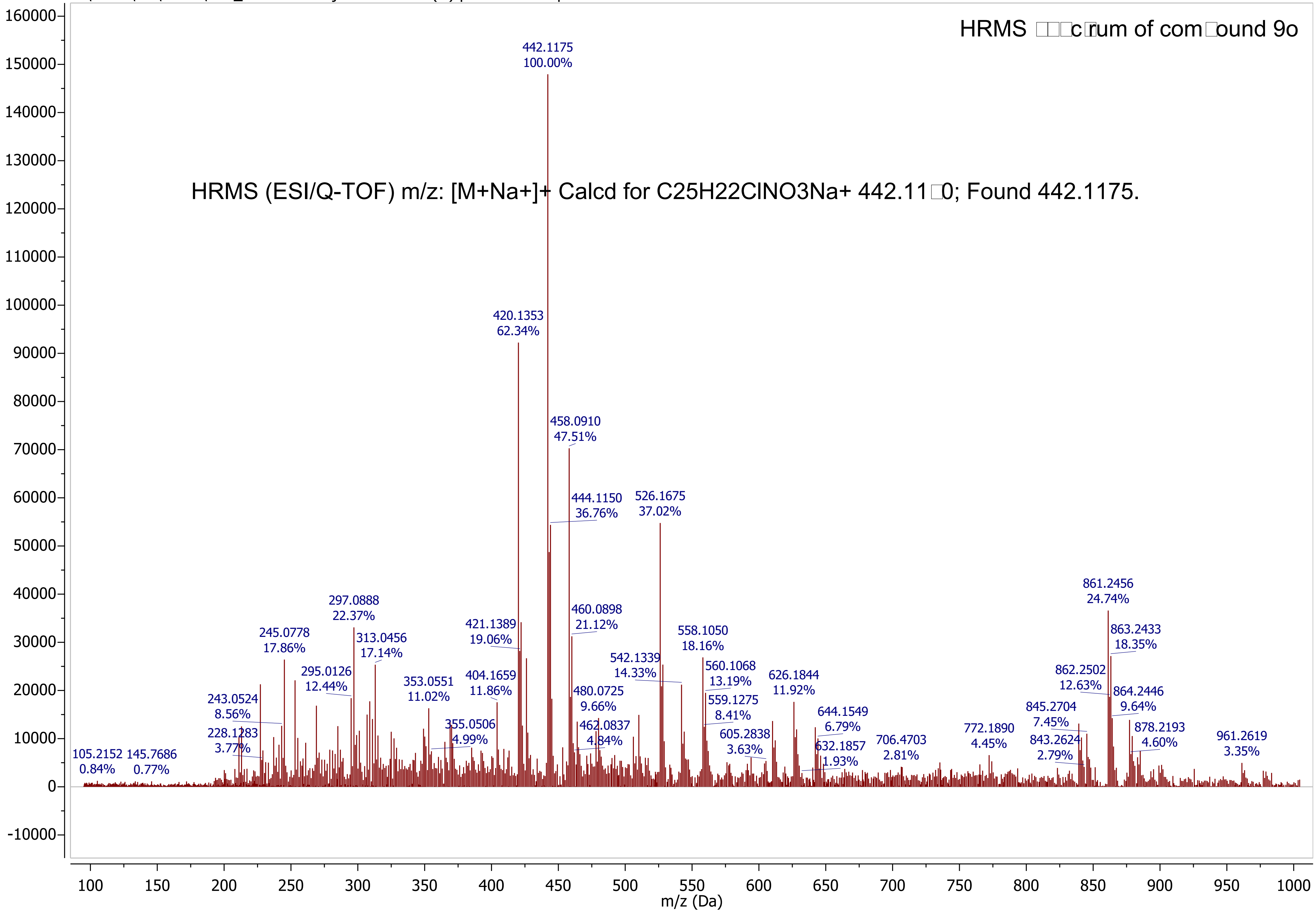

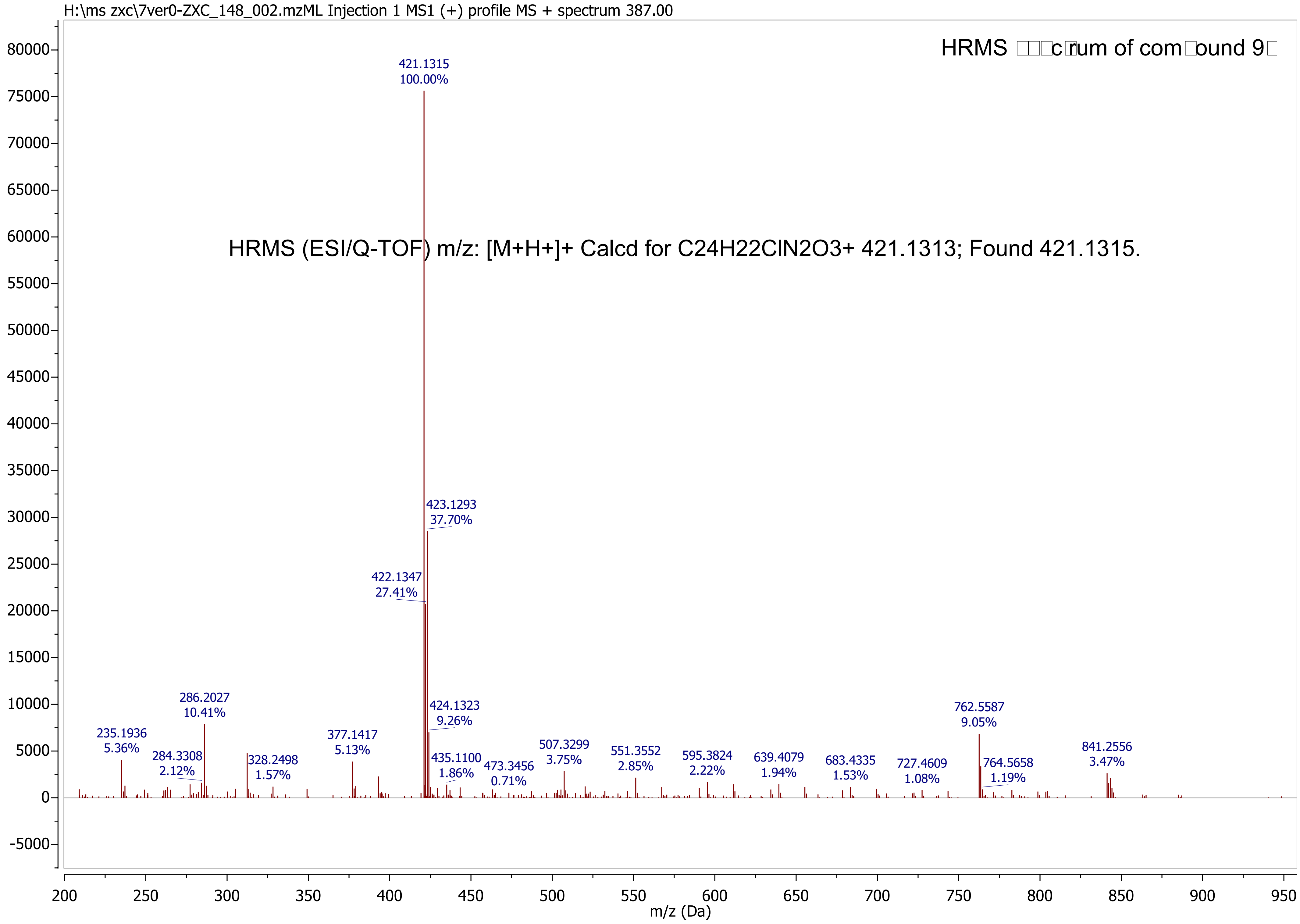
H:\ms zxc\7ver0-ZXC_148_002.mzML Injection 1 MS1 (+) profile MS + spectrum 387.00
HRMS
HRMS (ESI/Q-TOF) m/z: [M+H+]+ Calcd for C24H22ClN2O3+ 421.1313; Found 421.1315.
421.1315
100.00%
423.1293
37.70%
422.1347
27.41%
424.1323
9.26%
286.2027
10.41%
235.1936
5.36%
284.3308
2.12%
328.2498
1.57%
377.1417
5.13%
435.1100
1.86%
473.3456
0.71%
507.3299
3.75%
551.3552
2.85%
595.3824
2.22%
639.4079
1.94%
683.4335
1.53%
727.4609
1.08%
762.5587
9.05%
764.5658
1.19%
841.2556
3.47%
m/z (Da)

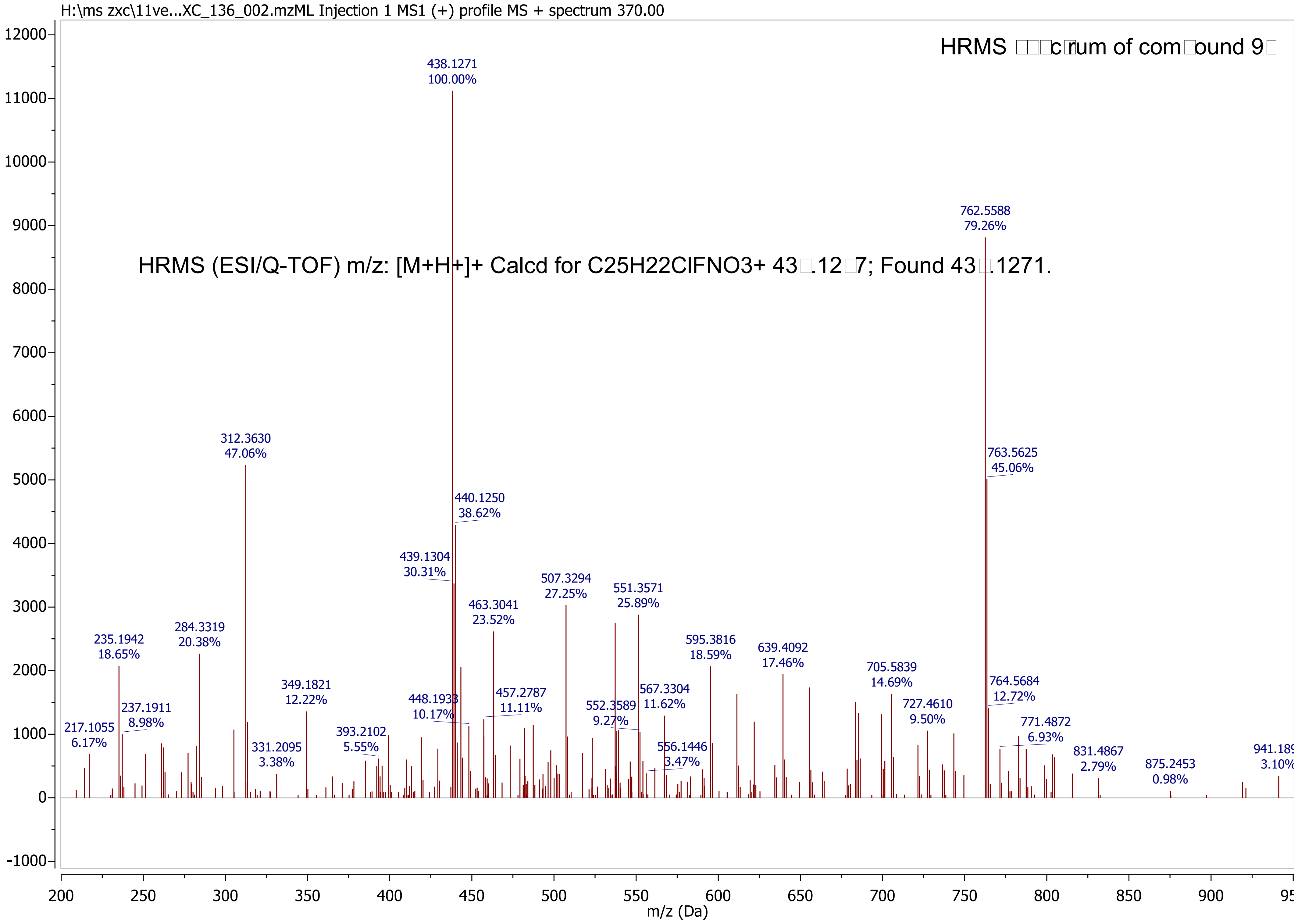
H:\ms zxc\11ve...XC_136_002.mzML Injection 1 MS1 (+) profile MS + spectrum 370.00
HRMS □□□c□rum of com□ound 9□
HRMS (ESI/Q-TOF) m/z: [M+H+]+ Calcd for C25H22ClFNO3+ 43□.12□7; Found 43□.1271.
438.1271 100.00%
762.5588 79.26%
312.3630 47.06%
763.5625 45.06%
440.1250 38.62%
439.1304 30.31%
507.3294 27.25%
551.3571 25.89%
463.3041 23.52%
284.3319 20.38%
235.1942 18.65%
595.3816 18.59%
639.4092 17.46%
705.5839 14.69%
764.5684 12.72%
349.1821 12.22%
567.3304 11.62%
457.2787 11.11%
448.1933 10.17%
727.4610 9.50%
552.3589 9.27%
237.1911 8.98%
771.4872 6.93%
217.1055 6.17%
393.2102 5.55%
556.1446 3.47%
331.2095 3.38%
941.189 3.10%
831.4867 2.79%
875.2453 0.98%
m/z (Da)

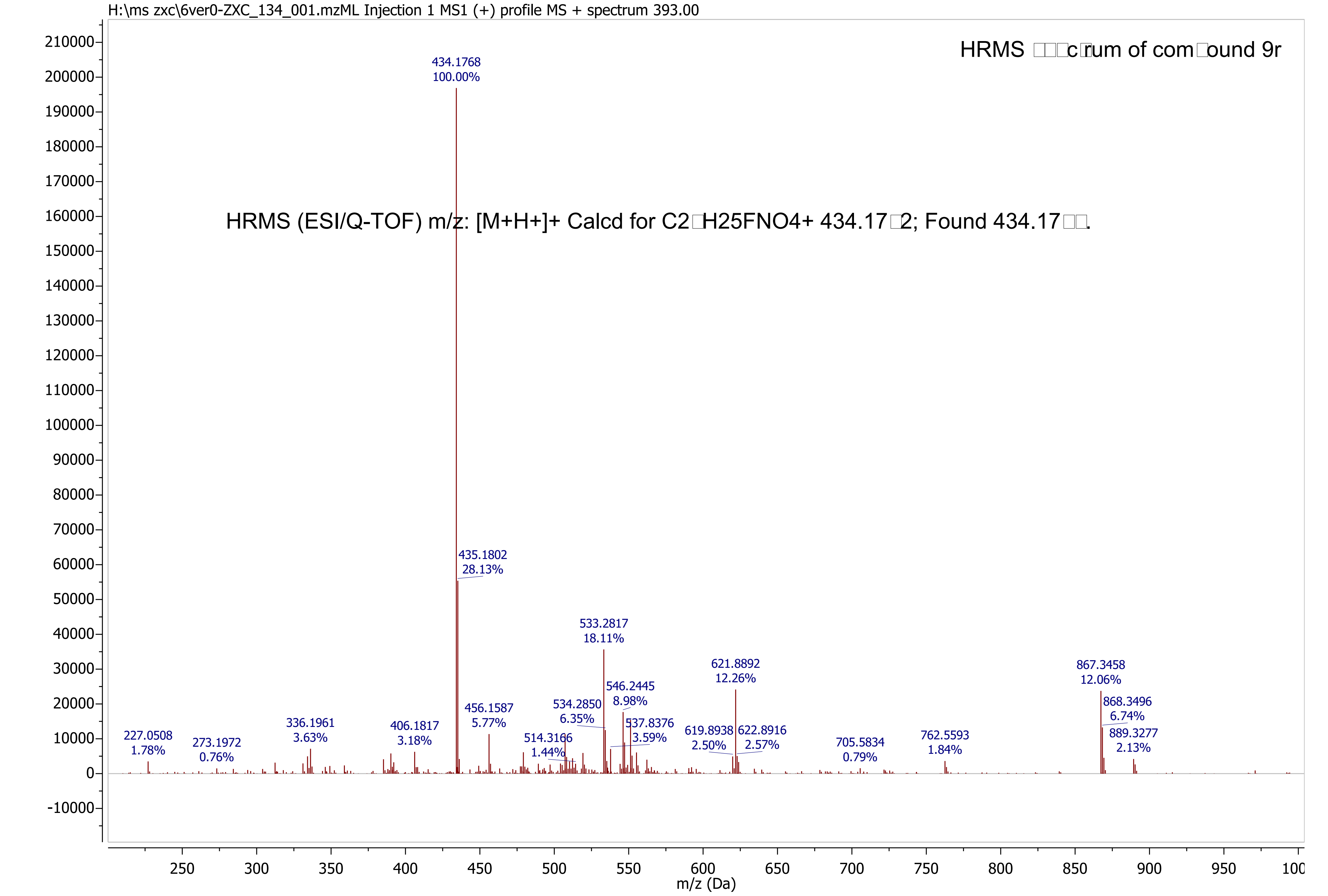
H:\ms zxc\6ver0-ZXC_134_001.mzML Injection 1 MS1 (+) profile MS + spectrum 393.00
HRMS □□□c□rum of com□ound 9r
HRMS (ESI/Q-TOF) m/z: [M+H+]+ Calcd for C2□H25FNO4+ 434.17□2; Found 434.17□□.
434.1768 100.00%
435.1802 28.13%
533.2817 18.11%
621.8892 12.26%
867.3458 12.06%
546.2445 8.98%
868.3496 6.74%
534.2850 6.35%
456.1587 5.77%
336.1961 3.63%
537.8376 3.59%
406.1817 3.18%
622.8916 2.57%
619.8938 2.50%
889.3277 2.13%
762.5593 1.84%
227.0508 1.78%
514.3166 1.44%
705.5834 0.79%
273.1972 0.76%
m/z (Da)

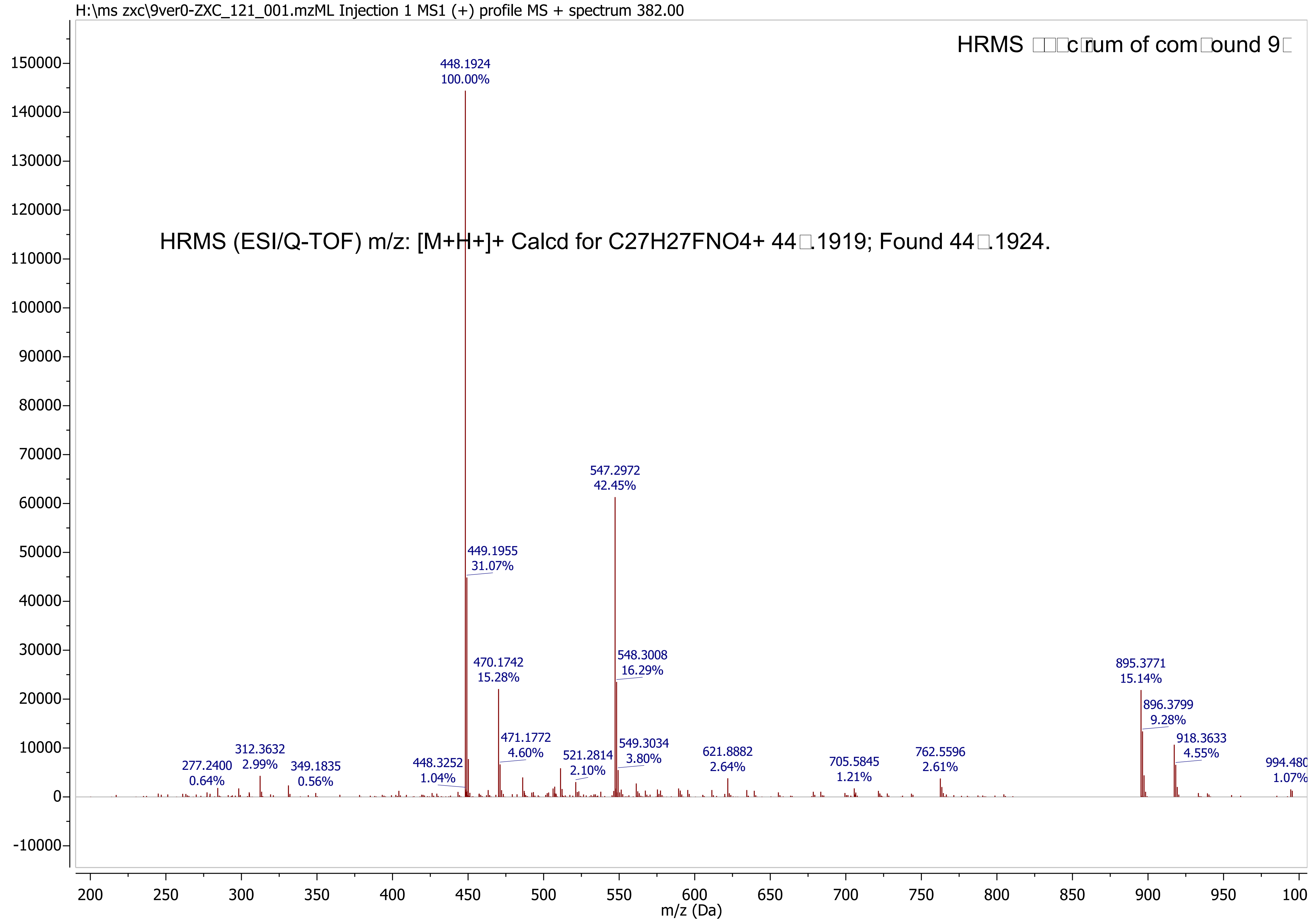
H:\ms zxc\9ver0-ZXC_121_001.mzML Injection 1 MS1 (+) profile MS + spectrum 382.00
HRMS □□□c□rum of com□ound 9□
HRMS (ESI/Q-TOF) m/z: [M+H+]+ Calcd for C27H27FNO4+ 44□.1919; Found 44□.1924.
448.1924 100.00%
449.1955 31.07%
547.2972 42.45%
470.1742 15.28%
548.3008 16.29%
895.3771 15.14%
896.3799 9.28%
471.1772 4.60%
549.3034 3.80%
918.3633 4.55%
312.3632 2.99%
277.2400 0.64%
349.1835 0.56%
448.3252 1.04%
521.2814 2.10%
621.8882 2.64%
705.5845 1.21%
762.5596 2.61%
994.480 1.07%
m/z (Da)

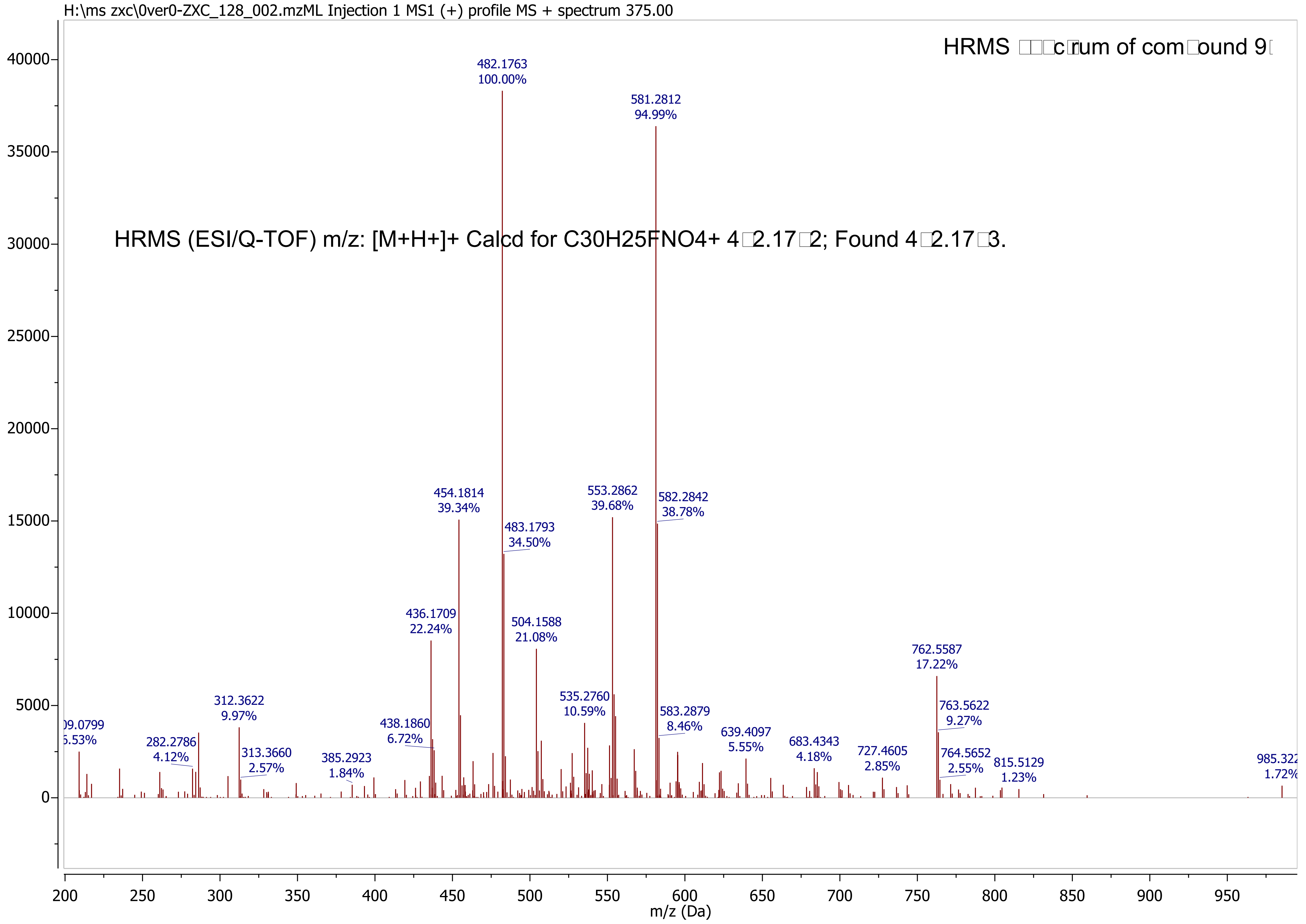
H:\ms zxc\0ver0-ZXC_128_002.mzML Injection 1 MS1 (+) profile MS + spectrum 375.00
HRMS [illegible]c[illegible]rum of com[illegible]ound 9[illegible]
HRMS (ESI/Q-TOF) m/z: [M+H+]+ Calcd for C30H25FNO4+ 4[illegible]2.17[illegible]2; Found 4[illegible]2.17[illegible]3.
482.1763 100.00%
581.2812 94.99%
454.1814 39.34%
553.2862 39.68%
582.2842 38.78%
483.1793 34.50%
436.1709 22.24%
504.1588 21.08%
762.5587 17.22%
535.2760 10.59%
312.3622 9.97%
763.5622 9.27%
583.2879 8.46%
438.1860 6.72%
639.4097 5.55%
282.2786 4.12%
313.3660 2.57%
385.2923 1.84%
683.4343 4.18%
727.4605 2.85%
764.5652 2.55%
815.5129 1.23%
m/z (Da)

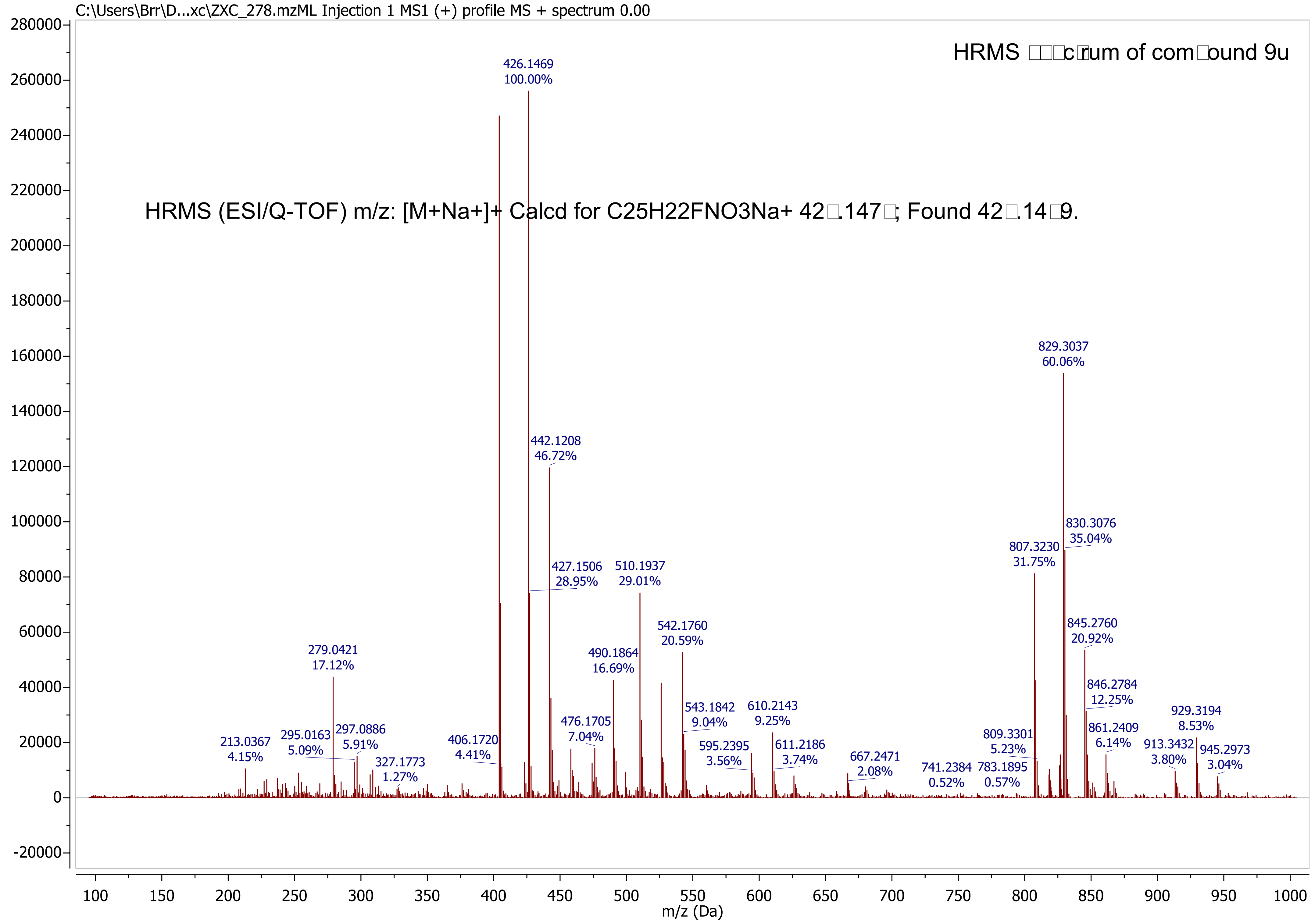

C:\Users\Brr\D...xc\ZXC_278.mzML Injection 1 MS1 (+) profile MS + spectrum 0.00
HRMS □□□c□rum of com□ound 9u
HRMS (ESI/Q-TOF) m/z: [M+Na+]+ Calcd for C25H22FNO3Na+ 42□.147□; Found 42□.14□9.
426.1469 100.00%
829.3037 60.06%
442.1208 46.72%
830.3076 35.04%
807.3230 31.75%
427.1506 28.95%
510.1937 29.01%
542.1760 20.59%
845.2760 20.92%
279.0421 17.12%
490.1864 16.69%
846.2784 12.25%
543.1842 9.04%
610.2143 9.25%
929.3194 8.53%
476.1705 7.04%
861.2409 6.14%
213.0367 4.15%
295.0163 5.09%
297.0886 5.91%
406.1720 4.41%
327.1773 1.27%
595.2395 3.56%
611.2186 3.74%
667.2471 2.08%
741.2384 0.52%
783.1895 0.57%
809.3301 5.23%
913.3432 3.80%
945.2973 3.04%
280000
260000
240000
220000
200000
180000
160000
140000
120000
100000
80000
60000
40000
20000
0
-20000
100 150 200 250 300 350 400 450 500 550 600 650 700 750 800 850 900 950 1000
m/z (Da)

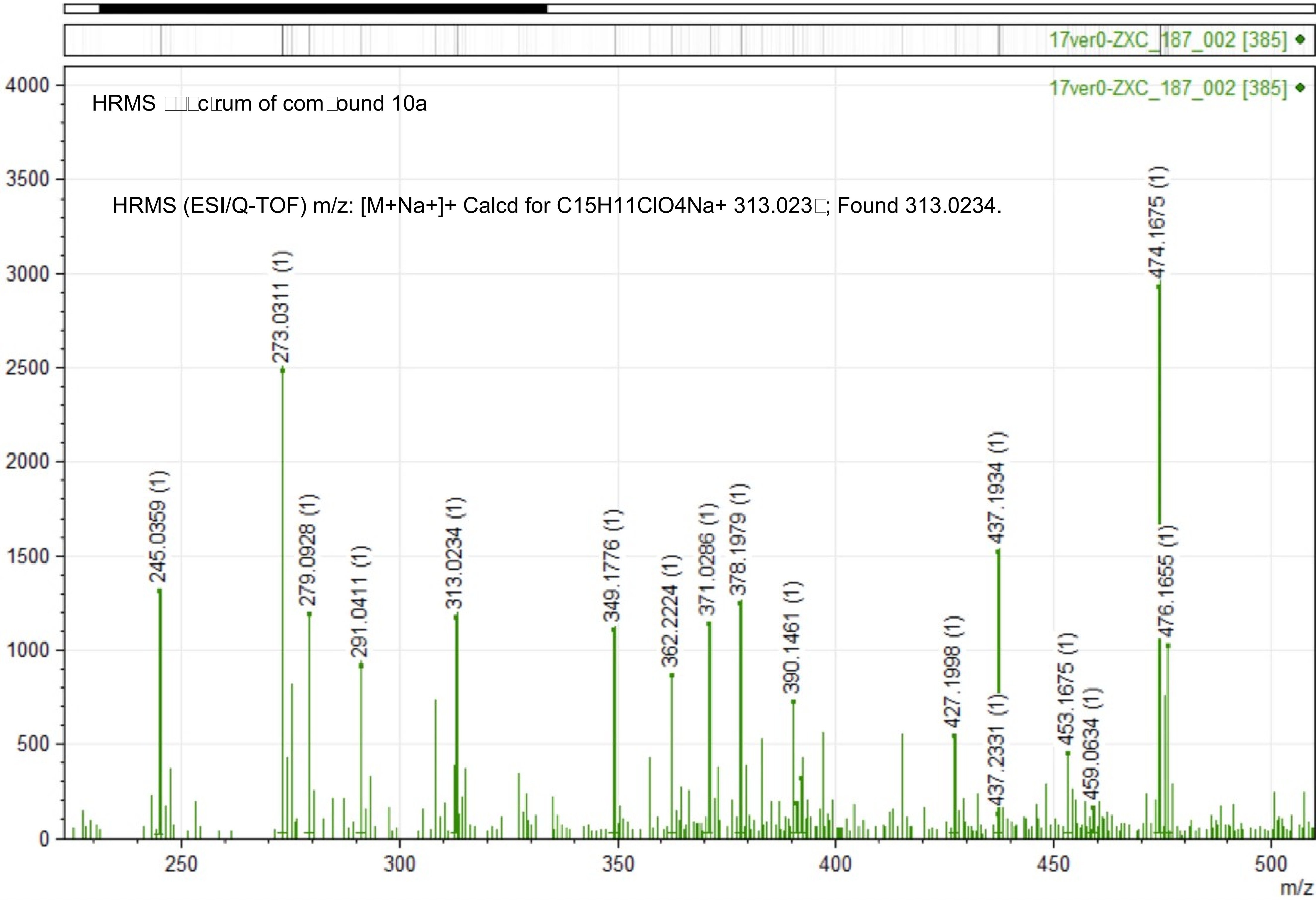
HRMS □□□c□rum of com□ound 10a
HRMS (ESI/Q-TOF) m/z: [M+Na+]+ Calcd for C15H11ClO4Na+ 313.023□; Found 313.0234.
17ver0-ZXC_187_002 [385]
245.0359 (1)
273.0311 (1)
279.0928 (1)
291.0411 (1)
313.0234 (1)
349.1776 (1)
362.2224 (1)
371.0286 (1)
378.1979 (1)
390.1461 (1)
427.1998 (1)
437.1934 (1)
437.2331 (1)
453.1675 (1)
459.0634 (1)
474.1675 (1)
476.1655 (1)
m/z

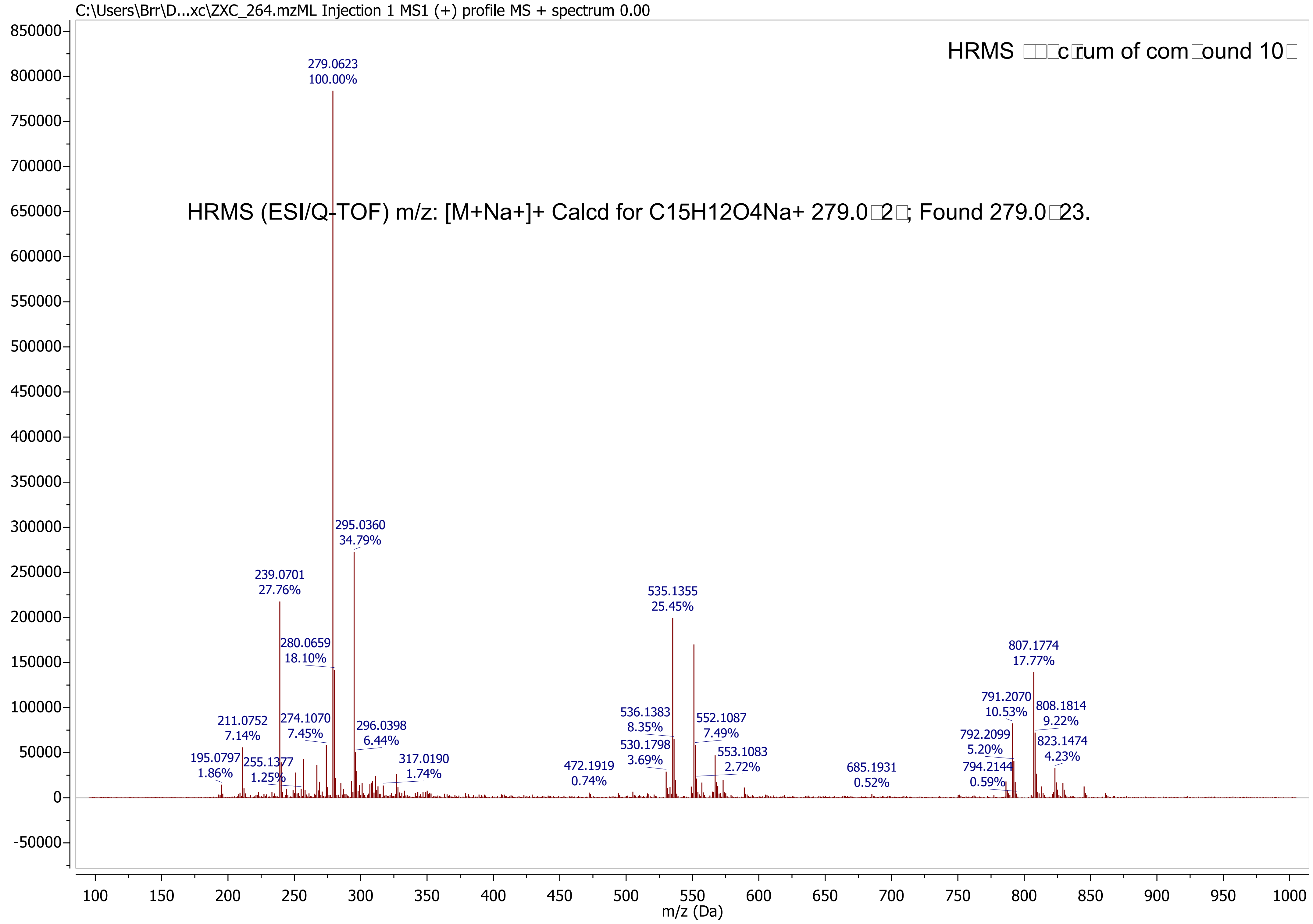
C:\Users\Brr\D...xc\ZXC_264.mzML Injection 1 MS1 (+) profile MS + spectrum 0.00
HRMS □□□c□rum of com□ound 10□
HRMS (ESI/Q-TOF) m/z: [M+Na+]+ Calcd for C15H12O4Na+ 279.0□2□; Found 279.0□23.
279.0623 100.00%
295.0360 34.79%
239.0701 27.76%
535.1355 25.45%
280.0659 18.10%
807.1774 17.77%
791.2070 10.53%
808.1814 9.22%
536.1383 8.35%
552.1087 7.49%
211.0752 7.14%
274.1070 7.45%
296.0398 6.44%
792.2099 5.20%
823.1474 4.23%
530.1798 3.69%
553.1083 2.72%
195.0797 1.86%
255.1377 1.25%
317.0190 1.74%
472.1919 0.74%
685.1931 0.52%
794.2144 0.59%
m/z (Da)

HRMS □□□c□rum of com□ound 10c

HRMS (ESI/Q-TOF) m/z: [M-H]- Calcd for C1□H13O5- 2□5.07□9; Found 2□5.07□□.

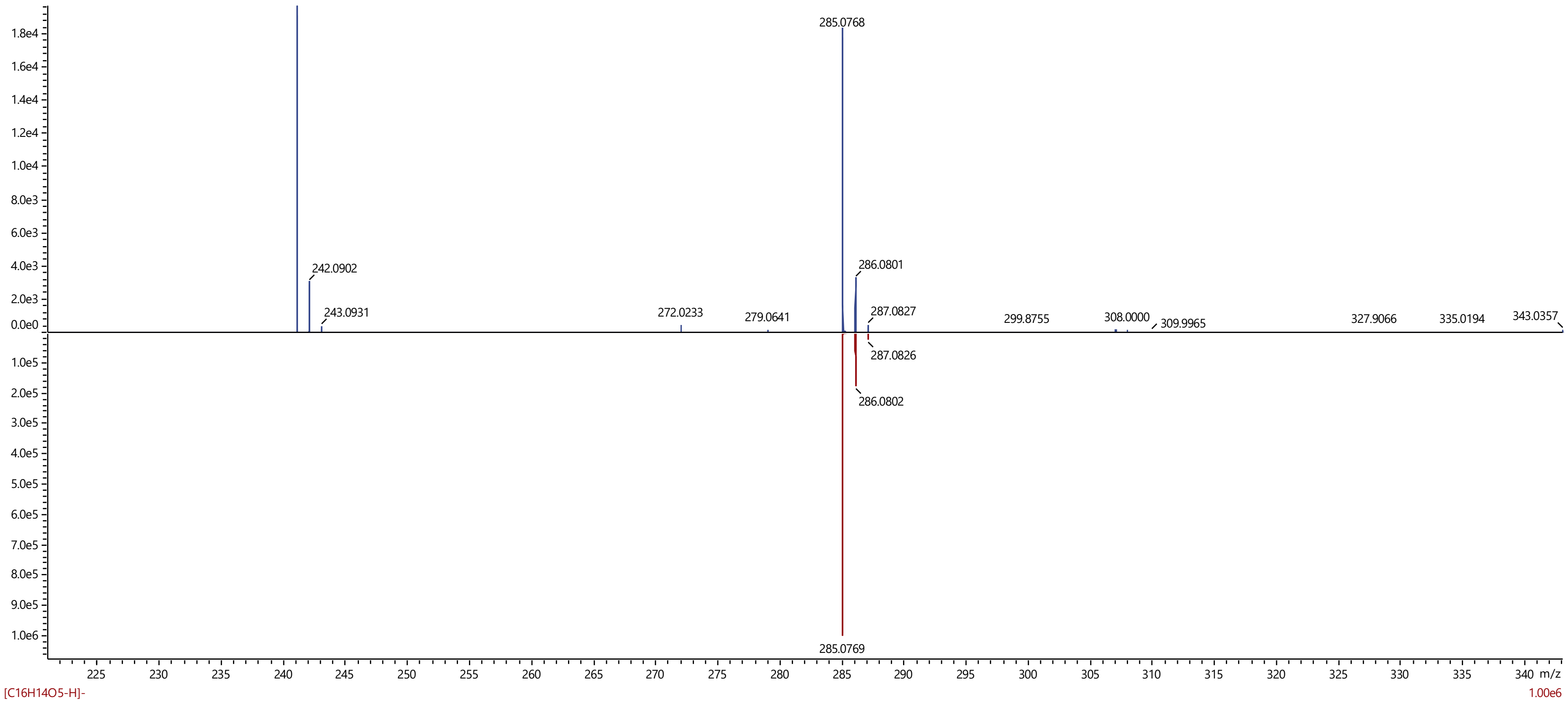

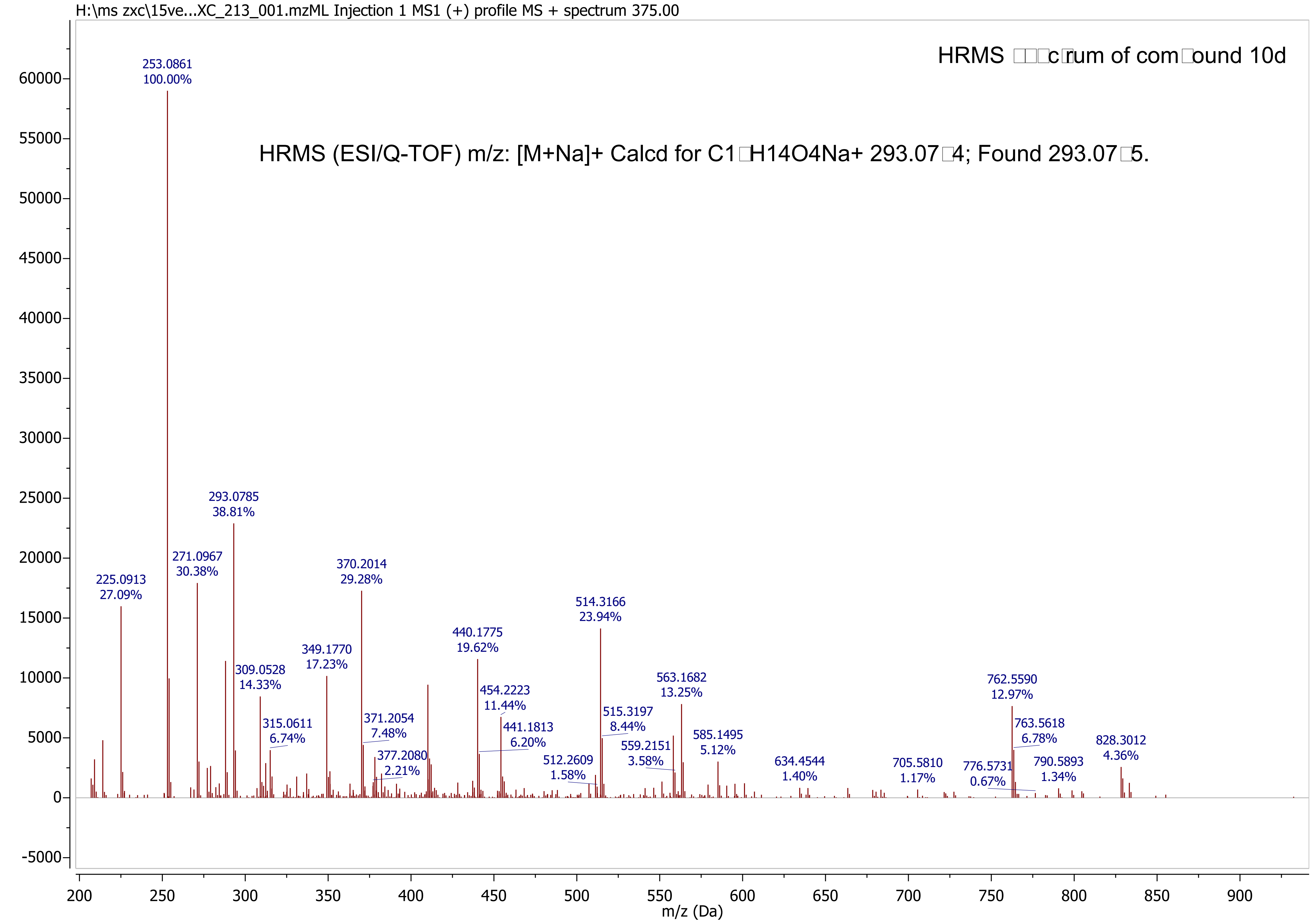
H:\ms zxc\15ve...XC_213_001.mzML Injection 1 MS1 (+) profile MS + spectrum 375.00
HRMS □□□c□rum of com□ound 10d
HRMS (ESI/Q-TOF) m/z: [M+Na]+ Calcd for C1□H14O4Na+ 293.07□4; Found 293.07□5.
253.0861 100.00%
293.0785 38.81%
271.0967 30.38%
370.2014 29.28%
225.0913 27.09%
514.3166 23.94%
440.1775 19.62%
349.1770 17.23%
309.0528 14.33%
563.1682 13.25%
762.5590 12.97%
454.2223 11.44%
515.3197 8.44%
371.2054 7.48%
763.5618 6.78%
315.0611 6.74%
441.1813 6.20%
585.1495 5.12%
828.3012 4.36%
559.2151 3.58%
377.2080 2.21%
512.2609 1.58%
634.4544 1.40%
790.5893 1.34%
705.5810 1.17%
776.5731 0.67%
60000
55000
50000
45000
40000
35000
30000
25000
20000
15000
10000
5000
0
-5000
200
250
300
350
400
450
500
550
600
650
700
750
800
850
900
m/z (Da)

C:\Users\Brr\D...xc\ZXC_255.mzML Injection 1 MS1 (+) profile MS + spectrum 0.00

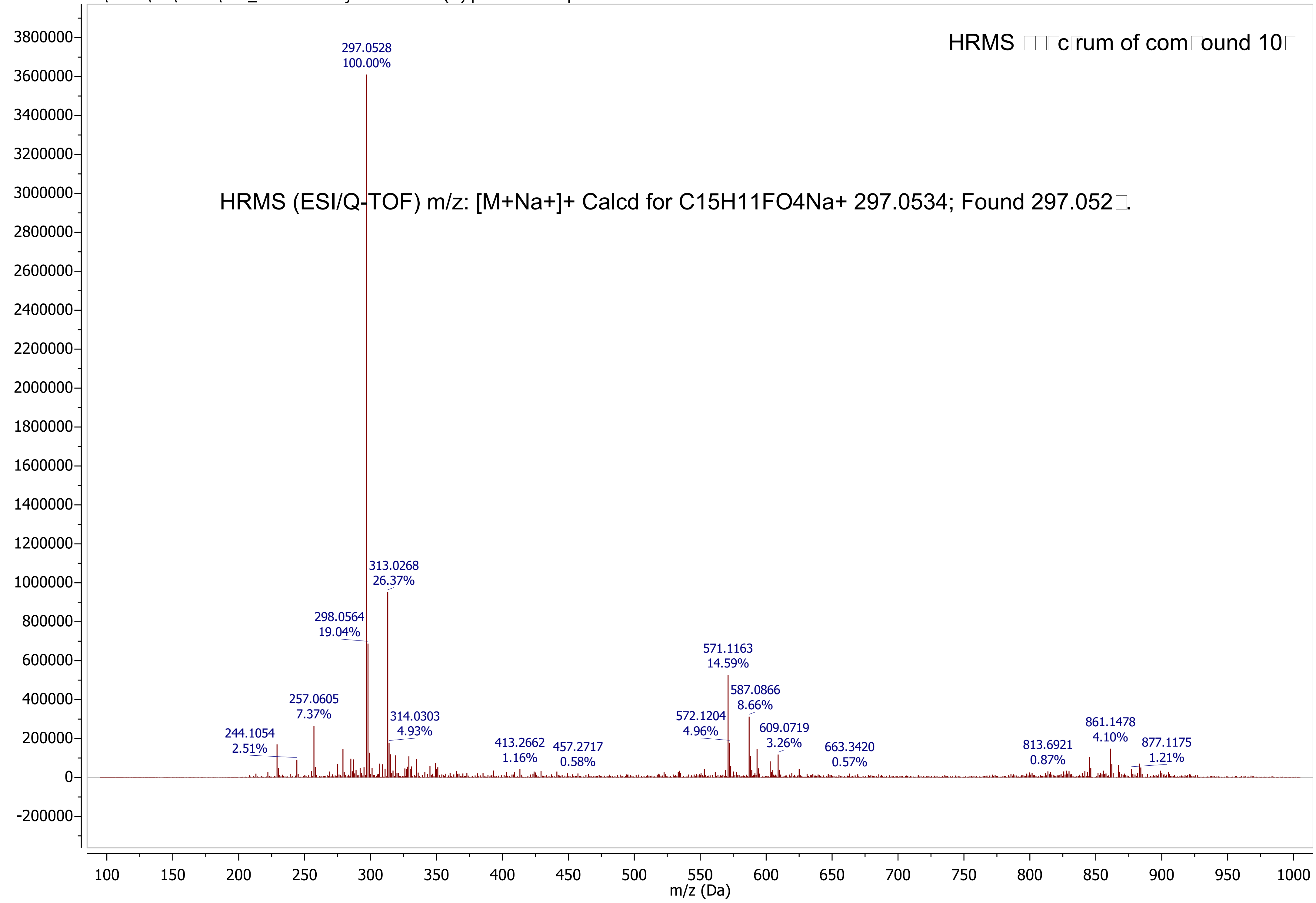

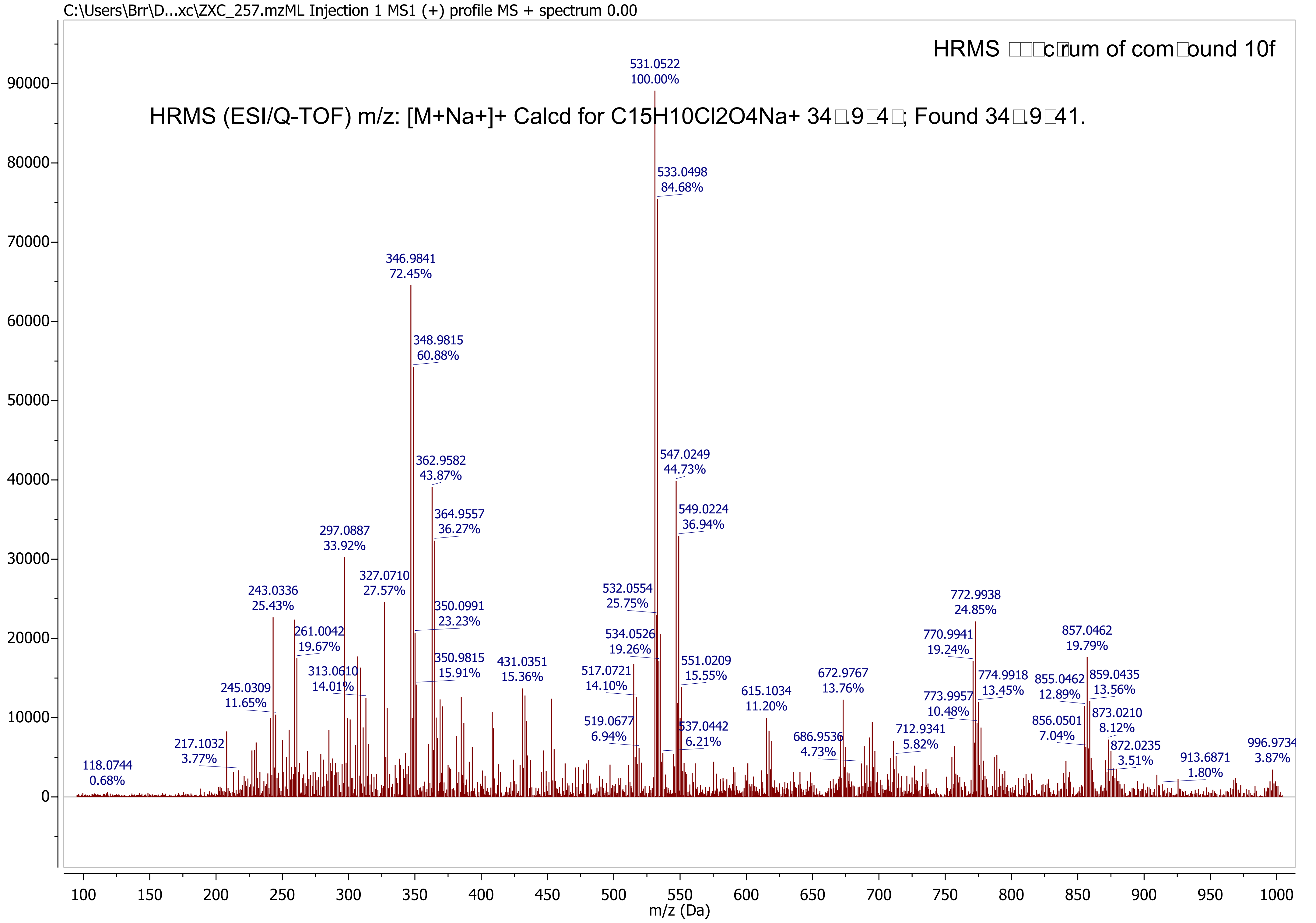
C:\Users\Brr\D...xc\ZXC_257.mzML Injection 1 MS1 (+) profile MS + spectrum 0.00
HRMS [illegible]c[illegible]rum of com[illegible]ound 10f
HRMS (ESI/Q-TOF) m/z: [M+Na+]+ Calcd for C15H10Cl2O4Na+ 34[illegible].9[illegible]4[illegible]; Found 34[illegible].9[illegible]41.
531.0522 100.00%
533.0498 84.68%
346.9841 72.45%
348.9815 60.88%
547.0249 44.73%
362.9582 43.87%
549.0224 36.94%
364.9557 36.27%
297.0887 33.92%
327.0710 27.57%
532.0554 25.75%
243.0336 25.43%
772.9938 24.85%
350.0991 23.23%
857.0462 19.79%
261.0042 19.67%
534.0526 19.26%
770.9941 19.24%
350.9815 15.91%
551.0209 15.55%
431.0351 15.36%
517.0721 14.10%
313.0610 14.01%
672.9767 13.76%
859.0435 13.56%
774.9918 13.45%
855.0462 12.89%
245.0309 11.65%
615.1034 11.20%
773.9957 10.48%
873.0210 8.12%
856.0501 7.04%
519.0677 6.94%
537.0442 6.21%
712.9341 5.82%
686.9536 4.73%
996.9734 3.87%
217.1032 3.77%
872.0235 3.51%
913.6871 1.80%
118.0744 0.68%
m/z (Da)

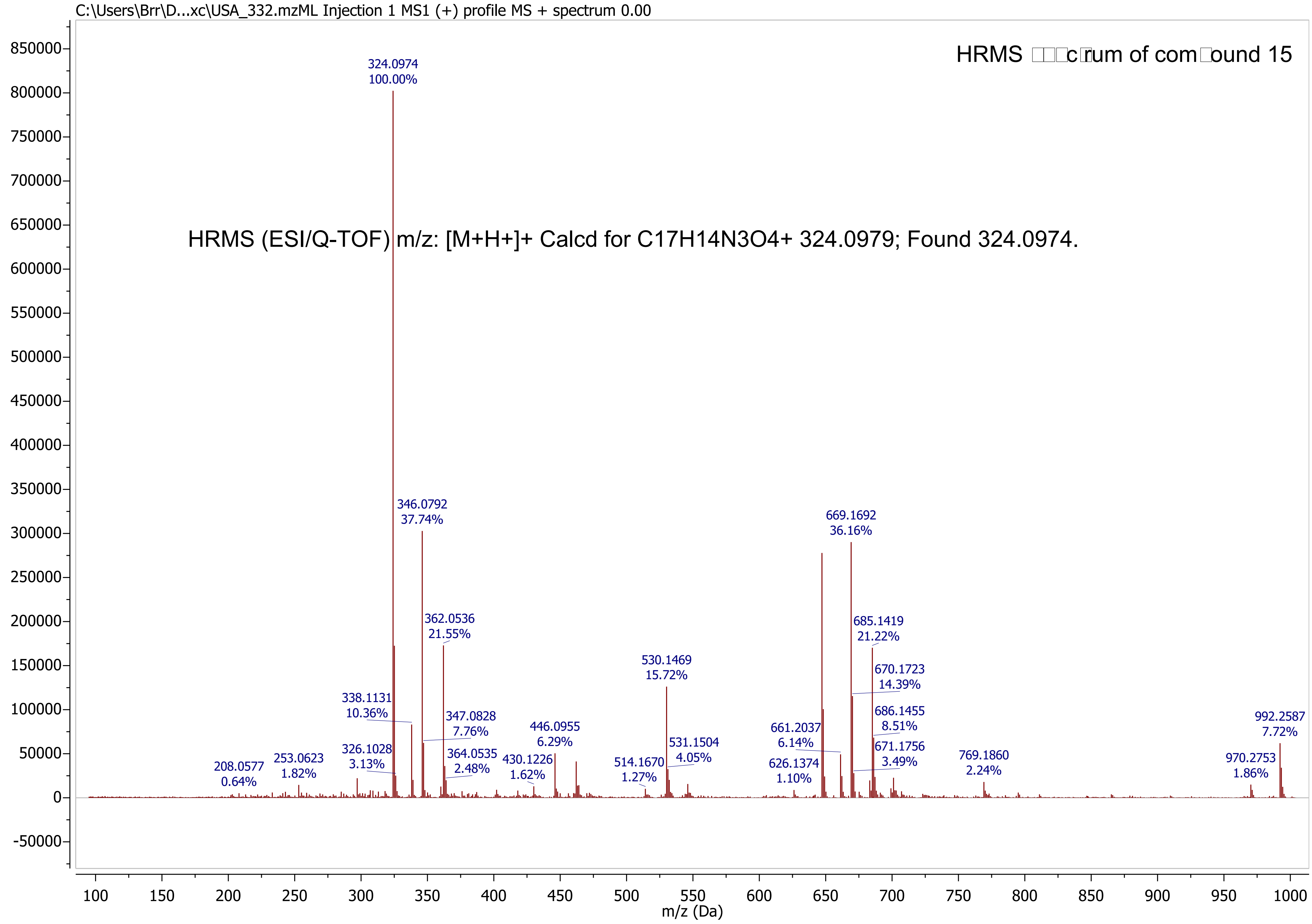

C:\Users\Brr\D...xc\USA_332.mzML Injection 1 MS1 (+) profile MS + spectrum 0.00
HRMS (ESI/Q-TOF) m/z: [M+H+]+ Calcd for C17H14N3O4+ 324.0979; Found 324.0974.
324.0974 100.00%
346.0792 37.74%
669.1692 36.16%
362.0536 21.55%
685.1419 21.22%
530.1469 15.72%
670.1723 14.39%
338.1131 10.36%
686.1455 8.51%
347.0828 7.76%
992.2587 7.72%
446.0955 6.29%
661.2037 6.14%
531.1504 4.05%
671.1756 3.49%
326.1028 3.13%
364.0535 2.48%
769.1860 2.24%
970.2753 1.86%
253.0623 1.82%
430.1226 1.62%
514.1670 1.27%
626.1374 1.10%
208.0577 0.64%
m/z (Da)

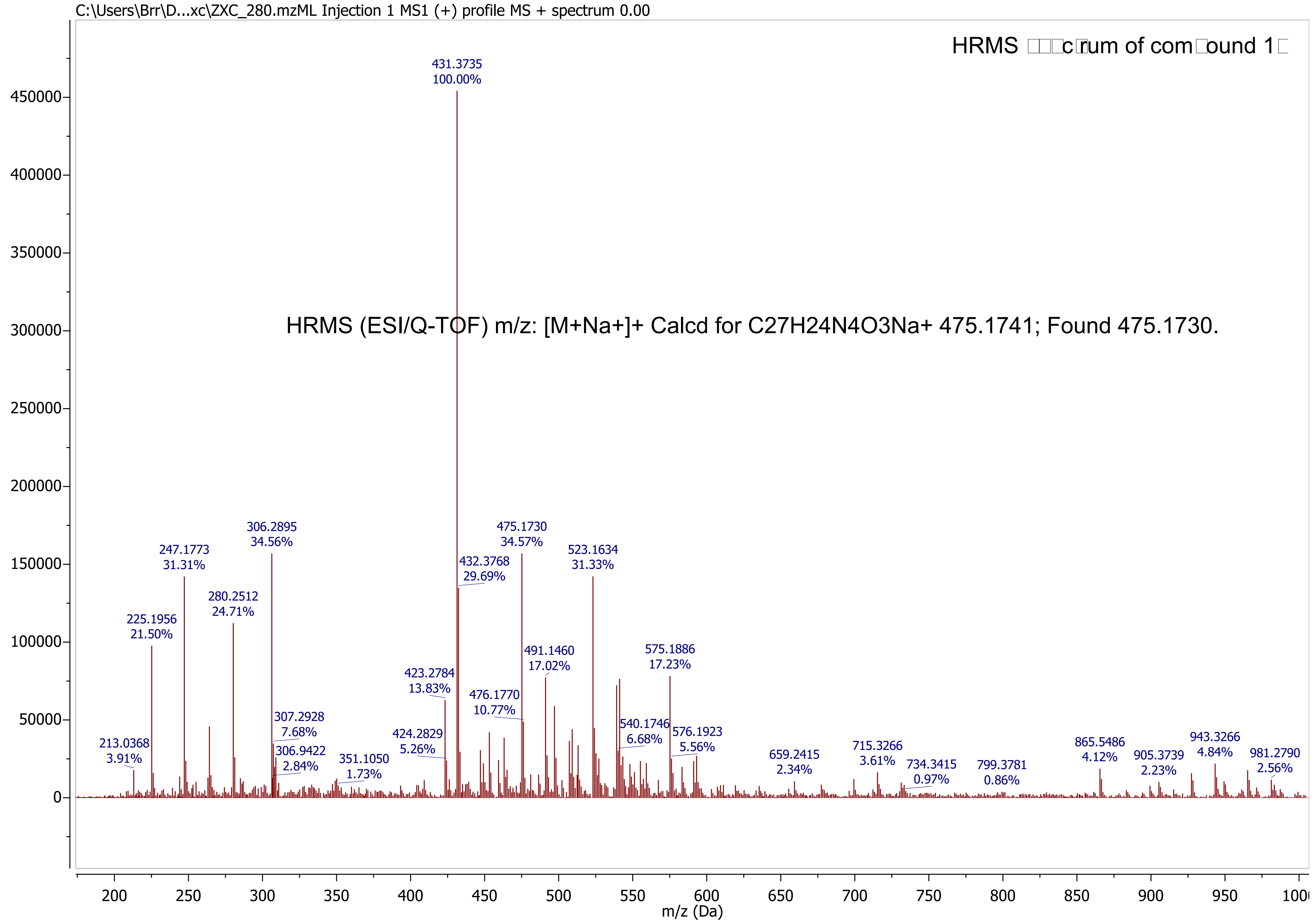
C:\Users\Brr\D...xc\ZXC_280.mzML Injection 1 MS1 (+) profile MS + spectrum 0.00
HRMS (ESI/Q-TOF) m/z: [M+Na+]+ Calcd for C27H24N4O3Na+ 475.1741; Found 475.1730.
431.3735 100.00%
306.2895 34.56%
475.1730 34.57%
247.1773 31.31%
523.1634 31.33%
432.3768 29.69%
280.2512 24.71%
225.1956 21.50%
491.1460 17.02%
575.1886 17.23%
423.2784 13.83%
476.1770 10.77%
307.2928 7.68%
540.1746 6.68%
576.1923 5.56%
424.2829 5.26%
213.0368 3.91%
306.9422 2.84%
351.1050 1.73%
659.2415 2.34%
715.3266 3.61%
734.3415 0.97%
799.3781 0.86%
865.5486 4.12%
905.3739 2.23%
943.3266 4.84%
981.2790 2.56%
m/z (Da)
